# Photochemical deracemization of 2,3-allenoic acids mediated by a sensitizing chiral phosphoric acid catalyst

**DOI:** 10.1039/d5sc05356k

**Published:** 2025-09-22

**Authors:** Max Stierle, Daniel Bitterlich, Julia Westermayr, Thorsten Bach

**Affiliations:** a Technische Universität München, School of Natural Sciences, Department of Chemistry and Catalysis Research Center Lichtenbergstrasse 4 85747 Garching Germany thorsten.bach@ch.tum.de https://www.ch.nat.tum.de/en/oc1/home/; b Leipzig University, Wilhelm-Ostwald-Institute for Physical and Theoretical Chemistry Linnéstraße 2 04103 Leipzig Germany; c Center for Scalable Data Analytics and Artificial Intelligence (ScaDS.AI) Leipzig/Dresden, Humboldtstraße 25 04105 Leipzig Germany

## Abstract

Photochemistry opens the possibility to convert a racemic mixture of a chiral compound into a distinct enantiomer in a single operation. Seven chiral phosphoric acids with a pendant thioxanthone chromophore were synthesized and evaluated as catalysts for the visible light-driven deracemization of 2,4-disubstituted 2,3-allenoic acids. A catalyst derived from 1,1′-spirobiindane-7,7′-diol (spinol) performed best and delivered 2-benzyl-4-*tert*-butyl-2,3-allenoic acids in 58–97% yield and with enantiomeric ratios (e.r.) varying between 71/29 and 85/15 depending on the benzyl substitution pattern. The major enantiomer was shown to be (*R*)-configured, and other 2,3-allenoic acids were also probed in the reaction. A mechanistic scenario for the observed enantioselectivity is provided that rests on experimental and quantum-chemical studies.

## Abstract 2

A phosphoric acid with a 1,1′-spirobiindane-7,7′-diol (spinol) backbone and two pendant thioxanthone groups was found to promote the photochemical deracemization of 2,3-allenoic acids.

## Graphical abstract

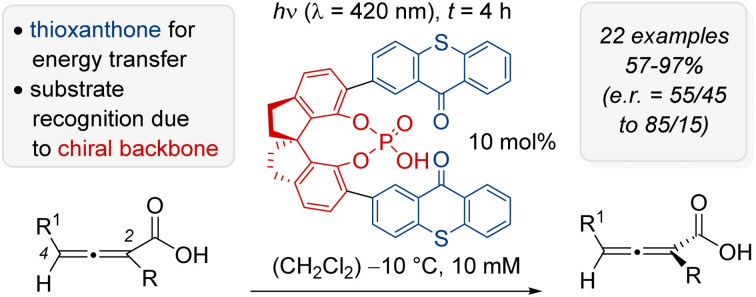

## Introduction

Since racemates of chiral compounds are typically available at a lower cost than enantiomerically pure compounds, extensive efforts have been undertaken to resolve racemic mixtures. A wide arsenal of separation techniques has become available to obtain the two enantiomers of a given chiral compound with high purity.^1^ Alternatively, it is possible to differentiate two enantiomers in a racemic mixture by kinetic resolution. Typically, chiral catalysts, often enzymes,^2^ are employed to involve one enantiomer in a chemical transformation while the other enantiomer remains unchanged. For the preparation of enantiopure carboxylic acid derivatives, a kinetic resolution reaction frequently involves either an esterification or an ester hydrolysis.^3^ Since ester and carboxylic acid are easily interconvertible, the process can be iterated, eventually leading to a single enantiomer. Alternatively, kinetic resolution reactions can be performed under conditions which allow for an equilibration of the two enantiomers (dynamic kinetic resolution),^4^ thus facilitating complete conversion of a racemic mixture into an enantioenriched or enantiopure product.

**Scheme 1:**
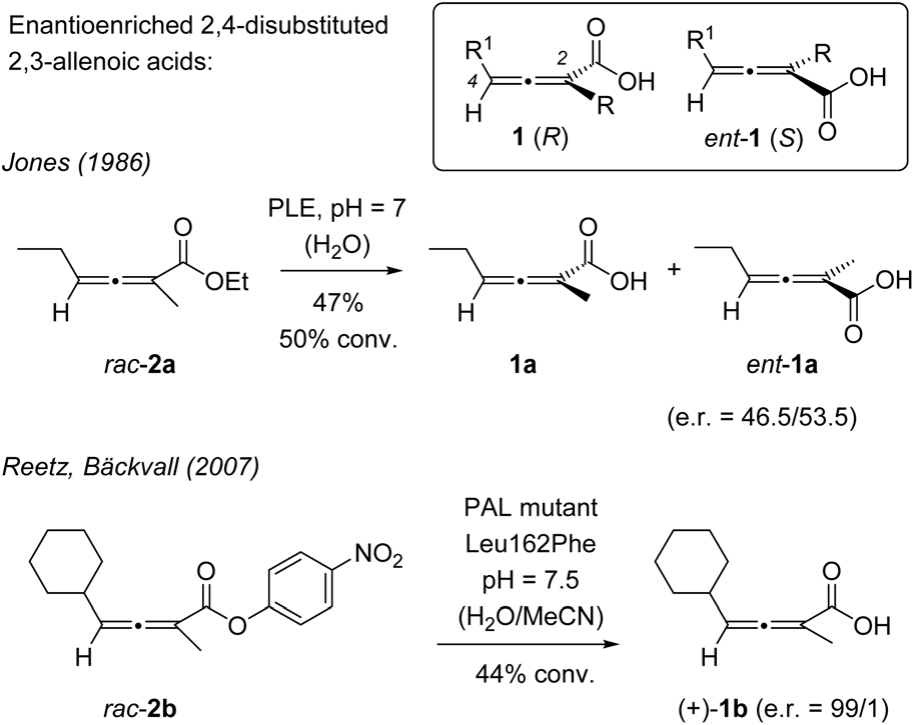
Enantioenriched 2,4-disubstituted 2,3-allenoic acids (1) obtained by enzymatic kinetic resolution of racemic esters 2. PLE = pig liver esterase. PAL = *Pseudomonas aeruginosa* lipase.

Allenes (1,2-propadienes) display an axis of chirality if they carry two sets of different substituents at their terminal carbon atoms. The compound class has received great interest due to its high synthetic utility and its potential for creating chiral structures with a defined absolute configuration.^5^ In the latter context, 2,4-disubstituted 2,3-allenoic acids invite kinetic resolutions by enzyme catalysis starting from an ester precursor. Pioneering work by Jones and co-workers^6^ revealed that enantioenriched 2,3-allenoic acids 1 can be obtained by hydrolysis of the respective allenoate (Scheme 1). Ethyl ester *rac*-2a was reported to deliver the (*S*)-configured allenoic acid in a low, yet detectable enantiomeric ratio (e.r.) of 53.5/46.5.

While subsequent work with wild-type enzymes delivered improved results,^7^ major breakthroughs were not achieved before directed evolution was applied to allenoate hydrolysis. The Reetz and the Bäckvall laboratories engineered a lipase by site-specific mutagenesis which processes almost exclusively one enantiomer of ester *rac*-2b to provide product (+)-1b with an excellent e.r.^8^ Although a method for the racemization of allenoates had been previously reported,^9^ a dynamic kinetic resolution was to the best of our knowledge never applied to the preparation of enantioenriched 2,4-disubstituted 2,3-allenoic acids. Despite the development of highly powerful alternative kinetic resolution methods,^10,11^ there is no direct access to this compound class that allows for a complete conversion of the respective racemate to a single enantiomer.

Given our interest in photochemical deracemization reactions of allenes and related axially chiral compounds,^12,13^ we considered possible catalyst designs to be applicable to chiral 2,3-allenoic acids. Deracemization promises the complete conversion of a racemic mixture to a given enantiomer under photochemical conditions.^14^ A suitable photocatalyst requires a binding motif for the carboxylic acid group and needs to display chromophores that induce the desired deracemization event. From previous work, it was known that thioxanthones are competent to facilitate promotion of allenes into their triplet state,^12*a*,*c*^ which in turn suggested that a catalyst with suitably placed thioxanthones^15^ might enable the required differen-tiation of enantiomeric starting materials (*vide infra*). We now report on the synthesis of new chiral phosphoric acid-based^16^ thioxanthone catalysts^17,18^ and their application to the photochemical deracemization of 2,4-disubstituted 2,3-allenoic acids (1). A catalyst derived from 1,1′-spirobiindane-7,7′-diol (spinol) evolved as best suited for this purpose, and its mode of action was studied in more detail by mechanistic and quantum-chemical investigations.

## Results and discussion

**Scheme 2:**
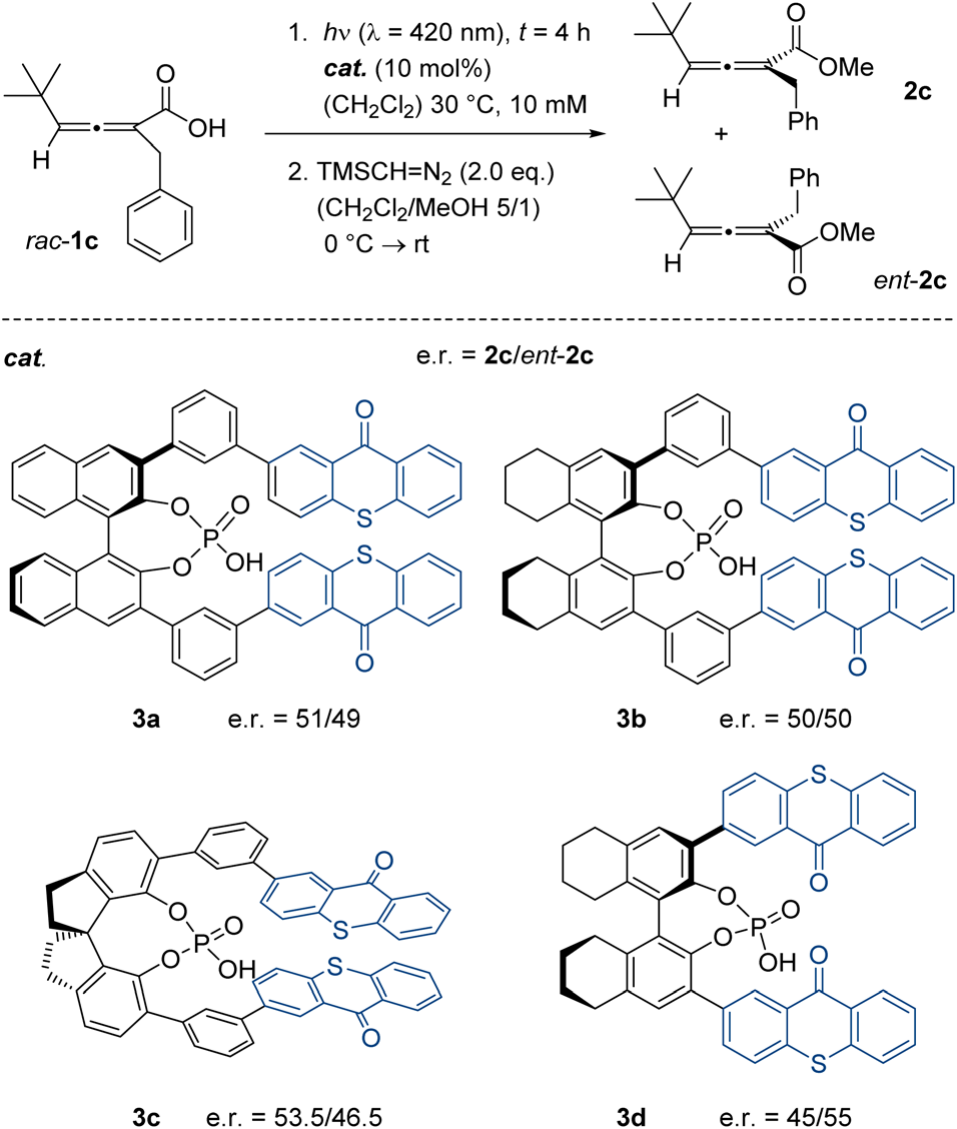
Photochemical deracemization attempts performed with 2,3-allenoic acid *rac*-1c and known catalysts 3a–3d. The acid was methylated to determine the enantiomeric ratio (e.r.) by chiral HPLC analysis.

Preliminary experiments were performed with chiral allenoic acid *rac*-1c, which was irradiated with light of fluorescent lamps at an emission maximum of *λ* = 420 nm (for details see the SI). At this wavelength, the allenoic acid is transparent, while thioxanthones typically absorb visible light in the blue region of the electromagnetic spectrum (*λ* ≤ 450 nm). Since separation of the two enantiomers of *rac*-1c by chiral HPLC was not feasible, the acid was converted into its methyl ester by treatment with trimethylsilyldiazomethane (TMS = trimethylsilyl). The methyl ester enantiomers 2c and *ent*-2c were separable and allowed for an assessment of their relative ratio by chiral HPLC (Scheme 2). Initial experiments were performed with previously reported chiral phosphoric acids, which were derived from 1,1′-bi-2-naphthol (binol, 3a),^18^ 5,5′,6,6′,7,7′,8,8′-octahydro-1,1′-bi-2-naphthol (octahydrobinol, 3b and 3d)^19^ or 1,1′-spirobiindane-7,7′-diol (spinol, 3c).^19^ At first sight, the outcome of the reactions was disappointing because there was no significant preference for one of the two enantiomers. Even prolonged irradiation did not lead to an improvement. On the positive side, we noted that the spinol-based catalyst 3c outperformed the related catalysts 3a and 3b, and that it seemed beneficial to move the thioxanthone chromophore closer to the chiral backbone. In catalysts 3a–3c, a *meta*-substituted phenyl group acts as a hinge connecting the chromophore with the phosphoric acid, while the chromophore is directly attached to the chiral backbone in catalyst 3d. The results indicated that it might be beneficial to prepare new spinol-based catalysts to which thioxanthone was directly attached at the 6- and 6′-positon of the biindane skeleton.

**Fig. 1:**
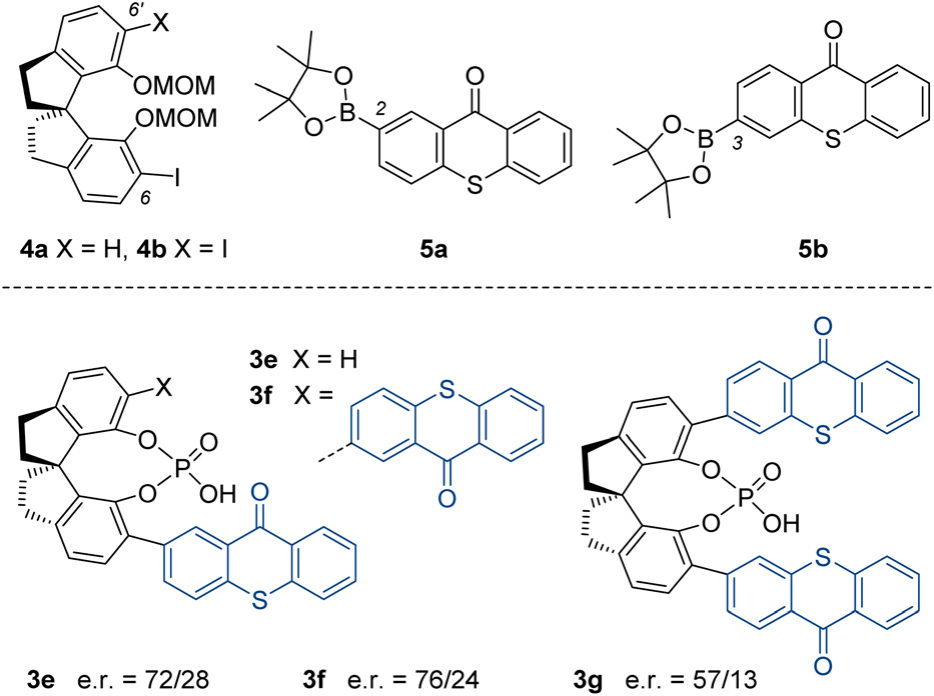
Starting materials 4–5 (top) required for the synthesis of spinol-based chiral phosphoric acids 3e–3g. The e.r. refers to the enantiomeric ratio obtained with the respective catalyst in the photochemical deracemization of 2,3-allenoic acid *rac*-1c (*cf.*Scheme 2).

The synthesis of the spinol derivatives commenced with the respective mono- or diiodinated precursors 4a ^20^ and 4b,^21^ the hydroxy groups of which were protected by methoxymethyl (MOM) substituents (Fig. 1). Subsequent Pd-catalysed cross-coupling with known boronates 5a^17*c*^ and 5b^22^ was immediately followed by removal of the MOM protecting groups and formation of the respective phosphates 3e–3g.

Catalyst 3e was obtained in an overall yield of 52%. The reaction to the 6,6′-disubstituted catalysts 3f (48% overall) and 3g (72% overall) turned out to be equally efficient, but the separation from impurities proved tedious in all cases. The performance of the catalysts notably improved when compared to the previous results obtained with catalysts 3a–3d in the photochemical deracemization. Particularly, the two-fold thioxanthone-substituted catalyst 3f combined a good enantiodiscrimination in the reaction of chiral acid *rac*-1c (e.r. = 76/24) with good availability on larger scale. Further optimisation experiments were consequently performed with the latter catalyst (Table 1). By choosing different reaction times (entries 1–4), it was confirmed that the photostationary state was reached after an irradiation time of four hours. There was no improvement of the e.r. at longer reaction times.

**Table 1:** Reaction optimisation for the photochemical deracemization of 2,3-allenoic acid *rac*-1c catalyzed by chiral Brønsted acid 3f (*cf.*Scheme 2 and Fig. 1)

entry^a^	3f [mol%]	Solvent	*T* [°C]	*t* [h]	Yield	e.r^b^
1	10	CH_2_Cl_2_	30	4	Quant	76/24
2	10	CH_2_Cl_2_	30	2	Quant	74/26
3	10	CH_2_Cl_2_	30	6^e^	Quant	75/25
4	10	CH_2_Cl_2_	30	22	78%	70/30
5^c^	10	CH_2_Cl_2_	30	4	88%	74/26
6^d^	10	CH_2_Cl_2_	30	4	93%	76/24
7	5	CH_2_Cl_2_	30	4	Quant	73/27
8	20	CH_2_Cl_2_	30	4	96%	74/26
9	10	CH_2_Cl_2_	−10	4	97%	82/18
10	10	CH_2_Cl_2_	−40	4	Quant	80/20
11^e^	10	CH_2_Cl_2_	−10	4	97	79/21
12	10	DCE^f^	30	4	96%	58/42
13	10	Toluene	30	4	Quant	65/35
14	10	Acetone	30	4	97%	51/49
15	10	TFT^g^	30	4	89%	72/28
16	10	HFX^h^	30	4	87%	75/25

a Unless otherwise noted, the reactions were performed at a substrate concentration of *c* = 10 mM. The product was isolated as its methyl ester 2c/*ent*-2c.

b The enantiomeric ratio (e.r.) was determined after methylation by chiral-phase HPLC analysis.

c *c* = 5 mM.

d *c* = 20 mM.

e The irradiation was performed at *λ* = 440 nm.

f 1,2-Dichloroethane.

g Trifluorotoluene.

h 1,3-Bis(trifluoromethyl)benzene.

The influence of the substrate concentration (*c*) was probed by changing the concentration to *c* = 5 mM (entry 5) and *c* = 20 mM (entry 6), respectively. There was no notable change which is why a concentration of *c* = 10 mM was routinely chosen. Likewise the catalyst loading (entries 7, 8) had little influence on the reaction outcome and the loading was kept at 10 mol%. The most significant parameter leading to an improvement of the enantioselectivity was the reaction temperature. At −10 °C, the e.r. increased to 82/18 (entry 9) but there was no further increase upon lowering the temperature to −40 °C (entry 10). Since the fluorescent light bulbs display a relative broad emission spectrum, other light sources with a narrower emission band were tested. One example is included in Table 1 (entry 11). Here, a light emitting diode (LED) with an emission maximum at *λ* = 440 nm was used. The outcome was somewhat inconsistent, possible due to the little overlap between emission and absorption by the catalyst (*vide infra*). On average, neither yield nor e.r. improved notably when choosing other light sources (see the SI for details). The choice of solvent was limited by the solubility of the chiral Brønsted acid. All tested solvents (entries 12–16) turned out to be inferior to dichloromethane, and we selected the conditions of entry 9 to study different substrates in photochemical deracemization reactions mediated by catalyst 3f.

**Scheme 3:**
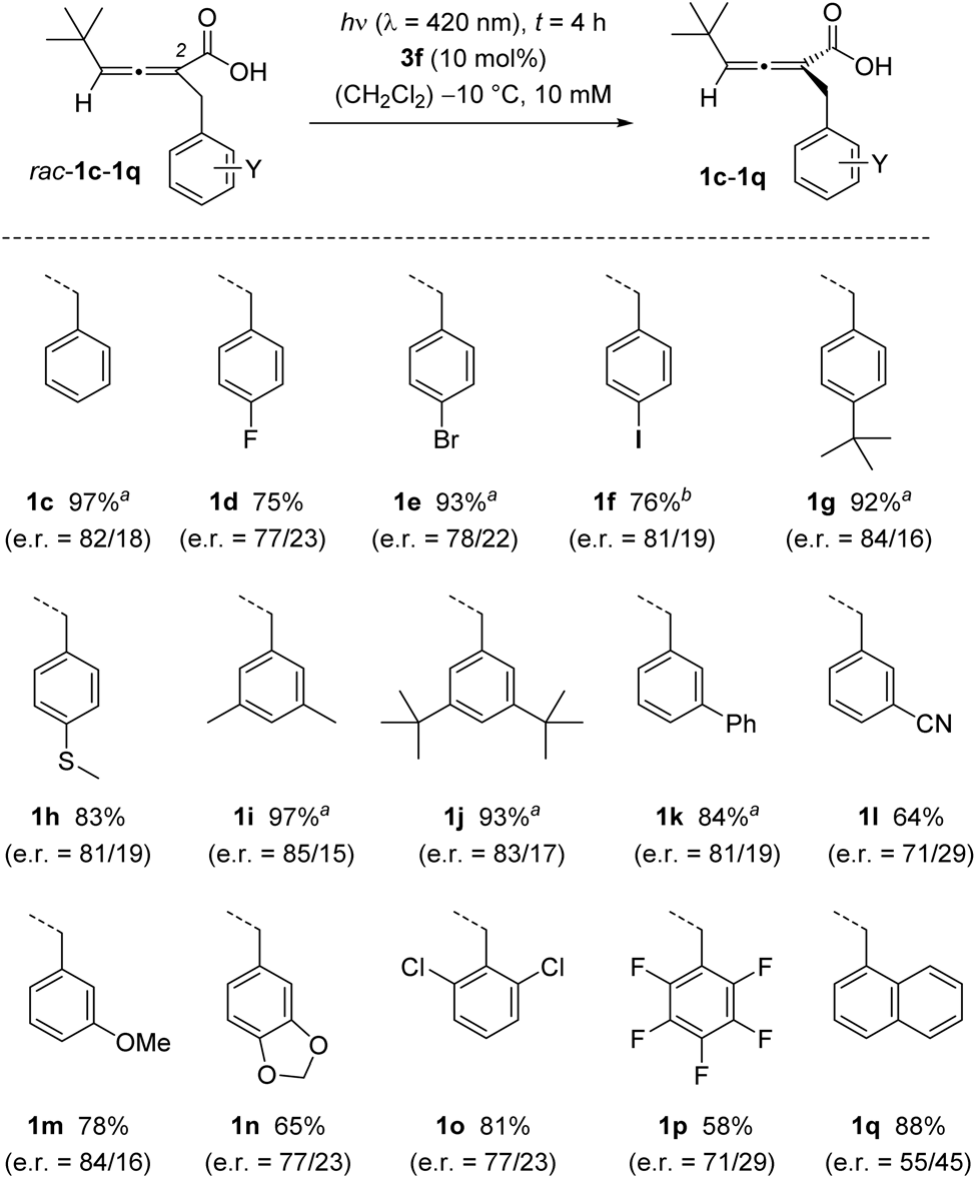
Photochemical deracemization of 2,3-allenoic acids *rac*-1c to *rac*-1q as catalysed by chiral Brønsted acid 3f (50 μmol scale). ^*a*^ Yield and e.r. of the product were obtained after methyl ester formation as described in Scheme 2. ^*b*^ When performed on 0.5 mmol scale the yield was 94% (e.r. = 80/20).

Initial substrate modifications concerned primarily the substituent in 2-position. Diversely substituted phenyl groups were probed at the benzylic position and it was shown that the protocol was compatible with alkyl (1g, 1i, 1j), fluoro (1d), chloro (1o), bromo (1e), iodo (1f), alkylsulfanyl (1h), phenyl (1k), cyano (1l) and alkoxy (1m) substituents (Scheme 3). In some cases, the ratio of the acid enantiomers could not be directly assessed, and the acid was converted into the respective methyl ester. Yields were consistently high but decreased for strongly electron deficient (1p) and electron rich (1n) substituents.

Although steric bulk at the 2-benzyl substituent did not change the enantioselectivity of the deracemization, as seen for example in the reaction of *rac*-1j, the 1-naphthyl group led to a notable decrease in e.r. (product 1q). It was possible to scale-up the reaction, and an improved yield was recorded when running the reactions on larger scale. For instance, acid 1f was obtained in 94% yield when the reaction was run on 0.5 mmol scale while the small-scale reaction (50 μmol) gave only a yield of 76%. Catalyst recovery was feasible, and 70% of the catalyst was re-isolated in the mentioned 0.5 mmol scale reaction.

In general, the free acids elute relatively slowly from column, and some product was likely lost because it was too diluted for detection in late fractions or it remained on column. The issue was particularly relevant on small scale, and the lower yields for cases in which the acids were isolated are partially due to their high polarity. For example, product 1l was obtained in only 64% as the acid, but the yield was 95% for the methyl ester 2l under otherwise identical reaction conditions.

**Scheme 4:**
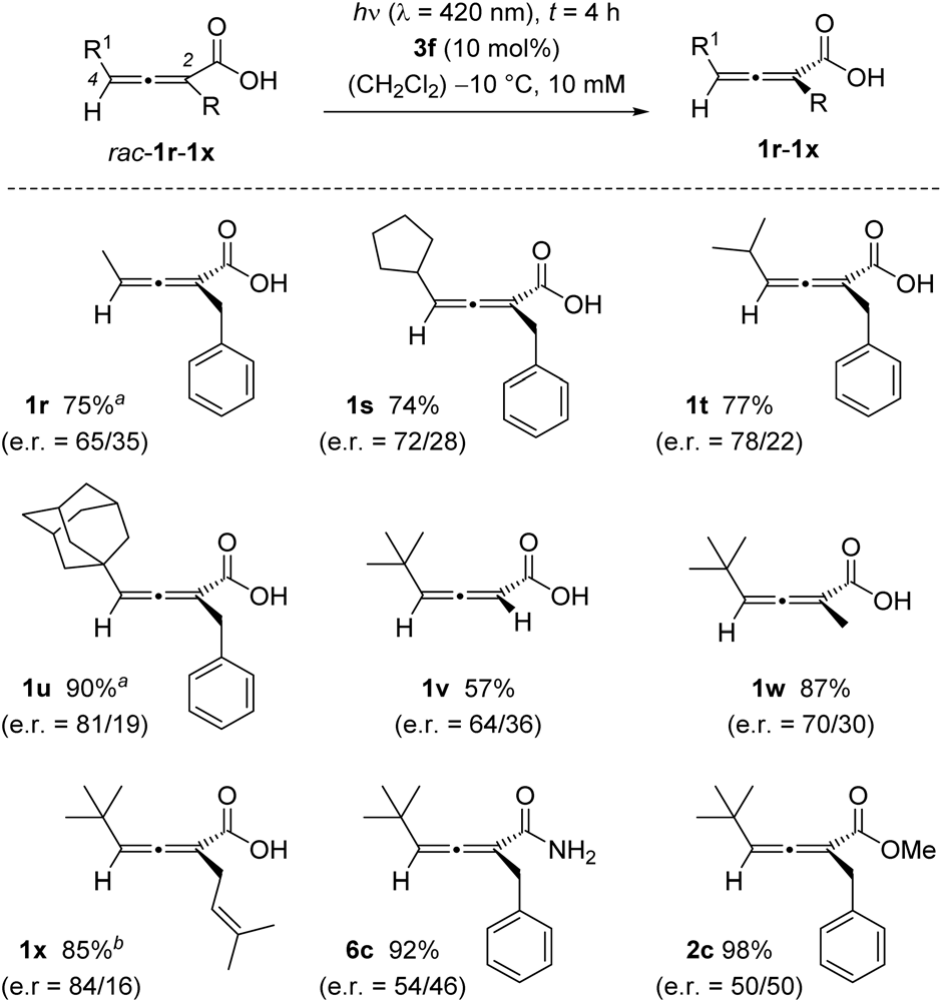
Influence of substitution and binding motif on the outcome of the photochemical deracemization of 2,3-allenoic acids *rac*-1r to *rac*-1x and of allenoic acid derivatives *rac*-6c and *rac*-2c. ^*a*^ Yield and e.r. of the product were obtained after methyl ester formation as described in Scheme 2. ^*b*^ Yield of the isolated carboxylic acid, the e.r. was determined after primary amide formation.

Expectedly, the size of the substituents in 2- and 4-position plays a significant role in the enantiomer discrimination by the catalyst. If the size of the substituent R^1^ at C4 was decreased, the e.r. of the product acid was lower than for standard substrate 1c (R^1^ = *tert*-butyl). The methyl-substituted acid 1r (R^1^ = methyl) was obtained in a comparably low e.r. of 65/35. When increasing the steric bulk of R^1^, the e.r. improved as seen for 1s, 1t, and 1u. Likewise, small groups R at position C2 led to relatively low enantioselectivity (Scheme 4).

For R = H (1v) and R = methyl (1w), the e.r. was lower than for the standard substrate 1c with R = benzyl. A dimethylallyl group at C2, however, seems to mimic the benzyl group well, and acid 1x was successfully deracemized in good yield (85%, e.r. = 84/16). Phenyl groups in position C2 or C4 of the allene lead to a bathochromic shift of the UV/Vis absorption which likely invites a direct excitation and hampers the enantioselectivity in an attempted deracemization reaction (see the SI for details). Although an association of the carboxylic acid to the catalyst by hydrogen bonding was assumed to be an integral part of the mode of action, we confirmed the critical influence of the binding site by subjecting other allenoic acid derivatives to the deracemization conditions. Both amide *rac*-6c and ester *rac*-2c resulted in low or no enantioselectivity.

**Scheme 5:**
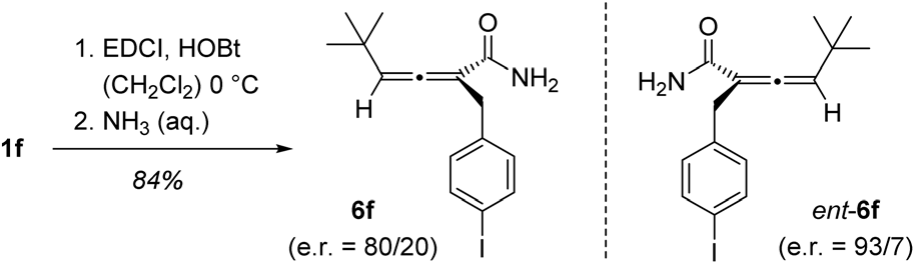
Proof of absolute configuration: Enantioenriched carboxylic acid was converted into amide 6f (EDCI = 1-ethyl-3-(3-dimethylaminopropyl)carbodiimide, HOBt = 1-hydroxybenzotriazole) which was compared on chiral HPLC with a sample of amide *ent*-6f,^12*c*^ the absolute configuration of which was known.

The absolute configuration of the products was established by comparison with authentic material of known configuration. Enantioenriched acid 1f was converted to amide 6f which was shown by chiral HPLC analysis to exist as an 80/20 mixture of enantiomers 6f/*ent*-6f. The absolute configuration of minor enantiomer *ent*-6f was known from previous work.^12*c*^ On chiral HPLC, the enantiomers are baseline separated which allowed to assign the absolute configuration of the stereogenic axis as (*R*). The same exercise was performed for acid 1h confirming the assignment. All other configuration assignments were based on analogy with the major enantiomer being (*R*)-configured (Scheme 5).

**Scheme 6:**
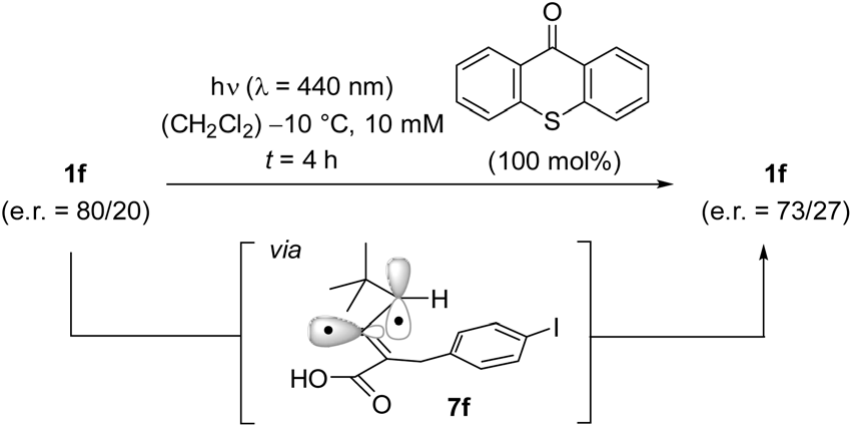
2,3-Allenoic acid 1f is not configurationally stable upon excitation. In the presence of thioxanthen-9-one, irradiation at *λ* = 440 nm leads to a decrease in the enantiomeric ratio, presumably *via* achiral triplet intermediate 5.

Mechanistically, the configurational instability of allenes 1 seems to be the result of a sensitized excitation leading to an achiral triplet intermediate.^23^ Experiments with enantioenriched (scalemic) substrate 1f (e.r. = 80/20) performed at *λ* = 440 nm and at *T* = −10 °C showed the compound to be configurationally stable. If achiral thioxanthen-9-one was added and the reaction was performed under otherwise unchanged conditions (Scheme 6) a slight but clearly detectable racemization was observed. The most likely scenario to account for the racemization is a sensitization by thioxanthen-9-one, the triplet energy [E(T_1_)] of which has been determined as E(T_1_) = 274 kJ mol^−1^ (77 K, methylcyclohexane-isopentane).^24^ The intermediate allene-derived diradical 7f^25^ is achiral and reforms statistically either one of the two product enantiomers 1f and *ent*-1f. The only small e.r. change is attributed to the low absorption coefficient *ε* of thioxanthen-9-one at *λ* = 440 nm (*ε* ≤ 1 L mol^−1^ cm^−1^ in CH_2_Cl_2_).

**Fig. 2:**
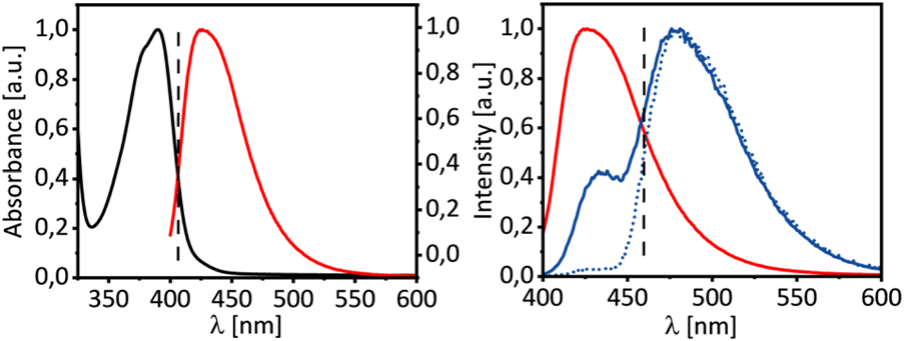
Photophysical properties of catalyst 3f: Normalized absorption and emission spectra at ambient temperature in CH_2_Cl_2_ solution (left). Emission spectra recorded at r.t. (red solid line) and 77 K (blue solid line) in CH_2_Cl_2_ (*λ*_exc_ = 385 nm). The blue dotted line shows the phosphorescence spectrum at 77 K (*λ*_exc_ = 385 nm, *t*_delay_ = 100 μs) in CH_2_Cl_2_.

At shorter wavelength, employing fluorescent lamps emitting at *λ* = 420 nm, the racemization was more extensive (e.r. = 66/34) under otherwise identical conditions. However, it was surprisingly found, that substrate 1f undergoes a detectable racemization (e.r. = 76/24) even in the absence of the catalyst. We ascribe this observation to a minor emission band of the fluorescent lamps occurring at *λ* = 365 nm (for the spectral data see the SI). In the deracemization experiments, this emission should be inconsequential, since catalyst 3f has a far more extensive absorption at this wavelength than substrate *rac*-1f and it should be almost exclusively excited. The allenoic acid absorbs minimally at *λ* = 365 nm with *ε* ≤ 1 L mol^−1^ cm^−1^ (CH_2_Cl_2_) while the absorption coefficient for 3f is *ε* = 5630 L mol^−1^ cm^−1^ (CH_2_Cl_2_). In fact, the photophysical properties of catalyst 3f (Fig. 2) are similar to parent thioxanthen-9-one.^26^

The energy of the lowest lying singlet state was determined from the crossing point of the absorption and fluorescence emission as E(S_1_) = 295 kJ mol^−1^. The compound shows an intense luminescence at 77 K which is mostly due to phosphorescence. The triplet energy was calculated from the point of inflection in the phosphorescence spectrum as E(T_1_) = 260 ± 2 kJ mol^−1^ (77 K, CH_2_Cl_2_).

**Scheme 7:**
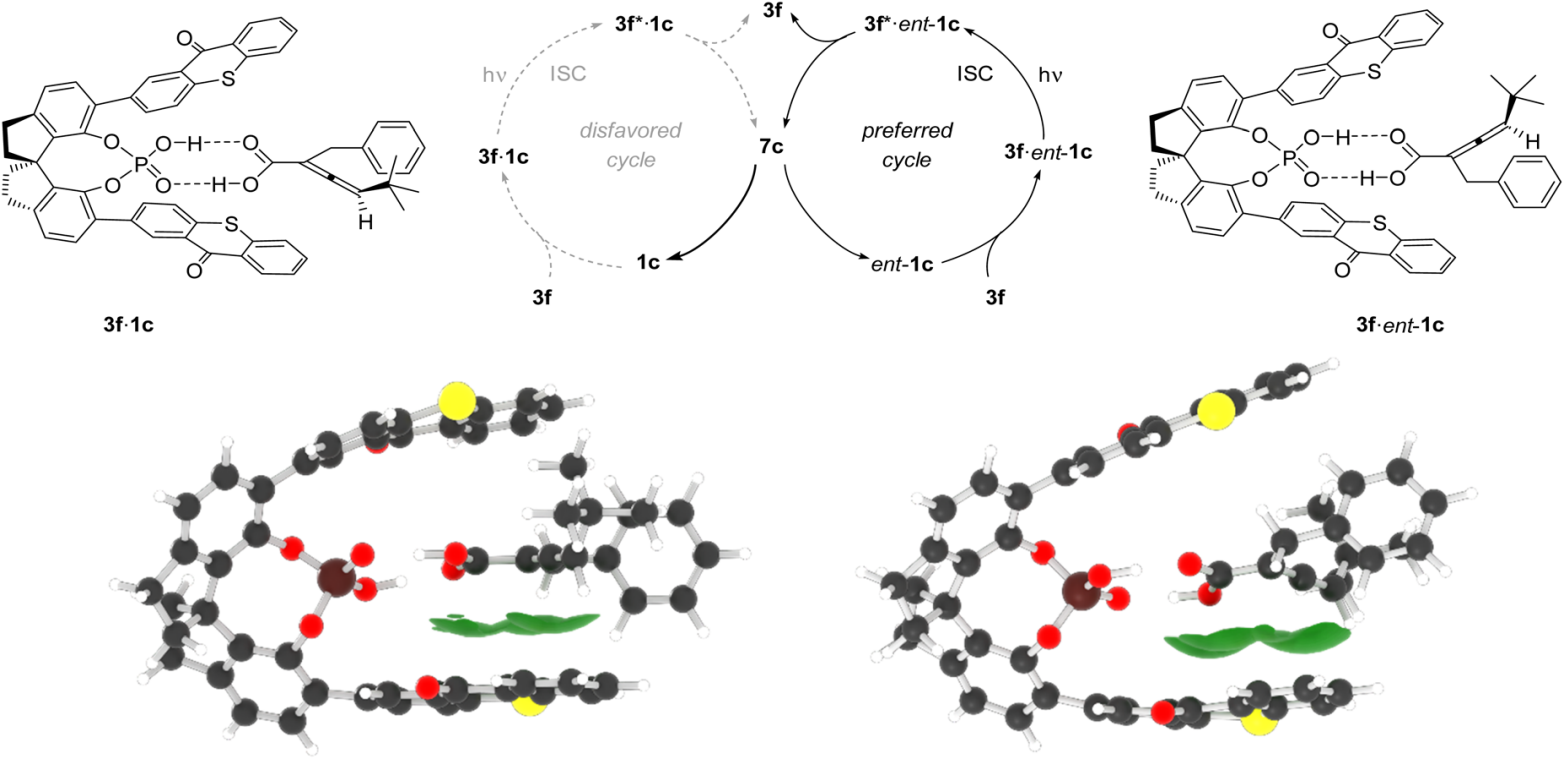
Mechanistic scenario for the deracemization of racemic allenoic acid *rac*-1c upon irradiation in the presence of catalyst 3f. Triplet energy transfer is faster in complex 3f·*ent*-1c than in complex 3f·1c because the average distance of the thioxanthone chromophore to the allene is smaller. Triplet energy transfer from excited 3f* to the allene leads to formation of achiral triplet intermediate 5c (*cf.*Scheme 6) which dissociates from the phosphoric acid and forms either one of the two enantiomers 1c or *ent*-1c of the allenoic acid. The lowest minimum energy conformations of each enantiomer with catalyst 3f are shown below, in addition to the overlap of scaled van der Waals radii (green areas) to visualize the proximity of the allenoic acids to the catalyst.

The most coherent explanation for the observed enantiomer differentiation is based on a preferred energy transfer to one substrate enantiomer of allenoic acid. The situation is depicted for the parent compound *rac*-1c in Scheme 7 and was further corroborated by quantum-chemical calculations. Dexter energy transfer^27^ is strongly distance dependent, and proximity of the triplet energy donor and the respective acceptor facilitate energy transfer.^28^ We propose that enantiomer *ent*-1c is more readily processed by photoexcited catalyst 3f* because the thioxanthone and the allene chromophore are spatially closer in complex 3f·*ent*-1c than in the diastereomeric complex 3f·1c. If this is correct the catalytic cycle involving the former complex is preferred and the rate of racemization *via* achiral intermediate 7c is higher for allenoic acid enantiomer *ent*-1c. In the photostationary state, the enantiomer 1c, which is more slowly processed, prevails and is isolated in excess.

For theoretical investigations, minimum energy conformers were computed using conformational sampling^29–31^ of complexes 3f·*ent*-1c and 3f·1c with the GFN2-xTB^32,33^ method, followed by subsequent geometry optimization and frequency calculation to confirm the correct minimum energy structures. Therefore, the PBEh-3c^34–37^ composite method including implicit solvation^38,39^ was used. In addition, thermodynamic corrections^33,40,41^ were included to the electronic energies that were computed using the PW6B95-D3(BJ)/def2-QZVP^35,36,42,43^ level of theory. The most stable minimum energy conformers of both complexes are visualized in Scheme 7. The Gibbs free enthalpy of both diastereomeric complexes is almost identical with a calculated Δ*G* of only 0.473 kJ mol^−1^. Subsequent analyses were based on all sampled ground-state minimum energy conformers (40 for 3f·*ent*-1c and 24 for 3f·1c) that were weighted by a Boltzmann distribution. Computational details on the sampling procedure, energy calculations, and analyses can be found in the SI.

As Dexter-type triplet energy transfers are strongly distance dependent, we analysed the interatomic distances of the two distinct diastereomeric complexes as previous investigations of similar allene deracemizations have shown that distances observed in the ground-state equilibrium geometries are similar to those in the triplet manifold.^12*c*^ Analysis revealed that the distance between the thioxanthone moiety and the conjugated atoms in the allene is smaller in case of complex 3f·*ent*-1c compared to complex 3f·1c, both for the lowest minimum energy conformer as well as in the Boltzmann-weighted average. To avoid dependencies on arbitrary reference points and the resulting introduction of a bias, we used the overlap of scaled van der Waals radii^44^ as a measure for the proximity of the relevant groups. A larger volume indicates a better overlap of the radii and a greater proximity of the two components within the assembly. The resulting volumes obtained for the minimum structures, 2.98 Å^3^ for 3f·1c and 3.81 Å^3^ for 3f·*ent*-1c, are plotted in addition to the structures in Scheme 7 (green areas). The Boltzmann-weighted averages, 2.97 Å^3^ for 3f·1c and 3.72 Å^3^ for 3f·*ent*-1c, confirm the observation obtained from the lowest minimum energy conformers. The differences in the volumina are clearly visible and are additionally supported by the distances of each atom in the allene moiety to the thioxanthone plane that are plotted in the SI. These results confirm previously observed trends that shorter distances are in correlation with lower barriers in Dexter-type triplet-energy transfers.^28^

Since the distance difference for the diastereomeric complexes 3f·1*vs.*3f·*ent*-1 is relatively low, the achieved enantioselectivity is not as high as in previous cases.^12^ While the enantioselectivities obtained with spinol-derived catalyst 3f are moderate, our computational analysis shows that the selectivity correlates with differences in Dexter energy transfer efficiency between diastereomeric complexes, quantified by Boltzmann-weighted proximity/overlap descriptors. This mechanistic handle suggests avenues for optimization, such as closer or asymmetric placement of the thioxanthone unit, tuning of the chromophore's triplet energy, or reinforcement of the hydrogen-bonding network. These strategies, together with data-driven screening based on the computed descriptors^45^ or machine learning optimization techniques,^46^ provide a roadmap for future improvements in enantioselectivity.

An interesting twist of the Dexter energy transfer mechanism relates to the fact that a dual catalytic system is conceivable, in which achiral thioxanthen-9-one and a chiral phosphoric acid with a suitable chromophore act synergistically. Preliminary results showed the approach to be viable but the enantioselectivities remained lower than with the sensitizing phosphoric acid 3f (see the SI for details).

**Scheme 8:**
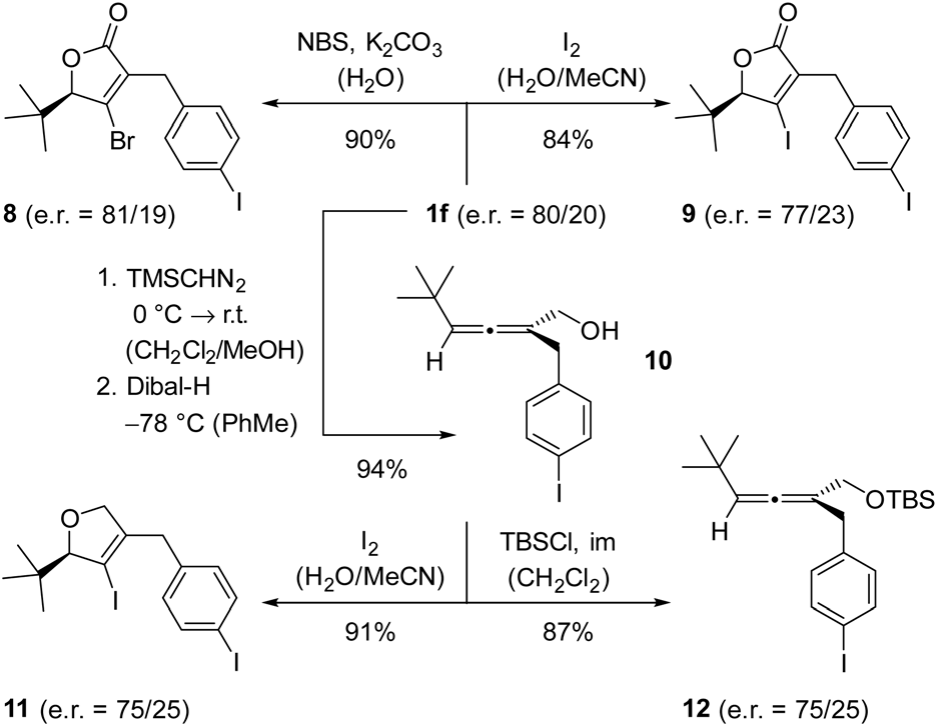
Consecutive reactions of scalemic allenoic acid 1f. Abbreviations: Dibal-H = diisobutylaluminium hydride; im = imidazole; NBS = *N*-bromosuccinimide; TBS = *tert*-butyldimethylsilyl.

The transformation of axially chiral allenes to products with a stereogenic center has been studied extensively in prior work. In particular, the Diels–Alder reaction has been broadly utilized and was shown to occur with high chirality transfer.^47^ In the present study, we have mainly investigated a consecutive halolactonization^48^ or haloesterification (Scheme 8).^49^ We found the bromolactonization of scalemic allenoic acid 1f to occur with perfect chirality transfer generating lactone 8 in high yield. The absolute configuration of the major enantiomer was assigned based on the assumption that the temporarily formed bromonium ion is substituted intramolecularly by the carboxylic acid.^48,50^ The iodolactonization to product 9 proceeded smoothly but the enantiomeric purity was not fully retained. Upon reduction of the acid to alcohol 10, the iodoetherification delivered the desired ether 11 in an e.r. of 75/25. Since the e.r. of alcohol 10 could not be determined by chiral HPLC, it was transformed into silyl ether 12. Based on the fact that its e.r. was identical to the e.r. of the iodoetherification product, it seems most likely that the observed loss of enantiomeric purity is due to the reduction step while the absolute configuration is retained in the two consecutive reactions.

## Conclusions

In summary, we have successfully shown that a chiral phosphoric acid can be employed as a single photocatalyst for a photochemical deracemization reaction. The catalyst operates likely by triplet energy transfer to the allenoic acid, inducing a racemization *via* the triplet state of the substrate. Proper positioning of the thioxanthone chromophore in the catalyst is crucial for the differentiation of the two substrate enantiomers. The relatively high flexibility of catalyst and allenoic acid precludes the catalyst from recruiting a single allene enantiomer in the energy transfer step. Although there is a preference for processing one enantiomer over the other, the degree of differentiation and consequently the enantioselectivity are yet not as optimal as previously seen for azabicyclo[3.1.1]nonan-2-one-based energy transfer catalysts. Still, the results provide an excellent starting point for further catalyst optimization and design.

## Author contributions

M. S. and T. B. developed the project. Funding was acquired by T. B. and J. W.; M. S. designed and performed the synthetic experiments. M. S. generated and validated the experimental data, D. B. the computational data. T. B. and J. W. administered the project and supervised the research. All authors wrote, reviewed, and edited the manuscript.

## Conflicts of interest

There are no conflicts to declare.

## Supplementary Material

SC-OLF-D5SC05356K-s001

## Data Availability

The data that supports the findings of this study are available in the supplementary material (SI) of this article. Primary research data are openly available in the repository RADAR4Chem at https://doi.org/10.22000/14d69n7httd5f1yw.^51^ Supplementary information: synthetic procedures and full characterization for all starting materials and products (1c–1x, 2c, 2e, 2g, 2i, 2j, 2k, 2r, 2u, 3e–3g, 6c, 6f, 6x, 8–12), spectroscopic and computational data. See DOI: https://doi.org/10.1039/d5sc05356k.
