# Supplementary material for: Photochemical deracemization of 2,3-allenoic acids mediated by a sensitizing chiral phosphoric acid catalyst
Source: Chem Sci. 2025 Sep 22;16(42):19711–9. doi: 10.1039/d5sc05356k (PMC12477638; doi:10.1039/d5sc05356k)
Supplement: SC-016-D5SC05356K-s001 [file SC-016-D5SC05356K-s001.pdf]

Supplementary Information (SI) for Chemical Science.
This journal is © The Royal Society of Chemistry 2025

Electronic Supplementary Information for:

Photochemical deracemization of 2,3-allenoic acids mediated by a sensitizing chiral phosphoric acid catalyst

Max Stierle,[a] Daniel Bitterlich,[b] Julia Westermayr,[b,c] and Thorsten Bach*[a]

[a]Department of Chemistry and Catalysis Research Center (CRC), School of Natural Sciences
Technische Universität München
D-85747 Garching, Germany

[b]Wilhelm-Ostwald-Institute for Physical and Theoretical Chemistry
Leipzig University
D-04103 Leipzig, Germany

[c]Center for Scalable Data Analytics and Artificial Intelligence (ScaDS.AI)
D-04105 Leipzig, Germany

Table of Contents

1. General Information	3
2. Analytical Methods	5
3. Computational Studies	7
4. Synthesis of Phosphoric Acid Catalysts **3e-3g**	10
5. Substrate Synthesis and Photochemical Deracemization of *rac*-**1**	16
6. Determination of the Absolute Configuration	55
7. Synthesis and Deracemization of Phenyl-Substituted Allene Carboxylic Acids	57
8. Racemization Experiments and Additional UV/VIS Spectra	60
9. Deracemization Reactions Employing a Dual Catalyst System	64
10. Consecutive Reactions	65
11. Emission Spectra and Triplet Energy Measurements	71
12. Chiral HPLC/GLC Traces	72
13. NMR Spectra	104
14. Data Sheets of Light Sources	152
15. References	154

1. General Information

All reactions sensitive to air or moisture were carried out in flame-dried glassware under positive argon pressure using standard *Schlenk* techniques.

Commercially available chemicals were used without further purification unless stated differently.

Dichloromethane (CH_2Cl_2) was dried using a MBSP 800 MBraun purification system with the following columns:

Dichloromethane: 2 × MB-KOL-A type 2 (aluminium oxide)

The following dry solvents are commercially available and were used without further purification: Dioxane (*Acros Organics*, 99.5% extra dry, over molecular sieves), pyridine (*Acros Organics*, 99.5% extra dry, over molecular sieves), methanol (*Acros Organics*, 99.8% extra dry, over molecular sieves), 1,2-dichloroethane (*Acros Organics*, 99.5% extra dry, over molecular sieves), toluene (*Acros Organics*, 99.85% extra dry, over molecular sieves), acetone *Acros Organics*, 99.8% extra dry). 1,2-dimethoxyethane (DME, >99%) and α,α,α,α',α',α'-hexafluoro-*m*-xylene (HFX, 98%) were purchased from *TCI Europe*. α,α,α-Trifluorotoluene (Tft) was bought from *Sigma Aldrich* (*Merck*). Ethanol (EtOH) was used after simple distillation of the technical solvent.

Deuterated solvents were purchased from *Deutero*.

Technical solvents were distilled prior to use for column chromatography [hexane (Hex), pentane (p), dichloromethane, methanol (MeOH), ethyl acetate (EtOAc), diethyl ether (Et_2O)]. Flash column chromatography was performed on silica 60 (*Merck*, 230-400 mesh) with the indicated solvent mixtures (v/v). Solvents used in photochemical reactions (CH_2Cl_2, DCE, acetone) were degassed by four freeze-pump-thaw cycles and stored over 4 Å molecular sieves, or the respective reaction solutions were degassed under a continuous argon flow under ultrasonication for fifteen minutes (PhMe, HFX, Tft).

Photochemical reactions (λ = 420 nm) were performed in flame dried *Duran* tubes (diameter = 1 cm) in a positive geometry setup (cylindrical array of 16 fluorescent light tubes Luzchem LZC-420, 8 W nominal power) with the sample placed in the center of the illumination chamber.Reactions at low temperatures were performed using a *Duran* cooling finger attached to a cryostat (*Huber* CC505 or *Huber* CC 80).[1]

For reactions at 0 °C ice/water mixtures were used as coolant.

The catalysts **3a-3d**,[2] catalyst precursors **4a**[3] and **4b**,[4] as well as the borylated thioxanthone derivatives **5a**[5] and **5b**[6] were prepared according to literature procedures or under slight derivations of these. The ethyl allenoates leading to allene carboxylic acids *rac*-**1c**-**1f**, *rac*-**1h**-**1i**, *rac*-**1l**, *rac*-**1o**-**1p**, *rac*-**1w** and *rac*-**SI-1** were synthesized according to a literature procedure.[7]

2. Analytical Methods

Melting points (M.p.) were determined using a *Kofler* heating bar and are uncorrected.

Thin layer chromatography (TLC) was performed on silica coated glass plates (*Merck*, silica 60 F254) using detection by UV-light (λ = 254 nm) and/or by staining with a potassium permanganate solution [$KMnO_4$] followed by heating.

$KMnO_4$-staining solution: potassium permanganate (3.00 g), potassium carbonate (20.0 g), and 5% aqueous sodium hydroxide solution (5.00 mL) in water (300 mL).

Infrared spectra (IR) were recorded on a Perkin Elmer Frontier IR-FTR spectrometer by ATR technique. The signal intensity is stated using the following abbreviations: s (strong), m (medium), w (weak).

Nuclear Magnetic Resonance Spectra (NMR) were recorded at room temperature either on a Bruker AVHD-400, AVHD-500, or an AV-III-500 (equipped with a QNP cryo sample head). ^{1}H NMR spectra were calibrated to the residual proton signal of chloroform-d_1 (δ = 7.26 ppm) or dimethylsulfoxide-d_6 (δ = 2.50 ppm). ^{13}C NMR spectra were referenced to the 13C triplet of chloroform-d_1 (δ = 77.16 ppm) or to the ^{13}C septet of dimethylsulfoxide-d_6 (δ = 39.5 ppm). ^{31}P NMR spectra were measured without reference. The following abbreviations were used to indicate the multiplicities of a signal: br – broad, s – singlet, d – doublet, t – triplet, p – pentet, m – multiplet. Apparent multiplets resulting from equal coupling constants of magnetically non-equivalent protons are marked as virtual (*virt.*). Assignments of ^{1}H and ^{13}C NMR signals were based on two-dimensional NMR experiments (COSY, HSQC, HMBC, NOESY).

High Resolution Mass Spectra (HRMS) were measured on a Q Exactive Plus from *Thermo Fisher Scientific* (HRMS-ESI) with an orbitrap mass analyzer or on a *Thermo Scientific* DFS-HRMS spectrometer (EI, 70 eV).

UV-Vis Spectroscopy was performed on a *Perkin Elmer* Lambda 365+ UV-Vis spectrometer using a *Hellma* precision cell (quartz SUPRASIL®) with a pathlength of 1 mm.

Chiral Gas Liquid Chromatography (GLC) was performed on an *Agilent* 7890 B gas chromatograph using a Cyclosil-B column (30 m, 0.25 mm, 0.25 μm, SN: USF620714H) with a flame ionization detector.

High Performance Liquid Chromatography (HPLC) was performed using a *Thermo Fisher* Ultimate 3000 device equipped with one of the following chiral stationary phases [ChiralPak AD-H (250 × 4.6 mm), ChiralPak IC (250 × 4.6 mm), ChiralPak AS-H (250 × 4.6 mm),

ChiralPak IA (250 × 4.6 mm), Chiralcel OJ-RH (150 × 4.6 mm) or Chiralcel OD-RH (150 × 4.6 mm), *Daicel Chemical Industries*] with LPG 3400SD Pump, WPS3000SL Autosampler and a DAD 3000 photodiode array (detection at λ = 215 nm or λ = 210).

Specific Rotation was determined using a *Bellingham+Stanley* ADP440+ polarimeter and is reported as follows: $[\alpha]_D^T$ (c in g per 100 mL solvent).

Luminescence Measurements were performed on a Horiba Scientific FluoroMax-4P instrument equipped with a continuous Xe source for steady state measurements and a Xe flashlight source for the observation of phosphorescence spectra. All measurements were performed in quartz tubes (inner diameter = 4 mm) under nitrogen-atmosphere. If necessary, the samples were cooled in a small quartz Dewar vessel with liquid nitrogen (77 K).

3. Computational Studies

Sampling and Energy Calculations

To obtain statistically significant results, sampling of different minimum energy conformers of the catalyst-substrate assemblies was conducted using crest[8] at the GFN2-xTB[9] level of theory with implicit solvation. Implicit solvation was accounted for with the ALPB model[10] using a dielectric constant of 8.93 corresponding to dichloromethane. The iMTD-GC sampling workflow[11] with default parameters was used. For each conformer, the corresponding tautomer was created and all of the resulting conformers were subsequently optimized using the composite method PBEh-3c.[12] This method employs the D3 dispersion correction with the Becke-Johnson-Damping[13,14] model to include missing dispersion correction. It also accounts for the basis set superposition error.[15] For geometry optimizations and subsequent frequency calculations, solvent effects were included using the conductor-like polarizable continuum model (CPCM)[16] for implicit solvation. The Gibbs free enthalpies were calculated using the qRRHO approach.[15] Final electronic energies of the conformers were calculated at the PW6B95-D3(BJ)/def2-QZVP level of theory.[14,17] Geometry optimizations, frequency calculations and single point calculations were performed using the ORCA software package version 6.0.[18] Calculations in ORCA employed the RIJ approximation with the def2/J[19] auxillary basis set. Corrections for the solvation free enthalpy δG_{solv} were obtained using the GFN2-xTB level of theory[10,20] and the ALPB[10] model with the "bar1M" option.[10] To ensure that the found minimum energy conformers were true minimum energy structures, frequency calculations were performed, ensuring no negative frequencies were found. As some conformers converged to the same minimum, duplicates were removed, resulting in final ensembles of 24 conformers for **3f**·**1c** and 40 conformers for **3f**·*ent*-**1c**.

Boltzmann Weighting

Analysis was conducted for the lowest minimum energy conformers and a Boltzmann distribution of all sampled minimum structures. Boltzmann weights, w_i, at the reaction temperature of 263.15 K were obtained for conformer *i* as

$$w_i = \frac{e^{-\frac{\Delta G_i}{k_B T}}}{\sum_j e^{-\frac{\Delta G_j}{k_B T}}}.$$

The relative free energies were obtained as the difference of the energies of the individual conformer and the minimum energy conformer of the ensemble. The free enthalpies for the

conformers, G_i, were obtained by combining the final electronic energies E_{el} from PW6B95-D3(BJ)/def2-QZVP calculations, thermochemical corrections, $G_{\text{T,R,V}}$, due to finite temperature effects obtained from the frequency calculations, and the free solvation enthalpies, δG_{solv}, as:

$$G_i = E_{\text{el},i} + G_{\text{T,R,V},i} + \delta G_{\text{solv},i}$$

Methods used for each of the computational steps are additionally summarized in Table S3.1.

Table S3.1. *Computational details for each calculation step.*

Conformer search	iMTD-GC sampling workflow[5] as implemented in crest[1,2] @ GFN2-xTB[3] + implicit solvation in DCM with the ALPB model.[4]
Geometry optimization and frequency calculations	PBEh-3c[6–9] with implicit solvation in DCM with the CPCM model[10,11]
Electronic energies E_{el}	PW6B95-D3(BJ)/def2-QZVP[8,12,13,16]
Finite temperature corrections to the free enthalpy including zero point vibrational energy $G_{\text{T,R,V}}$	qRRHO-model[9] based on the PBEh-3c frequencies
Solvation free enthalpy correction δG_{solv}	GFN2-xTB/ALPB(DCM) with the xtb program[4,17] as contained in Orca 6.0, including the standard state correction going from 1 bar of ideal gas and 1 mol/L liquid solution (keyword “bar1M”)

Analysis and Van der Waals Radii Computation

As previously mentioned, analysis was conducted for the lowest minimum energy conformer and the Boltzmann-weighted ensemble for each diastereoisomer. The overlaps of the van der Waals spheres, which were obtained from van der Waals radii[21] using a scaling factor of 1.2, are computed using numerical integration. The code for the computation of van der Waals radii and subsequent analysis is additionally provided as supplementary information. To further support the analysis, we computed the distances from the thioxanthone plane to each atom in the allene moiety, which is visualized in **Figure S3.1**. As can be seen, the difference in the distances between the allene atoms and the thioxanthone plane of the two diastereomers is either negligible or smaller for the **3f**·*ent*-**1c** complex compared to the **3f**·**1c** complex.

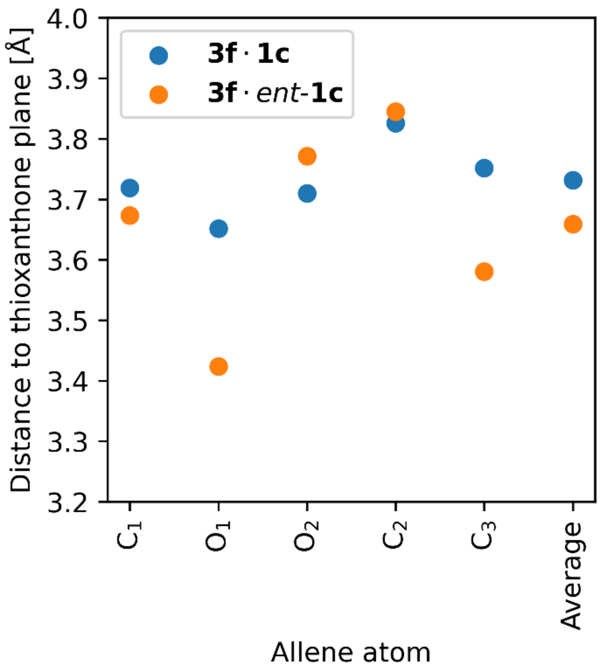

Figure S3.1. Distances of the allene atoms (numbered according to Scheme 1 in the main text) to the plane spanned by the thioxanthone molecule for the two different diastereoisomers.

4. Synthesis of Phosphoric Acid Catalysts 3e-3g

(*R*)-2-(12-Hydroxy-12-oxido-4,5,6,7-tetrahydrodiindeno[7,1-*de*:1',7'-*fg*][1,3,2]dioxaphosphocin-1-yl)-9*H*-thioxanthen-9-one (3e)

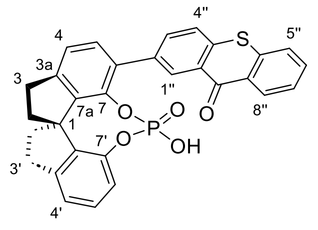

$C_{30}H_{21}O_5PS$
524.08 g/mol

Suzuki Cross-coupling:

In analogy to a literature procedure:[4] A product mixture containing **4a**[3] (556 mg, 62 wt%, 739 µmol, 1.00 eq.), and borylated thioxanthone **5a** (435 mg, 1.29 mmol, 1.75 eq.) were dissolved in DME (8.6 mL), cataCXium® A (15.4 mg, 42.9 µmol, 6 mol%) and $Pd(OAc)_2$ (7.70 mg, 34.4 µmol, 5 mol%) were added. Subsequently, 1 M K_2CO_{3aq} (8.6 mL, 8.58 mmol, 11.6 eq.) was added and the reaction mixture was heated at 80 °C for 15 hours. After the indicated reaction time CH_2Cl_2 was added and the phases were separated. The aqueous layer was extracted with CH_2Cl_2 (3 × 15 mL). The combined organic extracts were washed with saturated NH_4Cl_{aq} (1 × 20 mL) and water (1 × 20 mL) and were dried over Na_2SO_4. After filtration and removal of the solvent under reduced pressure, purification by column chromatography (silica, P/Et_2O = 90/10 → 85/15) yielded the corresponding cross-coupled product (358 mg, 650 µmol, 88%) as a yellow solid.

MOM-Deprotection:

According to a modified literature procedure:[22] A fraction of the cross-coupled product (150 mg, 272 µmol, 1.00 eq.) was dissolved in 1,4-dioxane (5.5 mL) and conc. HCl_{aq} (544 µL, 6.54 mmol, 24.0 eq.) was added. The reaction mixture was stirred at room temperature for 4 hours, after which water was added (5 mL). The phases were separated, and the aqueous phase was extracted with CH_2Cl_2 (3 × 10 mL). The combined organic extracts were dried over Na_2SO_4, filtered and the solvent was removed under reduced pressure. The obtained crude diol was used without further purification in the next step.

Phosphorylation:

A fraction of the crude diol (82.9 mg, 179 µmol, 1.00 eq.) was dissolved in pyridine (6 mL), and $POCl_3$ (54.9 mg, 33.5 µL, 358 µmol, 2.00 eq.) was added dropwise at room temperature.

The obtained reaction solution was stirred at 60 °C for 17 hours. Subsequently, water (6 mL) was added at room temperature and the reaction mixture was heated at 100 °C for 3 hours. The reaction mixture was cooled to room temperature and 6 N HCl_{aq} (12.2 mL) was added, followed by heating to 100 °C for 1 hour. The reaction mixture was extracted with CH_2Cl_2 (3 × 20 mL), and the combined organic extracts were dried over Na_2SO_4, filtered and the solvent was removed under reduced pressure. Purification by column chromatography (silica, CH_2Cl_2/MeOH = 95/5) yielded a yellow solid which was redissolved in CH_2Cl_2 (15 mL) and washed with 6 N HCl_{aq} (2 × 15 mL). Drying of the organic layer over Na_2SO_4, filtration and removal of all solvents under reduced pressure yielded phosphoric acid **3e** (55.8 mg, 106 µmol, 59%) as a yellow solid.

TLC: R_f = 0.48 (CH_2Cl_2/MeOH = 95/5) [UV].

M.p.: >230 °C

^{1}H NMR (500 MHz, DMSO-d_6, 300 K): δ [ppm] = 8.56 – 8.51 (m, 1H, C1”-H), 8.49 (dd, ^3J = 8.2 Hz, ^4J = 1.5 Hz, 1H, C8”-H), 7.91 – 7.83 (m, 3H, C_{Ar}-H), 7.79 (ddd, ^3J = 8.3 Hz, ^3J = 7.0 Hz, ^4J = 1.5 Hz, 1H, C6”-H), 7.60 (ddd, ^3J = 8.2 Hz, ^3J = 7.0 Hz, ^4J = 1.2 Hz, 1H, C7”-H), 7.39 (d, ^3J = 7.7 Hz, 1H, C_{Ar}-H), 7.28 (d, ^3J = 7.7 Hz, 1H, C_{Ar}-H), 7.21 (*virt.* t, $^3J \approx {}^3J$ = 7.6 Hz, 1H, C5’-H), 7.16 (d, ^3J = 7.6 Hz, 1H, C4’-H), 6.88 (d, ^3J = 7.6 Hz, 1H, C6’-H), 3.22 – 2.98 (m, 2H, C_{Alk}-H), 2.94 – 2.71 (m, 2H, C_{Alk}-H), 2.38 – 2.19 (m, 2H, C_{Alk}-H), 2.05 – 1.95 (m, 1H, C_{Alk}-H), 1.95 – 1.80 (m, 1H, C_{Alk}-H).

^{13}C NMR (126 MHz, DMSO-d_6, 300 K): δ [ppm] = 178.8 (s, C=O), 146.2 (s, C_{Ar}), 146.2 (s, C_{Ar}), 146.0 (s, C_{Ar}), 146.0 (s, C_{Ar}), 143.0 (d, $^2J_{C\text{-}P}$ = 8.3 Hz, C_{Ar}), 140.8 (d, $^3J_{C\text{-}P}$ = 3.2 Hz, C_{Ar}), 139.6 (d, $^3J_{C\text{-}P}$ = 3.2 Hz, C_{Ar}), 136.8 (s, C_{Ar}), 136.5 (s, C_{Ar}), 135.0 (s, C_{Ar}), 134.2 (d, C_{Ar}), 133.1 (d, C_{Ar}), 132.9 (d, $^3J_{C\text{-}P}$ = 3.2 Hz, C_{Ar}), 129.8 (d, C_{Ar}), 129.5 (d, C1”), 129.2 (d, C8”), 128.4 (d, C5’), 128.3 (s, C_{Ar}), 126.9 (d, C7”), 126.7 (d, C_{Ar}), 126.4 (d, C_{Ar}), 122.4 (d, C_{Ar}), 122.1 (d, C4’), 121.4 (d, $^3J_{C\text{-}P}$ = 2.3 Hz, C6’), 58.9 (s, C1), 38.6 (t, C_{Alk}), 38.2 (t, C_{Alk}), 30.0 (t, C_{Alk}), 29.9 (t, C_{Alk}).

^{31}P NMR (203 MHz, DMSO-d_6, 300 K): δ [ppm] = −12.14.

IR (ATR): ṽ (cm^{-1}) = 2950 (w), 1636 (m), 1590 (m), 1436 (m), 1136 (m), 1001 (s), 892 (s).

HRMS (ESI): calc. for [M + H^+]: 525.0921; found: 525.0917.

Specific Rotation: $[\alpha]_D^{25}$ = 240 (c = 1.0, $CHCl_3$).

(*R*)-2,2'-(12-Hydroxy-12-oxido-4,5,6,7-tetrahydrodiindeno[7,1-*de*:1',7'-*fg*][1,3,2]dioxaphosphocine-1,10-diyl)bis(9*H*-thioxanthen-9-one) (3f)

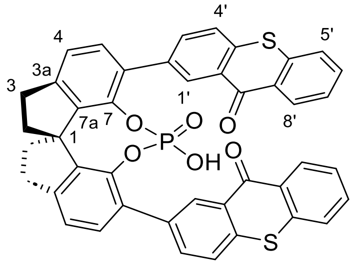

$C_{43}H_{27}O_6PS_2$
734.78 g/mol

Suzuki Cross-coupling:

In analogy to a literature procedure:[4] Compound **4b** (400 mg, 675 µmol, 1.00 eq.) and borylated thioxanthone **5a** (571 mg, 1.69 mmol, 2.50 eq.) were dissolved in DME (6.8 mL), cataCXium® A (12.1 mg, 33.8 µmol, 5 mol%) and $Pd(OAc)_2$ (6.1 mg, 27.0 µmol, 4 mol%) were added. Subsequently, 1 M K_2CO_{3aq} (6.75 mL, 6.75 mmol, 10.0 eq.) was added and the reaction mixture was heated at 80 °C for 22 hours. After the indicated reaction time CH_2Cl_2 was added and the phases were separated. The aqueous layer was extracted with CH_2Cl_2 (3 × 15 mL). The combined organic extracts were washed with saturated NH_4Cl_{aq} (1 × 30 mL) and water (1 × 30 mL) and were dried over Na_2SO_4. After filtration and removal of the solvent under reduced pressure, purification by column chromatography (silica, P/Et_2O = 90/10 → P/Et_2O = 80/20 + 20% CH_2Cl_2) yielded the corresponding cross-coupled product (454 mg, 596 µmol, 88%) as a yellow solid.

MOM-Deprotection:

According to a modified literature procedure:[22] A fraction of the cross-coupled product (435 mg, 572 µmol, 1.00 eq.) was dissolved in 1,4-dioxane (11.2 mL) and conc. HCl_{aq} (1.14 mL, 13.7 mmol, 24.0 eq.) was added. The reaction mixture was heated at 60 °C for 2.5 hours, after which water was added (10 mL). The phases were separated, and the aqueous layer was extracted with CH_2Cl_2 (3 × 15 mL). The combined organic extracts were dried over Na_2SO_4, filtered and the solvent was removed under reduced pressure. The obtained crude diol was used without further purification in the next step.

Phosphorylation:

The crude diol (385 mg, 572 µmol, 1.00 eq.) was dissolved in pyridine (13.5 mL) and $POCl_3$ (175 mg, 107 µL, 1.14 mmol, 2.00 eq.) was added dropwise at room temperature. The obtained reaction solution was stirred at 60 °C for 19 hours. Subsequently, water (13.5 mL) was added

at room temperature and the reaction mixture was heated at 100 °C for 3 hours. The reaction mixture was cooled to room temperature and 6 N HCl_{aq} (26 mL) was added, followed by heating to 100 °C for 1 hour. The reaction mixture was extracted with CH_2Cl_2 (3 × 30 mL), and the combined organic extracts were dried over Na_2SO_4, filtered and the solvent was removed under reduced pressure. Purification by column chromatography (silica, CH_2Cl_2/MeOH = 98/2 → 90/10) yielded a yellow solid which was redissolved in CH_2Cl_2 (20 mL) and washed with 6 N HCl_{aq} (2 × 15 mL). Drying of the organic layer over Na2SO4, filtration and removal of all solvents under reduced pressure yielded phosphoric acid **3f** (233 mg, 317 µmol, 55% over two steps) as a yellow solid.

TLC: R_f = 0.19 (CH_2Cl_2/MeOH = 95/5) [UV].

M.p.: >230 °C

^{1}H NMR (500 MHz, DMSO-d_6, 300 K): δ [ppm] = 8.49 (d, ^4J = 1.8 Hz, 2H, C1'-H), 8.43 (dd, ^3J = 8.2 Hz, ^4J = 1.5 Hz, 2H, C8'-H), 7.85 – 7.77 (m, 6H, C3'-H, C4'-H, C5'-H), 7.74 (ddd, ^3J = 8.3 Hz, ^3J = 7.0 Hz, ^4J = 1.5 Hz, 2H, C6'-H), 7.55 (ddd, ^3J = 8.2 Hz, ^3J = 7.0 Hz, ^4J = 1.5 Hz, 2H, C7'-H), 7.39 (d, ^3J = 7.7 Hz, 2H, C5-H), 7.29 (d, ^3J = 7.7 Hz, 2H, C4-H), 3.30 – 3.07 (m, 2H, C3-H^a), 3.02 – 2.85 (m, 2H, 2H, C3-H^b), 2.43 – 2.31 (m, 2H, C2-H^a), 2.12 – 1.98 (m, 2H, C2-H^b).

^{13}C NMR (126 MHz, DMSO-d_6, 300 K): δ [ppm] = 178.8 (s, C=O), 146.1 (s, C3a), 143.0 (d, $^2J_{C\text{-}P}$ = 8.5 Hz, C7), 141.0 (d, $^3J_{C\text{-}P}$ = 3.1 Hz, C7a), 136.7 (s, C2'), 136.4 (s, C4'a), 134.9 (s, C_{Ar}), 134.1 (d, C_{Ar}), 133.0 (d, C_{Ar}), 132.7 (d, $^3J_{C\text{-}P}$ = 3.2 Hz, C6), 129.6 (d, C5), 129.5 (d, C1'), 129.1 (d, C8'), 128.3 (s, C_{Ar}), 128.1 (s, C_{Ar}), 126.9 (d, C7'), 126.7 (d, C_{Ar}), 126.2 (d, C_{Ar}), 122.4 (d, C4), 59.4 (s, C1), 38.6 (t, C2), 29.9 (t, C3).

^{31}P NMR (162 MHz, DMSO-d_6, 300 K): δ [ppm] = −13.3.

IR (ATR): $\tilde{\nu}$ (cm^{-1}) = 2961 (w), 1634 (m), 1591 (m), 1437 (m), 1260 (s), 1009 (s), 747 (s).

HRMS (ESI): calc. for [M + H^+]: 735.1060; found: 735.1051.

Specific Rotation: $[\alpha]_D^{25}$ = 302 (c = 1.0, $CHCl_3$).

(*R*)-3,3'-(12-Hydroxy-12-oxido-4,5,6,7-tetrahydrodiindeno[7,1-*de*:1',7'-*fg*][1,3,2]dioxapho sphocine-1,10-diyl)bis(9*H*-thioxanthen-9-one) (3g)

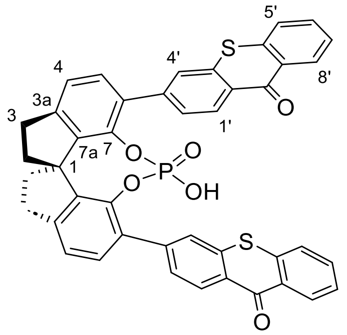

$C_{43}H_{27}O_6PS_2$
734.78 g/mol

Suzuki Cross-coupling:

In analogy to a literature procedure:[4] Compound **4b** (300 mg, 507 µmol, 1.00 eq.) and borylated thioxanthone **5b** (428 mg, 1.27 mmol, 2.50 eq.) were dissolved in DME (5.1 mL), cataCXium® A (9.08 mg, 25.3 µmol, 5 mol%) and $Pd(OAc)_2$ (4.55 mg, 20.3 µmol, 4 mol%) were added. Subsequently, 1 M K_2CO_{3aq} (5.07 mL, 5.07 mmol, 10.0 eq.) was added and the reaction mixture was heated at 80 °C for 17 hours. After the indicated reaction time CH_2Cl_2 was added and the phases were separated. The aqueous layer was extracted with CH_2Cl_2 (3 × 10 mL). The combined organic extracts were washed with saturated NH_4Cl_{aq} (1 × 20 mL) and water (1 × 20 mL) and were dried over Na_2SO_4. After filtration and removal of the solvent under reduced pressure, purification by column chromatography (silica, P → P/Et_2O = 75/25) yielded the corresponding cross-coupled product (382 mg, 502 µmol, 99%) as a yellow solid.

MOM-Deprotection:

According to a modified literature procedure:[22] The cross-coupled product (382 mg, 502 µmol, 1.00 eq.) was dissolved in 1,4-dioxane (10.1 mL) and conc. HCl_{aq} (1.00 mL, 12.1 mmol, 24.0 eq.) was added. The reaction mixture was heated at 60 °C for 18 hours, after which water was added (10 mL). The phases were separated, and the aqueous layer was extracted with CH_2Cl_2 (3 × 15 mL). The combined organic extracts were dried over Na_2SO_4, filtered and the solvent was removed under reduced pressure. The obtained crude diol was used without further purification in the next step.

Phosphorylation:

The crude diol was dissolved in pyridine (11.8 mL) and $POCl_3$ (154 mg, 93.9 µL, 1.00 mmol, 2.00 eq.) was added dropwise at room temperature. The obtained reaction solution was stirred

at 60 °C for 17 hours. Subsequently, water (11.8 mL) was added at room temperature and the reaction mixture was heated at 100 °C for 3 hours. The reaction mixture was cooled to room temperature and 6 N HCl_{aq} (23 mL) was added, followed by heating to 100 °C for 1 hour. The reaction mixture was extracted with CH_2Cl_2 (3 × 30 mL), and the combined organic extracts were dried over Na_2SO_4, filtered and the solvent was removed under reduced pressure. Purification by column chromatography (silica, CH_2Cl_2 → CH_2Cl_2/MeOH 96/4) yielded a yellow solid which was redissolved in CH_2Cl_2 (20 mL) and washed with 6 N HCl_{aq} (2 × 15 mL). Drying of the organic layer over Na_2SO_4, filtration and removal of all solvents under reduced pressure yielded phosphoric acid **3g** (268 mg, 365 µmol, 73% over two steps) as a yellow solid.

TLC: R_f = 0.43 (CH_2Cl_2/MeOH = 90/10) [UV].

M.p.: >230 °C

^{1}H NMR (500 MHz, DMSO-d_6, 300 K): δ [ppm] = 8.43 (dd, ^3J = 8.2 Hz, ^4J = 1.5 Hz, 2H, C8'-H), 8.40 (d, ^3J = 8.4 Hz, 2H, C1'-H), 7.89 (d, ^4J = 1.7 Hz, 2H, C4'-H), 7.81 (dd, ^3J = 8.3 Hz, ^4J = 1.4 Hz, 2H, C5'-H), 7.74 (ddd, ^3J = 8.3 Hz, ^3J = 7.0 Hz, ^4J = 1.5 Hz, 2H, C6'-H), 7.65 (dd, ^3J = 8.4 Hz, ^4J = 1.7 Hz, 2H, C2'-H), 7.55 (ddd, ^3J = 8.2 Hz, ^3J = 7.0 Hz, ^4J = 1.4 Hz, 2H, C7'-H), 7.44 (d, ^3J = 7.7 Hz, 2H, C5-H), 7.28 (d, ^3J = 7.7 Hz, 2H, C4-H), 3.24 – 3.06 (m, 2H, C3-H^a), 2.97 – 2.79 (m, 2H, C3-H^b), 2.41 – 2.29 (m, 2H, C2-H^a), 2.13 – 2.02 (m, 2H, C2-H^b).

^{13}C NMR (126 MHz, DMSO-d_6, 300 K): δ [ppm] = 178.6 (s, C=O), 146.7 (s, C3a), 143.3 (d, $^2J_{C\text{-}P}$ = 8.2 Hz, C7), 142.8 (s, C_{Ar}), 141.1 (d, $^3J_{C\text{-}P}$ = 3.3 Hz, C7a), 136.7 (s, C_{Ar}), 136.3 (s, C_{Ar}), 133.0 (d, C6'), 132.1 (d, $^3J_{C\text{-}P}$ = 3.2 Hz, C6), 129.8 (d, C5), 129.1 (d, C8'), 128.7 (d, C1'), 128.4 (s, C_{Ar}), 128.3 (d, C2'), 126.8 (d, C_{Ar}), 126.7 (d, C_{Ar}), 126.6 (d, C_{Ar}), 122.2 (d, C4), 59.3 (s, C1), 38.6 (t, C2), 29.9 (t, C3).[§]

[§]One of the aromatic carbon atoms was not detected by ^{13}C NMR, presumably due to signal overlap.

^{31}P NMR (203 MHz, DMSO-d_6, 300 K): δ [ppm] = −13.1.

IR (ATR): ṽ (cm^{-1}) = 2927 (w), 1635 (m), 1598 (s), 1437 (m), 1005 (m), 747 (s).

HRMS (ESI): calc. for [M + H^+]: 735.1060; found: 735.1043.

Specific Rotation: $[\alpha]_D^{25}$ = 486 (c = 1.0, $CHCl_3$).

5. Substrate Synthesis and Photochemical Deracemization of *rac*-1

*General Procedure 1 (GP 1): Synthesis of Ethyl Allenoates leading to rac-**1***[7]

(Carbethoxymethylene)triphenylphosphorane (1.00 eq.) was dissolved in chloroform (0.43 M) and the respective benzyl bromide (1.27 eq.) was added portion wise. After heating the obtained solution at 70 °C overnight, the solvent was removed under reduced pressure to obtain the respective phosphonium salt as an off-white foam. The thereby obtained salt was redissolved in dichloromethane (0.35 M) and NEt_3 (2.20 eq.) was added dropwise. The solution was stirred for 30 minutes and the respective acid chloride (1.00 eq.) was added dropwise over 30 minutes. After further stirring overnight, the reaction mixture was filtered over *Celite*® and after repeated flushing with dichloromethane the solvent was removed under reduced pressure. The crude product was purified by flash column chromatography to obtain the ethyl allenoates leading to *rac*-**1**. Most ethyl allenoates are literature known and a detailed procedure is only provided for compounds not previously described.[7]

*General Procedure 2 (GP 2): Synthesis of Allene Carboxylic Acids rac-**1***[7]

The corresponding ethyl allenoate was dissolved in water / ethanol (1/1 % v/v, 0.5 M) and freshly grinded sodium hydroxide (1.10 eq.) was added. The obtained reaction mixture was heated at 100 °C for 3 h. The reaction mixture was cooled to room temperature and 4 N HCl_{aq} was added until an acidic pH (pH = 1-2) was reached. The reaction mixture was extracted (3 × EtOAc), the combined organic phases were dried over Na_2SO_4, filtered and the solvent was removed under reduced pressure. The obtained crude acids were purified by flash column chromatography to obtain allene carboxylic acids *rac*-**1**.

*General Procedure 3 (GP 3): Synthesis of Methyl Allenoates from Allene Carboxylic Acids rac-**1***

The respective allene carboxylic acid *rac*-**1** (1.00 eq.) was dissolved in dichloromethane / methanol (5/1 % v/v, 83 mM) and at 0 °C a solution of trimethylsilyldiazomethane (2.1 M in hexane, 2.00 eq.) was added dropwise. The obtained yellow solution was stirred for 1.5 h at room temperature and acetic acid (~50 µL) was added to destroy excess trimethylsilyldiazomethane. The obtained colorless solution was stirred for further two minutes and all solvents were removed under reduced pressure. The obtained crude product was purified by flash column chromatography (silica, P/Et_2O = 90/10) to obtain the respective methyl allenoates.

*General Procedure 4 (GP 4): Photochemical Deracemization of Allene Carboxylic Acids rac-**1***

A solution of the respective allene carboxylic acid *rac*-**1** (1.00 eq.) and chiral phosphoric acid catalyst **3f** (10 mol%) in dichloromethane (c = 10 mM) was cooled to −10 °C and irradiated (λ = 420 nm) for 4 h. The solvent was removed under reduced pressure and the obtained crude product was purified by flash column chromatography (silica, Et_2O) to obtain the enantioenriched allene carboxylic acids **1**.

2-Benzyl-5,5-dimethylhexa-2,3-dienoic acid (*rac*-1c)

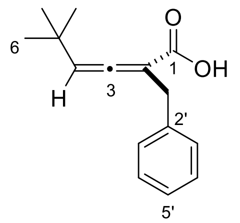

$C_{15}H_{18}O_2$
230.31 g/mol

According to **GP2** the respective ethyl allenoate[7] (2.35 g, 9.10 mmol, 1.00 eq.) was dissolved in EtOH (9 mL) and water (9 mL). NaOH (400 mg, 10.0 mmol, 1.10 eq.) was added, and the reaction mixture was heated at 100 °C for three hours. After cooling to room temperature 4 N HCl_{aq} was added until an acidic pH (pH = 1-2) was reached. The reaction mixture was extracted with EtOAc (3 × 20 mL), the combined organic phases were dried over Na_2SO_4, filtered and the solvent was removed under reduced pressure. The obtained crude acid was purified by flash column chromatography (silica, P/Et_2O = 90/10) to obtain allene carboxylic acid *rac*-**1c** (986 mg, 4.29 mmol, 47%) as a colorless solid.

TLC: R_f = 0.44 (Hex/EtOAc = 70/30) [UV/$KMnO_4$].

^{1}H NMR (400 MHz, $CDCl_3$, 300 K): δ [ppm] = 7.31 – 7.25 (m, 2H, C4'-H), 7.23 – 7.16 (m, 3H, C3'-H, C5'-H), 5.49 (*virt.* t, $^5J \approx ^5J$ = 2.7 Hz, 1H, C4-H), 3.58 (dd, ^2J = 15.2 Hz, ^5J = 2.7 Hz, 1H, C1'-H^a), 3.52 (dd, ^2J = 15.2 Hz, ^5J = 2.7 Hz, 1H, C1'-H^b), 0.96 (s, 9H, $C[CH_3]_3$).

^{13}C NMR (101 MHz, $CDCl_3$, 300 K): δ [ppm] = 209.9 (s, C3), 172.8 (s, C1), 139.2 (s, C2'), 129.3 (d, C3'), 128.4 (d, C4'), 126.4 (d, C5'), 107.6 (d, C4), 102.0 (s, C2), 35.1 (t, C1'), 33.2 (s, *C*[CH_3]$_3$), 29.9 (q, C[*C*H_3]$_3$).

Analytical data matched those previously reported in the literature.[7]

Methyl (*R*)-2-benzyl-5,5-dimethylhexa-2,3-dienoate (2c)

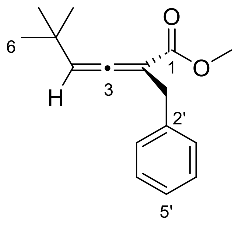

$C_{16}H_{20}O_2$
244.33 g/mol

*Synthesis of rac-**2c**:*

According to **GP3** allene carboxylic acid *rac*-**1c** (46.1 mg, 200 µmol, 1.00 eq.) was reacted with trimethylsilyldiazomethane (2.1 M in hexane, 190 µL, 400 µmol, 2.00 eq.) to afford the methyl ester *rac*-**2c** (48.4 mg, 198 µmol, 99%) as a colorless oil.

Photochemical Deracemization of Allene Carboxylic Acid:

According to **GP4** allene carboxylic acid *rac*-**1c** (11.5 mg, 50.0 µmol, 1.00 eq.) was reacted with phosphoric acid catalyst **3f** (3.67 mg, 5.00 µmol, 10 mol%). After removing the solvent under reduced pressure, the crude product was subjected to the esterification conditions described in **GP3**. After column chromatography, enantioenriched methyl ester **2c** (11.8 mg, 48.4 µmol, 97%, e.r. = 82/18) was isolated as a colorless oil.

*Attempted Deracemization of rac-**2c**:*

According to **GP4** allene carboxylic acid methyl ester *rac*-**2c** (12.2 mg, 50.0 µmol, 1.00 eq.) was reacted with phosphoric acid catalyst **3f** (3.67 mg, 5.00 µmol, 10 mol%). After removing the solvent under reduced pressure, the crude product was subjected to column chromatography and methyl ester *rac*-**2c** was recovered (11.9 mg, 48.8 µmol, 98%, e.r. = 50/50) as a colorless oil.

TLC: R_f = 0.71 (Hex/EtOAc = 80/20) [UV/$KMnO_4$].

^{1}H NMR (400 MHz, $CDCl_3$, 300 K): δ [ppm] = 7.30 – 7.24 (m, 2H, C4'-H), 7.23 – 7.16 (m, 3H, C3'-H, C5'-H), 5.42 (*virt.* t, $^5J \approx {}^5J$ = 2.7 Hz, 1H, C4-H), 3.73 (s, 3H, CO_2CH_3), 3.59 (dd, ^2J = 15.2 Hz, ^5J = 2.7 Hz, 1H, C1'-H^a), 3.53 (dd, ^2J = 15.2 Hz, ^5J = 2.7 Hz, 1H, C1'-H^b), 0.95 (s, 9H, C[CH_3]$_3$).

^{13}C NMR (101 MHz, $CDCl_3$, 300 K): δ [ppm] = 208.7 (s, C3), 168.0 (s, *C*O_2CH_3), 139.4 (s, C2'), 129.3 (d, C3'), 128.3 (d, C4'), 126.4 (C5'), 107.3 (d, C4), 102.2 (s, C2), 52.3 (q, CO_2*C*H_3), 35.6 (t, C1'), 33.0 (s, *C*[CH_3]$_3$), 30.0 (q, C[*C*H_3]$_3$).

IR (ATR): $\tilde{\nu}$ (cm^{-1}) = 2958 (w), 1956 (w), 1712 (s), 1251 (m), 1266 (m), 822 (w), 700 (s).

HRMS (ESI): calc. for [M + H^+]: 245.1537; found: 245.1533.

Chiral HPLC: t_{R1} = 18.8 min t_{R2} = 19.3 min (*Daicel* Chiralcel OJ-RH, 150×4.6 mm, MeCN/H_2O = 20/80 → 100/0, 1 mL/min, λ = 215 nm).

(*R*)-2-(4-Fluorobenzyl)-5,5-dimethylhexa-2,3-dienoic acid (1d)

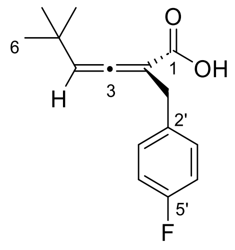

$C_{15}H_{17}FO_2$
248.30 g/mol

*Synthesis of rac-**1d**:*

According to **GP2** the respective ethyl allenoate[7] (2.01 g, 7.27 mmol, 1.00 eq.) was dissolved in EtOH (7 mL) and water (7 mL). NaOH (320 mg, 8.00 mmol, 1.10 eq.) was added, and the reaction mixture was heated at 100 °C for three hours. After cooling to room temperature 4 N HCl_{aq} was added until an acidic pH (pH = 1-2) was reached. The reaction mixture was extracted with EtOAc (3 × 15 mL), the combined organic phases were dried over Na_2SO_4, filtered and the solvent was removed under reduced pressure. The obtained crude acid was purified by flash column chromatography (silica, P/Et_2O = 90/10 → 85/15) to obtain allene carboxylic acid *rac*-**1d** (719 mg, 2.89 mmol, 40%) as a colorless solid.

Photochemical Deracemization of Allene Carboxylic Acid:

According to **GP4** allene carboxylic acid *rac*-**1d** (12.4 mg, 50.0 µmol, 1.00 eq.) was reacted with phosphoric acid catalyst **3f** (3.67 mg, 5.00 µmol, 10 mol%). After removing the solvent under reduced pressure, the crude product was purified by flash column chromatography to obtain the enantioenriched allene carboxylic acid **1d** (9.3 mg, 37.5 µmol, 75%, e.r. = 77/23) as a colorless solid.

TLC: R_f = 0.48 (Hex/EtOAc = 70/30) [UV/$KMnO_4$].

^{1}H NMR (400 MHz, $CDCl_3$, 300 K): δ [ppm] = 7.23 – 7.12 (m, 2H, C3'-H), 7.04 – 6.79 (m, 2H, C4'-H), 5.51 (*virt.* t, $^5J \approx {}^5J$ = 2.7 Hz, 1H, C4-H), 3.54 (dd, ^2J = 15.3 Hz, ^5J = 2.7 Hz, 1H, C1'-H^a), 3.48 (dd, ^2J = 15.3 Hz, ^5J = 2.7 Hz, 1H, C1'-H^b), 0.97 (s, 9H, C[CH_3]$_3$).

^{13}C NMR (101 MHz, $CDCl_3$, 300 K): δ [ppm] = 209.8 (s, C3), 172.7 (s, C1), 161.7 (d, $^1J_{CF}$ = 244 Hz, C5'), 134.9 (d, $^4J_{CF}$ = 3.4 Hz, C2'), 130.7 ($^3J_{CF}$ = 7.8 Hz, C3'), 115.1 (d, $^2J_{CF}$ = 21.2 Hz, C4'), 107.8 (d, C4), 101.9 (s, C2), 34.3 (t, C1'), 33.2 (s, *C*[CH_3]$_3$), 30.0 (q, C[*C*H_3]$_3$).

^{19}F NMR (376 MHz, $CDCl_3$, 300 K): δ [ppm] = -117.0 – -117.1 (m).

Chiral HPLC: t_{R1} = 6.1 min t_{R2} = 6.7 min (*Daicel* Chiralpak AD-H, 250×4.6 mm, n-Hep/iso-PrOH = 90/10, 1 mL/min, λ = 210 nm).

NMR data matched those previously reported in the literature.[7]

2-(4-Bromobenzyl)-5,5-dimethylhexa-2,3-dienoic acid (*rac*-1e)

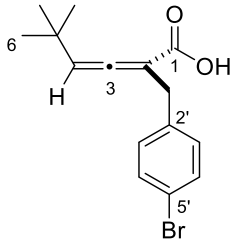

$C_{15}H_{17}BrO_2$
309.20 g/mol

According to **GP2** the respective ethyl allenoate[7] (613 mg, 1.82 mmol, 1.00 eq.) was dissolved in EtOH (2 mL) and water (2 mL). NaOH (80.0 mg, 2.00 mmol, 1.10 eq.) was added, and the reaction mixture was heated at 100 °C for three hours. After cooling to room temperature 4 N HCl_{aq} was added until an acidic pH (pH = 1-2) was reached. The reaction mixture was extracted with EtOAc (3 × 10 mL), the combined organic phases were dried over Na_2SO_4, filtered and the solvent was removed under reduced pressure. The obtained crude acid was purified by flash column chromatography (silica, P/Et_2O = 90/10 → 80/20) to obtain allene carboxylic acid *rac*-**1e** (301 mg, 975 µmol, 54%) as a colorless solid.

TLC: R_f = 0.46 (Hex/EtOAc = 70/30) [UV/$KMnO_4$].

^{1}H NMR (400 MHz, $CDCl_3$, 300 K): δ [ppm] = 7.42 – 7.35 (m, 2H, C4'-H), 7.13 – 7.06 (m, 2H, C3'-H), 5.52 (*virt.* t, $^5J \approx {}^5J$ = 2.6 Hz, 1H, C4-H), 3.52 (dd, ^2J = 15.2 Hz, ^5J = 2.6 Hz, 1H, C1'-H^a), 3.46 (dd, ^2J = 15.2 Hz, ^5J = 2.6 Hz, 1H, C1'-H^b), 0.99 (s, 9H, C[CH_3]$_3$).

^{13}C NMR (101 MHz, $CDCl_3$, 300 K): δ [ppm] = 209.8 (s, C3), 172.4 (s, C1), 138.2 (s, C5'), 131.5 (d, C4'), 131.0 (d, C3'), 120.3 (s, C2'), 107.8 (d, C4), 101.4 (s, C2), 34.6 (t, C1'), 33.3 (s, *C*[CH_3]$_3$), 30.0 (q, C[*C*H_3]$_3$).

Analytical data matched those previously reported in the literature.[7]

Methyl (*R*)-2-(4-bromobenzyl)-5,5-dimethylhexa-2,3-dienoate (2e)

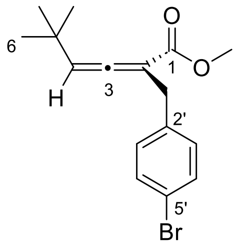

$C_{16}H_{19}BrO_2$
323.23 g/mol

*Synthesis of rac-**2e**:*

According to **GP3** allene carboxylic acid *rac*-**1e** (30.9 mg, 100 µmol, 1.00 eq.) was reacted with trimethylsilyldiazomethane (2.1 M in hexane, 95.2 µL, 200 µmol, 2.00 eq.) to afford the methyl ester *rac*-**2e** (32.2 mg, 99.6 µmol, *quant.*) as a colorless oil.

Photochemical Deracemization of Allene Carboxylic Acid:

According to **GP4** allene carboxylic acid *rac*-**1e** (15.5 mg, 50.0 µmol, 1.00 eq.) was reacted with phosphoric acid catalyst **3f** (3.67 mg, 5.00 µmol, 10 mol%). After removing the solvent under reduced pressure, the crude product was subjected to the esterification conditions described in **GP3**. After column chromatography, enantioenriched methyl ester **2e** (15.1 mg, 46.6 µmol, 93%, e.r. = 78/22) was isolated as a colorless oil.

TLC: R_f = 0.65 (Hex/EtOAc = 80/20) [UV/$KMnO_4$].

M.p.: 33 °C.

^{1}H NMR (500 MHz, $CDCl_3$, 300 K): δ [ppm] = 7.43 – 7.35 (m, 2H, C4'-H), 7.14 – 7.00 (m, 2H, C3'-H), 5.45 (*virt.* t, $^5J \approx {}^5J$ = 2.7 Hz, 1H, C4-H), 3.72 (s, 3H, CO_2CH_3), 3.53 (dd, ^2J = 15.1 Hz, ^5J = 2.7 Hz, 1H, C1'-H^a), 3.46 (dd, ^2J = 15.1 Hz, ^5J = 2.7 Hz, 1H, C1'-H^b), 0.97 (s, 9H, C[CH_3]$_3$).

^{13}C NMR (101 MHz, $CDCl_3$, 300 K): δ [ppm] = 208.6 (s, C3), 167.8 (s, C1), 138.5 (s, C2'), 131.4 (d, C4'), 131.0 (d, C3'), 120.2 (s, C5'), 107.5 (d, C4), 101.7 (s, C2), 52.3 (q, CO_2*C*H_3), 35.1 (t, C1'), 33.1 (s, *C*[CH_3]$_3$), 30.0 (q, C[*C*H_3]$_3$).

IR (ATR): $\tilde{\nu}$ (cm^{-1}) = 2960 (w), 1954 (w), 1704 (s), 1487 (m), 1266 (s), 840 (s), 734 (s).

HRMS (ESI): calc. for [M + H^+]: 323.0642; found: 323.0636.

Chiral HPLC: t_{R1} = 21.4 min t_{R2} = 22.0 min (*Daicel* Chiralcel OJ-RH, 150×4.6 mm, MeCN/H_2O = 20/80 → 100/0, 1 mL/min, λ = 215 nm, 5 °C).

(*R*)-2-(4-Iodobenzyl)-5,5-dimethylhexa-2,3-dienoic acid (1f)

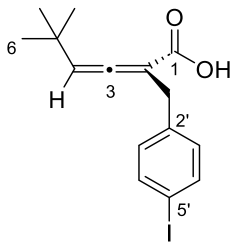

$C_{15}H_{17}IO_2$
356.20 g/mol

*Synthesis of rac-**1f**:*

According to **GP2** the respective ethyl allenoate[7] (1.51 g, 3.93 mmol, 1.00 eq.) was dissolved in EtOH (4 mL) and water (4 mL). NaOH (173 mg, 4.32 mmol, 1.10 eq.) was added, and the reaction mixture was heated at 100 °C for three hours. After cooling to room temperature 4 N HCl_{aq} was added until an acidic pH (pH = 1-2) was reached. The reaction mixture was extracted with EtOAc (3 × 15 mL), the combined organic phases were dried over Na_2SO_4, filtered and the solvent was removed under reduced pressure. The obtained crude acid was purified by flash column chromatography (silica, P/Et_2O = 95/5 → 85/15) to obtain allene carboxylic acid *rac*-**1f** (904 mg, 2.54 mmol, 65%) as a colorless solid.

Photochemical Deracemization of Allene Carboxylic Acid:

According to **GP4** allene carboxylic acid *rac*-**1f** (17.8 mg, 50.0 µmol, 1.00 eq.) was reacted with phosphoric acid catalyst **3f** (3.67 mg, 5.00 µmol, 10 mol%). After removing the solvent under reduced pressure, the crude product was purified by flash column chromatography to obtain the enantioenriched allene carboxylic acid **1f** (13.6 mg, 38.2 µmol, 76%, e.r. = 81/19) as a colorless solid.

TLC: R_f = 0.49 (Hex/EtOAc = 70/30) [UV/$KMnO_4$].

^{1}**H NMR** (400 MHz, $CDCl_3$, 300 K): δ [ppm] = 7.64 – 7.54 (m, 2H, C4'-H), 7.04 – 6.91 (m, 2H, C3'-H), 5.52 (*virt.* t, $^5J \approx {}^5J$ = 2.6 Hz, 1H, C4-H), 3.51 (dd, ^2J = 15.2 Hz, ^5J = 2.6 Hz, 1H, C1'-H^a), 3.45 (dd, ^2J = 15.2 Hz, ^5J = 2.6 Hz, 1H, C1'-H^b), 0.99 (s, 9H, C[CH_3]$_3$).

^{13}**C NMR** (101 MHz, $CDCl_3$, 300 K): δ [ppm] = 209.8 (s, C3), 172.2 (s, C1), 138.9 (s, C2'), 137.5 (d, C4'), 131.3 (d, C3'), 107.9 (d, C4), 101.3 (s, C2), 91.7 (C5'), 34.7 (t, C1'), 33.3 (s, *C*[CH_3]$_3$), 30.0 (q, C[*C*H_3]$_3$).

Chiral HPLC: t_{R1} = 17.0 min t_{R2} = 17.7 min (*Daicel* Chiralcel OJ-RH, 150×4.6 mm, MeCN/H_2O = 20/80 → 100/0, 1 mL/min, λ = 215 nm).

NMR data matched those previously reported in the literature.[7]

2-(4-(Tert-butyl)benzyl)-5,5-dimethylhexa-2,3-dienoic acid (*rac*-1g)

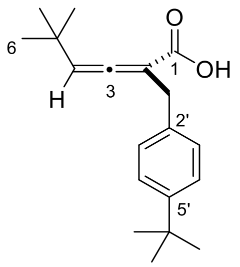

$C_{19}H_{26}O_2$
286.42 g/mol

According to **GP2** the respective ethyl allenoate[7] (1.32 g, 4.20 mmol, 1.00 eq.) was dissolved in EtOH (4 mL) and water (4 mL). NaOH (185 mg, 4.62 mmol, 1.10 eq.) was added, and the reaction mixture was heated at 100 °C for three hours. After cooling to room temperature 4 N HCl_{aq} was added until an acidic pH (pH = 1-2) was reached. The reaction mixture was extracted with EtOAc (3 × 15 mL), the combined organic phases were dried over Na_2SO_4, filtered and the solvent was removed under reduced pressure. The obtained crude acid was purified by flash column chromatography (silica, P/Et_2O = 90/10 → 80/20) to obtain allene carboxylic acid *rac*-**1g** (606 mg, 2.12 mmol, 51%) as a colorless solid.

TLC: R_f = 0.23 (Hex/EtOAc = 70/30) [UV/$KMnO_4$].

^{1}**H NMR** (400 MHz, $CDCl_3$, 300 K): δ [ppm] = 7.34 – 7.28 (m, 2H, C4'-H), 7.18 – 7.06 (m, 2H, C3'-H), 5.47 (*virt.* t, $^5J \approx {}^5J$ = 2.7 Hz, 1H, C4-H), 3.55 (dd, ^2J = 15.3 Hz, ^5J = 2.7 Hz, 1H, C1'-H^a), 3.50 (dd, ^2J = 15.3 Hz, ^5J = 2.7 Hz, 1H, C1'-H^b), 1.30 (s, 9H, C6'[CH_3]$_3$), 0.94 (s, 9H, C5[CH_3]$_3$).

^{13}**C NMR** (101 MHz, $CDCl_3$, 300 K): δ [ppm] = 209.9 (s, C3), 172.9 (s, C1), 149.3 (s, C5'), 136.0 (s, C2'), 129.0 (d, C3'), 125.2 (d, C4'), 107.6 (d, C4), 102.2 (s, C2), 34.5 (t, C1'), 34.5 (s, *C*6'[CH_3]$_3$), 33.1 (s, *C*5[CH_3]$_3$), 31.5 (s, C6'[*C*H_3]$_3$), 29.9 (s, C5[*C*H_3]$_3$).

Analytical data matched those previously reported in the literature.[7]

Methyl (*R*)-2-(4-(tert-butyl)benzyl)-5,5-dimethylhexa-2,3-dienoate (2g)

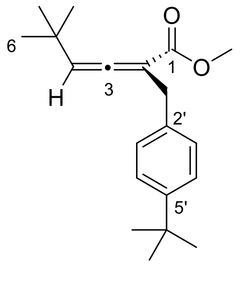

$C_{20}H_{28}O_2$
300.44 g/mol

*Synthesis of rac-**2g**:*

According to **GP3** allene carboxylic acid *rac*-**1g** (28.6 mg, 100 µmol, 1.00 eq.) was reacted with trimethylsilyldiazomethane (2.1 M in hexane, 95.2 µL, 200 µmol, 2.00 eq.) to afford the methyl ester *rac*-**2g** (28.5 mg, 94.9 µmol, 95%) as a colorless oil.

Photochemical Deracemization of Allene Carboxylic Acid:

According to **GP4** allene carboxylic acid *rac*-**1g** (14.3 mg, 50.0 µmol, 1.00 eq.) was reacted with phosphoric acid catalyst **3f** (3.67 mg, 5.00 µmol, 10 mol%). After removing the solvent under reduced pressure, the crude product was subjected to the esterification conditions described in **GP3**. After column chromatography, enantioenriched methyl ester **2g** (14.2 mg, 45.8 µmol, 92%, e.r. = 84/16) was isolated as a colorless oil.

TLC: R_f = 0.68 (Hex/EtOAc = 80/20) [UV/$KMnO_4$].

^{1}**H NMR** (500 MHz, $CDCl_3$, 300 K): δ [ppm] = 7.33 – 7.27 (m, 2H, C4'-H), 7.16 – 7.10 (m, 2H, C3'-H), 5.40 (*virt.* t, $^5J \approx {}^5J$ = 2.8 Hz, 1H, C4-H), 3.73 (s, 3H, CO_2CH_3), 3.55 (dd, ^2J = 15.3 Hz, ^5J = 2.8 Hz, 1H, C1'-H^a), 3.50 (dd, ^2J = 15.3 Hz, ^5J = 2.8 Hz, 1H, C1'-H^b), 1.29 (s, 9H, C6'[CH_3]$_3$), 0.92 (s, 9H, C5[CH_3]$_3$).

^{13}**C NMR** (101 MHz, $CDCl_3$, 300 K): δ [ppm] = 208.7 (s, C3), 168.1 (s, C1), 149.2 (s, C5'), 136.3 (s, C2'), 129.0 (d, C3'), 125.2 (d, C4'), 107.2 (d, C4), 102.4 (s, C2), 52.2 (q, CO_2*C*H_3), 35.0 (t, C1'), 34.5 (s, *C*6'[CH_3]$_3$), 33.0 (s, *C*5[CH_3]$_3$), 31.5 (q, C6'[*C*H_3]$_3$), 29.9 (q, C5[*C*H_3]$_3$).

IR (ATR): ṽ (cm^{-1}) = 2958 (m), 1957 (w), 1714 (s), 1436 (w), 1266 (m), 841 (w), 700 (w).

HRMS (ESI): calc. for [M + H^+]: 301.2163; found: 301.2158.

Chiral HPLC: t_{R1} = 21.9 min t_{R2} = 22.6 min (*Daicel* Chiralcel OJ-RH, 150×4.6 mm, MeCN/H_2O = 20/80 → 100/0, 1 mL/min, λ = 215 nm).

(*R*)-5,5-Dimethyl-2-(4-(methylthio)benzyl)hexa-2,3-dienoic acid (1h)

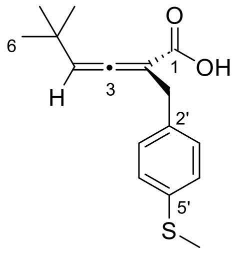

$C_{16}H_{20}O_2S$
276.39 g/mol

*Synthesis of rac-**1h**:*

According to **GP2** the respective ethyl allenoate[7] (1.70 g, 5.58 mmol, 1.00 eq.) was dissolved in EtOH (6 mL) and water (6 mL). NaOH (246 mg, 6.14 mmol, 1.10 eq.) was added, and the reaction mixture was heated at 100 °C for three hours. After cooling to room temperature 4 N HCl_{aq} was added until an acidic pH (pH = 1-2) was reached. The reaction mixture was extracted with EtOAc (3 × 15 mL), the combined organic phases were dried over Na_2SO_4, filtered and the solvent was removed under reduced pressure. The obtained crude acid was purified by flash column chromatography (silica, P/Et_2O = 90/10 → 85/15) to obtain allene carboxylic acid *rac*-**1h** (1.30 g, 4.71 mmol, 84%) as a colorless solid.

Photochemical Deracemization of Allene Carboxylic Acid:

According to **GP4** allene carboxylic acid *rac*-**1h** (13.8 mg, 50.0 µmol, 1.00 eq.) was reacted with phosphoric acid catalyst **3f** (3.67 mg, 5.00 µmol, 10 mol%). After removing the solvent under reduced pressure, the crude product was purified by flash column chromatography to obtain the enantioenriched allene carboxylic acid **1h** (11.4 mg, 41.2 µmol, 83%, e.r. = 81/19) as a colorless solid.

TLC: R_f = 0.38 (Hex/EtOAc = 70/30) [UV/$KMnO_4$].

^{1}H NMR (400 MHz, $CDCl_3$, 300 K): δ [ppm] = 7.22 – 7.17 (m, 2H, C4'-H), 7.16 – 7.10 (m, 2H, C3'-H), 5.51 (*virt.* t, $^5J \approx {}^5J$ = 2.6 Hz, 1H, C4-H), 3.53 (dd, ^2J = 15.2 Hz, ^5J = 2.6 Hz, 1H,

C1'-H[a]), 3.48 (dd, ^2J = 15.2 Hz, ^5J = 2.6 Hz, 1H, C1'-H[b]), 2.46 (s, 3H, SCH_3), 0.98 (s, 9H, $C[CH_3]_3$).

^{13}C NMR (101 MHz, $CDCl_3$, 300 K): δ [ppm] = 209.7 (s, C3), 171.9 (s, C1), 136.4 (s, C5'), 136.2 (s, C2'), 129.8 (d, C3'), 127.2 (d, C4'), 101.7 (d, C4), 34.7 (t, C1'), 33.2 (s, *C*[CH_3]$_3$), 30.0 (q, C[*C*H_3]$_3$), 16.5 (q, SCH_3).

Chiral HPLC: t_{R1} = 15.9 min t_{R2} = 16.7 min (*Daicel* Chiralcel OJ-RH, 150×4.6 mm, MeCN/H_2O = 20/80 → 100/0, 1 mL/min, λ = 215 nm).

NMR data matched those previously reported in the literature.[7]

2-(3,5-Dimethylbenzyl)-5,5-dimethylhexa-2,3-dienoic acid (*rac*-1i)

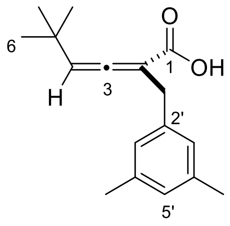

$C_{17}H_{22}O_2$
258.36 g/mol

According to **GP2** the respective ethyl allenoate[7] (900 mg, 3.14 mmol, 1.00 eq.) was dissolved in EtOH (3 mL) and water (3 mL). NaOH (138 mg, 3.46 mmol, 1.10 eq.) was added, and the reaction mixture was heated at 100 °C for three hours. After cooling to room temperature 4 N HCl_{aq} was added until an acidic pH (pH = 1-2) was reached. The reaction mixture was extracted with EtOAc (3 × 10 mL), the combined organic phases were dried over Na_2SO_4, filtered and the solvent was removed under reduced pressure. The obtained crude acid was purified by flash column chromatography (silica, P/Et_2O = 95/5 → 85/15) to obtain allene carboxylic acid *rac*-**1i** (583 mg, 2.25 mmol, 72%) as a colorless solid.

TLC: R_f = 0.61 (Hex/EtOAc = 70/30) [UV/$KMnO_4$].

^{1}H NMR (400 MHz, $CDCl_3$, 300 K): δ [ppm] = 6.84 – 6.81 (m, 3H, C3'-H, C5'-H), 5.50 (*virt.* t, $^5J \approx {}^5J$ = 2.6 Hz, 1H, C4-H), 3.51 (dd, ^2J = 15.1 Hz, ^5J = 2.6 Hz, 1H, C1'-H[a]), 3.44 (dd, ^2J = 15.1 Hz, ^5J = 2.6 Hz, 1H, C1'-H[b]), 2.27 (s, 6H, C4'-CH_3), 0.99 (s, 9H, $C[CH_3]_3$).

^{13}C NMR (101 MHz, $CDCl_3$, 300 K): δ [ppm] = 209.9 (s, C3), 172.4 (s, C1), 139.0 (s, C2'), 137.8 (s, *C*4'-CH_3), 128.0 (d, C5'), 127.0 (d, C3'), 107.5 (d, C4), 101.9 (s, C2), 34.9 (t, C1'), 33.2 (s, *C*[CH_3]$_3$), 30.0 (q, C[*C*H_3]$_3$), 21.4 (q, C4'-*C*H_3).

Analytical data matched those previously reported in the literature.[7]

Methyl (*R*)-2-(3,5-dimethylbenzyl)-5,5-dimethylhexa-2,3-dienoate (2i)

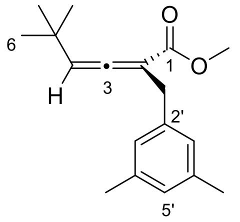

$C_{18}H_{24}O_2$
272.39 g/mol

*Synthesis of rac-**2j**:*

According to **GP3** allene carboxylic acid *rac*-**1i** (25.8 mg, 100 µmol, 1.00 eq.) was reacted with trimethylsilyldiazomethane (2.1 M in hexane, 95.2 µL, 200 µmol, 2.00 eq.) to afford the methyl ester *rac*-**2i** (27.0 mg, 99.3 µmol, 99%) as a colorless oil.

Photochemical Deracemization of Allene Carboxylic Acid:

According to **GP4** allene carboxylic acid *rac*-**1i** (12.9 mg, 50.0 µmol, 1.00 eq.) was reacted with phosphoric acid catalyst **3f** (3.67 mg, 5.00 µmol, 10 mol%). After removing the solvent under reduced pressure, the crude product was subjected to the esterification conditions described in **GP3**. After column chromatography, enantioenriched methyl ester **2i** (13.2 mg, 48.5 µmol, 97%, e.r. = 85/15) was isolated as a colorless oil.

TLC: R_f = 0.58 (Hex/EtOAc = 90/10) [UV/$KMnO_4$].

^{1}H NMR (400 MHz, $CDCl_3$, 300 K): δ [ppm] = 6.96 – 6.72 (m, 3H, C3'-H, C5'-H), 5.43 (*virt.* t, $^5J \approx {}^5J$ = 2.6 Hz, 1H, C4-H), 3.72 (s, 3H, CO_2CH_3), 3.52 (dd, ^2J = 15.0 Hz, ^5J = 2.6 Hz, 1H, C1'-H^a), ^2J = 15.0 Hz, ^5J = 2.6 Hz, 1H, C1'-H^b), 2.27 (s, 6H, C4'-CH_3), 0.98 (s, 9H, C[CH_3]$_3$).

^{13}C NMR (101 MHz, $CDCl_3$, 300 K): δ [ppm] = 208.8 (s, C3), 168.1 (s, *C*O_2CH_3), 139.2 (s, C2'), 137.7 (s, C4'), 128.0 (d, C5'), 127.1 (d, C3'), 107.0 (d, C4), 102.2 (s, C2), 52.2 (q, CO_2*C*H_3), 35.3 (t, C1'), 33.1 (s, *C*[CH_3]$_3$), 30.0 (q, C[*C*H_3]$_3$), 21.4 (q, C4'-*C*H_3).

IR (ATR): ṽ (cm^{-1}) = 2960 (w), 1956 (w), 1713 (s), 1435 (w), 1250 (s), 1079 (s), 707 (w).

HRMS (ESI): calc. for [M + H$^+$]: 273.1850; found: 273.1844.

Chiral HPLC: t_{R1} = 19.1 min t_{R2} = 19.7 min (*Daicel* Chiralcel OJ-RH, 150×4.6 mm, MeCN/H_2O = 20/80 → 100/0, 1 mL/min, λ = 215 nm).

2-(3,5-Di-*tert*-butylbenzyl)-5,5-dimethylhexa-2,3-dienoic acid (*rac*-1j)

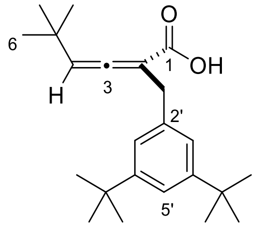

$C_{23}H_{34}O_2$
342.52 g/mol

Synthesis of the respective Ethyl Allenoate:

According to **GP1** 1-(bromomethyl)-3,5-di-*tert*-butylbenzene (1.99 g, 7.04 mmol, 1.27 eq.) was reacted with (carbethoxymethylene)triphenylphosphorane (1.93 g, 5.54 mmol, 1.00 eq.) to obtain the benzylated phosphonium salt. The salt was further converted with NEt_3 (1.23 g, 1.70 mL, 12.2 mmol, 2.20 eq.) and 3,3-dimethylbutanoyl chloride (746 mg, 799 µL, 5.54 mmol, 1.00 eq.) to afford after flash column chromatography the desired ethyl allenoate (1.45 g, 3.92 mmol, 71%) as a slightly yellow oil. The compound was used without further characterization in the next step.

*Synthesis of rac-**1j**:*

According to **GP2** the respective ethyl allenoate (1.36 g, 3.67 mmol, 1.00 eq.) was dissolved in EtOH (3 mL) and water (3 mL). NaOH (161 mg, 4.04 mmol, 1.10 eq.) was added, and the reaction mixture was heated at 100 °C for three hours. After cooling to room temperature 4 N HCl_{aq} was added until an acidic pH (pH = 1-2) was reached. The reaction mixture was extracted with EtOAc (3 × 10 mL), the combined organic phases were dried over Na_2SO_4, filtered and the solvent was removed under reduced pressure. The obtained crude acid was purified by flash column chromatography (silica, P/Et_2O = 95/5 → 85/15) to obtain allene carboxylic acid *rac*-**1j** (620 mg, 1.81 mmol, 49%) as a colorless solid.

TLC: R_f = 0.63 (Hex/EtOAc = 70/30) [UV/$KMnO_4$].

M.p.: 133 °C

^{1}H NMR (400 MHz, $CDCl_3$, 300 K): δ [ppm] = 7.28 – 7.21 (m, 1H, C5'-H), 7.03 (d, ^4J = 1.9 Hz, C3'-H), 5.43 (*virt.* t, $^5J \approx {}^5J$ = 2.9 Hz, 1H, C4-H), 3.58 (dd, ^2J = 15.3 Hz, ^5J = 2.9 Hz, 1H, C1'-H^a), 3.52 (dd, ^2J = 15.3 Hz, ^5J = 2.9 Hz, 1H, C1'-H^b), 1.30 (s, 18H, C4'-C[CH_3]$_3$), 0.88 (s, 9H, C5[CH_3]$_3$).

^{13}**C NMR** (101 MHz, $CDCl_3$, 300 K): δ [ppm] = 209.8 (s, C3), 172.4 (s, C2), 150.6 (s, C4'), 137.9 (s, C2'), 123.7 (d, C3'), 120.4 (d, C5'), 107.7 (d, C4), 102.6 (s, C2), 35.6 (t, C1'), 34.9 (s, C4'-*C*[CH_3]$_3$), 33.1 (s, *C*5[CH_3]$_3$), 31.7 (q, C4'-C[*C*H_3]$_3$), 29.9 (q, C5[*C*H_3]$_3$).

IR (ATR): ṽ (cm^{-1}) = 2959 (m), 1952 (m), 1670 (s), 1275 (m), 1252 (m), 876 (m), 726 (m).

HRMS (ESI): calc. for [M + H^+]: 343.2632; found: 343.2626.

Methyl (*R*)-2-(3,5-di-*tert*-butylbenzyl)-5,5-dimethylhexa-2,3-dienoate (2j)

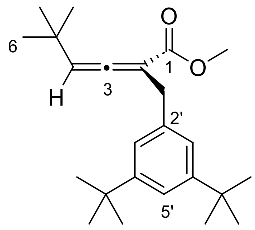

$C_{24}H_{36}O_2$
356.55 g/mol

*Synthesis of rac-**2j**:*

According to **GP3** allene carboxylic acid *rac*-**1j** (34.3 mg, 100 µmol, 1.00 eq.) was reacted with trimethylsilyldiazomethane (2.1 M in hexane, 95.2 µL, 200 µmol, 2.00 eq.) to afford the methyl ester *rac*-**2j** (34.5 mg, 96.7 µmol, 97%) as a colorless solid.

Photochemical Deracemization of Allene Carboxylic Acid:

According to **GP4** allene carboxylic acid *rac*-**1j** (17.1 mg, 50.0 µmol, 1.00 eq.) was reacted with phosphoric acid catalyst **3f** (3.67 mg, 5.00 µmol, 10 mol%). After removing the solvent under reduced pressure, the crude product was subjected to the esterification conditions described in **GP3**. After column chromatography, enantioenriched methyl ester **2j** (16.6 mg, 46.6 µmol, 93%, e.r. = 83/17) was isolated as a colorless solid.

TLC: R_f = 0.74 (Hex/EtOAc = 80/20) [UV/$KMnO_4$].

M.p.: 60 °C.

^{1}**H NMR** (500 MHz, $CDCl_3$, 300 K): δ [ppm] = 7.25 – 7.23 (m, 1H, C5'-H), 7.02 (d, ^4J = 1.8 Hz, C3'-H), 5.35 (*virt.* t, $^5J \approx {}^5J$ = 3.0 Hz, 1H, C4-H), 3.74 (s, 3H CO_2CH_3), 3.58 (dd, ^2J = 15.3 Hz, ^5J = 3.0 Hz, 1H, C1'-H^a), 3.52 (dd, ^2J = 15.3 Hz, ^5J = 3.0 Hz, 1H, C1'-H^b), 1.30 (s, 18H, C4'-C[CH_3]$_3$), 0.86 (s, 9H, C5[CH_3]$_3$).

^{13}C NMR (126 MHz, $CDCl_3$, 300 K): δ [ppm] = 208.6 (s, C3), 168.1 (s, CO_2CH_3), 150.5 (s, C4'), 138.0 (s, C2'), 123.7 (d, C3'), 120.3 (d, C5'), 107.3 (d, C4), 102.8 (s, C2), 52.3 (q, CO_2CH_3), 36.0 (t, C1'), 34.8 (s, C4'-$C[CH_3]_3$), 32.9 (s, $C5[CH_3]_3$), 31.6 (q, C4'-$C[CH_3]_3$), 29.9 (q, $C5[CH_3]_3$).

IR (ATR): ṽ (cm^{-1}) = 2958 (m), 1956 (w), 1705 (s), 1251 (s), 1266 (m), 876 (w), 711 (w).

HRMS (ESI): calc. for [M + H^+]: 357.2789; found: 357.2781.

Chiral HPLC: t_{R1} = 21.5 min t_{R2} = 22.9 min (*Daicel* Chiralcel OJ-RH, 150×4.6 mm, $MeCN/H_2O$ = 20/80 → 100/0, 1 mL/min, λ = 215 nm).

2-([1,1'-Biphenyl]-3-ylmethyl)-5,5-dimethylhexa-2,3-dienoic acid (*rac*-1k)

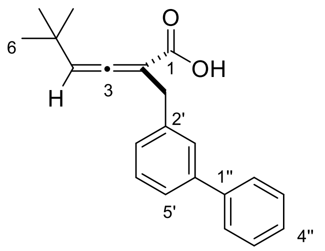

$C_{21}H_{22}O_2$
306.41 g/mol

Synthesis of the respective Ethyl Allenoate:

According to **GP1** 3-(bromomethyl)-1,1'-biphenyl (1.74 g, 7.04 mmol, 1.27 eq.) was reacted with (carbethoxymethylene)triphenylphosphorane (1.93 g, 5.54 mmol, 1.00 eq.) to obtain the benzylated phosphonium salt. The salt was further converted with NEt_3 (1.23 g, 1.70 mL, 12.2 mmol, 2.20 eq.) and 3,3-dimethylbutanoyl chloride (746 mg, 799 µL, 5.54 mmol, 1.00 eq.) to afford after flash column chromatography the desired ethyl allenoate (1.25 g, 3.74 mmol, 68%) as a colorless oil. The compound was used without further characterization in the next step.

*Synthesis of rac-**1k**:*

According to **GP2** the respective ethyl allenoate (1.20 g, 3.59 mmol, 1.00 eq.) was dissolved in EtOH (4 mL) and water (4 mL). NaOH (158 mg, 3.95 mmol, 1.10 eq.) was added, and the reaction mixture was heated at 100 °C for three hours. After cooling to room temperature 4 N HCl_{aq} was added until an acidic pH (pH = 1-2) was reached. The reaction mixture was extracted with EtOAc (3 × 15 mL), the combined organic phases were dried over Na_2SO_4, filtered and the solvent was removed under reduced pressure. The obtained crude acid was purified by flash

column chromatography (silica, P/Et_2O = 95/5 → 75/25) to obtain allene carboxylic acid *rac*-**1k** (582 mg, 1.90 mmol, 53%) as a colorless oil.

TLC: R_f = 0.63 (Hex/EtOAc = 70/30) [UV/$KMnO_4$].

^{1}H NMR (400 MHz, $CDCl_3$, 300 K): δ [ppm] = 7.61 – 7.54 (m, 2H, C2"-H), 7.47 – 7.41 (m, 4H, C3'-H, C5'-H, C3"-H), 7.38 – 7.32 (m, 2H, C6'-H, C4"-H), 7.23 – 7.19 (m, 1H, C7'-H), 5.52 (*virt.* t, $^5J \approx {}^5J$ = 2.7 Hz, 1H, C4-H), 3.66 (dd, ^2J = 15.3 Hz, ^5J = 2.7 Hz, 1H, C1'-H^a), 3.60, (dd, ^2J = 15.3 Hz, ^5J = 2.7 Hz, 1H, C1'-H^b), 0.96 (s, 9H, C[CH_3]$_3$).

^{13}C NMR (101 MHz, $CDCl_3$, 300 K): δ [ppm] = 210.0 (s, C3), 172.8 (s, C1), 141.4 (s, C1"), 141.4 (s, C4'), 139.7 (s, C2'), 128.8 (d, C3"), 128.8 (d, C3'), 128.3 (d, C6'), 128.2 (d, C7'), 127.3 (d, C4"), 127.3 (d, C2"), 125.4 (d, C5'), 107.8 (d, C4), 102.0 (s, C2), 35.2 (t, C1'), 33.2 (s, *C*[CH_3]$_3$), 29.9 (q, C[*C*H_3]$_3$).

IR (ATR): ṽ (cm^{-1}) = 2961 (m), 1954 (w), 1676 (s), 1277 (m), 1251 (m), 754 (s), 697 (s).

HRMS (ESI): calc. for [M + H^+]: 307.1693; found: 307.1687.

Methyl (*R*)-2-([1,1'-biphenyl]-3-ylmethyl)-5,5-dimethylhexa-2,3-dienoate (2k)

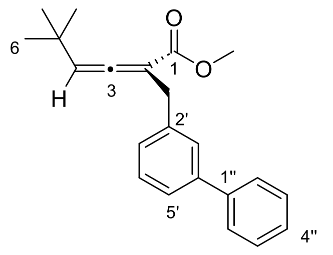

$C_{22}H_{24}O_2$
320.43 g/mol

*Synthesis of rac-**2k**:*

According to **GP3** allene carboxylic acid *rac*-**1k** (29.2 mg, 100 µmol, 1.00 eq.) was reacted with trimethylsilyldiazomethane (2.1 M in hexane, 95.2 µL, 200 µmol, 2.00 eq.) to afford the methyl ester *rac*-**2k** (30.0 mg, 98.0 µmol, 98%) as a colorless oil.

Photochemical Deracemization of Allene Carboxylic Acid:

According to **GP4** allene carboxylic acid *rac*-**1k** (15.3 mg, 50.0 µmol, 1.00 eq.) was reacted with phosphoric acid catalyst **3f** (3.67 mg, 5.00 µmol, 10 mol%). After removing the solvent under reduced pressure, the crude product was subjected to the esterification conditions

described in **GP3**. After column chromatography, enantioenriched methyl ester **2k** (13.5 mg, 42.2 µmol, 84%, e.r. = 81/19) was isolated as a colorless oil.

TLC: R_f = 0.74 (Hex/EtOAc = 80/20) [UV/$KMnO_4$].

^{1}H NMR (400 MHz, $CDCl_3$, 300 K): δ [ppm] = 7.60 – 7.54 (m, 2H, C2”-H), 7.46 – 7.40 (m, 4H, C3’-H, C5’-H, C3”-H), 7.37 – 7.30 (m, 2H, C6’-H, C4”-H), 7.24 – 7.14 (m, 1H, C7’-H), 5.45 (*virt.* t, $^5J \approx {}^5J$ = 2.8 Hz, 1H, C4-H), 3.74 (s, 3H, CO_2CH_3), 3.66 (dd, ^2J = 15.2 Hz, ^5J = 2.8 Hz, 1H, C1’-H^a), 3.60 (dd, ^2J = 15.2 Hz, ^5J = 2.8 Hz, 1H, C1’-H^b), 0.94 (s, 9H, C[CH_3]$_3$).

^{13}C NMR (101 MHz, $CDCl_3$, 300 K): δ [ppm] = 208.8 (s, C3), 168.0 (s, *C*O_2CH_3), 141.5 (s, C1”), 141.3 (s, C4’), 139.9 (s, C2’), 128.8 (d, C3”), 128.7 (d, C3’), 128.3 (d, C6’), 128.2 (d, C7’), 127.3 (d, C4”), 125.3 (d, C5’), 107.4 (d, C4), 102.2 (s, C2), 52.3 (q, CO_2*C*H_3), 35.7 (t, C1’), 33.0 (s, *C*[CH_3]$_3$), 30.0 (q, C[*C*H_3]$_3$).

IR (ATR): ṽ (cm^{-1}) = 2960 (m), 1956 (w), 1710 (s), 1250 (s), 1075 (s), 754 (s), 697 (s).

HRMS (ESI): calc. for [M + H^+]: 321.1850; found: 321.1834.

Chiral HPLC: t_{R1} = 21.9 min t_{R2} = 22.7 min (*Daicel* Chiralcel OJ-RH, 150×4.6 mm, MeCN/H_2O = 20/80 → 100/0, 1 mL/min, λ = 215 nm).

(*R*)-2-(3-Cyanobenzyl)-5,5-dimethylhexa-2,3-dienoic acid (1l)

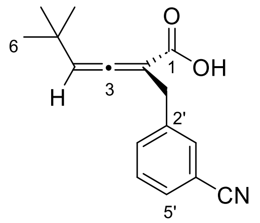

$C_{16}H_{17}NO_2$
255.32 g/mol

*Synthesis of rac-**1l**:*

According to **GP2** the respective ethyl allenoate[7] (668 mg, 1.98 mmol, 1.00 eq.) was dissolved in EtOH (2 mL) and water (2 mL). NaOH (87.1 mg, 2.18 mmol, 1.10 eq.) was added, and the reaction mixture was heated at 100 °C for three hours. After cooling to room temperature 4 N HCl_{aq} was added until an acidic pH (pH = 1-2) was reached. The reaction mixture was extracted with EtOAc (3 × 10 mL), the combined organic phases were dried over Na_2SO_4, filtered and the solvent was removed under reduced pressure. The obtained crude acid was purified by flash

column chromatography (silica, P/Et_2O = 90/10 → 85/15) to obtain allene carboxylic acid *rac*-**1l** (182 mg, 713 µmol, 36%) as a colorless solid.

Photochemical Deracemization of Allene Carboxylic Acid:

According to **GP4** allene carboxylic acid *rac*-**1l** (12.8 mg, 50.0 µmol, 1.00 eq.) was reacted with phosphoric acid catalyst **3f** (3.67 mg, 5.00 µmol, 10 mol%). After removing the solvent under reduced pressure, the crude product was purified by flash column chromatography to obtain the enantioenriched allene carboxylic acid **1l** (8.2 mg, 32.1 µmol, 64%, e.r. = 71/29) as a colorless solid.

TLC: R_f = 0.35 (Hex/EtOAc = 70/30) [UV/$KMnO_4$].

^{1}H NMR (400 MHz, $CDCl_3$, 300 K): δ [ppm] = 7.52 – 7.49 (m, 2H, C3'-H, C5'-H), 7.48 – 7.44 (m, 1H, C7'-H), 7.42 – 7.35 (m, 1H, C6'-H), 5.56 (*virt.* t, $^5J \approx {}^5J$ = 2.6 Hz, 1H, C4-H), 3.60 (dd, ^2J = 15.3 Hz, ^5J = 2.6 Hz, 1H, C1'-H^a), 3.55 (dd, ^2J = 15.3 Hz, ^5J = 2.6 Hz, 1H, C1'-H^b), 0.98 (s, 9H, C[CH_3]$_3$).

^{13}C NMR (101 MHz, $CDCl_3$, 300 K): δ [ppm] = 209.9 (s, C3), 172.3 (s, C1), 140.8 (s, C2'), 133.8 (d, C7'), 132.8 (d, C3'), 130.3 (d, C5'), 129.2 (d, C6'), 119.0 (s, CN), 112.5 (s, C4'), 108.2 (d, C4), 100.9 (s, C2), 34.7 (t, C1'), 33.3 (s, *C*[CH_3]$_3$), 30.0 (q, C[*C*H_3]$_3$).

Chiral HPLC: t_{R1} = 7.6 min t_{R2} = 9.5 min (*Daicel* Chiralpak IC, 250×4.6 mm, n-Hep/iso-PrOH = 90/10, 1 mL/min, λ = 210 nm).

NMR data matched those previously reported in the literature.[7]

Methyl (*R*)-2-(3-cyanobenzyl)-5,5-dimethylhexa-2,3-dienoate (2l)

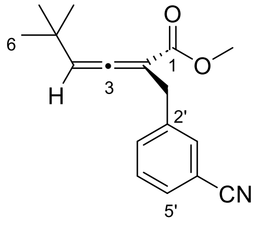

$C_{17}H_{19}NO_2$
269.34 g/mol

*Synthesis of rac-**2l**:*

According to **GP3** allene carboxylic acid *rac*-**1l** (25.5 mg, 100 µmol, 1.00 eq.) was reacted with trimethylsilyldiazomethane (2.1 M in hexane, 95.2 µL, 200 µmol, 2.00 eq.) to afford the methyl ester *rac*-**2l** (25.7 mg, 95.5 µmol, 96%) as a colorless oil.

Photochemical Deracemization of Allene Carboxylic Acid:

According to **GP4** allene carboxylic acid *rac*-**1l** (12.8 mg, 50.0 µmol, 1.00 eq.) was reacted with phosphoric acid catalyst **3f** (3.67 mg, 5.00 µmol, 10 mol%). After removing the solvent under reduced pressure, the crude product was subjected to the esterification conditions described in **GP3**. After column chromatography, enantioenriched methyl ester **2l** (12.1 mg, 47.3 µmol, 95%, e.r. = 70/30) was isolated as a colorless oil.

TLC: R_f = 0.72 (Hex/EtOAc = 70/30) [UV/$KMnO_4$].

^{1}H NMR (400 MHz, $CDCl_3$, 300 K): δ [ppm] = 7.52 – 7.48 (m, 2H, C3'-H, C5'-H), 7.48 – 7.43 (m, 1H, C7'), 7.41 – 7.34 (m, 1H, C6'-H), 5.49 (*virt.* t, $^5J \approx {}^5J$ = 2.8 Hz, 1H, C4-H), 3.73 (s, 3H, CO_2CH_3), 3.61 dd, ^2J = 15.3 Hz, ^5J = 2.8 Hz, 1H, C1'-H^a), 3.56 (dd, ^2J = 15.3 Hz, ^5J = 2.8 Hz, 1H, C1'-H^b), 0.96 (s, 9H, C[CH_3]$_3$).

^{13}C NMR (101 MHz, $CDCl_3$, 300 K): δ [ppm] = 208.6 (s, C3), 167.6 (s, C1), 141.0 (s, C2'), 133.9 (d, C7'), 132.8 (d, C3'), 130.3 (d, C5'), 129.2 (d, C6'), 119.1 (s, CN), 112.4 (s, C4'), 107.9 (d, C4), 101.2 (s, C2), 52.5 (q, CO_2CH_3), 35.2 (t, C1'), 33.1 (s, *C*[CH_3]$_3$), 30.0 (q, C[*C*H_3]$_3$).

IR (ATR): ṽ (cm^{-1}) = 2961 (w), 2230 (w), 1956 (w), 1709 (s), 1435 (w), 1269 (m), 687 (m).

HRMS (ESI): calc. for [M + H^+]: 270.1489; found: 270.1483.

Chiral HPLC: t_{R1} = 16.4 min t_{R2} = 16.9 min (*Daicel* Chiralcel OJ-RH, 150×4.6 mm, MeCN/H_2O = 20/80 → 100/0, 1 mL/min, λ = 215 nm).

(*R*)-2-(3-Methoxybenzyl)-5,5-dimethylhexa-2,3-dienoic acid (1m)

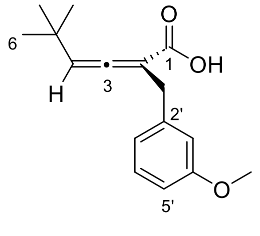

$C_{16}H_{20}O_3$
260.33 g/mol

Synthesis of the respective Ethyl Allenoate:

According to **GP1** 1-(bromomethyl)-3-methoxybenzene (1.41 g, 985 µL, 7.04 mmol, 1.27 eq.) was reacted with (carbethoxymethylene)triphenylphosphorane (1.93 g, 5.54 mmol, 1.00 eq.) to

obtain the benzylated phosphonium salt. The salt was further converted with NEt_3 (1.23 g, 1.70 mL, 12.2 mmol, 2.20 eq.) and 3,3-dimethylbutanoyl chloride (746 mg, 799 µL, 5.54 mmol, 1.00 eq.) to afford after flash column chromatography the desired ethyl allenoate (821 mg, 2.84 mmol, 51%) as a colorless oil. The compound was used without further characterization in the next step.

*Synthesis of rac-**1m**:*

According to **GP2** the respective ethyl allenoate (776 mg, 2.69 mmol, 1.00 eq.) was dissolved in EtOH (3 mL) and water (3 mL). NaOH (118 mg, 2.96 mmol, 1.10 eq.) was added, and the reaction mixture was heated at 100 °C for three hours. After cooling to room temperature 4 N HCl_{aq} was added until an acidic pH (pH = 1-2) was reached. The reaction mixture was extracted with EtOAc (3 × 10 mL), the combined organic phases were dried over Na_2SO_4, filtered and the solvent was removed under reduced pressure. The obtained crude acid was purified by flash column chromatography (silica, P/Et_2O = 95/5 → 85/15) to obtain allene carboxylic acid *rac*-**1m** (351 mg, 1.35 mmol, 50%) as a colorless solid.

Photochemical Deracemization of Allene Carboxylic Acid:

According to **GP4** allene carboxylic acid *rac*-**1m** (13.0 mg, 50.0 µmol, 1.00 eq.) was reacted with phosphoric acid catalyst **3f** (3.67 mg, 5.00 µmol, 10 mol%). After removing the solvent under reduced pressure, the crude product was purified by flash column chromatography to obtain the enantioenriched allene carboxylic acid **1m** (10.1 mg, 38.8 µmol, 78%, e.r. = 84/16) as a colorless solid.

TLC: R_f = 0.31 (Hex/EtOAc = 70/30) [UV/$KMnO_4$].

M.p.: 54 °C.

^{1}H NMR (400 MHz, $CDCl_3$, 300 K): δ [ppm] = 7.22 – 7.14 (m, 1H, C6'-H), 6.83 – 6.79 (m, 1H, C7'-H), 6.79 – 6.71 (m, 2H, C3'-H, C5'-H), 5.51 (*virt.* t, $^5J \approx {}^5J$ = 2.6 Hz, 1H, C4-H), 3.79 (s, 3H, OCH_3), 3.56 (dd, ^2J = 15.1 Hz, ^5J = 2.6 Hz, 1H, C1'-H^a), 3.50 (dd, ^2J = 15.1 Hz, ^5J = 2.6 Hz, 1H, C1'-H^b), 0.99 (s, 9H, $C[CH_3]_3$).

^{13}C NMR (101 MHz, $CDCl_3$, 300 K): δ [ppm] = 209.9 (s, C3), 172.5 (s, C1), 159.7 (s, C4'), 140.8 (s, C2'), 129.3 (d, C6'), 121.7 (d, C7'), 114.9 (d, C3'), 112.0 (d, C5'), 107.6 (d, C4), 101.8 (s, C2), 55.3 (q, OCH_3), 35.2 (t, C1'), 33.2 (s, $\mathit{C}[CH_3]_3$), 30.0 (q, $C[\mathit{C}H_3]_3$).

IR (ATR): ṽ (cm^{-1}) = 2957 (w), 1953 (w), 1671(s), 1259 (s), 1167 (m), 783 (m), 707 (s).

HRMS (ESI): calc. for [M + H$^+$]: 261.1486; found: 261.1486.

Chiral HPLC: t_{R1} = 7.2 min t_{R2} = 8.5 min (*Daicel* Chiralpak AD-H, 250×4.6 mm, n-Hep/iso-PrOH = 90/10, 1 mL/min, λ = 210 nm).

(*R*)-2-(Benzo[*d*][1,3]dioxol-5-ylmethyl)-5,5-dimethylhexa-2,3-dienoic acid (1n)

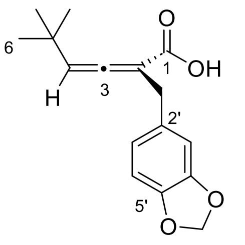

$C_{16}H_{18}O_4$
274.32 g/mol

Synthesis of the respective Ethyl Allenoate:

According to **GP1** 5-(bromomethyl)benzo[*d*][1,3]dioxole (956 mg, 4.45 mmol, 1.27 eq.) was reacted with (carbethoxymethylene)triphenylphosphorane (1.22 g, 3.50 mmol, 1.00 eq.) to obtain the benzylated phosphonium salt. The salt was further converted with NEt_3 (779 mg, 1.07 mL, 7.70 mmol, 2.20 eq.) and 3,3-dimethylbutanoyl chloride (471 mg, 505 µL, 3.50 mmol, 1.00 eq.) to afford after flash column chromatography the desired ethyl allenoate (522 mg, 1.72 mmol, 49%) as a colorless oil. The compound was used without further characterization in the next step.

*Synthesis of rac-**1n**:*

According to **GP2** the respective ethyl allenoate (488 mg, 1.61 mmol, 1.00 eq.) was dissolved in EtOH (2 mL) and water (2 mL). NaOH (71.0 mg, 1.78 mmol, 1.10 eq.) was added, and the reaction mixture was heated at 100 °C for three hours. After cooling to room temperature 4 N HCl_{aq} was added until an acidic pH (pH = 1-2) was reached. The reaction mixture was extracted with EtOAc (3 × 10 mL), the combined organic phases were dried over Na_2SO_4, filtered and the solvent was removed under reduced pressure. The obtained crude acid was purified by flash column chromatography (silica, P/Et_2O = 95/5 → 85/15) to obtain allene carboxylic acid *rac*-**1n** (205 mg, 746 µmol, 46%) as a colorless oil.

Photochemical Deracemization of Allene Carboxylic Acid:

According to **GP4** allene carboxylic acid *rac*-**1n** (13.7 mg, 50.0 µmol, 1.00 eq.) was reacted with phosphoric acid catalyst **3f** (3.67 mg, 5.00 µmol, 10 mol%). After removing the solvent

under reduced pressure, the crude product was purified by flash column chromatography to obtain the enantioenriched allene carboxylic acid **1n** (8.9 mg, 32.4 µmol, 65%, e.r. = 77/23) as a colorless oil.

TLC: R_f = 0.24 (Hex/EtOAc = 70/30) [UV/$KMnO_4$].

^{1}H NMR (400 MHz, $CDCl_3$, 300 K): δ [ppm] = 6.73 – 6.70 (m, 2H, C3'-H, C6'-H), 6.69 – 6.65 (m, 1H, C7'-H), 5.91 (s, 2H, OCH_2O), 5.52 (*virt.* t, $^5J \approx {}^5J$ = 2.6 Hz, 1H, C4-H), 3.49 (dd, ^2J = 15.2 Hz, ^5J = 2.6 Hz, 1H, C1'-H^a), 3.50 (dd, ^2J = 15.2 Hz, ^5J = 2.6 Hz, 1H, C1'-H^b), 1.01 (s, 9H, $C[CH_3]_3$).

^{13}C NMR (101 MHz, $CDCl_3$, 300 K): δ [ppm] = 209.8 (s, C3), 172.6 (s, C1), 147.6 (s, C4'), 146.2 (s, C5'), 133.1 (s, C2'), 122.2 (d, C7'), 109.8 (d, C3'), 108.2 (d, C6'), 107.6 (t, C1'), 102.0 (s, C2), 100.9 (t, OCH_2O), 34.9 (t, C1'), 33.3 (s, *C*$[CH_3]_3$), 30.0 (s, C[*C*$H_3]_3$).

IR (ATR): ṽ (cm^{-1}) = 2963 (w), 1957 (w), 1671 (s), 1259 (s), 1041 (m), 911 (w), 707 (s).

HRMS (ESI): calc. for [M + H^+]: 275.1278; found: 275.1280.

Chiral HPLC: t_{R1} = 9.6 min t_{R2} = 11.1 min (*Daicel* Chiralpak AD-H, 250×4.6 mm, n-Hep/iso-PrOH = 90/10, 1 mL/min, λ = 210 nm).

(*R*)-2-(2,6-Dichlorobenzyl)-5,5-dimethylhexa-2,3-dienoic acid (1o)

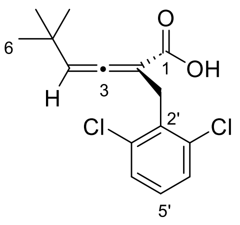

$C_{15}H_{16}Cl_2O_2$
299.19 g/mol

*Synthesis of rac-**1o**:*

According to **GP2** the respective ethyl allenoate[7] (717 mg, 2.19 mmol, 1.00 eq.) was dissolved in EtOH (2 mL) and water (2 mL). NaOH (245 mg, 6.13 mmol, 1.10 eq.) was added, and the reaction mixture was heated at 100 °C for three hours. After cooling to room temperature 4 N HCl_{aq} was added until an acidic pH (pH = 1-2) was reached. The reaction mixture was extracted with EtOAc (3 × 10 mL), the combined organic phases were dried over Na_2SO_4, filtered and the solvent was removed under reduced pressure. The obtained crude acid was purified by flash

column chromatography (silica, P/Et_2O = 90/10 → Et_2O) to obtain allene carboxylic acid *rac*-**1o** (496 mg, 1.66 mmol, 76%) as a colorless solid.

Photochemical Deracemization of Allene Carboxylic Acid:

According to **GP4** allene carboxylic acid *rac*-**1o** (15.0 mg, 50.0 µmol, 1.00 eq.) was reacted with phosphoric acid catalyst **3f** (3.67 mg, 5.00 µmol, 10 mol%). After removing the solvent under reduced pressure, the crude product was purified by flash column chromatography to obtain the enantioenriched allene carboxylic acid **1o** (12.1 mg, 40.4 µmol, 81%, e.r. = 77/23) as a colorless solid.

TLC: R_f = 0.41 (Hex/EtOAc = 70/30) [UV/$KMnO_4$].

^{1}H NMR (400 MHz, $CDCl_3$, 300 K): δ [ppm] = 7.28 (d, ^3J = 8.1 Hz, 2H, C4'-H), 7.14 – 7.04 (m, 1H, C5'-H), 5.43 (dd, ^5J = 4.4 Hz, ^5J = 3.1 Hz, 1H, C4-H), 3.99 (dd, ^2J = 15.9 Hz, ^5J = 3.1 Hz, 1H, C1'-H^a), 3.82 (dd, ^2J = 15.9 Hz, ^5J = 4.4 Hz, 1H, C1'-H^b), 0.82 (s, 9H, C[CH_3]$_3$).

^{13}C NMR (101 MHz, $CDCl_3$, 300 K): δ [ppm] = 208.4 (s, C3), 172.2 (s, C1), 136.3 (s, C3'), 135.5 (s, C2'), 128.3 (d, C5'), 128.1 (d, C4'), 109.0 (d, C4), 99.3 (s, C2), 33.1 (s, *C*[CH_3]$_3$), 30.2 (t, C1'), 29.7 (q, C[*C*H_3]$_3$).

Chiral HPLC: t_{R1} = 5.6 min t_{R2} = 6.3 min (*Daicel* Chiralcel AD-H, 250×4.6 mm, n-Hep/iso-PrOH = 90/10, 1 mL/min, λ = 210 nm).

NMR data matched those previously reported in the literature.[7]

(*R*)-5,5-Dimethyl-2-((perfluorophenyl)methyl)hexa-2,3-dienoic acid (1p)

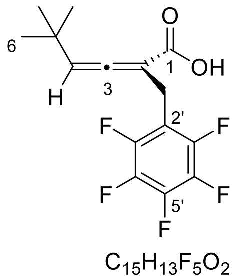

$C_{15}H_{13}F_5O_2$
320.26 g/mol

*Synthesis of rac-**1p**:*

According to **GP2** the respective ethyl allenoate[7] (1.94 g, 5.57 mmol, 1.00 eq.) was dissolved in EtOH (7 mL) and water (7 mL). NaOH (245 mg, 6.13 mmol, 1.10 eq.) was added, and the reaction mixture was heated at 100 °C for three hours. After cooling to room temperature 4 N

HCl_{aq} was added until an acidic pH (pH = 1-2) was reached. The reaction mixture was extracted with EtOAc (3 × 15 mL), the combined organic phases were dried over Na_2SO_4, filtered and the solvent was removed under reduced pressure. The obtained crude acid was purified by flash column chromatography (silica, P/Et_2O = 90/10 → 80/20) to obtain allene carboxylic acid *rac*-**1p**(395 mg, 1.24 mmol, 22%) as a colorless solid.

Photochemical Deracemization of Allene Carboxylic Acid:

According to **GP4** allene carboxylic acid *rac*-**1p** (16.1 mg, 50.0 µmol, 1.00 eq.) was reacted with phosphoric acid catalyst **3f** (3.67 mg, 5.00 µmol, 10 mol%). After removing the solvent under reduced pressure, the crude product was purified by flash column chromatography to obtain the enantioenriched allene carboxylic acid **1p** (9.2 mg, 28.8 µmol, 58%, e.r. = 71/29) as a colorless solid.

TLC: R_f = 0.34 (Hex/EtOAc = 70/30) [UV/$KMnO_4$].

^{1}H NMR (500 MHz, $CDCl_3$, 300 K): δ [ppm] = 5.56 (*virt.* t, $^5J \approx {}^5J$ = 3.3 Hz, 1H, C4-H), 3.72 – 3.66 (m, 1H, C1'-H^a), 3.66 – 3.61 (m, 1H, C1-H^b), 0.95 (s, 9H, C[CH_3]$_3$).

^{13}C NMR (126 MHz, $CDCl_3$, 300 K): δ [ppm] = 208.4 (s, C3), 171.1 (s, C1), 146.4 – 146.1 (m, C_{Ar}), 144.4 – 144.1 (m, C_{Ar}), 138.7 – 138.3 (m, C_{Ar}), 136.7 – 136.3 (m, C_{Ar}), 109.6 (d, C4), 98.9 (s, C2), 33.3 (t, C1'), 29.8 (s, *C*[CH_3]$_3$), 25.8 (q, C[*C*H_3]$_3$).

^{19}F NMR (471 MHz, $CDCl_3$, 300 K): δ [ppm] = -142.3 – -142.6 (m, 2F), -155.9 – -157.2 (m, 1F), -162.6 – -163.0 (m, 2F).

Chiral HPLC: t_{R1} = 16.8 min t_{R2} = 18.3 min (*Daicel* Chiralcel OD-RH, 150×4.6 mm, MeCN/H_2O = 20/80 → 100/0, 1 mL/min, λ = 215 nm).

NMR data matched those previously reported in the literature.[7]

(*R*)-5,5-Dimethyl-2-(naphthalen-1-ylmethyl)hexa-2,3-dienoic acid (1q)

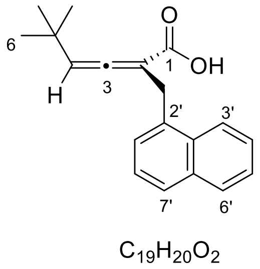

$C_{19}H_{20}O_2$
280.37 g/mol

Synthesis of the respective Ethyl Allenoate:

According to **GP1** 1-(bromomethyl)naphthalene (1.56 g, 1.08 mL, 7.04 mmol, 1.27 eq.) was reacted with (carbethoxymethylene)triphenylphosphorane (1.93 g, 5.54 mmol, 1.00 eq.) to obtain the benzylated phosphonium salt. The salt was further converted with NEt_3 (1.23 g, 1.70 mL, 12.2 mmol, 2.20 eq.) and 3,3-dimethylbutanoyl chloride (746 mg, 799 µL, 5.54 mmol, 1.00 eq.) to afford after flash column chromatography the desired ethyl allenoate (1.33 g, 4.31 mmol, 78%) as a yellow oil. The compound was used without further characterization in the next step.

*Synthesis of rac-**1q**:*

According to **GP2** the respective ethyl allenoate (1.27 g, 4.12 mmol, 1.00 eq.) was dissolved in EtOH (4 mL) and water (4 mL). NaOH (181 mg, 4.53 mmol, 1.10 eq.) was added, and the reaction mixture was heated at 100 °C for three hours. After cooling to room temperature 4 N HCl_{aq} was added until an acidic pH (pH = 1-2) was reached. The reaction mixture was extracted with EtOAc (3 × 15 mL), the combined organic phases were dried over Na_2SO_4, filtered and the solvent was removed under reduced pressure. The obtained crude acid was purified by flash column chromatography (silica, P/Et_2O = 90/10 → 80/20) to obtain allene carboxylic acid *rac*-**1q** (509 mg, 1.82 mmol, 44%) as a colorless solid.

Photochemical Deracemization of Allene Carboxylic Acid:

According to **GP4** allene carboxylic acid *rac*-**1q** (14.0 mg, 50.0 µmol, 1.00 eq.) was reacted with phosphoric acid catalyst **1q** (3.67 mg, 5.00 µmol, 10 mol%). After removing the solvent under reduced pressure, the crude product was purified by flash column chromatography to obtain the enantioenriched allene carboxylic acid **1q** (12.4 mg, 44.2 µmol, 88%, e.r. = 55/45) as a colorless oil.

TLC: R_f = 0.35 (Hex/EtOAc = 70/30) [UV/$KMnO_4$].

M.p.: 136 °C.

^{1}H NMR (400 MHz, $CDCl_3$, 300 K): δ [ppm] = 8.02 – 7.95 (m, 1H, C_{Ar}-H), 7.86 – 7.81 (m, 1H, C_{Ar}-H), 7.76 – 7.66 (m, 1H, C_{Ar}-H), 7.52 – 7.43 (m, 2H, C_{Ar}-H), 7.42 – 7.34 (m, 2H, C_{Ar}-H), 5.23 (*virt.* t, $^5J \approx {}^5J$ = 3.2 Hz, 1H, C4-H), 4.08 (dd, ^2J = 16.0 Hz, ^5J = 3.2 Hz, 1H, C1'-H^a), 4.01 (dd, ^2J = 16.0 Hz, ^5J = 3.2 Hz, 1H, C1'-H^b), 0.67 (s, 9H, $C[CH_3]_3$).

^{13}C NMR (101 MHz, $CDCl_3$, 300 K): δ [ppm] = 209.9 (s, C3), 172.7 (s, C1), 135.3 (s, C_{Ar}), 134.0 (s, C_{Ar}), 132.2 (s, C_{Ar}), 128.7 (d, C_{Ar}), 127.4 (d, C_{Ar}), 127.4 (d, C_{Ar}), 126.0 (d, C_{Ar}), 125.6

(d, C_{Ar}), 125.5 (d, C_{Ar}), 124.6 (d, C_{Ar}), 108.2 (d, C4), 101.8 (s, C2), 32.9 (s, *C*[CH_3]$_3$), 32.0 (t, C2'), 29.6 (q, C[*C*H_3]$_3$).

IR (ATR): $\tilde{\nu}$ (cm^{-1}) = 2961 (w), 1953 (w), 1666 (s), 1285 (m), 775 (s), 788 (s).

HRMS (ESI): calc. for [M + H^+]: 281.1537; found: 281.1532.

Chiral HPLC: t_{R1} = 6.6 min t_{R2} = 9.3 min (*Daicel* Chiralpak AD-H, 250×4.6 mm, n-Hep/iso-PrOH = 90/10, 1 mL/min, λ = 210 nm).

2-Benzylpenta-2,3-dienoic acid (*rac*-1r)

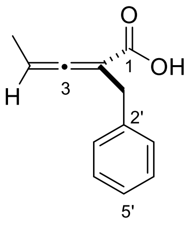

$C_{12}H_{12}O_2$
188.23 g/mol

Synthesis of the respective Ethyl Allenoate:

According to **GP1** benzyl bromide (1.87 g, 1.30 mL, 10.9 mmol, 1.27 eq.) was reacted with (carbethoxymethylene)triphenylphosphorane (3.00 g, 8.61 mmol, 1.00 eq.) to obtain the benzylated phosphonium salt. The salt was further converted with NEt_3 (1.92 g, 2.64 mL, 18.9 mmol, 2.20 eq.) and propionyl chloride (797 mg, 822 µL, 8.61 mmol, 1.00 eq.) to afford after flash column chromatography the desired ethyl allenoate (1.32 g, 6.11 mmol, 71%) as a colorless oil. The compound was used without further characterization in the next step.

*Synthesis of rac-**1r**:*

According to **GP2** the respective ethyl allenoate (1.20 g, 5.55 mmol, 1.00 eq.) was dissolved in EtOH (5 mL) and water (5 mL). NaOH (244 mg, 6.10 mmol, 1.10 eq.) was added, and the reaction mixture was heated at 100 °C for three hours. After cooling to room temperature 4 N HCl_{aq} was added until an acidic pH (pH = 1-2) was reached. The reaction mixture was extracted with EtOAc (3 × 15 mL), the combined organic phases were dried over Na_2SO_4, filtered and the solvent was removed under reduced pressure. The obtained crude acid was purified by flash column chromatography (silica, P/Et_2O = 95/5 → 75/25) to obtain allene carboxylic acid *rac*-**1r** (194 mg, 1.04 mmol, 19%) as a colorless solid.

TLC: R_f = 0.46 (Hex/EtOAc = 50/50) [UV/$KMnO_4$].

M.p.: 92 °C.

^{1}H NMR (400 MHz, $CDCl_3$, 300 K): δ [ppm] = 7.30 – 7.25 (m, 2H, C4'-H), 7.24 – 7.17 (m, 3H, C3'-H, C5'-H), 5.58 – 5.46 (m, 1H, C4-H), 3.56 (dd, ^2J = 15.0 Hz, ^5J = 2.4 Hz, 1H, C1'-H^a), 3.50 (dd, ^2J = 15.0 Hz, ^5J = 2.4 Hz, 1H, C1'-H^b), 1.72 (d, ^3J = 7.3 Hz, 3H, CH_3).

^{13}C NMR (101 MHz, $CDCl_3$, 300 K): δ [ppm] = 212.6 (s, C3), 172.6 (s, C1), 139.3 (s, C2'), 129.0 (d, C3'-H), 128.4 (d, C4'), 126.4 (d, C5'), 99.8 (s, C2), 90.7 (d, C4), 35.1 (t, C1'), 13.0 (q, CH_3).

IR (ATR): ṽ (cm^{-1}) = 2905 (w), 1956 (w), 1670 (s), 1284 (m), 1069 (w), 910 (m), 699 (s).

HRMS (ESI): calc. for [M + H^+]: 189.0911; found: 189.0908.

Methyl (*R*)-2-benzylpenta-2,3-dienoate (2r)

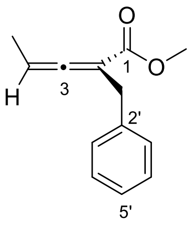

$C_{13}H_{14}O_2$
202.25 g/mol

*Synthesis of rac-**2r**:*

According to **GP3** allene carboxylic acid *rac*-**1r** (37.7 mg, 200 µmol, 1.00 eq.) was reacted with trimethylsilyldiazomethane (2.1 M in hexane, 190 µL, 400 µmol, 2.00 eq.) to afford the methyl ester *rac*-**2r** (38.6 mg, 196 µmol, 98%) as a colorless oil.

Photochemical Deracemization of Allene Carboxylic Acid:

According to **GP4** allene carboxylic acid *rac*-**1r** (9.4 mg, 50.0 µmol, 1.00 eq.) was reacted with phosphoric acid catalyst **3f** (3.67 mg, 5.00 µmol, 10 mol%). After removing the solvent under reduced pressure, the crude product was subjected to the esterification conditions described in **GP3**. After column chromatography, enantioenriched methyl ester **2r** (7.6 mg, 37.6 µmol, 75%, e.r. = 65/35) was isolated as a colorless oil.

TLC: R_f = 0.60 (Hex/EtOAc = 80/20) [UV/$KMnO_4$].

^{1}H NMR (400 MHz, $CDCl_3$, 300 K): δ [ppm] = 7.27 – 7.22 (m, 2H, C4'-H), 7.21 – 7.13 (m, 3H, C3'-H, C5'-H), 5.47 – 5.36 (m, 1H, C4-H), 3.69 (s, 3H, CO_2CH_3), 3.55 (dd, ^2J = 15.0 Hz,

^{5}J = 2.4 Hz, 1H, C1'-H[a]), 3.50 (dd, ^{2}J = 15.0 Hz, ^{5}J = 2.4 Hz, 1H, C1'-H[b]), 1.66 (d, ^{3}J = 7.3 Hz, 3H, C4-CH_3).

^{13}C NMR (101 MHz, $CDCl_3$, 300 K): δ [ppm] = 211.4 (s, C3), 167.8 (s, *C*O_2CH_3), 139.6 (s, C2'), 129.0 (d, C3'), 128.4 (d, C4'), 126.4 (d, C5'), 99.9 (s, C2), 90.3 (d, C4), 52.3 (q, CO_2*C*H_3), 35.5 (t, C1'), 13.2 (q, C4-CH_3).

IR (ATR): $\tilde{\nu}$ (cm^{-1}) = 2951 (w), 1959 (w), 1711 (s), 1435 (w), 1263 (m), 1085 (m), 698 (s).

HRMS (ESI): calc. for [M + H^+]: 203.1067; found: 203.1064.

Chiral HPLC: t_{R1} = 16.8 min t_{R2} = 17.3 min (*Daicel* Chiralcel OJ-RH, 150×4.6 mm, MeCN/H_2O = 20/80 → 100/0, 1 mL/min, λ = 215 nm).

(*R*)-2-Benzyl-4-cyclopentylbuta-2,3-dienoic acid (1s)

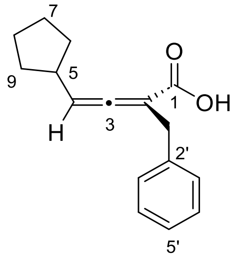

$C_{16}H_{18}O_2$
242.32 g/mol

Synthesis of the respective Ethyl Allenoate:

According to **GP1** benzyl bromide (1.25 g, 866 µL, 7.29 mmol, 1.27 eq.) was reacted with (carbethoxymethylene)triphenylphosphorane (2.00 g, 5.74 mmol, 1.00 eq.) to obtain the benzylated phosphonium salt. The salt was further converted with NEt_3 (1.28 g, 1.76 mL, 12.6 mmol, 2.20 eq.) and 2-cyclopentylacetyl chloride (841 mg, 774 µL, 5.74 mmol, 1.00 eq.) to afford after flash column chromatography the desired ethyl allenoate (783 mg, 2.90 mmol, 51%) as a colorless oil. The compound was used without further characterization in the next step.

*Synthesis of rac-**1s**:*

According to **GP2** the respective ethyl allenoate (750 mg, 2.78 mmol, 1.00 eq.) was dissolved in EtOH (3 mL) and water (3 mL). NaOH (122 mg, 3.06 mmol, 1.10 eq.) was added, and the reaction mixture was heated at 100 °C for three hours. After cooling to room temperature 4 N HCl_{aq} was added until an acidic pH (pH = 1-2) was reached. The reaction mixture was extracted

with EtOAc (3 × 15 mL), the combined organic phases were dried over Na_2SO_4, filtered and the solvent was removed under reduced pressure. The obtained crude acid was purified by flash column chromatography (silica, P/Et_2O = 90/10 → 80/20) to obtain allene carboxylic acid *rac*-**1s** (330 mg, 1.36 mmol, 49%) as a colorless oil.

Photochemical Deracemization of Allene Carboxylic Acid:

According to **GP4** allene carboxylic acid *rac*-**1s** (12.1 mg, 50.0 µmol, 1.00 eq.) was reacted with phosphoric acid catalyst **3f** (3.67 mg, 5.00 µmol, 10 mol%). After removing the solvent under reduced pressure, the crude product was purified by flash column chromatography to obtain the enantioenriched allene carboxylic acid **1s** (8.9 mg, 36.8 µmol, 74%, e.r. = 72/28) as a colorless oil.

TLC: R_f = 0.38 (Hex/EtOAc = 70/30) [UV/$KMnO_4$].

^{1}H NMR (400 MHz, $CDCl_3$, 300 K): δ [ppm] = 7.31 – 7.24 (m, 2H, C4'-H), 7.23 – 7.16 (m, 3H, C3'-H, C5'-H), 5.58 (*virt.* dt, ^3J = 6.5 Hz, $^5J \approx {}^5J$ = 2.5 Hz, 1H, C4-H), 3.57 (dd, ^2J = 15.1 Hz, ^5J = 2.5 Hz, 1H, C1'-H^a), 3.52 (dd, ^2J = 15.1 Hz, ^5J = 2.5 Hz, 1H, C1'-H^b), 2.49 (h,^3J = 7.0 Hz, 1H, C7-H), 1.81 – 1.66 (m, 2H, C6/C9-H), 1.63 – 1.39 (m, 4H, C7-H, C8-H), 1.33 – 1.13 (m, 2H, C9/C6-H).

^{13}C NMR (101 MHz, $CDCl_3$, 300 K): δ [ppm] = 211.0 (s, C3), 172.1 (s, C1), 139.3 (s, C2'), 129.1 (d, C3'), 128.4 (d, C4'), 126.4 (d, C5'), 101.0 (d, C4-H), 101.0 (s, C2), 38.6 (d, C5), 35.1 (t, C1'), 32.7 (t, C6/C9), 32.6 (t, C9/C6), 25.0 (t, C7/C8), 24.9 (t, C8/C7).

IR (ATR): ṽ (cm^{-1}) = 2953 (m), 1952 (w), 1675 (s), 1420 (m), 1277 (m), 697 (s).

HRMS (ESI): calc. for [M + H^+]: 243.1380; found: 243.1381.

Chiral HPLC: t_{R1} = 5.7 min t_{R2} = 7.7 min (*Daicel* Chiralpak AD-H, 250×4.6 mm, n-Hep/iso-PrOH = 90/10, 1 mL/min, λ = 210 nm).

(*R*)-2-Benzyl-5-methylhexa-2,3-dienoic acid (1t)

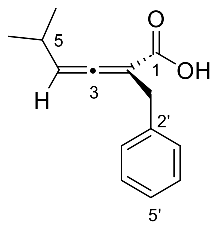

$C_{14}H_{16}O_2$
216.28 g/mol

Synthesis of the respective Ethyl Allenoate:

According to **GP1** benzyl bromide (1.25 g, 866 µL, 7.29 mmol, 1.27 eq.) was reacted with (carbethoxymethylene)triphenylphosphorane (2.00 g, 5.74 mmol, 1.00 eq.) to obtain the benzylated phosphonium salt. The salt was further converted with NEt_3 (1.28 g, 1.76 mL, 12.6 mmol, 2.20 eq.) and isovaleryl chloride (692 mg, 700 µL, 5.74 mmol, 1.00 eq.) to afford after flash column chromatography the desired ethyl allenoate (927 mg, 3.80 mmol, 66%) as a yellow oil. The compound was used without further characterization in the next step.

*Synthesis of rac-**1t**:*

According to **GP2** the respective ethyl allenoate (917 mg, 3.75 mmol, 1.00 eq.) was dissolved in EtOH (4 mL) and water (4 mL). NaOH (165 mg, 4.13 mmol, 1.10 eq.) was added, and the reaction mixture was heated at 100 °C for three hours. After cooling to room temperature 4 N HCl_{aq} was added until an acidic pH (pH = 1-2) was reached. The reaction mixture was extracted with EtOAc (3 × 15 mL), the combined organic phases were dried over Na_2SO_4, filtered and the solvent was removed under reduced pressure. The obtained crude acid was purified by flash column chromatography (silica, P/Et_2O = 90/10 → 70/30) to obtain allene carboxylic acid *rac*-**1t** (269 mg, 1.23 mmol, 30%) as a colorless solid.

Photochemical Deracemization of Allene Carboxylic Acid:

According to **GP4** allene carboxylic acid *rac*-**1t** (10.8 mg, 50.0 µmol, 1.00 eq.) was reacted with phosphoric acid catalyst **3f** (3.67 mg, 5.00 µmol, 10 mol%). After removing the solvent under reduced pressure, the crude product was purified by flash column chromatography to obtain the enantioenriched allene carboxylic acid **1t** (8.3 mg, 38.4 µmol, 77%, e.r. = 78/22) as a colorless solid.

TLC: R_f = 0.43 (Hex/EtOAc = 70/30) [UV/$KMnO_4$].

M.p.: 48 °C.

^{1}H NMR (400 MHz, $CDCl_3$, 300 K): δ [ppm] = 7.31 – 7.24 (m, 2H, C4'-H), 7.23 – 7.15 (m, 3H, C3'-H, C5'-H), 5.54 (*virt.* dt, ^3J = 6.1 Hz, $^5J \approx {}^5J$ = 2.6 Hz, 1H, C4-H), 3.58 (dd, ^2J = 15.1 Hz, ^5J = 2.6 Hz, 1H, C1'-H^a), 3.52 (dd, ^2J = 15.1 Hz, ^5J = 2.6 Hz, 1H, C1'-H^b), 2.42 – 2.28 (m, 1H, C5-H), 0.94 (dd, ^3J = 6.8 Hz, ^4J = 2.3 Hz, 6H, CH_3).

^{13}C NMR (101 MHz, $CDCl_3$, 300 K): δ [ppm] = 210.6 (s, C3), 171.6 (s, C1), 139.3 (s, C2'), 129.2 (d, C3'), 128.4 (d, C4'), 126.5 (d, C5'), 103.4 (d, C4), 101.5 (s, C2), 35.1 (t, C1'), 28.2 (d, C5), 22.4 (CH_3), 22.1 (CH_3).

IR (ATR): ṽ (cm^{-1}) = 2967 (w), 1953 (w), 1671(s), 1278 (s), 907 (w), 698 (s).

HRMS (ESI): calc. for [M + H^+]: 217.1224; found: 217.1222.

Chiral HPLC: t_{R1} = 5.6 min t_{R2} = 6.7 min (*Daicel* Chiralpak AD-H, 250×4.6 mm, n-Hep/iso-PrOH = 90/10, 1 mL/min, λ = 210 nm).

4-(Adamantan-1-yl)-2-benzylbuta-2,3-dienoic acid (*rac*-1u)

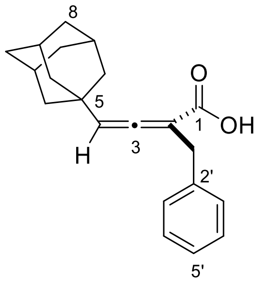

$C_{21}H_{24}O_2$
308.42 g/mol

Synthesis of the respective Ethyl Allenoate:

According to **GP1** benzyl bromide (1.20 g, 836 µL, 7.04mmol, 1.27 eq.) was reacted with (carbethoxymethylene)triphenylphosphorane (1.93 g, 5.54 mmol, 1.00 eq.) to obtain the benzylated phosphonium salt. The salt was further converted with NEt_3 (1.23 g, 1.70 mL, 12.2 mmol, 2.20 eq.) and adamantan-1-ylacetyl chloride (prepared from the corresponding acid, 1.08 g, 5.54 mmol, 1.00 eq.) to afford after flash column chromatography the desired ethyl allenoate (1.02 g, 3.04 mmol, 55%) as a colorless oil. The compound was used without further characterization in the next step.

*Synthesis of rac-**1u**:*

According to **GP2** the respective ethyl allenoate (953 mg, 2.83 mmol, 1.00 eq.) was dissolved in EtOH (3 mL) and water (3 mL). NaOH (125 mg, 3.12 mmol, 1.10 eq.) was added, and the reaction mixture was heated at 100 °C for three hours. After cooling to room temperature 4 N HCl_{aq} was added until an acidic pH (pH = 1-2) was reached. The reaction mixture was extracted with EtOAc (3 × 15 mL), the combined organic phases were dried over Na_2SO_4, filtered and the solvent was removed under reduced pressure. The obtained crude acid was purified by flash column chromatography (silica, P/Et_2O = 90/10 → 80/20) to obtain allene carboxylic acid *rac*-**1u** (340 mg, 1.10 mmol, 39%) as a colorless solid.

TLC: R_f = 0.38 (Hex/EtOAc = 70/30) [UV/$KMnO_4$].

M.p.: 141 °C.

^{1}H NMR (400 MHz, $CDCl_3$, 300 K): δ [ppm] = 7.31 – 7.24 (m, 2H, C4'-H), 7.24 – 7.14 (m, 3H, C3'-H, C5'-H), 5.37 (*virt.* t, $^5J \approx {}^5J = 2.6$ Hz, 1H, C4-H), 3.58 (dd, $^2J = 15.1$ Hz, $^5J = 2.6$ Hz, 1H, C1'-H^a), 3.52 (dd, $^2J = 15.1$ Hz, $^5J = 2.6$ Hz, 1H, C1'-H^b), 1.99 – 1.86 (m, 3H, C7-H), 1.73 – 1.63 (m, 3H, C8-H^a), 1.64 – 1.57 (m, 3H, C8-H^b), 1.56 – 1.42 (m, 6H, C6-H).

^{13}C NMR (101 MHz, $CDCl_3$, 300 K): δ [ppm] = 210.6 (s, C3), 172.4 (s, C1), 139.3 (s, C2'), 129.3 (d, C3'), 128.4 (d, C4'), 126.4 (d, C5'), 107.4 (d, C4), 101.9 (s, C2), 42.6 (t, C6), 36.7 (t, C8), 35.3 (s, C5), 35.2 (t, C1'), 28.7 (d, C7).

IR (ATR): ṽ (cm^{-1}) = 2900 (m), 2848 (m), 1952 (w), 1671(s), 1283 (s), 697 (s).

HRMS (ESI): calc. for [M + H$^+$]: 309.1850; found: 309.1849.

Methyl (*R*)-4-(adamantan-1-yl)-2-benzylbuta-2,3-dienoate (2u)

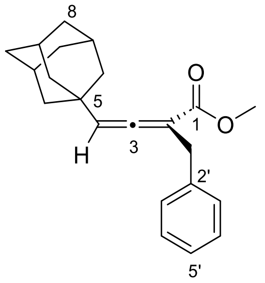

$C_{22}H_{26}O_2$
322.45 g/mol

*Synthesis of rac-**2u**:*

According to **GP3** allene carboxylic acid *rac*-**1u** (30.8 mg, 100 µmol, 1.00 eq.) was reacted with trimethylsilyldiazomethane (2.1 M in hexane, 95.2 µL, 200 µmol, 2.00 eq.) to afford the methyl ester *rac*-**2u** (32.2 mg, 100 µmol, *quant.*) as a colorless oil.

Photochemical Deracemization of Allene Carboxylic Acid:

According to **GP4** allene carboxylic acid *rac*-**1u** (15.4 mg, 50.0 µmol, 1.00 eq.) was reacted with phosphoric acid catalyst **3f** (3.67 mg, 5.00 µmol, 10 mol%). After removing the solvent under reduced pressure, the crude product was subjected to the esterification conditions described in **GP3**. After column chromatography, enantioenriched methyl ester **2u** (14.5 mg, 45.0 µmol, 90%, e.r. = 81/19) was isolated as a colorless oil.

TLC: R_f = 0.65 (Hex/EtOAc = 80/20) [UV/$KMnO_4$].

^{1}H NMR (400 MHz, $CDCl_3$, 300 K): δ [ppm] = 7.28 – 7.22 (m, 2H, C4'-H), 7.21 – 7.12 (m, 3H, C3'-H, C5'-H), 5.27 (*virt.* t, $^{5}J \approx {}^{5}J$ = 2.7 Hz, 1H, C4-H), 3.70 (q, 3H, CO_2CH_3), 3.56 (dd, ^{2}J = 15.1 Hz, ^{5}J = 2.7 Hz, 1H, C1'-H^a), 3.50 (dd, ^{2}J = 15.1 Hz, ^{5}J = 2.7 Hz, 1H, C1'-H^b), 1.95 – 1.83 (m, 3H, C7-H), 1.69 – 1.61 (m, 3H, C8-H^a), 1.59 – 1.53 (m, 3H, C8-H^b), 1.52 – 1.39 (m, 6H, C6-H).

^{13}C NMR (101 MHz, $CDCl_3$, 300 K): δ [ppm] = 209.4 (s, C3), 168.1 (s, C1), 139.5 (s, C2'), 129.4 (d, C3'), 128.3 (d, C4'), 126.3 (d, C5'), 107.0 (d, C4), 102.2 (s, C2), 52.3 (q, $CO_2\mathit{C}H_3$), 42.6 (t, C6), 36.7 (t, C8), 35.7 (t, C1'), 35.1 (s, C5), 28.7 (d, C7).

IR (ATR): ṽ (cm^{-1}) = 2902 (m), 2847 (m), 1955 (w), 1710 (s), 1435 (w), 1259 (s), 699 (s).

HRMS (ESI): calc. for [M + H^+]: 323.2006; found: 323.2006.

Chiral HPLC: t_{R1} = 23.8 min t_{R2} = 26.4 min (*Daicel* Chiralcel OJ-RH, 150×4.6 mm, MeCN/H_2O = 20/80 → 100/0, 1 mL/min, λ = 215 nm).

(*R*)-5,5-Dimethylhexa-2,3-dienoic acid (1v)

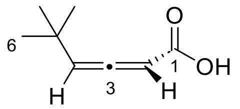

$C_8H_{12}O_2$
140.18 g/mol

Synthesis of the respective Ethyl Allenoate:

According to **GP1** carbethoxymethylene)triphenylphosphorane (3.86 g, 11.1 mmol, 1.00 eq.) was converted with NEt_3 (1.35 g, 1.84 mL, 13.3 mmol, 1.20 eq.) and 3,3-dimethylbutanoyl chloride (1.49 g, 1.54 mL, 11.1 mmol, 1.00 eq.) to afford after flash column chromatography the desired ethyl allenoate (1.19 g, 7.09 mmol, 64%) as a colorless oil. The compound was used without further characterization in the next step.

*Synthesis of rac-**1v**:*

According to **GP2** the respective ethyl allenoate (1.19 g, 7.05 mmol, 1.00 eq.) was dissolved in EtOH (7 mL) and water (7 mL). NaOH (310 mg, 7.75 mmol, 1.10 eq.) was added, and the reaction mixture was heated at 100 °C for three hours. After cooling to room temperature 4 N HCl_{aq} was added until an acidic pH (pH = 1-2) was reached. The reaction mixture was extracted with EtOAc (3 × 15 mL), the combined organic phases were dried over Na_2SO_4, filtered and the solvent was removed under reduced pressure. The obtained crude acid was purified by flash

column chromatography (silica, P/Et_2O = 85/15) to obtain allene carboxylic acid *rac*-**1v** (68.8 mg, 491 µmol, 7%) as a colorless solid.

Photochemical Deracemization of Allene Carboxylic Acid:

According to **GP4** allene carboxylic acid *rac*-**1v** (7.00 mg, 50.0 µmol, 1.00 eq.) was reacted with phosphoric acid catalyst **3f** (3.67 mg, 5.00 µmol, 10 mol%). After removing the solvent under reduced pressure, the crude product was purified by flash column chromatography to obtain the enantioenriched allene carboxylic acid **1v** (4.0 mg, 28.6 µmol, 57%, e.r. = 64/36) as a colorless solid.

TLC: R_f = 0.29 (Hex/EtOAc = 70/30) [UV/$KMnO_4$].

M.p.: 43 °C.

^{1}H NMR (400 MHz, $CDCl_3$, 300 K): δ [ppm] = 5.66 (d, ^4J = 6.0 Hz, 1H, C4-H), 5.63 (d, ^4J = 6.0 Hz, 1H, C2-H), 1.13 (s, 9H, C[CH_3]$_3$).

^{13}C NMR (101 MHz, $CDCl_3$, 300 K): δ [ppm] = 211.9 (s, C3), 170.8 (s, C1), 107.2 (d, C4), 89.2 (d, C2), 33.0 (s, *C*[CH_3]$_3$), 30.1 (q, C[*C*H_3]$_3$).

IR (ATR): ṽ (cm^{-1}) = 2963 (m), 1956 (m), 1685(s), 1285 (s), 918 (w), 703 (w).

HRMS (ESI): calc. for [M + H^+]: 141.0911; found: 141.0908.

Chiral GLC: t_{R1} = 39.8 min t_{R2} = 40.3 min [Cyclosil-B, 60 °C (0.5 min), 250 °C (2 °C/min), 250 °C (2 min)].

(*R*)-2,5,5-Trimethylhexa-2,3-dienoic acid (1w)

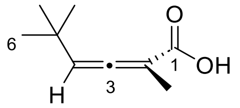

$C_9H_{14}O_2$
154.21 g/mol

*Synthesis of rac-**1w**:*

According to **GP2** the respective ethyl allenoate[7] (1.62 g, 8.89 mmol, 1.00 eq.) was dissolved in EtOH (9 mL) and water (9 mL). NaOH (391 mg, 9.78 mmol, 1.10 eq.) was added, and the reaction mixture was heated at 100 °C for three hours. After cooling to room temperature 4 N HCl_{aq} was added until an acidic pH (pH = 1-2) was reached. The reaction mixture was extracted with EtOAc (3 × 20 mL), the combined organic phases were dried over Na_2SO_4, filtered and

the solvent was removed under reduced pressure. The obtained crude acid was purified by flash column chromatography (silica, P/Et_2O = 85/15) to obtain allene carboxylic acid *rac*-**1w** (891 mg, 5.78 mmol, 65%) as a colorless solid.

Photochemical Deracemization of Allene Carboxylic Acid:

According to **GP4** allene carboxylic acid *rac*-**1w** (7.71 mg, 50.0 µmol, 1.00 eq.) was reacted with phosphoric acid catalyst **3f** (3.67 mg, 5.00 µmol, 10 mol%). After removing the solvent under reduced pressure, the crude product was purified by flash column chromatography to obtain the enantioenriched allene carboxylic acid **1w** (6.7 mg, 43.5 µmol, 87%, e.r. = 70/30) as a colorless solid.

TLC: R_f = 0.48 (Hex/EtOAc = 70/30) [UV/$KMnO_4$].

^{1}H NMR (400 MHz, $CDCl_3$, 300 K): δ [ppm] = 5.50 (q, ^5J = 2.9 Hz, 1H, C4-H), 1.86 (d, ^5J = 2.9 Hz, 3H, C2-CH_3), 1.10 (s, 9H, C[CH_3]$_3$).

^{13}C NMR (101 MHz, $CDCl_3$, 300 K): δ [ppm] = 209.4 (s, C3), 173.6 (s, C1), 105.9 (d, C4), 96.8 (s, C2), 33.2 (s, *C*[CH_3]$_3$), 30.2 (q, C[*C*H_3]$_3$), 15.1 (q, C2-*C*H_3).

Chiral GLC: t_{R1} = 40.4 min t_{R2} = 42.1 min [Cyclosil-B, 60 °C (0.5 min), 250 °C (2 °C/min), 250 °C (2 min)].

NMR data matched those previously reported in the literature.[7]

5,5-Dimethyl-2-(3-methylbut-2-en-1-yl)hexa-2,3-dienoic acid (*rac*-1x)

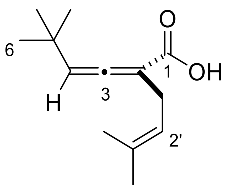

$C_{13}H_{20}O_2$
208.30 g/mol

Synthesis of the respective Ethyl Allenoate:

According to **GP1** 1-bromo-3-methylbut-2-ene (1.05 g, 813 µL, 7.04 mmol, 1.27 eq.) was reacted with (carbethoxymethylene)triphenylphosphorane (1.93 g, 5.54 mmol, 1.00 eq.) to obtain the allylated phosphonium salt. The salt was further converted with NEt_3 (1.23 g, 1.70 mL, 12.2 mmol, 2.20 eq.) and 3,3-dimethylbutanoyl chloride (746 mg, 799 µL, 5.54 mmol, 1.00 eq.) to afford after flash column chromatography the desired ethyl allenoate

(628 mg, 2.66 mmol, 48%) as a colorless oil. The compound was used without further characterization in the next step.

*Synthesis of rac-**1x**:*

According to **GP2** the respective ethyl allenoate (607 mg, 2.57 mmol, 1.00 eq.) was dissolved in EtOH (2 mL) and water (2 mL). NaOH (113 mg, 2.82 mmol, 1.10 eq.) was added, and the reaction mixture was heated at 100 °C for three hours. After cooling to room temperature 4 N HCl_{aq} was added until an acidic pH (pH = 1-2) was reached. The reaction mixture was extracted with EtOAc (3 × 7 mL), the combined organic phases were dried over Na_2SO_4, filtered and the solvent was removed under reduced pressure. The obtained crude acid was purified by flash column chromatography (silica, P/Et_2O = 85/15) to obtain allene carboxylic acid *rac*-**1x** (308 mg, 1.48 mmol, 58%) as an off-white solid.

TLC: R_f = 0.52 (Hex/EtOAc = 70/30) [UV/$KMnO_4$].

M.p.: 42 °C.

^{1}H NMR (400 MHz, $CDCl_3$, 300 K): δ [ppm] = 5.56 (*virt.* t, $^5J \approx {}^5J$ = 3.1 Hz, 1H, C4-H), 5.21 – 5.11 (m, 1H, C2'-H), 3.00 – 2.83 (m, 2H, C1'-H), 1.70 (d, ^4J = 1.3 Hz, 3H, C3'-$CH_3{}^a$), 1.63 (d, ^4J = 1.3 Hz, 3H, C3'-$CH_3{}^b$), 1.09 (s, 9H, C[CH_3]$_3$).

^{13}C NMR (101 MHz, $CDCl_3$, 300 K): δ [ppm] = 209.3 (s, C3), 172.8 (s, C1), 133.5 (s, C3'), 120.9 (d, C2'), 107.4 (d, C4-H), 101.4 (s, C2), 33.1 (s, *C*[CH_3]$_3$), 30.1 (q, C[*C*H_3]$_3$), 27.2 (t, C1'), 25.8 (q, C3'-*C*$H_3{}^a$), 18.0 (q, C3'-*C*$H_3{}^b$).

IR (ATR): ṽ (cm^{-1}) = 2962 (m), 1956 (w), 1676 (s), 1276 (m), 1254 (m), 1068 (w), 687 (w).

HRMS (ESI): calc. for [M + H^+]: 209.1537; found: 209.1538.

(*R*)-5,5-Dimethyl-2-(3-methylbut-2-en-1-yl)hexa-2,3-dienamide (6x)

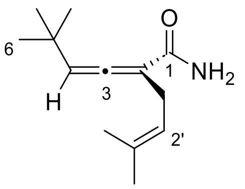

$C_{13}H_{21}NO$
207.32 g/mol

*Synthesis of rac-**6x**:*

According to a literature procedure[7] *rac*-**1x** (20.8 mg, 100 µmol, 1.00 eq.) was dissolved in CH_2Cl_2 (3.3 mL) and at 0 °C 1-hydroxybenzotriazole hydrate (18.4 mg, 120 µmol, 1.20 eq.) was added. After stirring for 10 minutes, 1-ethyl-3-(3-dimethylaminopropyl) carbodiimide (23.0 mg, 120 µmol, 1.20 eq.) was added and the obtained solution was stirred for further 30 minutes at the indicated temperature. Subsequently, a concentrated solution of ammonia in water (2.0 mL) was added and the biphasic reaction mixture was stirred overnight at room temperature. Water (10 mL) was added, the phases were separated and the aqueous phase was extracted with CH_2Cl_2 (3 × 10 mL). After washing with brine (25 mL), drying over Na_2SO_4, and filtration the solvent was removed under reduced pressure. The crude product was purified by flash column chromatography (silica, Et_2O) to obtain primary allene amide *rac*-**6x** (12.5 mg, 60.4 µmol, 60 %) as a colorless oil.

Photochemical Deracemization of Allene Carboxylic Acid:

According to **GP4** allene carboxylic acid *rac*-**1x** (10.4 mg, 50.0 µmol, 1.00 eq.) was reacted with phosphoric acid catalyst **3f** (3.67 mg, 5.00 µmol, 10 mol%). After removing the solvent under reduced pressure, the crude product was purified by flash column chromatography to obtain the enantioenriched allene carboxylic acid **1x** (8.9 mg, 42.7 µmol, 85%) as a colorless solid. For e.r. determination, **1x** was converted as described to the respective primary amide **6x** (e.r. = 84/16).

TLC: R_f = 0.43 (Hex/EtOAc = 50/50) [UV/$KMnO_4$].

^{1}H NMR (400 MHz, $CDCl_3$, 300 K): δ [ppm] = 5.87 (br s, 1H, NH), 5.57 (*virt.* t, $^5J \approx {}^5J$ = 3.3 Hz, 1H, C4-H), 5.30 (br s, 1H, NH), 5.20 – 5.13 (m, 1H, C2'-H), 3.05 – 2.85 (m, 2H, C1'-H), 1.70 (d, ^4J = 1.3 Hz, 3H, C3'-CH_3^a), 1.63 (d, ^4J = 1.3 Hz, 3H, C3'-CH_3^b), 1.09 (s, 9H, $C[CH_3]_3$).

^{13}**C NMR** (101 MHz, $CDCl_3$, 300 K): δ [ppm] = 204.0 (s, C3), 169.0 (s, C1), 133.3 (s, C3'), 121.2 (d, C2'), 108.9 (d, C4), 104.0 (s, C2), 33.2 (s, *C*[CH_3]$_3$), 30.2 (q, C[*C*H_3]$_3$), 26.5 (t, C1'), 25.8 (q, C3'-*C*H_3^a), 18.0 (q, C3'-*C*H_3^b).

IR (ATR): $\tilde{\nu}$ (cm^{-1}) = 3482 (w), 3335 (w), 3286 (w), 3208 (w), 2961 (m), 1951 (w), 1659 (s), 1592 (m), 1362 (m).

HRMS (ESI): calc. for [M + H^+]: 208.1696; found: 208.1698.

Chiral HPLC: t_{R1} = 6.3 min t_{R2} = 8.7 min (*Daicel* Chiralpak AS-H, 250×4.6 mm, n-Hep/iso-PrOH = 70/30, 1 mL/min, λ = 210 nm).

(*R*)-2-Benzyl-5,5-dimethylhexa-2,3-dienamide (6c)

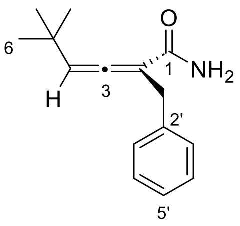

$C_{15}H_{19}NO$
229.32 g/mol

*Synthesis of rac-***6c***:*

According to a literature procedure[7] *rac*-**1c** (300 mg, 1.30 mmol, 1.00 eq.) was dissolved in CH_2Cl_2 (3.6 mL) and DCC (323 mg, 1.56 mmol, 1.20 eq.) was added at 0 °C. After stirring at 0 °C for 2.5 hours, a solution of NH_3 in THF (0.5 M, 2.61 mL, 1.31 mmol, 1.01 eq.) was added and the obtained reaction mixture was stirred at room temperature overnight. After the addition of water (7 mL), the phases were separated and the aqueous phase was extracted with CH_2Cl_2 (3 × 10 mL). Subsequently, the organic phases were washed with brine (20 mL), dried over Na_2SO_4, and filtered. The solvent was removed under reduced pressure and the obtained crude product was purified by flash column chromatography (silica, P/Et_2O = 60/40 → Et_2O) to obtain primary allene amide *rac*-**6c** (115 mg, 500 µmol, 38%) as a colorless solid.

Photochemical Deracemization of Primary Allene Amide:

In analogy to **GP4** primary allene amide *rac*-**6c** (11.5 mg, 50.0 µmol, 1.00 eq.) was reacted with phosphoric acid catalyst **3f** (3.67 mg, 5.00 µmol, 10 mol%). After removing the solvent under reduced pressure, the crude product was purified by flash column chromatography to obtain the

enantioenriched primary allene amide **6c** (10.6 mg, 46.1 μmol, 92%, e.r. = 54/46) as a colorless solid.

TLC: R_f = 0.50 (Et_2O) [UV/$KMnO_4$].

^{1}H NMR (400 MHz, $CDCl_3$, 300 K): δ [ppm] = 7.30 – 7.23 (m, 2H, C4'-H), 7.22 – 7.14 (m, 3H, C3'-H, C5'-H), 5.86 (br s, 1H, NH), 5.50 (br s, 1H, NH), 5.46 (*virt.* t, $^5J \approx {}^5J$ = 3.0 Hz, 1H, C4-H), 3.63 (dd, ^2J = 15.3 Hz, ^5J = 3.0 Hz, 1H, C1'-H^a), 3.50 (dd, ^2J = 15.3 Hz, ^5J = 3.0 Hz, 1H, C1'-H^b), 0.93 (s, 9H, C[CH_3]$_3$).

^{13}C NMR (101 MHz, $CDCl_3$, 300 K): δ [ppm] = 204.4 (s, C3), 168.7 (s, C1), 139.6 (s, C2'), 129.4 (d, C3'), 128.3 (d, C4'), 126.3 (d, C5'), 109.2 (d, C4), 104.9 (s, C2), 34.3 (t, C1'), 33.3 (s, *C*[CH_3]$_3$), 30.0 (s, C[*C*H_3]$_3$).

Chiral HPLC: t_{R1} = 7.6 min t_{R2} = 9.1 min (*Daicel* Chiralpak IA, 250×4.6 mm, n-Hep/iso-PrOH = 90/10, 1 mL/min, λ = 210 nm, 5 °C).

NMR data matched those previously reported in the literature.[7]

6. Determination of the Absolute Configuration

(*R*)-2-(4-Iodobenzyl)-5,5-dimethylhexa-2,3-dienamide (6f)

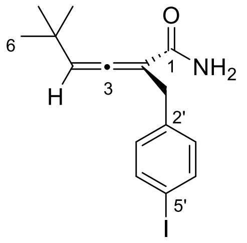

$C_{15}H_{18}INO$
355.22 g/mol

*Synthesis of rac-**6f**:*

According to a literature procedure[7] *rac*-**1f** (35.6 mg, 100 µmol, 1.00 eq.) was dissolved in CH_2Cl_2 (3.3 mL) and at 0 °C 1-hydroxybenzotriazole hydrate (18.4 mg, 120 µmol, 1.20 eq.) was added. After stirring for 10 minutes, 1-ethyl-3-(3-dimethylaminopropyl) carbodiimide (23.0 mg, 120 µmol, mmol, 1.20 eq.) was added and the obtained solution was stirred for further 30 minutes at the indicated temperature. Subsequently, a concentrated solution of ammonia in water (2.0 mL) was added and the biphasic reaction mixture was stirred overnight at room temperature. Water (5 mL) was added, the phases were separated and the aqueous phase was extracted with CH_2Cl_2 (3 × 10 mL). After washing with brine (20 mL), drying over Na_2SO_4, and filtration the solvent was removed under reduced pressure. The crude product was purified by flash column chromatography (silica, Et_2O) to obtain primary allene amide *rac*-**6f** (32.1 mg, 90.4 µmol, 90%) as a colorless solid.

Determination of the Absolute Configuration:

The described synthesis was repeated with enantioenriched material (e.r. = 80/20) and after column chromatography **6f** (29.8 mg, 83.9 µmol, 84%, e.r. = 80/20) was isolated as a colorless solid. Comparison with the HPLC traces reported in the literature[7] allowed for the assignment of the shown absolute configuration.

TLC: R_f = 0.71(EtOAc) [UV/$KMnO_4$].

^{1}H NMR (400 MHz, $CDCl_3$, 300 K): δ [ppm] = 7.64 – 7.46 (m, 2H, C4'-H), 6.99 – 6.90 (m, 2H, C3'-H), 5.85 (br s, 1H, NH), 5.50 (*virt.* t, $^5J \approx {}^5J$ = 2.9 Hz, 1H, C4-H), 5.37 (br s, 1H, NH), 3.56 (dd, ^2J = 15.2 Hz, ^5J = 2.9 Hz, 1H, C1'-H^a), 3.49 (dd, ^2J = 15.2 Hz, ^5J = 2.9 Hz, 1H, C1'-H^b), 0.96 (s, 9H, $C[CH_3]_3$).

^{13}C NMR (101 MHz, $CDCl_3$, 300 K): δ [ppm] = 204.3 (s, C3), 168.3 (s, C1), 139.4 (s, C2’), 137.4 (d, C4’), 131.5 (d, C3’), 109.4 (d, C4), 104.3 (s, C5’), 91.5 (s, C2), 33.9 (t, C1’), 33.4 (s, *C*[CH_3]$_3$), 30.1 (q, C[*C*H_3]$_3$).

Chiral HPLC: t_{R1} = 11.1 min t_{R2} = 14.3 min (*Daicel* Chiralpak AD-H, 250×4.6 mm, n-Hep/iso-PrOH = 90/10, 1 mL/min, λ = 210 nm).

*Racemic Product rac-**6f***

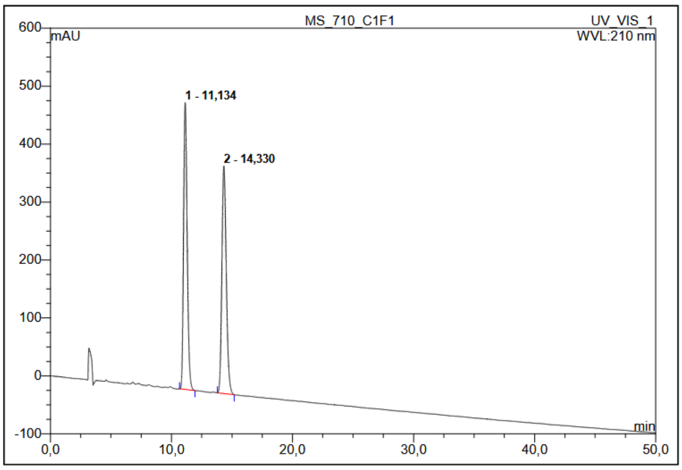

No.	Ret.Time min	Peak Name	Height mAU	Area mAU*min	Rel.Area %	Amount	Type
1	11,13	n.a.	494,865	170,578	50,62	n.a.	BMB
2	14,33	n.a.	392,774	166,419	49,38	n.a.	BMB
Total:			887,639	336,996	100,00	0,000	

*Enantioenriched Product **6f***

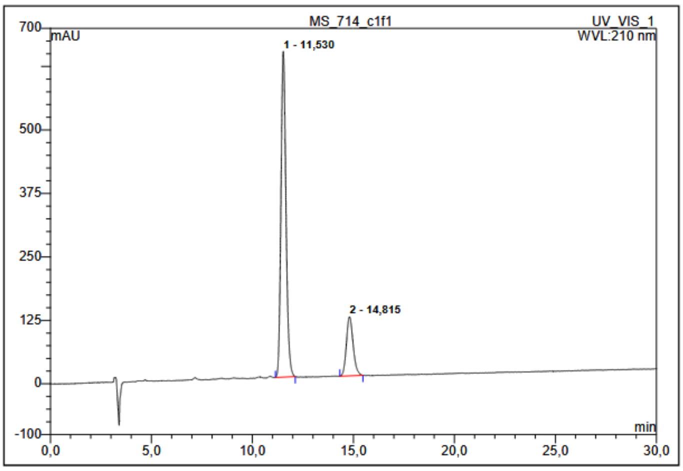

No.	Ret.Time min	Peak Name	Height mAU	Area mAU*min	Rel.Area %	Amount	Type
1	11,53	n.a.	641,478	184,733	80,47	n.a.	BMB
2	14,81	n.a.	116,146	44,830	19,53	n.a.	BMB
Total:			757,623	229,563	100,00	0,000	

7. Synthesis and Deracemization of Phenyl-Substituted Allene Carboxylic Acids

(*R*)-2-Benzyl-4-phenylbuta-2,3-dienoic acid (*rac*-SI-1)

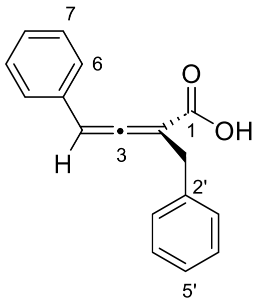

$C_{17}H_{14}O_2$
250.30 g/mol

*Synthesis of rac-**SI-1**:*

The respective ethyl allenoate[7] (770 mg, 2.77 mmol, 1.00 eq.) was dissolved in EtOH (5.4 mL), 2 M NaOH (27.7 mL, 55.3 mmol, 20.0 eq.) was added, and the reaction mixture was stirred at room temperature for four days. 4 N HCl_{aq} was added until an acidic pH (pH = 1-2) was reached. The reaction mixture was extracted with EtOAc (3 × 25 mL), the combined organic phases were dried over Na_2SO_4, filtered and the solvent was removed under reduced pressure. The obtained crude acid was purified by flash column chromatography (silica, P/Et_2O = 70/30) to obtain allene carboxylic acid *rac*-**SI-1** (333 mg, 1.33 mmol, 48%) as a colorless solid.

Photochemical Deracemization of Allene Carboxylic Acid:

According to **GP4** allene carboxylic acid *rac*-**SI-1** (12.5 mg, 50.0 µmol, 1.00 eq.) was reacted with phosphoric acid catalyst **3f** (3.67 mg, 5.00 µmol, 10 mol%). After removing the solvent under reduced pressure, the e.r. of the crude product was analyzed by chiral HPLC (e.r. = 57/43).

TLC: R_f = 0.22 (Hex/EtOAc = 70/30) [UV/$KMnO_4$].

^{1}H NMR (500 MHz, $CDCl_3$, 300 K): δ [ppm] = 7.33 – 7.28 (m, 2H, C_{Ar}-H), 7.27 – 7.16 (m, 8H, C_{Ar}-H), 6.58 (*virt.* t, $^5J \approx {}^5J$ = 2.4 Hz, 1H, C4-H), 3.69 (dd, ^2J = 14.8 Hz, ^5J = 2.4 Hz, 1H, C1'-H^a), 3.65 (dd, ^2J = 14.8 Hz, ^5J = 2.4 Hz, 1H, C1'-H^b).

^{13}C NMR (126 MHz, $CDCl_3$, 300 K): δ [ppm] = 214.5 (s, C3), 171.7 (s, C1), 138.8 (s, C2'), 131.5 (s, C5), 129.0 (d, C_{Ar}), 129.0 (d, C_{Ar}), 128.5 (d, C_{Ar}), 128.2 (d, C_{Ar}), 127.6 (d, C_{Ar}), 126.7 (d, C_{Ar}), 103.9 (s, C2), 99.1 (d, C4), 35.3 (t, C1').

Chiral HPLC: t_{R1} = 6.9 min t_{R2} = 8.1 min (*Daicel* Chiralpak AD-H, 250×4.6 mm, n-Hep/iso-PrOH = 80/20, 1 mL/min, λ = 210 nm).

NMR data matched those previously reported in the literature (*Chem. Eur. J.* 2010, *16*, 4739 – 4743, *Org. Lett.* 2024, *26*, 2430 – 2434).

(*R*)-5,5-Dimethyl-2-phenylhexa-2,3-dienoic acid (*rac*-SI-2)

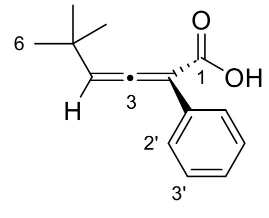

$C_{14}H_{16}O_2$
216.28 g/mol

Synthesis of the respective Ethyl Allenoate:

In analogy to **GP1** (2-ethoxy-2-oxo-1-phenylethyl)triphenylphosphonium iodide (3.65 g, 6.61 mmol, 1.00 eq.) was converted with NEt_3 (1.47 g, 2.03 mL, 14.5 mmol, 2.20 eq.) and 3,3-dimethylbutanoyl chloride (889 mg, 953 µL, 6.61 mmol, 1.00 eq.) to afford after flash column chromatography the desired ethyl allenoate (1.27 g, 5.21 mmol, 79%) as a colorless oil. The compound was used without further characterization in the next step.

*Synthesis of rac-**SI-2**:*

The respective ethyl allenoate (722 mg, 2.95 mmol, 1.00 eq.) was dissolved in EtOH (5.8 mL), 2 M NaOH (29.6 mL, 59.1 mmol, 20.0 eq.) was added, and the reaction mixture was stirred at room temperature for two days. 4 N HCl_{aq} was added until an acidic pH (pH = 1-2) was reached. The reaction mixture was extracted with EtOAc (3 × 30 mL), the combined organic phases were dried over Na_2SO_4, filtered and the solvent was removed under reduced pressure. The obtained crude acid was purified by flash column chromatography (silica, P/Et_2O = 95/5 → Et_2O) to obtain allene carboxylic acid *rac*-**SI-2** (90.1 mg, 416 µmol, 14%) as a colorless solid.

Photochemical Deracemization of Allene Carboxylic Acid:

According to **GP4** allene carboxylic acid *rac*-**SI-2** (10.8 mg, 50.0 µmol, 1.00 eq.) was reacted with phosphoric acid catalyst **3f** (3.67 mg, 5.00 µmol, 10 mol%). After removing the solvent under reduced pressure, the e.r. of the crude product was analyzed by chiral HPLC (e.r. = 58/42).

TLC: R_f = 0.38 (Hex/EtOAc = 70/30) [UV/$KMnO_4$].

M.p.: 123 °C.

^{1}H NMR (400 MHz, $CDCl_3$, 300 K): δ [ppm] = 7.56 – 7.50 (m, 2H, C2'-H), 7.39 – 7.32 (m, 2H, C3'-H), 7.30 – 7.26 (m, 1H, C4'-H), 5.85 (s, 1H, C4-H), 1.19 (s, 9H, $C[CH_3]_3$).

^{13}C NMR (101 MHz, $CDCl_3$, 300 K): δ [ppm] = 211.0 (s, C3), 171.8 (s, C1), 132.8 (s, C1'), 128.5 (d, C2'), 128.4 (d, C3'), 127.7 (d, C4'), 107.9 (d, C4), 104.0 (s, C2), 33.9 (s, $\mathit{C}[CH_3]_3$), 30.2 (q, $C[\mathit{C}H_3]_3$).

IR (ATR): ṽ (cm^{-1}) = 2963 (m), 1939 (w), 1676 (s), 1411 (m), 1292 (m), 692 (s).

HRMS (ESI): calc. for $[M + H^{+}]$: 217.1224; found: 217.1219.

Chiral HPLC: t_{R1} = 6.2 min t_{R2} = 7.6 min (*Daicel* Chiralpak AD-H, 250×4.6 mm, n-Hep/iso-PrOH = 90/10, 1 mL/min, λ = 210 nm).

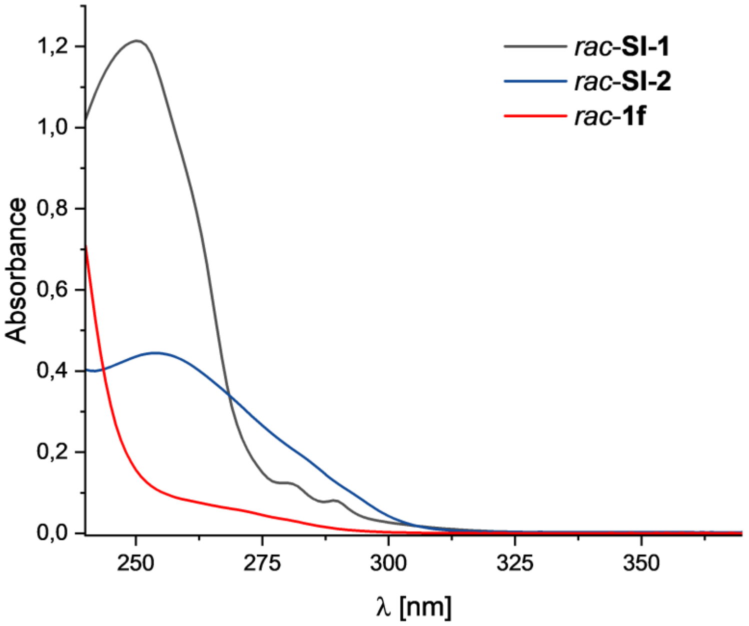

Figure S7.1. UV/VIS spectra of *rac*-**SI-1**, *rac*-**SI-2** and *rac*-**1f** (0.5 mM, dichloromethane, 1 mm cuvette). The phenyl-substituted allenoic acids show a bathochromic shift compared to *rac*-**1f**, which likely invites direct excitation.

8. Racemization Experiments and Additional UV/VIS Spectra

The experiments were performed in analogy to **GP4** on a 10 µmol scale using **1f** (e.r. = 80/20). The e.r. values were determined by HPLC analysis of the crude reaction mixtures.

*HPLC Trace of rac-**1f** with added Thioxanthone*

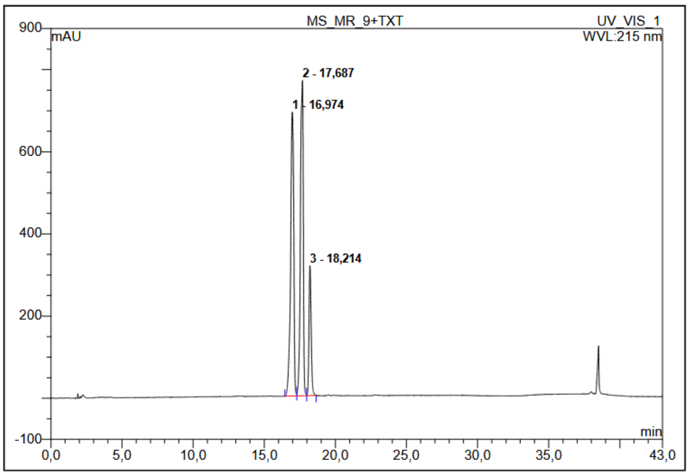

No.	Ret.Time min	Peak Name	Height mAU	Area mAU*min	Rel.Area %	Amount	Type
1	16,97	n.a.	691,070	176,227	43,06	n.a.	BM
2	17,69	n.a.	767,544	177,393	43,35	n.a.	M
3	18,21	n.a.	316,173	55,612	13,59	n.a.	MB
Total:			1774,786	409,232	100,00	0,000	

Racemization at 440 nm

A solution of **1f** (3.56 mg, 10.0 µmol, 1.00 eq.) and thioxanthone (2.12 mg, 10.0 µmol, 1.00 eq.) in dichloromethane (c = 10 mM) was cooled to −10 °C and irradiated (λ = 440 nm) for 4 h. The solvent was removed under reduced pressure and the obtained crude product was analyzed by HPLC (e.r. = 73/27).

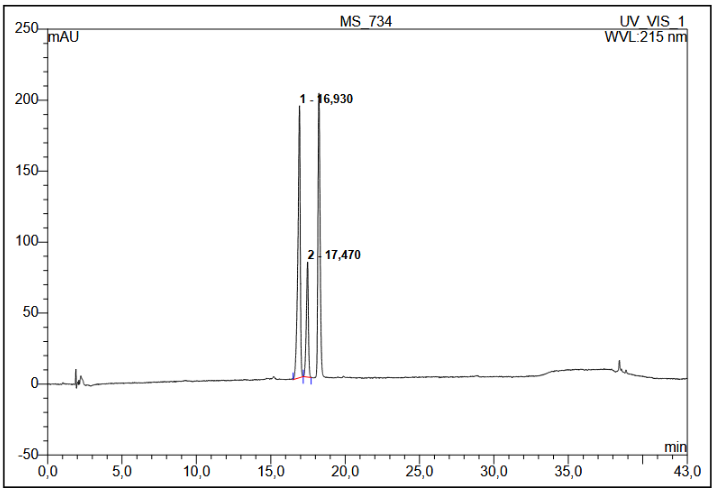

No.	Ret.Time min	Peak Name	Height mAU	Area mAU*min	Rel.Area %	Amount	Type
1	16,93	n.a.	191,636	35,043	73,27	n.a.	BMB*
2	17,47	n.a.	81,275	12,783	26,73	n.a.	BMB*
Total:			272,911	47,826	100,00	0,000	

Racemization at 420 nm

A solution of **1f** (3.56 mg, 10.0 µmol, 1.00 eq.) and thioxanthone (2.12 mg, 10.0 µmol, 1.00 eq.) in dichloromethane (c = 10 mM) was cooled to −10 °C and irradiated (λ = 420 nm) for 4 h. The solvent was removed under reduced pressure and the obtained crude product was analyzed by HPLC (e.r. = 66/34).

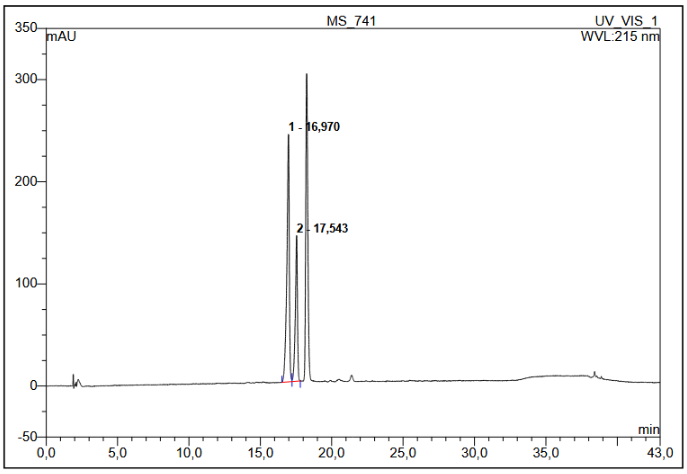

No.	Ret.Time min	Peak Name	Height mAU	Area mAU*min	Rel.Area %	Amount	Type
1	16,97	n.a.	242,526	47,790	66,11	n.a.	BM
2	17,54	n.a.	142,618	24,499	33,89	n.a.	MB
Total:			385,144	72,289	100,00	0,000	

Racemization at 420 nm without Thioxanthone

A solution of **1f** (3.56 mg, 10.0 µmol, 1.00 eq.) in dichloromethane (c = 10 mM) was cooled to −10 °C and irradiated (λ = 420 nm) for 4 h. The solvent was removed under reduced pressure and the obtained crude product was analyzed by HPLC (e.r. = 76/24).

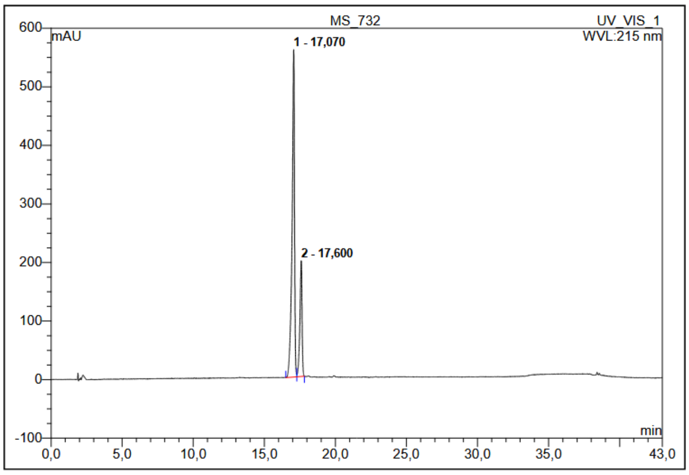

No.	Ret.Time min	Peak Name	Height mAU	Area mAU*min	Rel.Area %	Amount	Type
1	17,07	n.a.	559,188	109,539	76,35	n.a.	BM
2	17,60	n.a.	197,755	33,925	23,65	n.a.	MB
Total:			756,943	143,464	100,00	0,000	

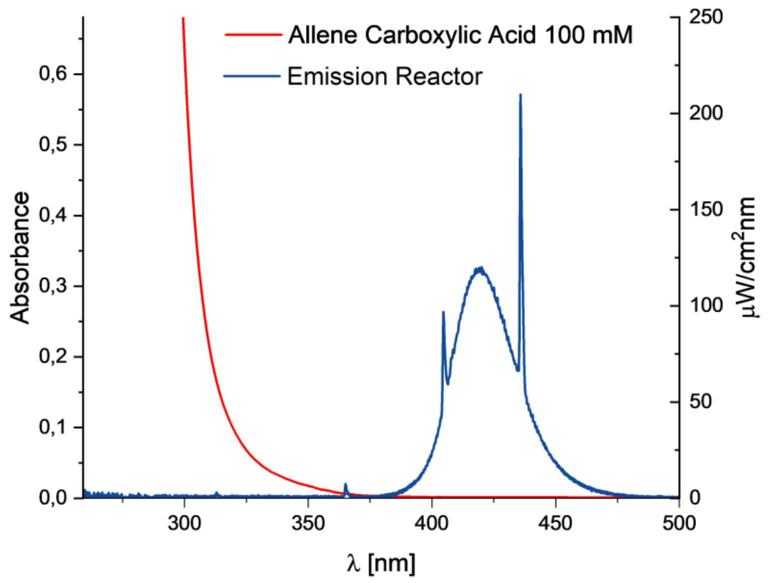

Figure S8.1. UV/VIS spectrum of allene carboxylic acid *rac*-**1f** (100 mM, dichloromethane, 1 mm cuvette) and emission of the employed 420 nm photoreactor.

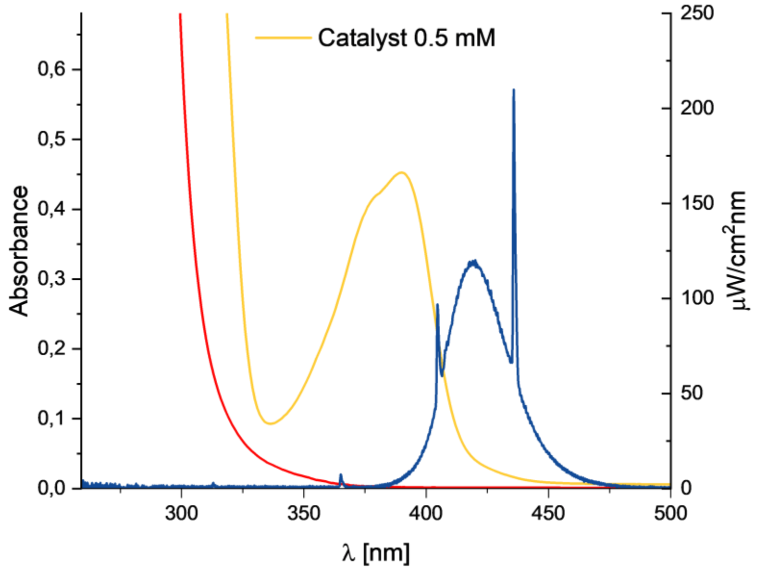

Figure S8.2. UV/VIS spectrum of allene carboxylic acid *rac*-**1f** (100 mM, dichloromethane, 1 mm cuvette) and emission of the employed 420 nm photoreactor. In yellow, UV/VIS spectrum of phosphoric acid **3f** (0.5 mM, dichloromethane, 1 mm cuvette).

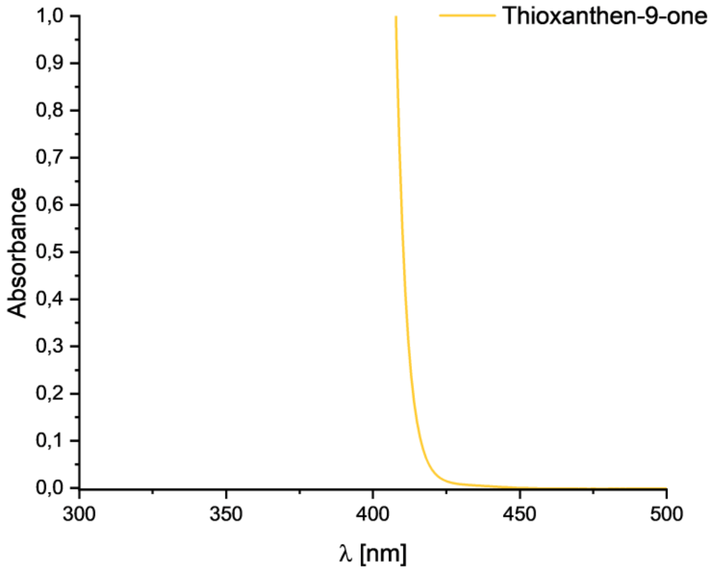

Figure S8.3. UV/VIS spectrum of thioxanthene-9-one (100 mM, dichloromethane, 1 mm cuvette).

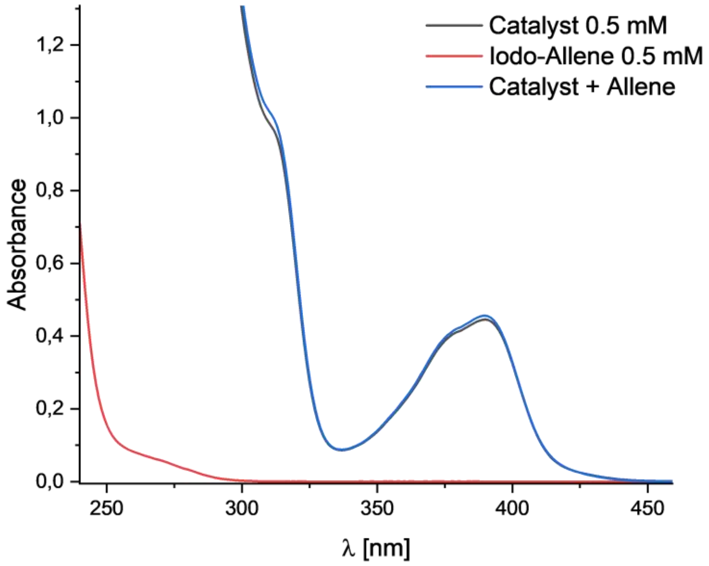

Figure S8.4. UV/VIS spectra of catalyst **3f** and *rac*-**1f** (0.5 mM, dichloromethane, 1 mm cuvette) and a mixture of both samples. There is no notable absorption shift of the allenoic acid.

9. Deracemization Reactions Employing a Dual Catalyst System

To evaluate a dual catalysis approach involving the use of external thioxanthene-9-one (**Txt**) in combination with a commercially available chiral phosphoric acid, the reactions presented in **Scheme S9.1** were performed in analogy to **GP4**.

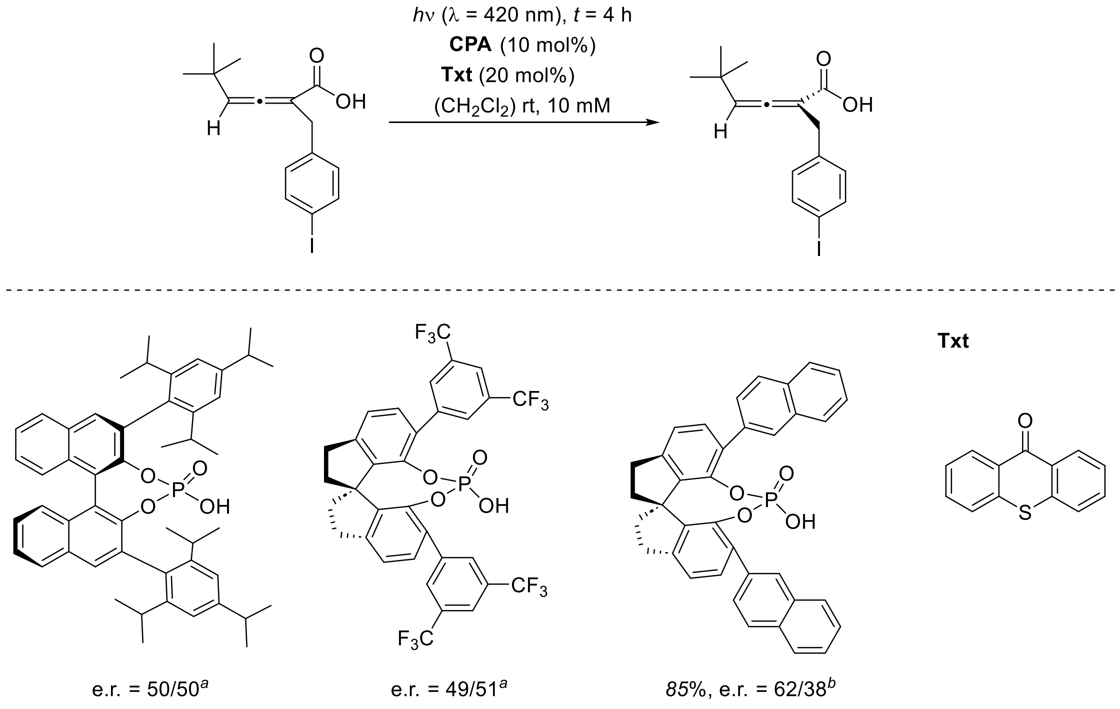

Scheme S9.1. [a] Deracemization reactions performed at room temperature on a 25 µmol scale, e.r. values determined by chiral HPLC analysis of the crude product. [b] Deracemization reaction performed on a 50 µmol scale at −10 °C, product was isolated by chromatography and the e.r. value was determined from the purified product.

10. Consecutive Reactions

(*R*)-4-Bromo-5-(*tert*-butyl)-3-(4-iodobenzyl)furan-2(5*H*)-one (8)

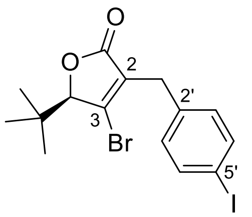

$C_{15}H_{16}BrIO_2$
435.10 g/mol

*Synthesis of rac-**8**:*

According to a literature procedure,[23] *rac*-**1f** (30.0 mg, 84.2 µmol, 1.00 eq.) was reacted in water (1.5 mL) with K_2CO_3 (11.6 mg, 84.2 µmol, 1.00 eq.) and NBS (30.0 mg, 168 µmol, 2.00 eq.) for five hours. After TLC analysis (Hex/EtOAc = 80/20) revealed complete conversion of the starting material, the reaction mixture was extracted with Et_2O (3 × 5 mL). The organic phases were dried over Na_2SO_4, filtered and the solvent was removed under reduced pressure. Purification by column chromatography (silica, P/Et_2O = 90/10) afforded *rac*-**8** (33.4 mg, 76.8 µmol, 91%) as a colorless oil.

*Synthesis of **8**:*

The described synthesis was repeated with enantioenriched material (23.5 mg, 66.0 µmol, e.r. = 80/20) and after column chromatography lactone **8** (25.8 mg, 59.3 µmol, 90%, e.r. = 81/19) was isolated as a colorless oil.

TLC: R_f = 0.47 (Hex/EtOAc = 90/10) [UV/$KMnO_4$].

^{1}H NMR (400 MHz, $CDCl_3$, 300 K): δ [ppm] = 7.75 – 7.52 (m, 2H, C4'-H), 7.17 – 6.47 (m, 2H, C3'-H), 4.88 – 4.37 (m, 1H. C4-H), 3.62 – 3.60 (m, 2H, C1'-H), 1.06 (s, 9H, $C[CH_3]_3$).

^{13}C NMR (101 MHz, $CDCl_3$, 300 K): δ [ppm] = 170.2 (s, C1), 143.0 (s, C3), 137.9 (d, C4'), 136.3 (s, C2'), 133.4 (s, C2), 130.8 (d, C3'), 92.4 (s, C5'), 90.4 (d, C4), 36.2 (s, $\mathit{C}[CH_3]_3$), 30.7 (t, C1'), 26.2 (q, $C[\mathit{C}H_3]_3$).

IR (ATR): ṽ (cm^{-1}) = 2968 (w), 1753 (s), 1643 (m), 1479 (m), 1316 (m), 1004 (s), 823 (w), 707 (m).

HRMS (ESI): calc. for [M + H^+]: 434.9452; found: 434.9432.

Chiral HPLC: t_{R1} = 6.4 min t_{R2} = 6.9 min (*Daicel* Chiralpak AD-H, 250×4.6 mm, n-Hep/iso-PrOH = 90/10, 1 mL/min, λ = 210 nm).

(*R*)-5-(*tert*-Butyl)-4-iodo-3-(4-iodobenzyl)furan-2(5*H*)-one (9)

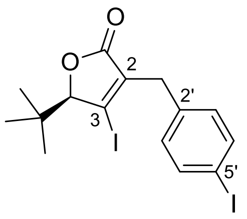

$C_{15}H_{16}I_2O_2$
482.10 g/mol

*Synthesis of rac-**9**:*

According to a literature procedure,[24] *rac*-**1f** (17.8 mg, 50.0 µmol, 1.00 eq.) was reacted in a mixture of MeCN (1.0 mL) and water (40 µL) with iodine (62.2 mg, 245 µmol, 4.90 eq.) for 14 hours. The reaction was stopped by addition of sat. $Na_2S_2O_{3aq}$ (3 mL), Et_2O was added and the layers were separated. The aqueous phase was extracted with Et_2O (3 × 5 mL) and the united organic phases were dried over Na_2SO_4. After filtration, the solvent was removed under reduced pressure and purification by column chromatography (silica, P/Et_2O = 90/10) afforded *rac*-**9** (21.0 mg, 43.6 µmol, 87%) as a colorless oil.

*Synthesis of **9**:*

The described synthesis was repeated with enantioenriched material (17.8 mg, 50.0 µmol, e.r. = 80/20) and after column chromatography lactone **7** (20.2 mg, 41.9 µmol, 84%, e.r. = 77/23) was isolated as a colorless oil.

TLC: R_f = 0.58 (Hex/EtOAc = 80/20) [UV/$KMnO_4$].

^{1}H NMR (400 MHz, $CDCl_3$, 300 K): δ [ppm] = 7.68 – 7.43 (m, 2H, C4'-H), 7.14 – 6.92 (m, 2H, C3'-H), 4.75 – 4.56 (m, 1H, C4-H), 3.69 – 3.58 (m, 2H, C1'-H), 1.09 (s, 9H, C[CH_3]$_3$).

^{13}C NMR (101 MHz, $CDCl_3$, 300 K): δ [ppm] = 169.6 (s, C1), 140.2 (s, C2), 137.8 (d, C4'), 136.4 (s, C2'), 130.9 (d, C3'), 117.7 (s, C3), 92.4 (s, C5'), 91.7 (d, C4), 36.5 (s, *C*[CH_3]$_3$), 33.3 (t, C1'), 26.7 (q, C[*C*H_3]$_3$).

IR (ATR): ṽ (cm^{-1}) = 2958 (w), 1743 (s), 1620 (w), 1482 (w), 1316 (w), 1031 (w), 1006 (m).

HRMS (ESI): calc. for [M + H^+]: 482.9313; found: 482.9289.

Chiral HPLC: t_{R1} = 21.4 min t_{R2} = 22.0 min (*Daicel* Chiralcel OJ-RH, 150×4.6 mm, MeCN/H_2O = 20/80 → 100/0, 1 mL/min, λ = 215 nm).

(*R*)-2-(4-Iodobenzyl)-5,5-dimethylhexa-2,3-dien-1-ol (10)

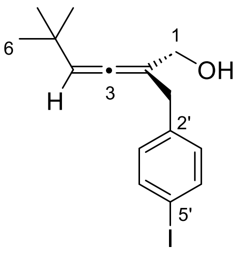

$C_{15}H_{19}IO$
342.22 g/mol

*Synthesis of rac-**10**:*

Allene carboxylic acid *rac*-**1f** (106.9 mg, 300 µmol, 1.00 eq.) was converted to the respective methyl ester (110 mg, 297 µmol, 99%) using **GP3**.

The methyl ester (40.3 mg, 109 µmol, 1.00 eq.) was dissolved in 1.2 mL PhMe and at −78 °C a solution of DIBAL-H (1 M in hexane, 229 µL, 2.10 eq.) was slowly added.[25] After 4 hours at −78 °C the reaction was stopped at this temperature by addition of ~0.5 mL MeOH. The reaction mixture was warmed to ambient temperature and 1 M HCl_{aq} (2.0 mL) was added. The phases were separated and the aqueous phase was extracted with Et_2O (3 × 5 mL) and the united organic phases were dried over Na_2SO_4. After filtration, the solvent was removed under reduced pressure and purification by column chromatography (silica, P/Et_2O = 90/10) afforded *rac*-**10** (35.4 mg, 103 µmol, 95%) as a colorless oil.

*Synthesis of **10**:*

Allene carboxylic acid **1f** (35.6 mg, 100 µmol, 1.00 eq., e.r. = 80/20) was converted to the respective methyl ester (35.0 mg, 94.6 µmol, 95%, e.r. = 80/20) using **GP3**.

The previously described reduction was repeated with the enantioenriched ester (33.8 mg, 91.3 µmol, e.r. = 80/20) and after column chromatography alcohol **10** (26.9 mg, 78.7 µmol, 86%) was isolated as a colorless oil.

Unfortunately, the enantiomers of *rac*-**10** were not separable on chiral HPLC in our hands, preventing us from reporting an e.r. value for this compound.

TLC: R_f = 0.38 (Hex/EtOAc = 80/20) [UV/$KMnO_4$].

^{1}H NMR (400 MHz, $CDCl_3$, 300 K): δ [ppm] = 7.66 – 7.50 (m, 2H, C4'-H), 7.04 – 6.84 (m, 2H, C3'-H), 5.58 – 4.73 (m, 1H, C4-H), 4.16 – 3.82 (m, 2H, C1-H), 3.35 – 3.24 (m, 2H, C1'-H), 0.95 (s, 9H, $C[CH_3]_3$).

^{13}C NMR (101 MHz, $CDCl_3$, 300 K): δ [ppm] = 197.5 (s, C3), 139.0 (s, C2'), 137.5 (d, C4'), 131.2 (d, C3'), 107.8 (d, C4), 106.7 (s, C2), 91.5 (s, C5'), 63.0 (t, C1), 36.4 (t, C1'), 32.4 (s, $\mathit{C}[CH_3]_3$), 30.2 (q, $C[\mathit{C}H_3]_3$).

IR (ATR): $\tilde{\nu}$ (cm^{-1}) = 3328 (w), 2958 (m), 1964 (w), 1484 (m), 1361 (m), 1007 (s), 809 (m).

HRMS (EI, 70 eV): calc. for $C_{15}H_{19}O^{127}I$ $[M]^+$: 342.0475; found: 342.0472.

(*R*)-2-(*tert*-Butyl)-3-iodo-4-(4-iodobenzyl)-2,5-dihydrofuran (11)

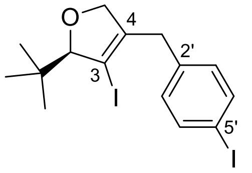

$C_{15}H_{18}I_2O$
468.12 g/mol

*Synthesis of rac-**11**:*

According to a literature procedure,[24] *rac*-**10** (34.2 mg, 100 µmol, 1.00 eq.) was reacted in a mixture of MeCN (2.0 mL) and water (80 µL) with iodine (124 mg, 490 µmol, 4.90 eq.) for 15 hours. The reaction was stopped by addition of sat. $Na_2S_2O_{3aq}$ (3 mL), Et_2O was added and the layers were separated. The aqueous phase was extracted with Et_2O (3 × 5 mL) and the united organic phases were washed with water (1 × 10 mL). After drying over Na_2SO_4 and filtration, the solvent was removed under reduced pressure and purification by column chromatography (silica, P/Et_2O = 90/10) afforded *rac*-**11** (42.5 mg, 90.8 µmol, 91%) as a slightly yellow oil.

*Synthesis of **11**:*

The synthesis was repeated with the previously obtained enantioenriched alcohol **10** (13.5 mg, 39.5 µmol) and after column chromatography ether **11** (17.6 mg, 37.6 µmol, 95%, e.r. = 75/25) was isolated as a slightly yellow oil.

TLC: R_f = 0.79 (Hex/EtOAc = 80/20) [UV/$KMnO_4$].

^{1}H NMR (400 MHz, $CDCl_3$, 300 K): δ [ppm] = 7.70 – 7.48 (m, 2H, C4'-H), 7.05 – 6.84 (m, 2H, C3'-H), 4.53 – 4.46 (m, 1H, C2-H), 4.46 – 4.38 (m, 1H, C5-H^a), 4.37 – 4.30 (m, 1H, C5-H^b), 3.61 – 3.51 (m, 1H, C1'-H^a), 3.49 – 3.31 (m, 1H, C1'-H^b), 1.05 (s, 9H, C[CH_3]$_3$).

^{13}C NMR (101 MHz, $CDCl_3$, 300 K): δ [ppm] = 145.3 (s, C4), 137.9 (d, C4'), 137.2 (s, C2'), 130.5 (d, C3'), 96.9 (d, C2), 92.0 (s, C5'), 87.2 (s, C3), 75.9 (t, C5), 37.5 (s, *C*[CH_3]$_3$), 36.1 (t, C1'), 27.0 (q, C[*C*H_3]$_3$).

IR (ATR): ṽ (cm^{-1}) = 2956 (m), 2849 (m), 1644 (w), 1482 (m), 1052 (s), 1009 (s), 811 (s).

HRMS (EI, 70 eV): calc. for $C_{15}H_{18}O^{127}I_2$ $[M]^+$: 467.9442; found: 467.9446.

Chiral HPLC: t_{R1} = 25.8 min t_{R2} = 27.2 min (*Daicel* Chiralcel OJ-RH, 150×4.6 mm, MeCN/H_2O = 20/80 → 100/0, 1 mL/min, λ = 215 nm).

(*R*)-*tert*-Butyl((2-(4-iodobenzyl)-5,5-dimethylhexa-2,3-dien-1-yl)oxy)dimethylsilane (12)

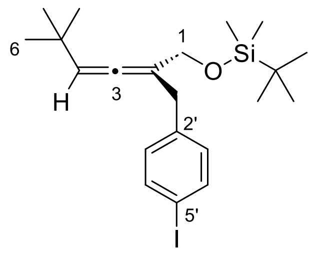

$C_{21}H_{33}IOSi$
456.48 g/mol

*Synthesis of rac-**12**:*

Alcohol *rac*-**10** (30.0 mg, 87.7 µmol, 1.00 eq.) was dissolved in CH_2Cl_2 (1.5 mL) and imidazole (8.95 mg, 131 µmol, 1.50 eq.), as well as TBSCl (17.2 mg, 114 µmol, 1.30 eq.) were added. After stirring for 16 h at room temperature, NH_4Cl_{aq} (2.0 mL) was added, the phases were separated and the aqueous phase was extracted with CH_2Cl_2 (3 × 5 mL). The organic phases were dried over Na_2SO_4, filtered and the solvent was removed under reduced pressure. Purification by column chromatography (silica, P/Et_2O = 90/10) afforded *rac*-**12** (36.6 mg, 80.2 µmol, 92%) as a colorless oil.

*Synthesis of **12**:*

The synthesis was repeated with the previously obtained enantioenriched alcohol **10** (13.5 mg, 39.5 µmol) and after column chromatography protected alcohol **12** (15.6 mg, 34.2 µmol, 87%, e.r. = 75/25) was isolated as a colorless oil.

TLC: R_f = 0.75 (Hex/EtOAc = 90/10) [UV/$KMnO_4$].

^{1}H NMR (400 MHz, $CDCl_3$, 300 K): δ [ppm] = 7.63 – 7.45 (m, 2H, C4'-H), 7.04 – 6.84 (m, 2H, C3'-H), 5.10 (*virt.* p, $^5J \approx {}^5J$ = 2.6 Hz, 1H, C4-H), 4.07 (d, ^5J = 2.6 Hz, 2H, C1-H), 3.29 (d, ^5J = 2.6 Hz, 2H, C1'-H), 0.92 (s, 9H, C5[CH_3]$_3$), 0.90 (s, SiC[CH_3]$_3$), 0.04 (s, 6H, $SiCH_3$).

^{13}C NMR (101 MHz, $CDCl_3$, 300 K): δ [ppm] = 198.8 (s, C3), 139.6 (s, C2'), 137.2 (d, C4'), 131.5 (d, C3'), 105.6 (s, C2), 105.0 (d, C4), 91.2 (s, C5'), 64.1 (t, C1), 36.0 (t, C1'), 32.3 (s, *C*5[CH_3]$_3$), 30.2 (q, C5[*C*H_3]$_3$), 26.1 (q, SiC[*C*H_3]$_3$), 18.5 (s, Si*C*[CH_3]$_3$), -5.1 (q, $SiCH_3^a$), -5.2 (q, $SiCH_3^b$).

IR (ATR): ṽ (cm^{-1}) = 2956 (m), 1966 (w), 1462 (m), 1472 (m), 1253 (m), 1068 (s), 835 (s), 773 (s).

HRMS (EI, 70 eV): calc. for $C_{21}H_{33}O^{127}I^{28}Si$ $[M]^+$: 456.1340; found: 456.1344.

Chiral HPLC: t_{R1} = 26.1 min t_{R2} = 30.8 min (*Daicel* Chiralcel OJ-RH, 150×4.6 mm, MeCN/H_2O = 20/80 → 100/0, 1 mL/min, λ = 215 nm).

11. Emission Spectra and Triplet Energy Measurements

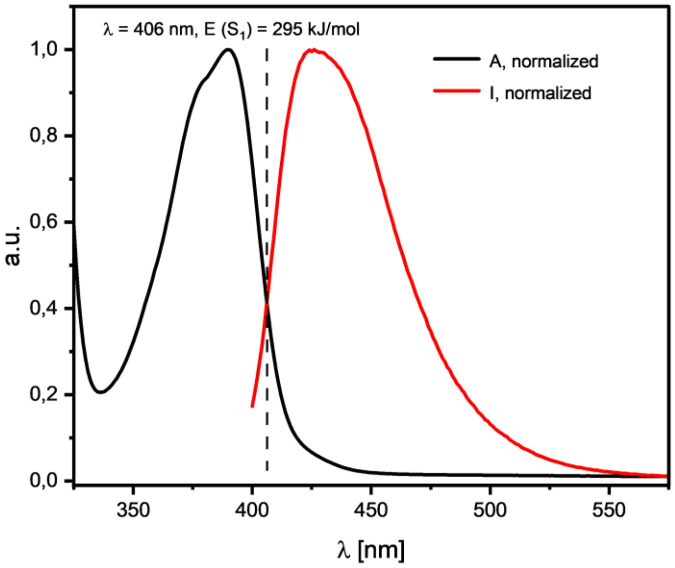

Figure S11.1. UV/VIS spectrum of **3f** in dichloromethane (c = 0.5 mM) normalized to the absorption maximum at 390 nm; luminescence of **3f** in dichloromethane (c = 50 µM) at room temperature, normalized to the emission maximum at 426 nm.

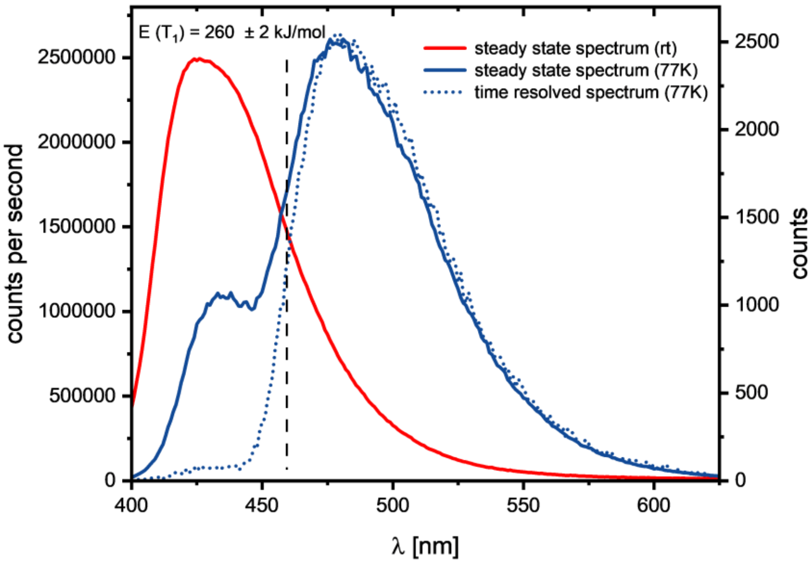

Figure S11.2. Steady state spectra of **3f** in dichloromethane (c = 50 µM) at room temperature and at 77 K given in counts per second (solid lines), time resolved spectrum of **3f** in dichloromethane (c = 50 µM) at 77 K after 100 µs delay in counts (dashed line).

12. Chiral HPLC/GLC Traces

Methyl (*R*)-2-benzyl-5,5-dimethylhexa-2,3-dienoate (2c)

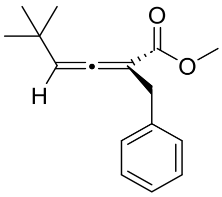

*Racemic Product rac-**2c***

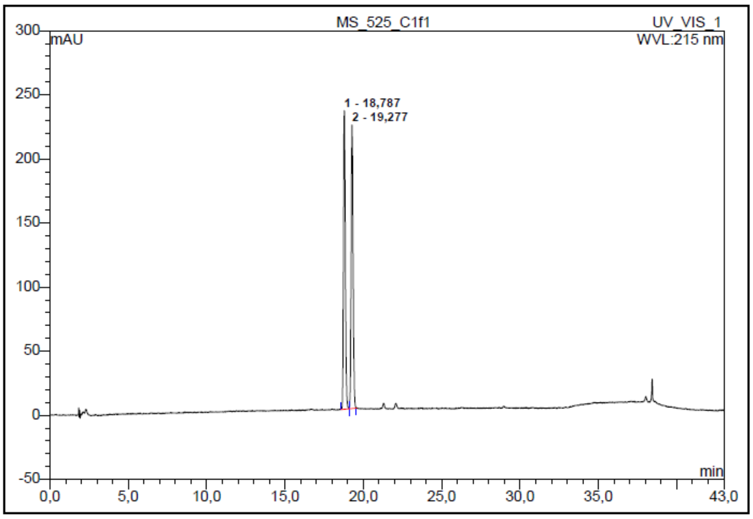

No.	Ret.Time min	Peak Name	Height mAU	Area mAU*min	Rel.Area %	Amount	Type
1	18,79	n.a.	233,127	32,432	50,19	n.a.	BM
2	19,28	n.a.	221,339	32,187	49,81	n.a.	MB
Total:			454,466	64,618	100,00	0,000	

*Enantioenriched Product **2c***

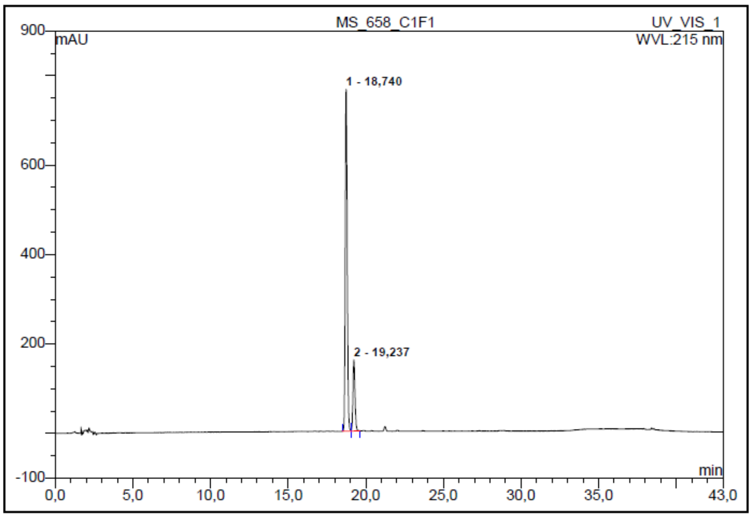

No.	Ret.Time min	Peak Name	Height mAU	Area mAU*min	Rel.Area %	Amount	Type
1	18,74	n.a.	764,263	113,765	82,34	n.a.	BM
2	19,24	n.a.	159,220	24,395	17,66	n.a.	MB
Total:			923,482	138,160	100,00	0,000	

*Attempted Deracemization of rac-**2c***

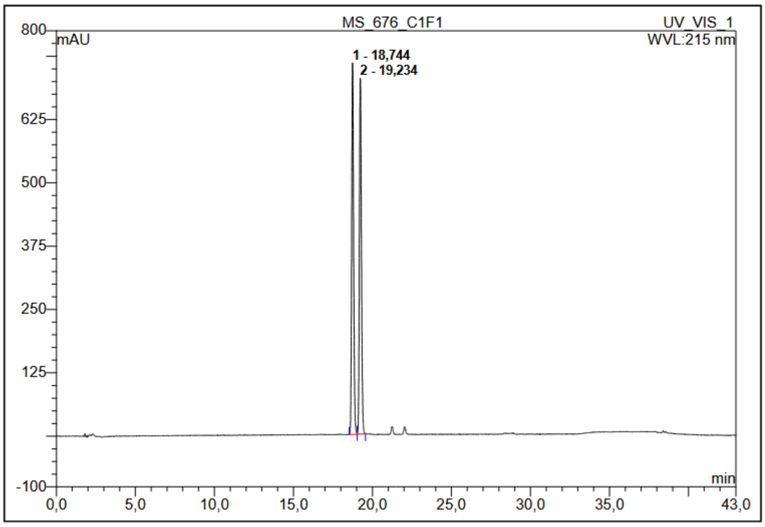

No.	Ret.Time<br>min	Peak Name	Height<br>mAU	Area<br>mAU*min	Rel.Area<br>%	Amount	Type
1	18,74	n.a.	733,541	104,573	49,95	n.a.	BM
2	19,23	n.a.	702,174	104,797	50,05	n.a.	MB
Total:			1435,715	209,370	100,00	0,000	

(*R*)-2-(4-Fluorobenzyl)-5,5-dimethylhexa-2,3-dienoic acid (1d)

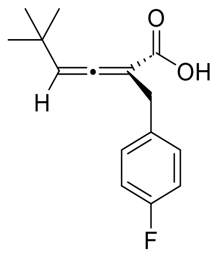

*Racemic Product rac-**1d***

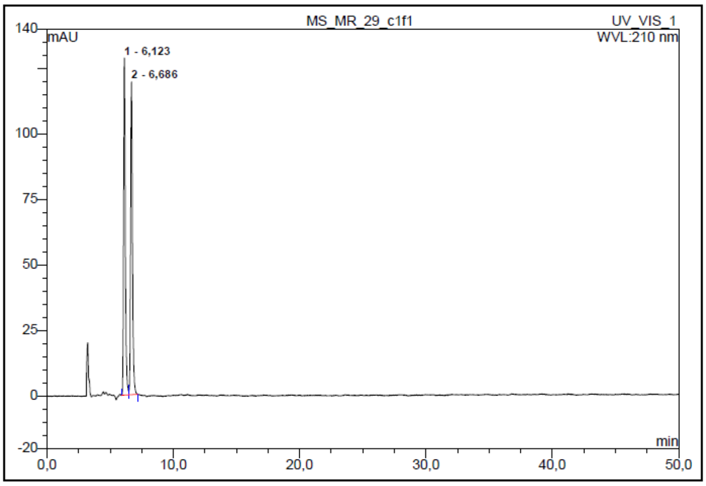

No.	Ret.Time min	Peak Name	Height mAU	Area mAU*min	Rel.Area %	Amount	Type
1	6,12	n.a.	128,454	20,645	49,80	n.a.	BM
2	6,69	n.a.	119,274	20,808	50,20	n.a.	MB
Total:			247,729	41,454	100,00	0,000	

*Enantioenriched Product **1d***

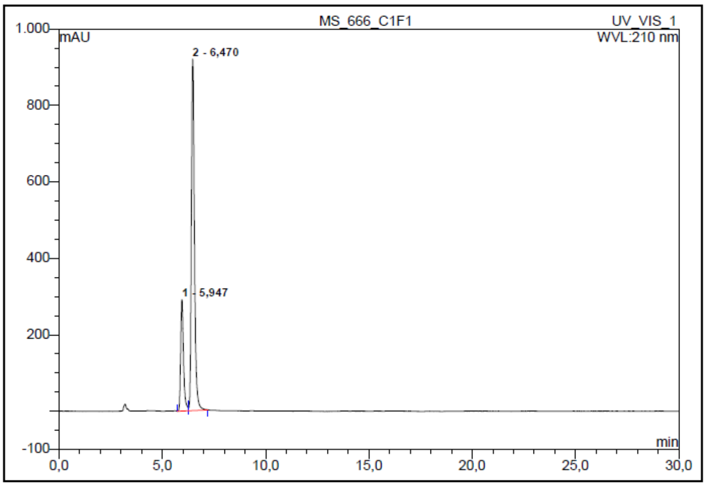

No.	Ret.Time min	Peak Name	Height mAU	Area mAU*min	Rel.Area %	Amount	Type
1	5,95	n.a.	291,642	46,417	22,74	n.a.	BM
2	6,47	n.a.	919,736	157,704	77,26	n.a.	MB
Total:			1211,378	204,121	100,00	0,000	

Methyl (*R*)-2-(4-bromobenzyl)-5,5-dimethylhexa-2,3-dienoate (2e)

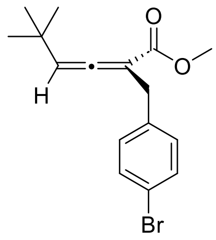

*Racemic Product rac-**2e***

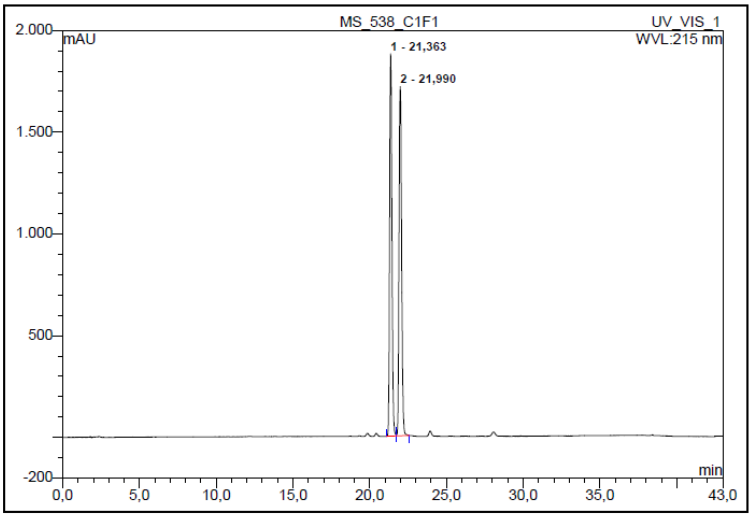

No.	Ret.Time min	Peak Name	Height mAU	Area mAU*min	Rel.Area %	Amount	Type
1	21,36	n.a.	1879,245	334,315	49,86	n.a.	BM
2	21,99	n.a.	1716,802	336,128	50,14	n.a.	MB
Total:			3596,048	670,443	100,00	0,000	

*Enantioenriched Product **2e***

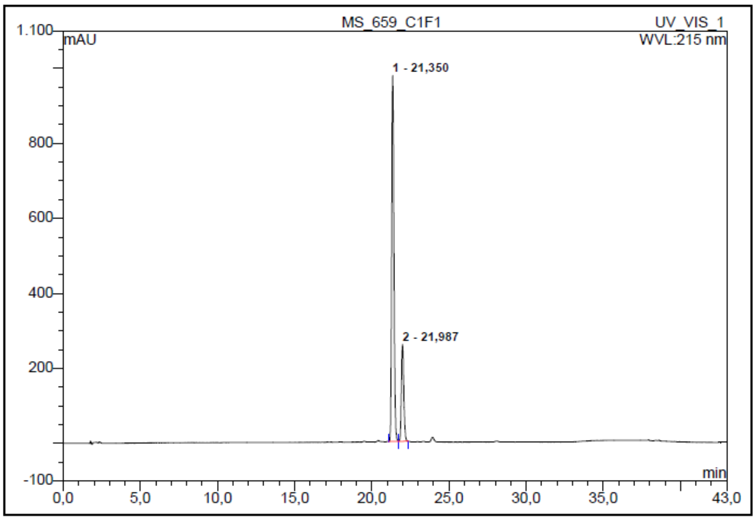

No.	Ret.Time min	Peak Name	Height mAU	Area mAU*min	Rel.Area %	Amount	Type
1	21,35	n.a.	976,365	168,346	78,23	n.a.	BM
2	21,99	n.a.	258,596	46,856	21,77	n.a.	MB
Total:			1234,961	215,202	100,00	0,000	

(*R*)-2-(4-Iodobenzyl)-5,5-dimethylhexa-2,3-dienoic acid (1f)

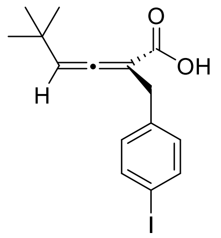

*Racemic Product rac-**1f***

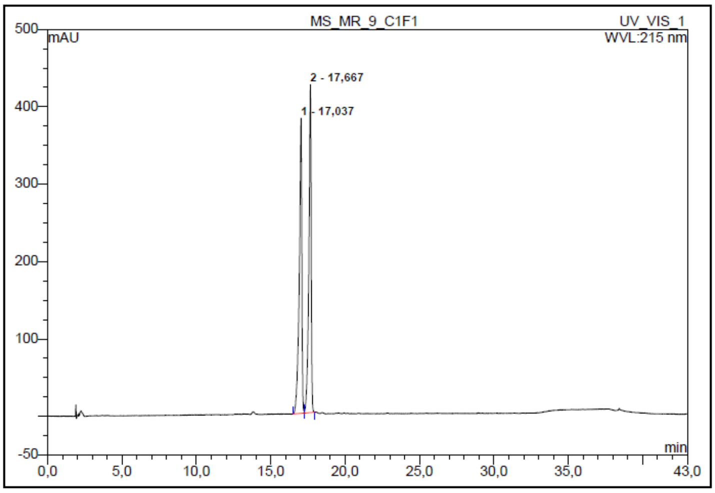

No.	Ret.Time min	Peak Name	Height mAU	Area mAU*min	Rel.Area %	Amount	Type
1	17,04	n.a.	381,271	75,335	49,91	n.a.	BM
2	17,67	n.a.	423,536	75,616	50,09	n.a.	MB
Total:			804,807	150,951	100,00	0,000	

*Enantioenriched Product **1f***

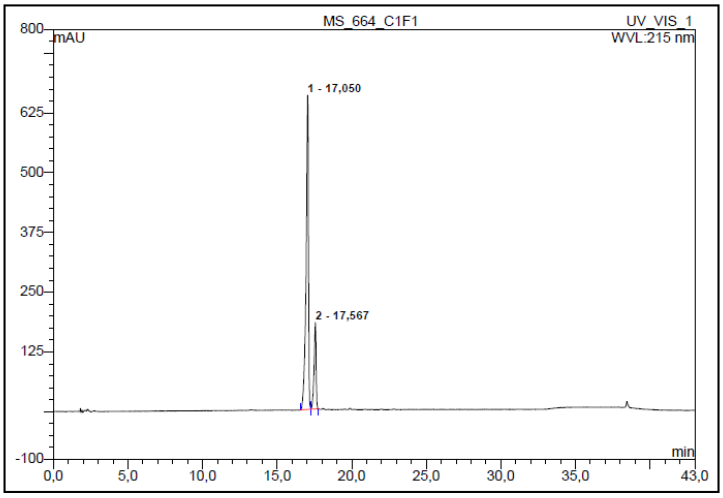

No.	Ret.Time min	Peak Name	Height mAU	Area mAU*min	Rel.Area %	Amount	Type
1	17,05	n.a.	657,596	119,785	80,95	n.a.	BM
2	17,57	n.a.	180,841	28,189	19,05	n.a.	MB
Total:			838,437	147,975	100,00	0,000	

*Enantioenriched Product **1f** (0.5 mmol Reaction)*

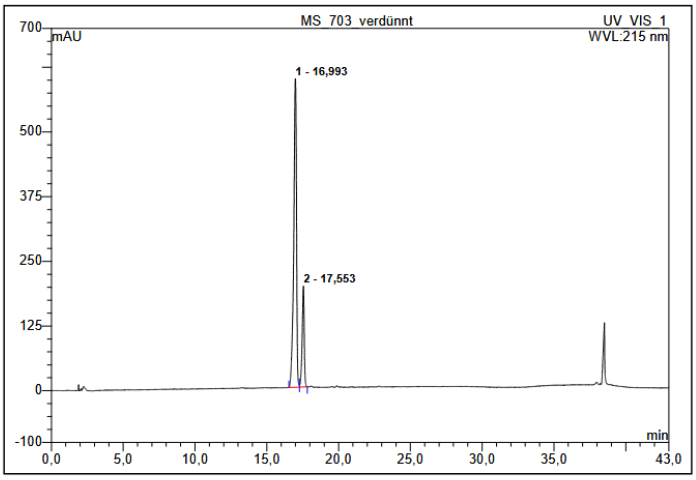

No.	Ret.Time min	Peak Name	Height mAU	Area mAU*min	Rel.Area %	Amount	Type
1	16,99	n.a.	596,468	132,879	80,45	n.a.	BM
2	17,55	n.a.	195,128	32,287	19,55	n.a.	MB
Total:			791,596	165,166	100,00	0,000	

Methyl (*R*)-2-(4-(tert-butyl)benzyl)-5,5-dimethylhexa-2,3-dienoate (2g)

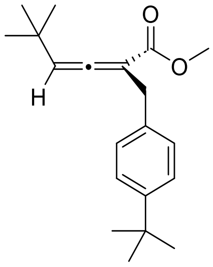

*Racemic Product rac-**2g***

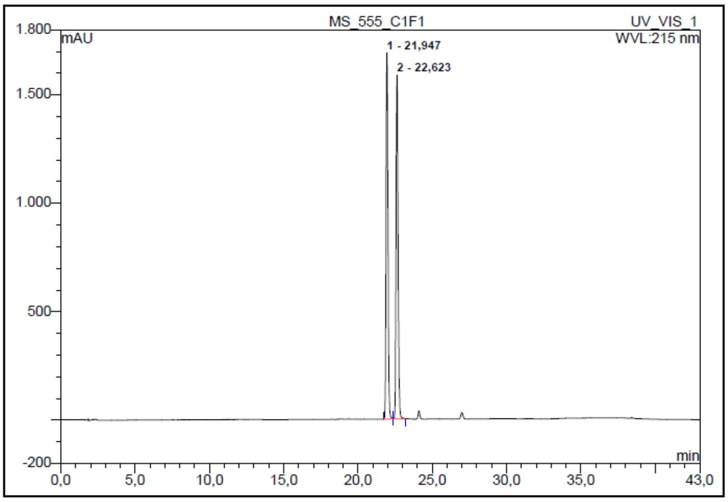

No.	Ret.Time min	Peak Name	Height mAU	Area mAU*min	Rel.Area %	Amount	Type
1	21,95	n.a.	1689,813	253,266	49,79	n.a.	BM
2	22,62	n.a.	1586,588	255,381	50,21	n.a.	MB
Total:			3276,401	508,647	100,00	0,000	

*Enantioenriched Product **2g***

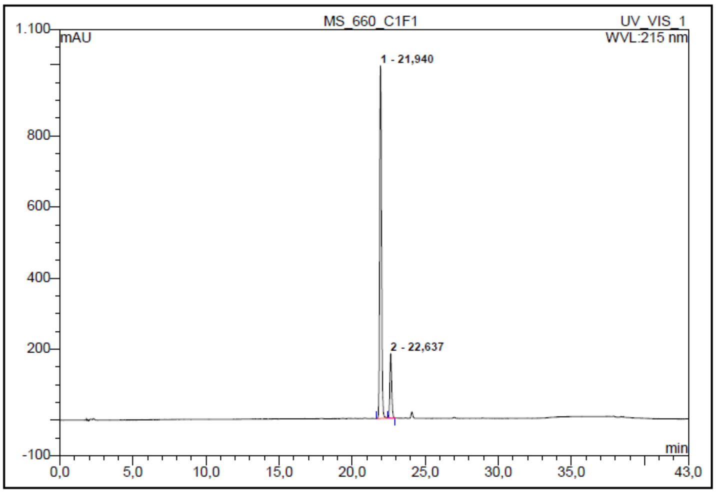

No.	Ret.Time min	Peak Name	Height mAU	Area mAU*min	Rel.Area %	Amount	Type
1	21,94	n.a.	992,696	144,505	84,48	n.a.	BMB
2	22,64	n.a.	181,505	26,557	15,52	n.a.	Rd
Total:			1174,201	171,062	100,00	0,000	

(*R*)-5,5-Dimethyl-2-(4-(methylthio)benzyl)hexa-2,3-dienoic acid (1h)

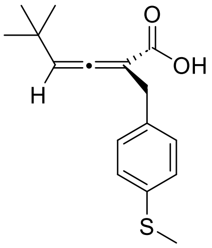

*Racemic Product rac-**1h***

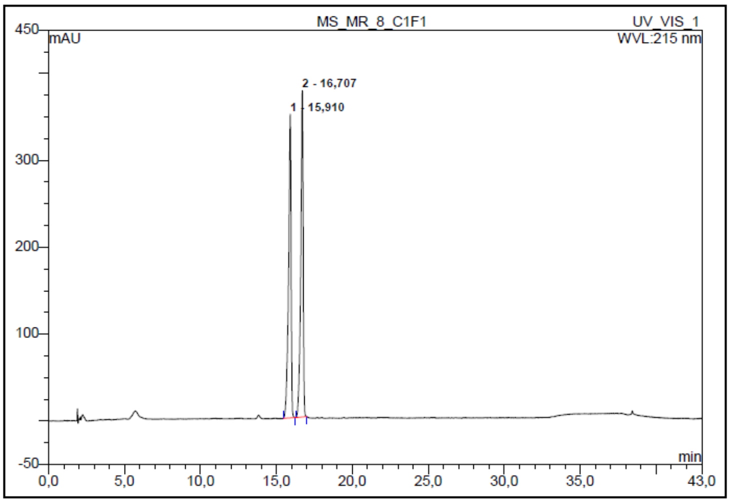

No.	Ret.Time min	Peak Name	Height mAU	Area mAU*min	Rel.Area %	Amount	Type
1	15,91	n.a.	349,302	67,231	49,51	n.a.	BMB
2	16,71	n.a.	375,526	68,556	50,49	n.a.	BMB
Total:			724,828	135,788	100,00	0,000	

*Enantioenriched Product **1h***

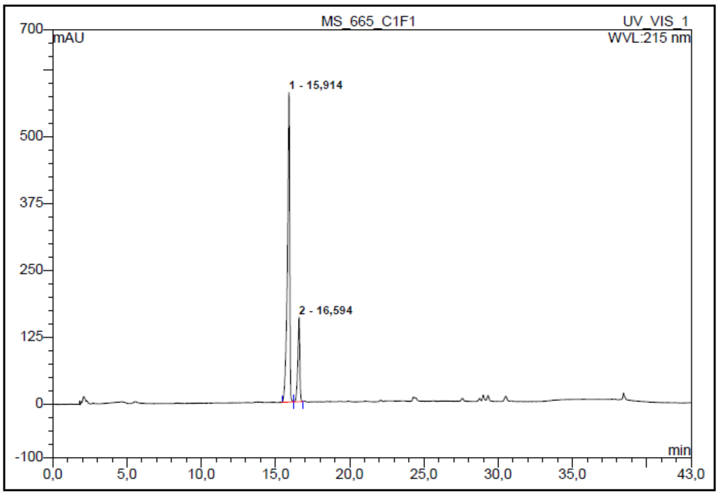

No.	Ret.Time min	Peak Name	Height mAU	Area mAU*min	Rel.Area %	Amount	Type
1	15,91	n.a.	578,469	103,841	80,89	n.a.	BM
2	16,59	n.a.	157,643	24,534	19,11	n.a.	MB
Total:			736,112	128,375	100,00	0,000	

Methyl (*R*)-2-(3,5-dimethylbenzyl)-5,5-dimethylhexa-2,3-dienoate (2i)

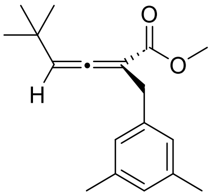

*Racemic Product rac-**2i***

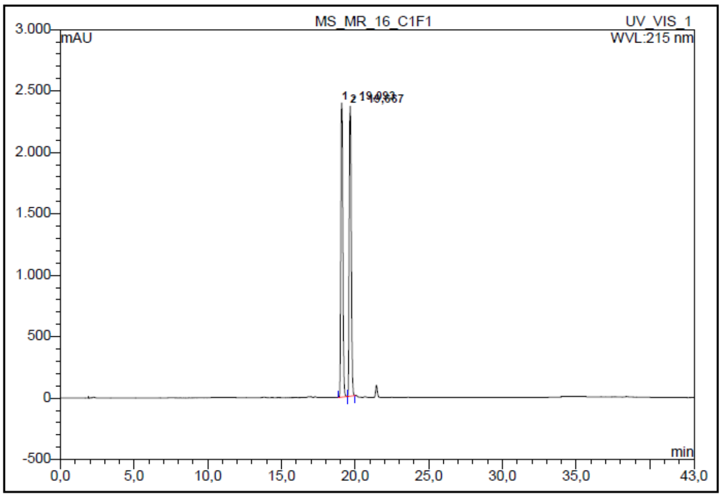

No.	Ret.Time min	Peak Name	Height mAU	Area mAU*min	Rel.Area %	Amount	Type
1	19,09	n.a.	2394,928	382,854	49,88	n.a.	BM
2	19,67	n.a.	2360,522	384,652	50,12	n.a.	MB
Total:			4755,450	767,506	100,00	0,000	

*Enantioenriched Product **2i***

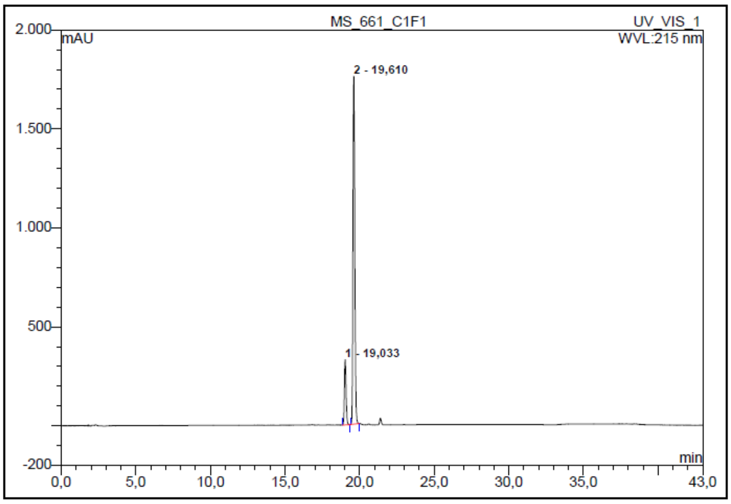

No.	Ret.Time min	Peak Name	Height mAU	Area mAU*min	Rel.Area %	Amount	Type
1	19,03	n.a.	328,957	43,343	14,66	n.a.	BMB
2	19,61	n.a.	1757,142	252,390	85,34	n.a.	BMB
Total:			2086,099	295,733	100,00	0,000	

Methyl (*R*)-2-(3,5-di-tert-butylbenzyl)-5,5-dimethylhexa-2,3-dienoate (2j)

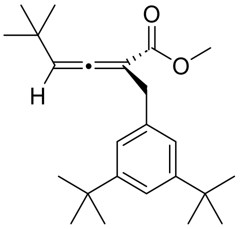

*Racemic Product rac-**2j***

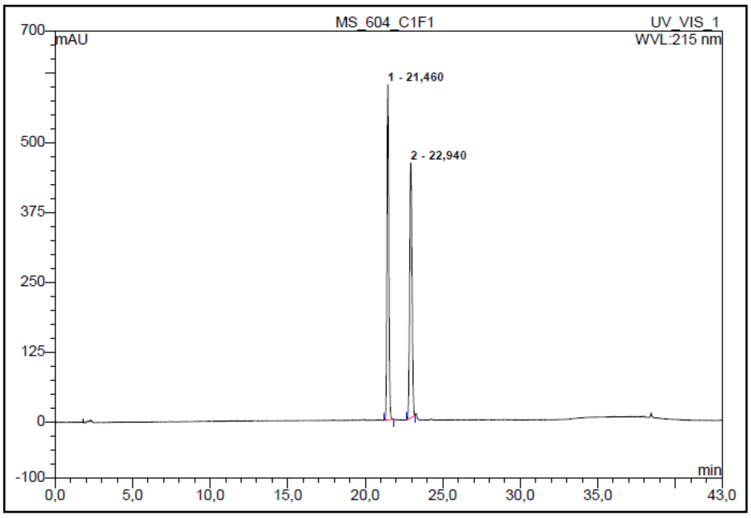

No.	Ret.Time min	Peak Name	Height mAU	Area mAU*min	Rel.Area %	Amount	Type
1	21,46	n.a.	599,997	82,177	50,07	n.a.	BMB
2	22,94	n.a.	455,836	81,935	49,93	n.a.	BMB
Total:			1055,832	164,113	100,00	0,000	

*Enantioenriched Product **2j***

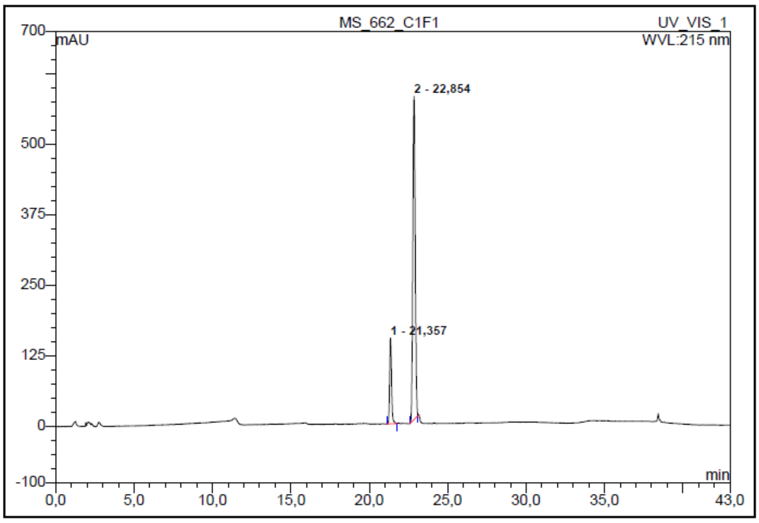

No.	Ret.Time min	Peak Name	Height mAU	Area mAU*min	Rel.Area %	Amount	Type
1	21,36	n.a.	152,260	21,620	17,37	n.a.	BMB
2	22,85	n.a.	572,186	102,839	82,63	n.a.	BMB
Total:			724,447	124,459	100,00	0,000	

Methyl (*R*)-2-([1,1'-biphenyl]-3-ylmethyl)-5,5-dimethylhexa-2,3-dienoate (2k)

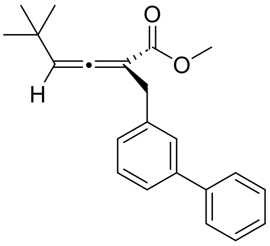

*Racemic Product rac-**2k***

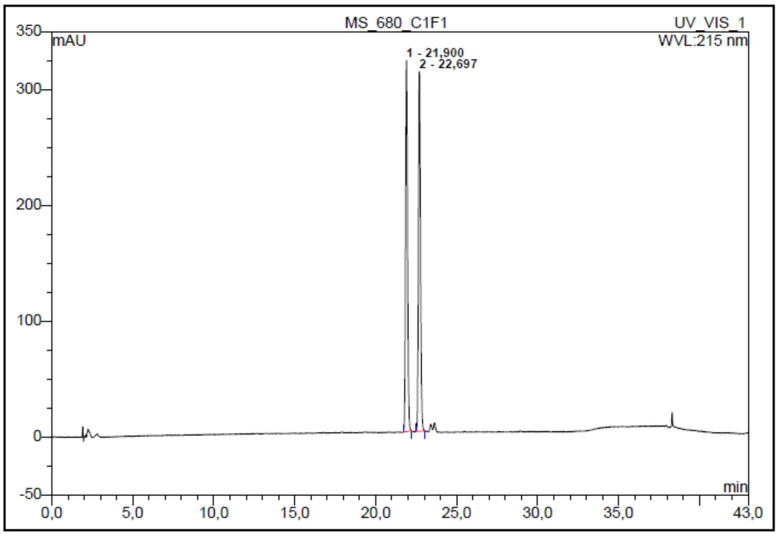

No.	Ret.Time min	Peak Name	Height mAU	Area mAU*min	Rel.Area %	Amount	Type
1	21,90	n.a.	320,336	45,699	49,94	n.a.	BMB
2	22,70	n.a.	310,556	45,811	50,06	n.a.	BMB
Total:			630,892	91,510	100,00	0,000	

*Enantioenriched Product **2k***

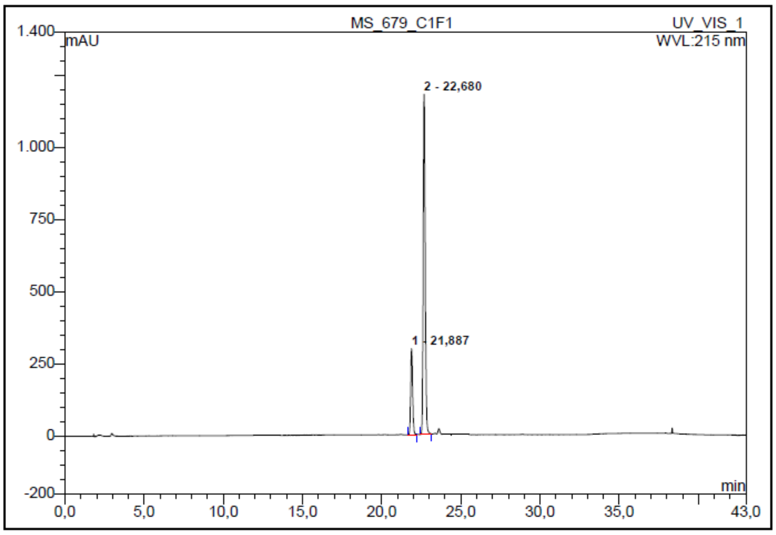

No.	Ret.Time min	Peak Name	Height mAU	Area mAU*min	Rel.Area %	Amount	Type
1	21,89	n.a.	298,090	42,485	19,19	n.a.	BM *
2	22,68	n.a.	1178,209	178,932	80,81	n.a.	BM *
Total:			1476,299	221,416	100,00	0,000	

(*R*)-2-(3-Cyanobenzyl)-5,5-dimethylhexa-2,3-dienoic acid (1l)

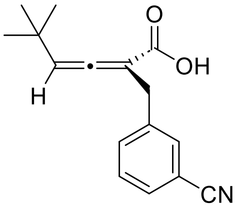

*Racemic Product rac-**1l***

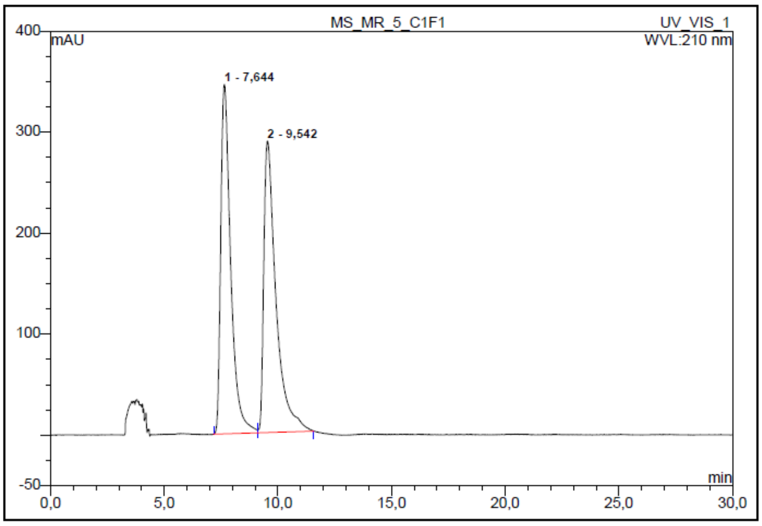

No.	Ret.Time min	Peak Name	Height mAU	Area mAU*min	Rel.Area %	Amount	Type
1	7,64	n.a.	345,959	175,797	49,50	n.a.	BM
2	9,54	n.a.	288,590	179,321	50,50	n.a.	MB
Total:			634,548	355,117	100,00	0,000	

*Enantioenriched Product **1l***

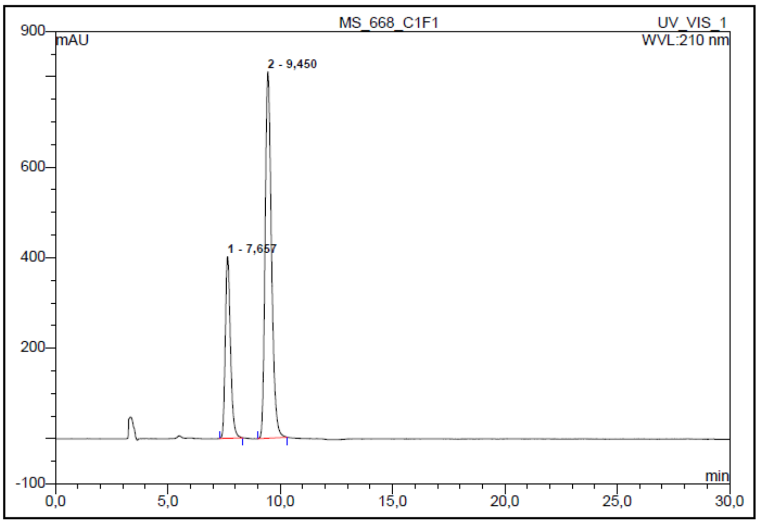

No.	Ret.Time min	Peak Name	Height mAU	Area mAU*min	Rel.Area %	Amount	Type
1	7,66	n.a.	401,252	107,446	28,80	n.a.	BMB
2	9,45	n.a.	809,749	265,614	71,20	n.a.	BMB
Total:			1211,000	373,060	100,00	0,000	

Methyl (*R*)-2-(3-cyanobenzyl)-5,5-dimethylhexa-2,3-dienoate (2l)

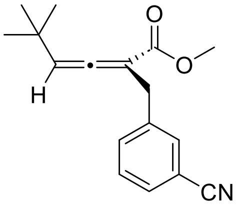

*Racemic Product rac-**2l***

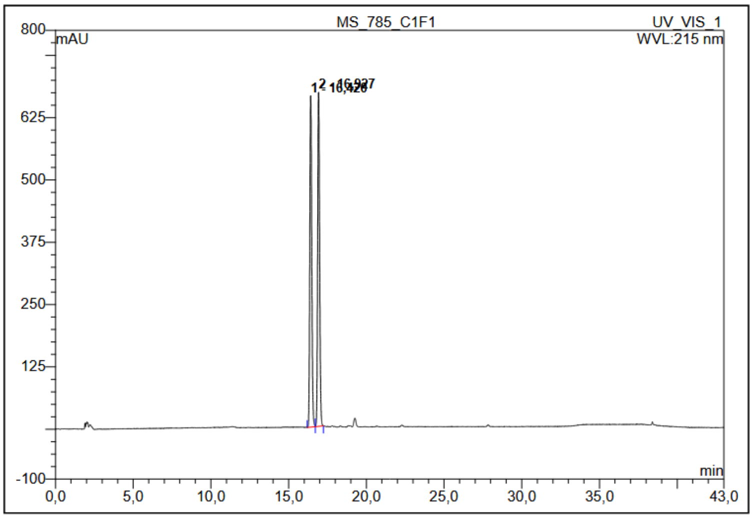

No.	Ret.Time min		Peak Name	Height mAU	Area mAU*min	Rel.Area %	Amount	Type
1	16,42	n.a.		665,183	96,197	49,92	n.a.	BM
2	16,93	n.a.		670,556	96,502	50,08	n.a.	MB
Total:				1335,739	192,699	100,00	0,000	

*Enantioenriched Product **2l***

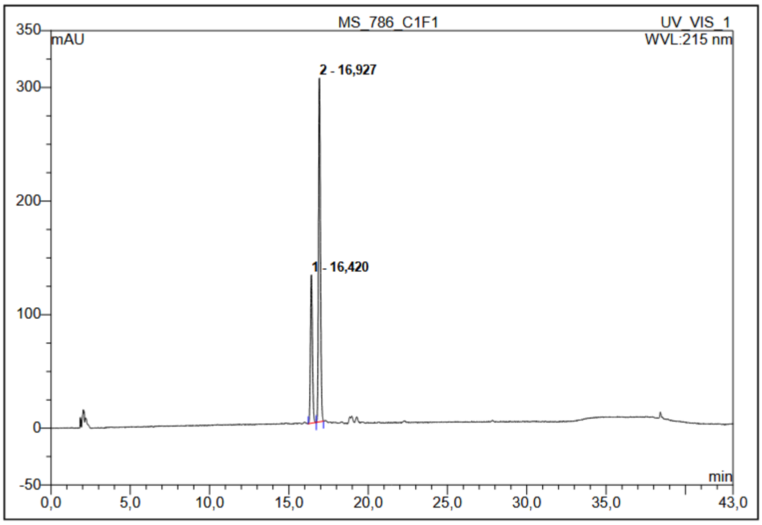

No.	Ret.Time min		Peak Name	Height mAU	Area mAU*min	Rel.Area %	Amount	Type
1	16,42	n.a.		130,653	18,765	30,30	n.a.	BM
2	16,93	n.a.		302,635	43,162	69,70	n.a.	MB
Total:				433,288	61,927	100,00	0,000	

(*R*)-2-(3-Methoxybenzyl)-5,5-dimethylhexa-2,3-dienoic acid (1m)

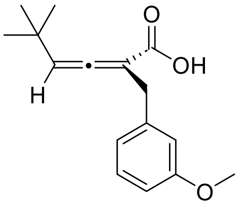

*Racemic Product rac-**1m***

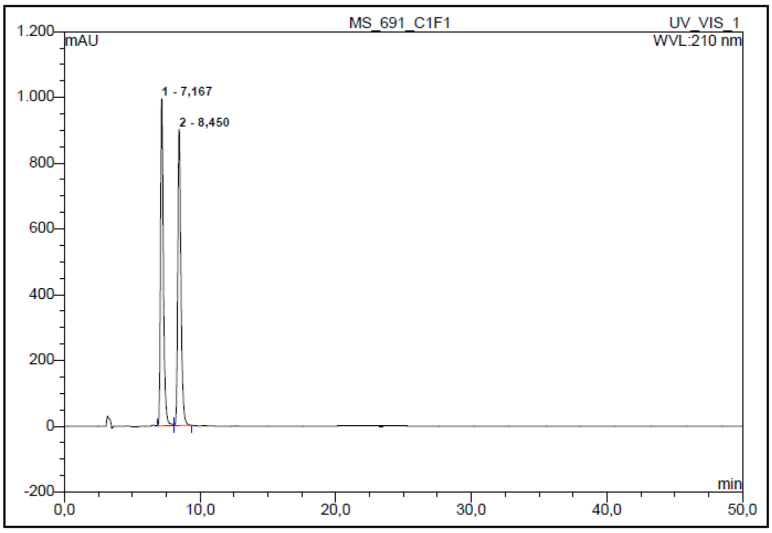

No.	Ret.Time min	Peak Name	Height mAU	Area mAU*min	Rel.Area %	Amount	Type
1	7,17	n.a.	995,796	246,299	49,99	n.a.	BM
2	8,45	n.a.	900,559	246,351	50,01	n.a.	MB
Total:			1896,355	492,650	100,00	0,000	

*Enantioenriched Product **1m***

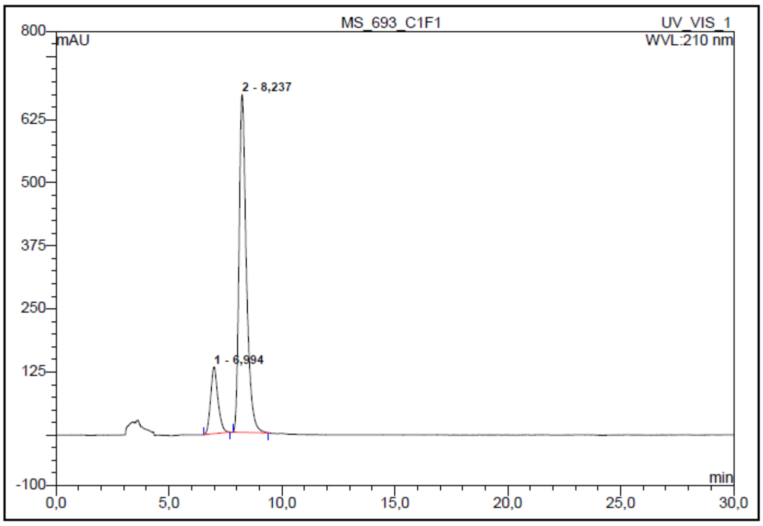

No.	Ret.Time min	Peak Name	Height mAU	Area mAU*min	Rel.Area %	Amount	Type
1	6,99	n.a.	132,394	49,504	16,44	n.a.	BMB*
2	8,24	n.a.	669,286	251,551	83,56	n.a.	BMB*
Total:			801,680	301,055	100,00	0,000	

(*R*)-2-(Benzo[*d*][1,3]dioxol-5-ylmethyl)-5,5-dimethylhexa-2,3-dienoic acid (1n)

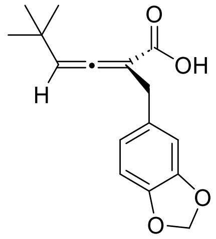

*Racemic Product rac-**1n***

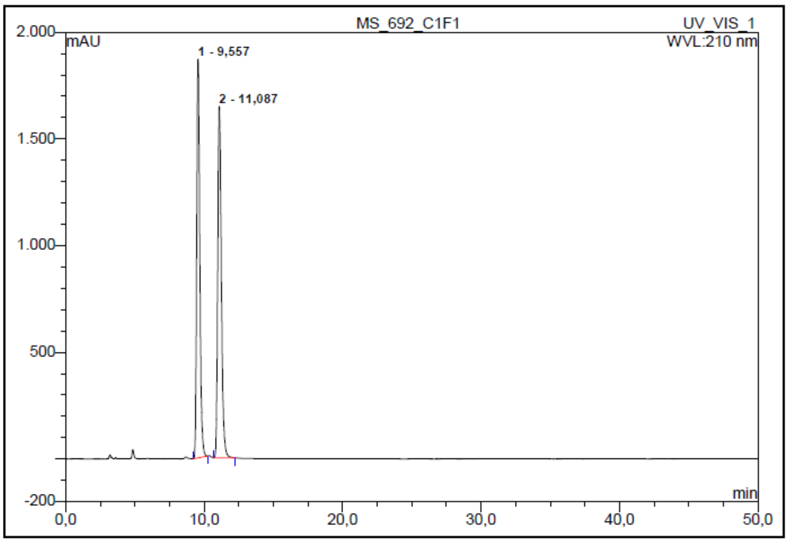

No.	Ret.Time min	Peak Name	Height mAU	Area mAU*min	Rel.Area %	Amount	Type
1	9,56	n.a.	1868,536	489,875	49,32	n.a.	BMB
2	11,09	n.a.	1646,939	503,303	50,68	n.a.	BMB
Total:			3515,475	993,178	100,00	0,000	

*Enantioenriched Product **1n***

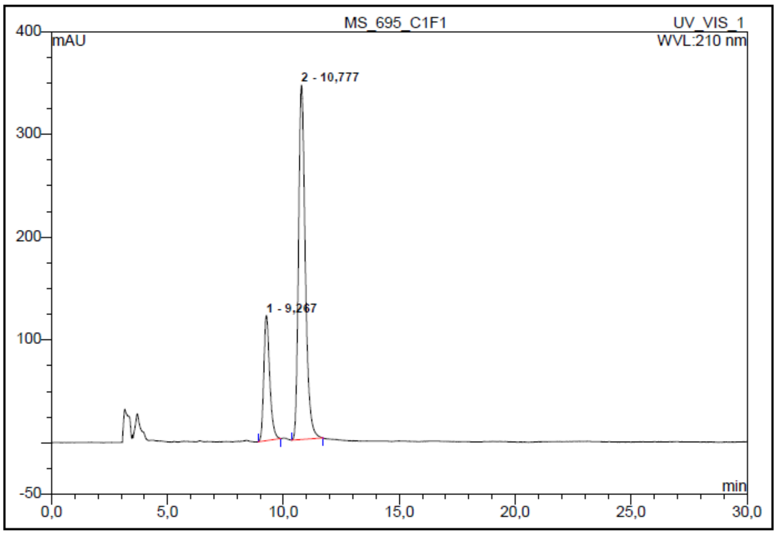

No.	Ret.Time min	Peak Name	Height mAU	Area mAU*min	Rel.Area %	Amount	Type
1	9,27	n.a.	121,716	35,987	23,48	n.a.	BMB
2	10,78	n.a.	344,896	117,294	76,52	n.a.	BMB
Total:			466,612	153,281	100,00	0,000	

(*R*)-2-(2,6-Dichlorobenzyl)-5,5-dimethylhexa-2,3-dienoic acid (1o)

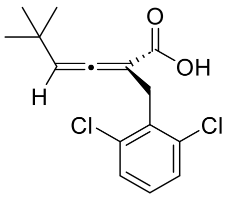

*Racemic Product rac-**1o***

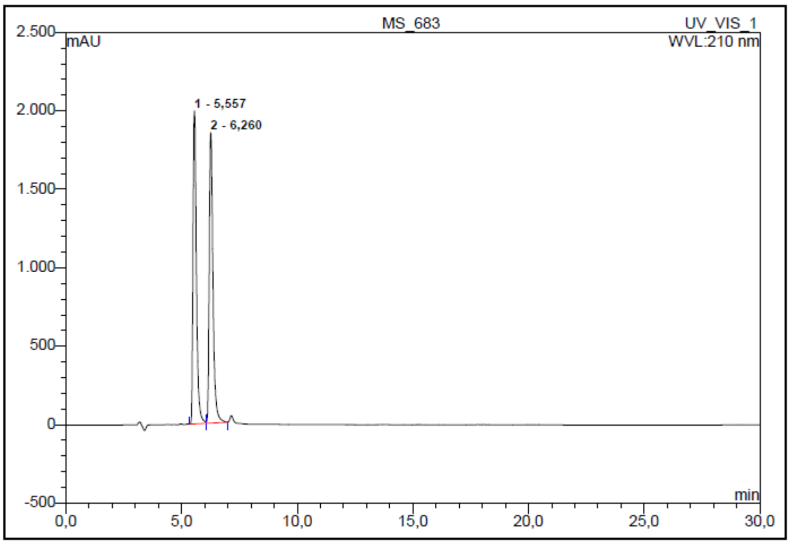

No.	Ret.Time min	Peak Name	Height mAU	Area mAU*min	Rel.Area %	Amount	Type
1	5,56	n.a.	1993,945	340,574	49,49	n.a.	BM *
2	6,26	n.a.	1851,363	347,557	50,51	n.a.	MB*
Total:			3845,308	688,131	100,00	0,000	

*Enantioenriched Product **1o***

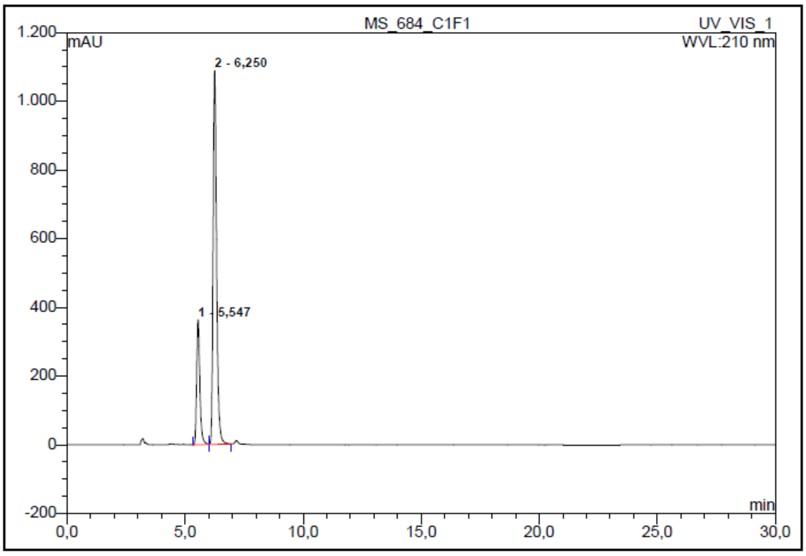

No.	Ret.Time min	Peak Name	Height mAU	Area mAU*min	Rel.Area %	Amount	Type
1	5,55	n.a.	363,858	57,006	23,35	n.a.	BM
2	6,25	n.a.	1088,006	187,163	76,65	n.a.	MB
Total:			1451,864	244,170	100,00	0,000	

(*R*)-5,5-Dimethyl-2-((perfluorophenyl)methyl)hexa-2,3-dienoic acid (1p)

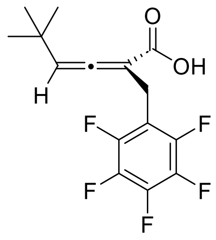

*Racemic Product rac-**1p***

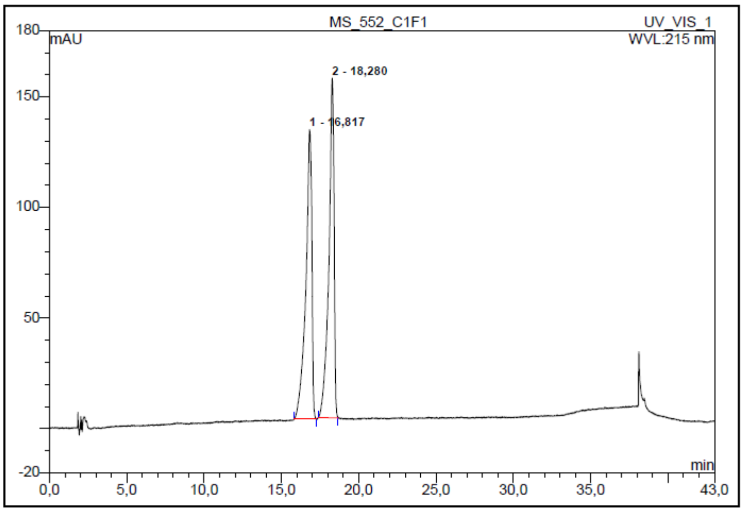

No.	Ret.Time min	Peak Name	Height mAU	Area mAU*min	Rel.Area %	Amount	Type
1	16,82	n.a.	130,786	59,160	49,70	n.a.	BMB
2	18,28	n.a.	153,651	59,883	50,30	n.a.	BMB
Total:			284,437	119,043	100,00	0,000	

*Enantioenriched Product **1p***

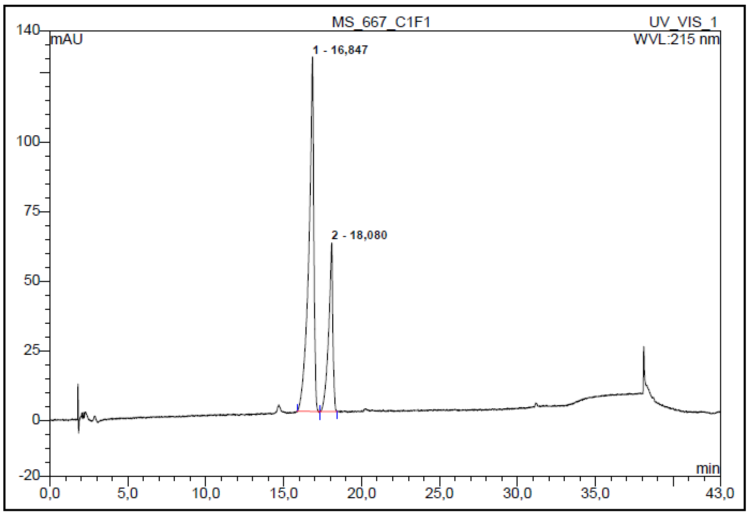

No.	Ret.Time min	Peak Name	Height mAU	Area mAU*min	Rel.Area %	Amount	Type
1	16,85	n.a.	127,287	47,520	71,48	n.a.	BM
2	18,08	n.a.	60,517	18,957	28,52	n.a.	MB
Total:			187,804	66,477	100,00	0,000	

(*R*)-5,5-Dimethyl-2-(naphthalen-1-ylmethyl)hexa-2,3-dienoic acid (1q)

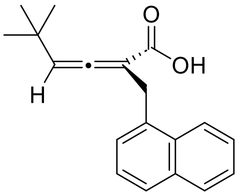

*Racemic Product rac-**1q***

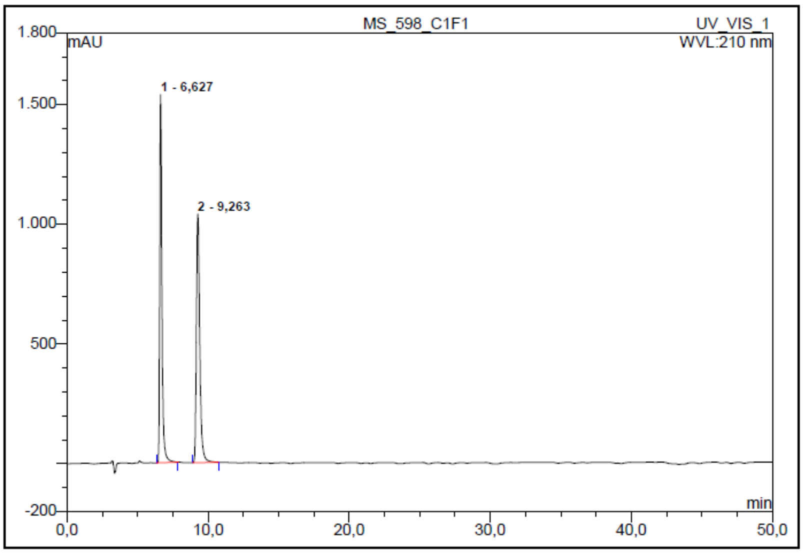

No.	Ret.Time min	Peak Name	Height mAU	Area mAU*min	Rel.Area %	Amount	Type
1	6,63	n.a.	1537,995	284,410	49,78	n.a.	BMB
2	9,26	n.a.	1039,677	286,892	50,22	n.a.	BMB
Total:			2577,672	571,302	100,00	0,000	

*Enantioenriched Product **1q***

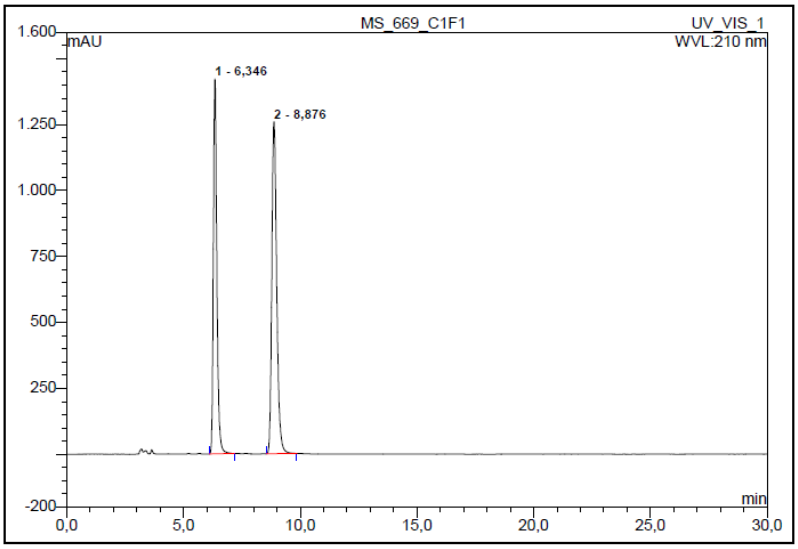

No.	Ret.Time min	Peak Name	Height mAU	Area mAU*min	Rel.Area %	Amount	Type
1	6,35	n.a.	1420,428	247,209	44,63	n.a.	BMB
2	8,88	n.a.	1258,111	306,643	55,37	n.a.	BMB
Total:			2678,540	553,852	100,00	0,000	

Methyl (*R*)-2-benzylpenta-2,3-dienoate (2r)

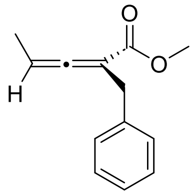

*Racemic Product rac-**2r***

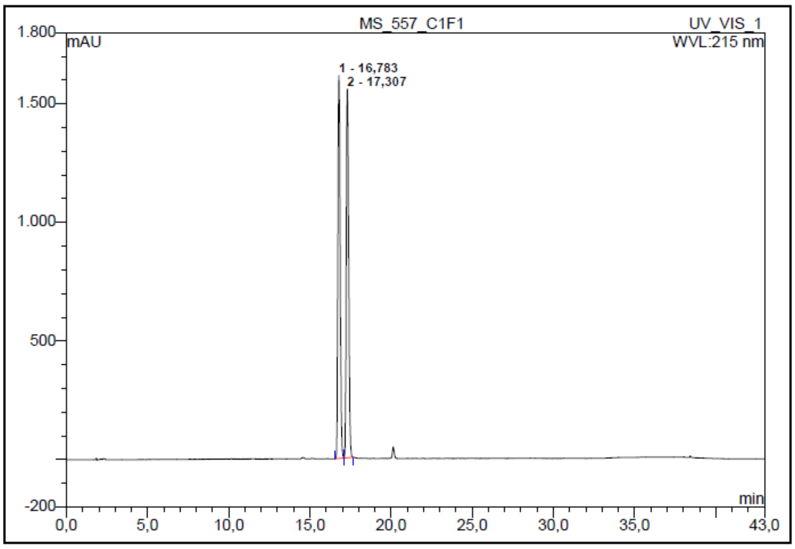

No.	Ret.Time min	Peak Name	Height mAU	Area mAU*min	Rel.Area %	Amount	Type
1	16,78	n.a.	1613,985	264,488	49,89	n.a.	BM
2	17,31	n.a.	1554,460	265,695	50,11	n.a.	MB
Total:			3168,446	530,182	100,00	0,000	

*Enantioenriched Product **2r***

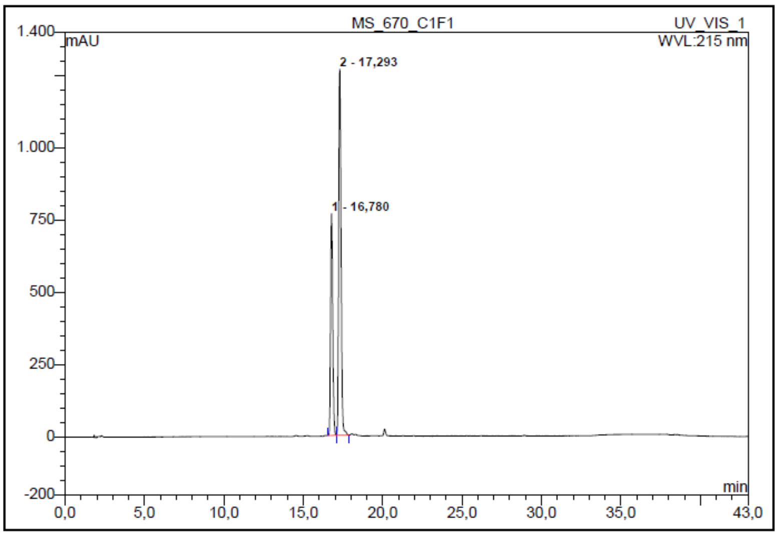

No.	Ret.Time min	Peak Name	Height mAU	Area mAU*min	Rel.Area %	Amount	Type
1	16,78	n.a.	766,022	120,850	35,48	n.a.	BM
2	17,29	n.a.	1264,389	219,769	64,52	n.a.	MB
Total:			2030,411	340,618	100,00	0,000	

(*R*)-2-Benzyl-4-cyclopentylbuta-2,3-dienoic acid (1s)

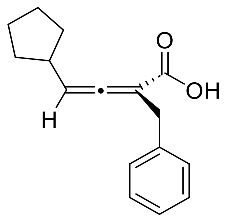

*Racemic Product rac-**1s***

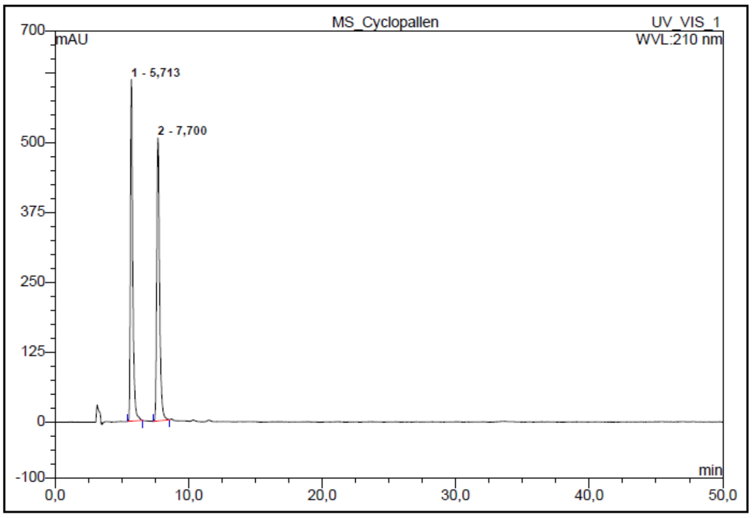

No.	Ret.Time min	Peak Name	Height mAU	Area mAU*min	Rel.Area %	Amount	Type
1	5,71	n.a.	610,770	126,050	50,10	n.a.	BMB
2	7,70	n.a.	505,977	125,548	49,90	n.a.	BMB
Total:			1116,747	251,598	100,00	0,000	

*Enantioenriched Product **1s***

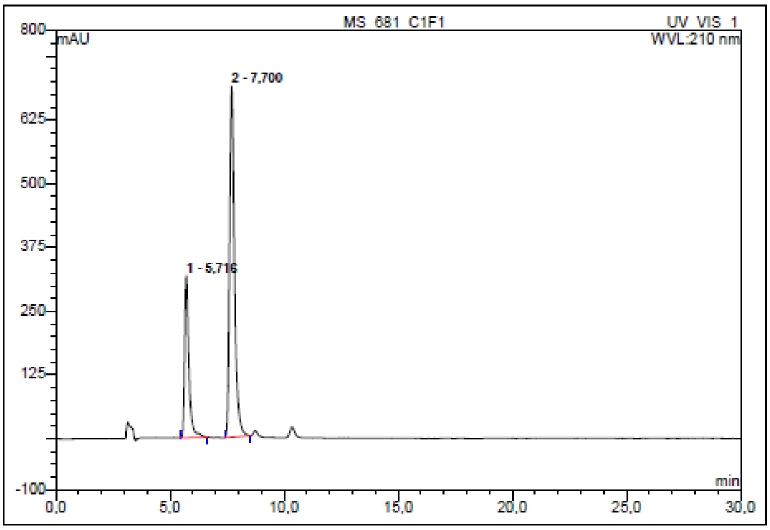

No.	Ret.Time min	Peak Name	Height mAU	Area mAU*min	Rel.Area %	Amount	Type
1	5,72	n.a.	317,238	67,972	28,38	n.a.	BMB
2	7,70	n.a.	687,791	171,531	71,62	n.a.	BMB
Total:			1005,028	239,503	100,00	0,000	

(*R*)-2-Benzyl-5-methylhexa-2,3-dienoic acid (1t)

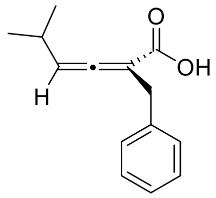

*Racemic Product rac-**1t***

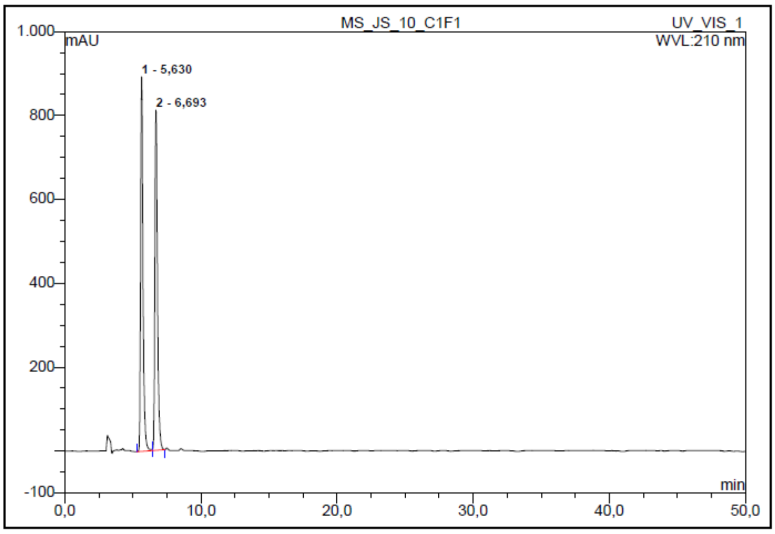

No.	Ret.Time min	Peak Name	Height mAU	Area mAU*min	Rel.Area %	Amount	Type
1	5,63	n.a.	893,226	180,727	50,33	n.a.	BM
2	6,69	n.a.	811,362	178,360	49,67	n.a.	MB
Total:			1704,588	359,087	100,00	0,000	

*Enantioenriched Product **1t***

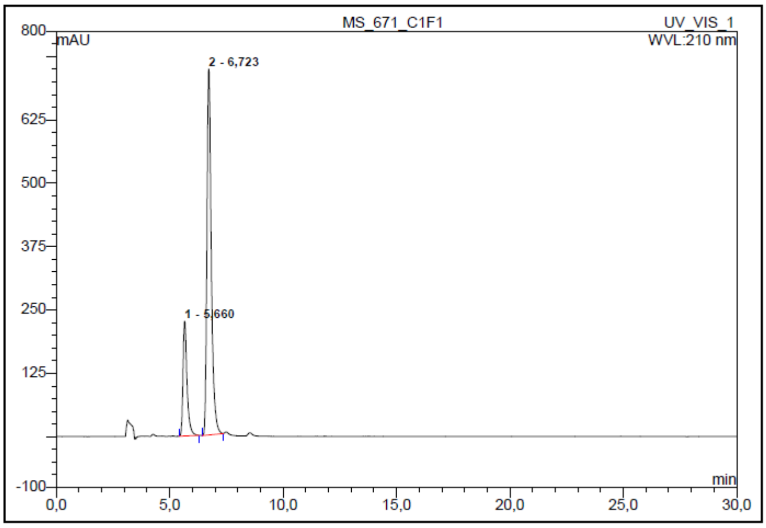

No.	Ret.Time min	Peak Name	Height mAU	Area mAU*min	Rel.Area %	Amount	Type
1	5,66	n.a.	226,321	46,222	22,36	n.a.	BMB
2	6,72	n.a.	721,552	160,458	77,64	n.a.	BMB
Total:			947,873	206,680	100,00	0,000	

Methyl (*R*)-4-((3*R*,5*R*,7*R*)-adamantan-1-yl)-2-benzylbuta-2,3-dienoate (1u)

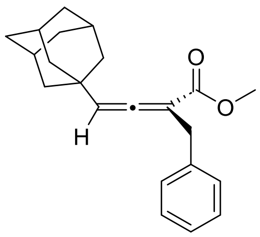

*Racemic Product rac-**1u***

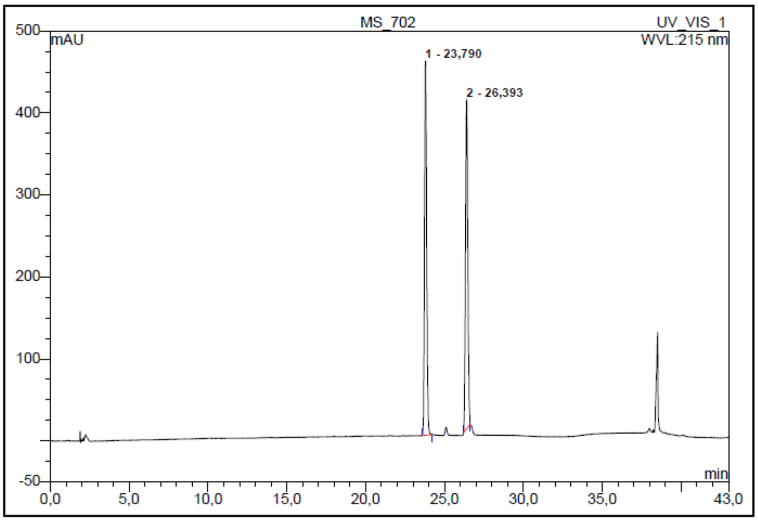

No.	Ret.Time min	Peak Name	Height mAU	Area mAU*min	Rel.Area %	Amount	Type
1	23,79	n.a.	456,486	71,528	51,12	n.a.	BMB
2	26,39	n.a.	401,348	68,392	48,88	n.a.	BMB*
Total:			857,834	139,920	100,00	0,000	

*Enantioenriched Product **1u***

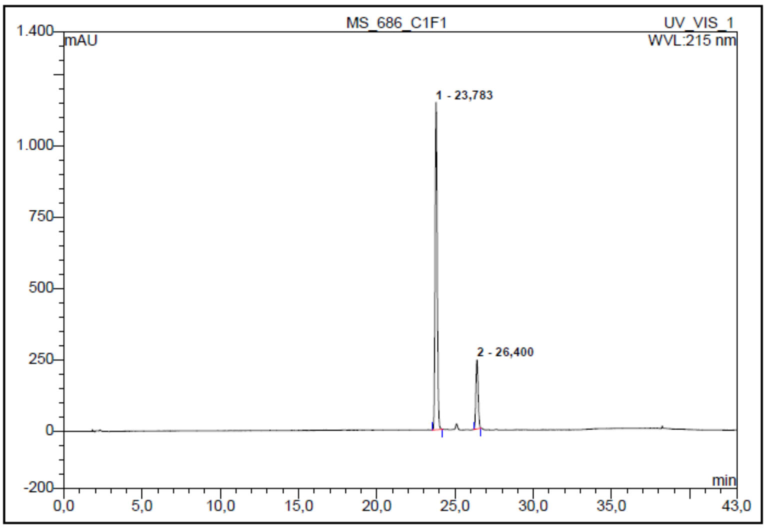

No.	Ret.Time min	Peak Name	Height mAU	Area mAU*min	Rel.Area %	Amount	Type
1	23,78	n.a.	1146,162	178,885	81,37	n.a.	BMB
2	26,40	n.a.	242,303	40,951	18,63	n.a.	BMB
Total:			1388,465	219,836	100,00	0,000	

(*R*)-5,5-Dimethylhexa-2,3-dienoic acid (1v)

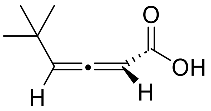

*Racemic Product rac-**1v***

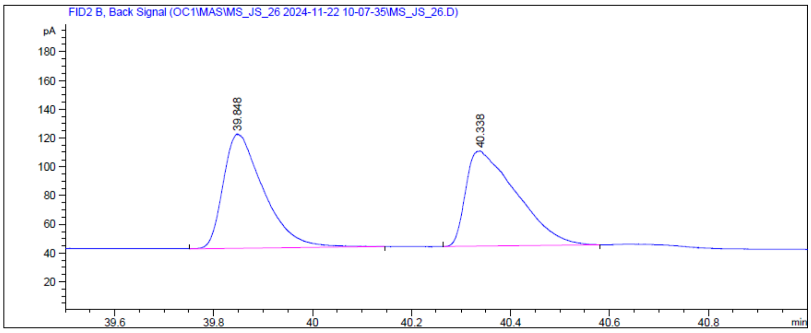

===
Area Percent Report
===

Sorted By : Signal
Multiplier : 1.0000
Dilution : 1.0000
Do not use Multiplier & Dilution Factor with ISTDs

Signal 1: FID2 B, Back Signal

Peak #	RetTime [min]	Type	Width [min]	Area [pA*s]	Height [pA]	Area %
1	39.848	BB	0.0709	449.77734	79.34736	48.86916
2	40.338	BB	0.0934	470.59326	66.13867	51.13084
Totals :				920.37061	145.48603	

*Enantioenriched Product **1v***

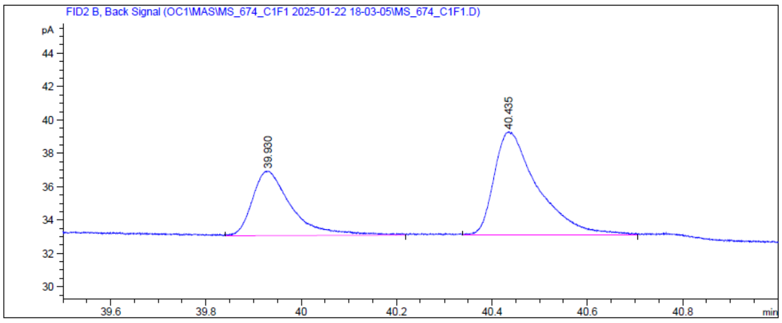

===
Area Percent Report
===

Sorted By : Signal
Multiplier : 1.0000
Dilution : 1.0000
Do not use Multiplier & Dilution Factor with ISTDs

Signal 1: FID2 B, Back Signal

Peak #	RetTime [min]	Type	Width [min]	Area [pA*s]	Height [pA]	Area %
1	39.930	BB	0.0819	22.03300	3.85358	35.86105
2	40.435	BB	0.0897	39.40693	6.16935	64.13895
Totals :				61.43993	10.02293	

(*R*)-2,5,5-Trimethylhexa-2,3-dienoic acid (1w)

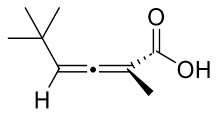

*Racemic Product rac-**1w***

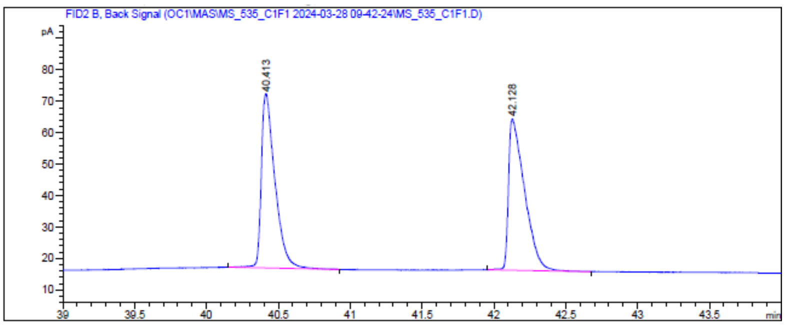


```
=====================================================================
                          Area Percent Report
=====================================================================

Sorted By             :      Signal
Multiplier            :      1.0000
Dilution              :      1.0000
Do not use Multiplier & Dilution Factor with ISTDs


Signal 1: FID2 B, Back Signal

Peak RetTime Type  Width     Area      Height     Area
  #   [min]        [min]   [pA*s]      [pA]        %
----|-------|----|-------|----------|----------|--------|
   1  40.413 BB    0.0925  365.69763   55.19336 49.51965
   2  42.128 BB    0.1017  372.79230   48.06238 50.48035

Totals :                   738.48993  103.25574
```

*Enantioenriched Product **1w***

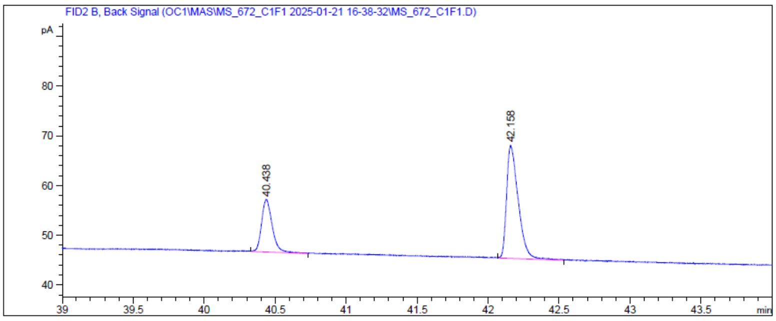


```
=====================================================================
                          Area Percent Report
=====================================================================

Sorted By             :      Signal
Multiplier            :      1.0000
Dilution              :      1.0000
Do not use Multiplier & Dilution Factor with ISTDs


Signal 1: FID2 B, Back Signal

Peak RetTime Type  Width     Area      Height     Area
  #   [min]        [min]   [pA*s]      [pA]        %
----|-------|----|-------|----------|----------|--------|
   1  40.438 BB    0.0738   54.38006   10.54429 29.72694
   2  42.158 BB    0.0819  128.55183   22.75368 70.27306

Totals :                   182.93190   33.29797
```

(*R*)-5,5-Dimethyl-2-(3-methylbut-2-en-1-yl)hexa-2,3-dienamide (6x)

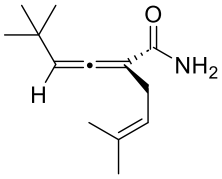

*Racemic Product rac-**6x***

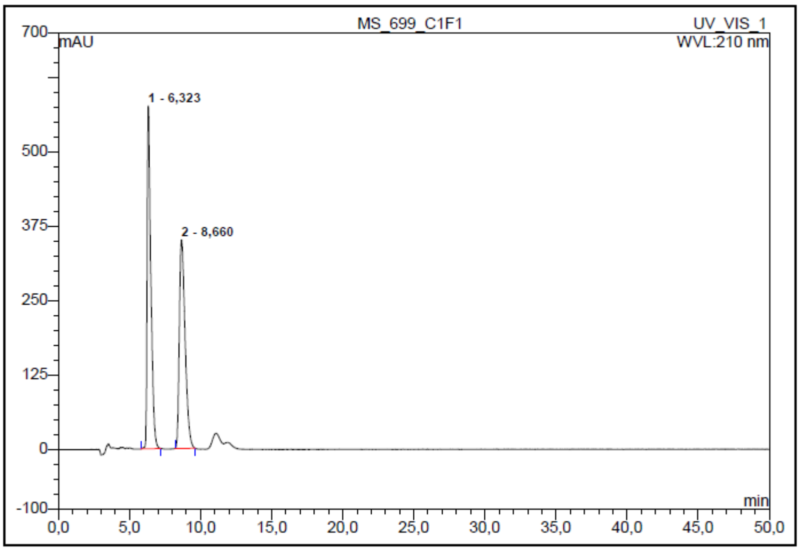

No.	Ret.Time min	Peak Name	Height mAU	Area mAU*min	Rel.Area %	Amount	Type
1	6,32	n.a.	575,702	174,202	51,26	n.a.	BMB
2	8,66	n.a.	350,345	165,617	48,74	n.a.	BMB
Total:			926,047	339,819	100,00	0,000	

*Enantioenriched Product **6x***

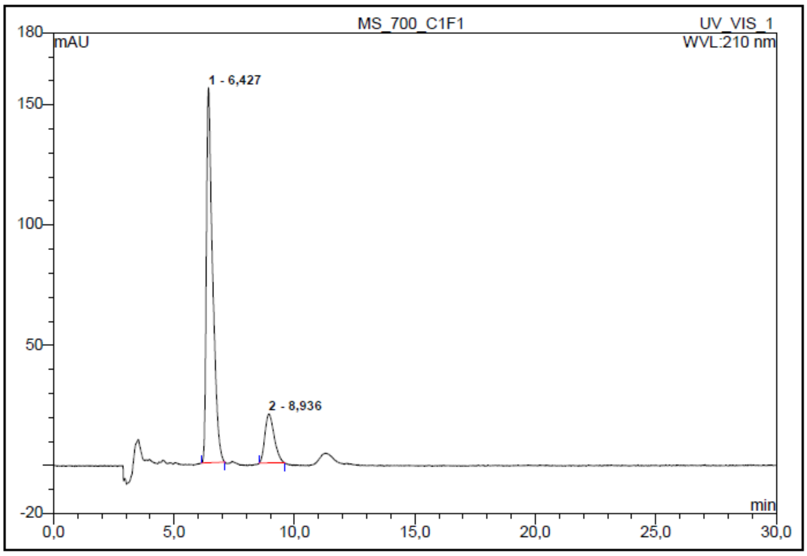

No.	Ret.Time min	Peak Name	Height mAU	Area mAU*min	Rel.Area %	Amount	Type
1	6,43	n.a.	155,953	46,913	83,79	n.a.	BMB
2	8,94	n.a.	20,360	9,075	16,21	n.a.	BMB*
Total:			176,313	55,988	100,00	0,000	

(*R*)-2-Benzyl-5,5-dimethylhexa-2,3-dienamide (6c)

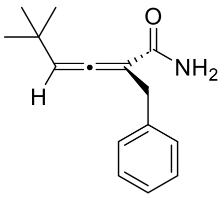

*Racemic Product rac-**6c***

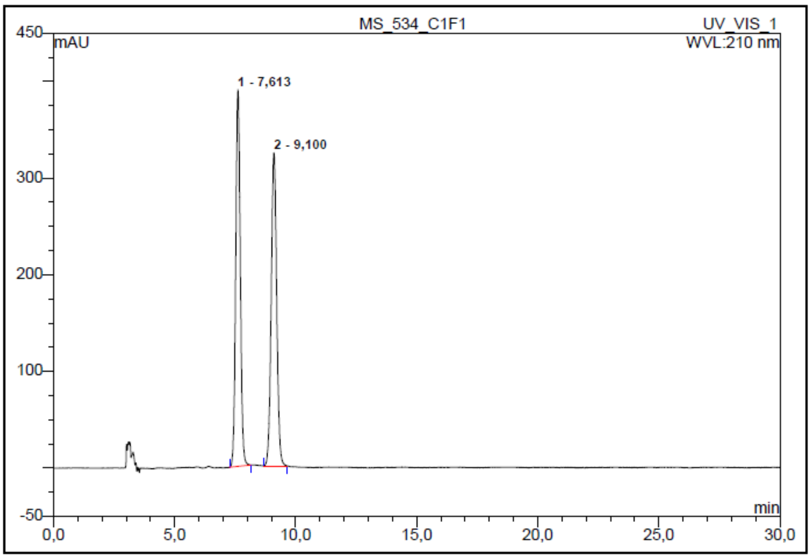

No.	Ret.Time min		Peak Name	Height mAU	Area mAU*min	Rel.Area %	Amount	Type
1	7,61	n.a.		389,658	85,200	50,11	n.a.	BMB
2	9,10	n.a.		323,808	84,821	49,89	n.a.	BMB
Total:				713,466	170,020	100,00	0,000	

*Enantioenriched Product **6c***

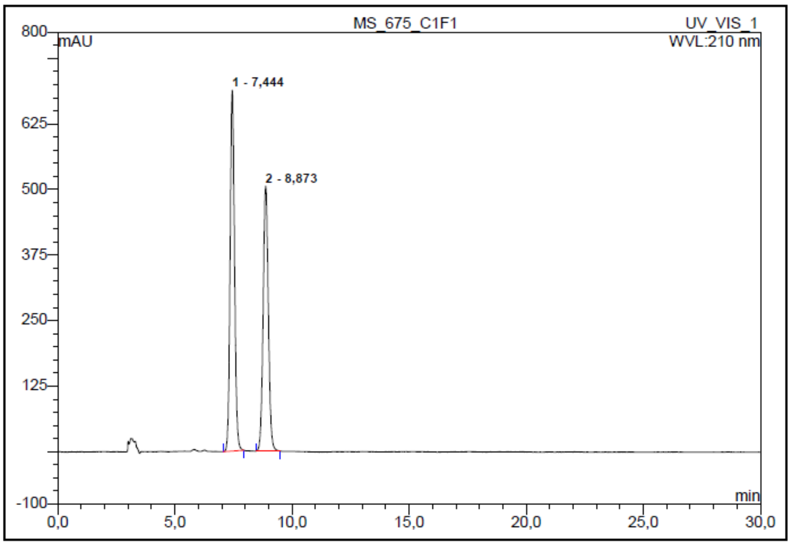

No.	Ret.Time min		Peak Name	Height mAU	Area mAU*min	Rel.Area %	Amount	Type
1	7,44	n.a.		688,320	150,269	53,73	n.a.	BMB
2	8,87	n.a.		505,137	129,386	46,27	n.a.	BMB
Total:				1193,457	279,655	100,00	0,000	

(*R*)-4-Bromo-5-(*tert*-butyl)-3-(4-iodobenzyl)furan-2(5*H*)-one (8)

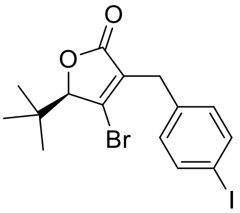

*Racemic Product rac-**8***

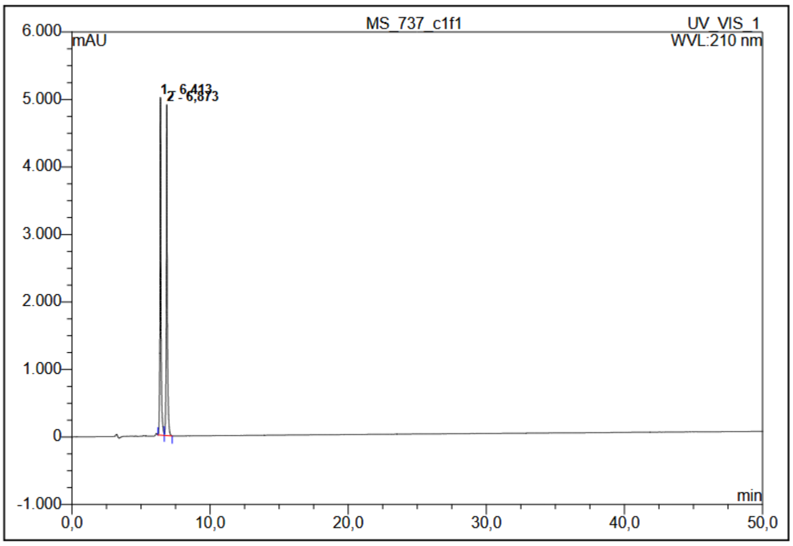

No.	Ret.Time min	Peak Name	Height mAU	Area mAU*min	Rel.Area %	Amount	Type
1	6,41	n.a.	5006,734	424,034	52,28	n.a.	BM
2	6,87	n.a.	4904,190	387,119	47,72	n.a.	MB
Total:			9910,924	811,153	100,00	0,000	

*Enantioenriched Product **8***

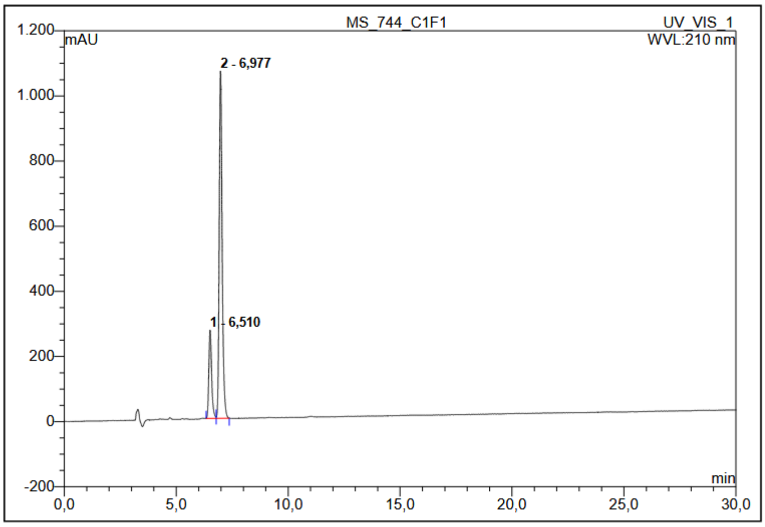

No.	Ret.Time min	Peak Name	Height mAU	Area mAU*min	Rel.Area %	Amount	Type
1	6,51	n.a.	271,100	39,360	19,41	n.a.	BM
2	6,98	n.a.	1066,808	163,440	80,59	n.a.	MB
Total:			1337,909	202,800	100,00	0,000	

(*R*)-5-(*tert*-Butyl)-4-iodo-3-(4-iodobenzyl)furan-2(5*H*)-one (9)

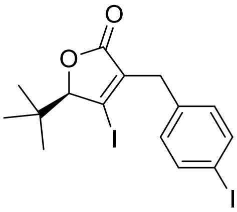

*Racemic Product rac-**9***

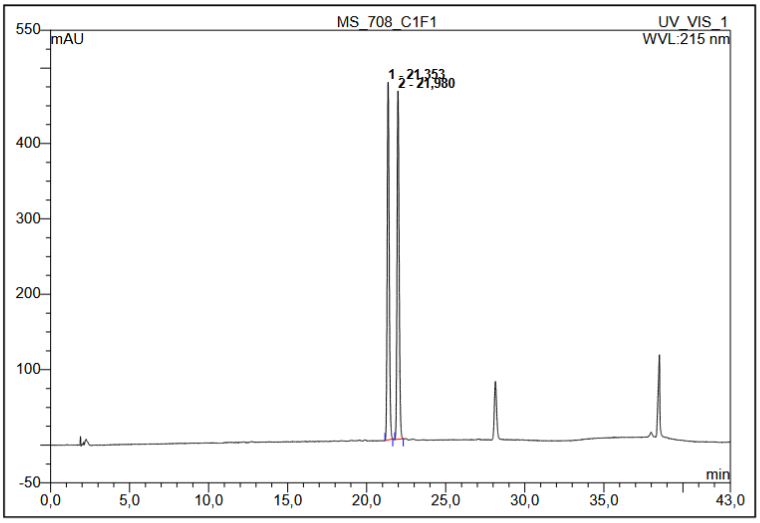

No.	Ret.Time min	Peak Name	Height mAU	Area mAU*min	Rel.Area %	Amount	Type
1	21,35	n.a.	474,422	69,470	49,96	n.a.	BMB
2	21,98	n.a.	462,028	69,593	50,04	n.a.	BMB
Total:			936,450	139,063	100,00	0,000	

*Enantioenriched Product **9***

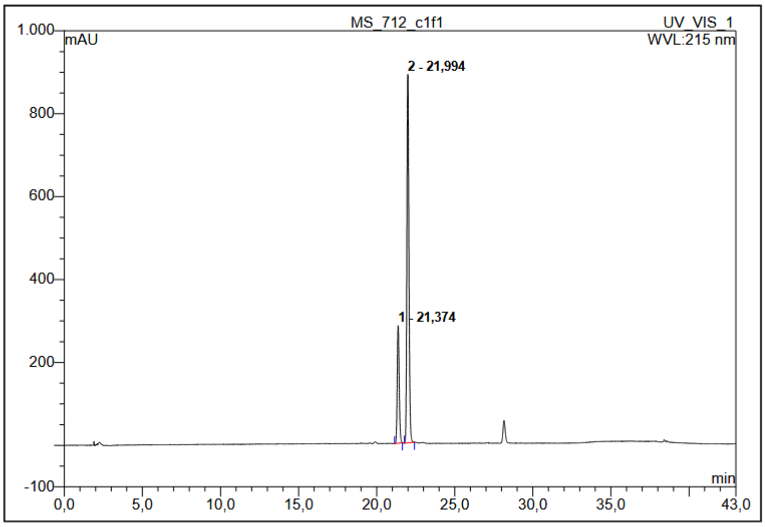

No.	Ret.Time min	Peak Name	Height mAU	Area mAU*min	Rel.Area %	Amount	Type
1	21,37	n.a.	284,079	41,604	23,47	n.a.	BMB
2	21,99	n.a.	888,803	135,634	76,53	n.a.	BMB
Total:			1172,882	177,238	100,00	0,000	

(*R*)-2-(*tert*-Butyl)-3-iodo-4-(4-iodobenzyl)-2,5-dihydrofuran (11)

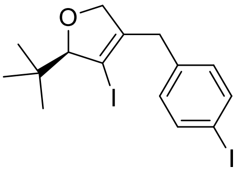

*Racemic Product rac-**11***

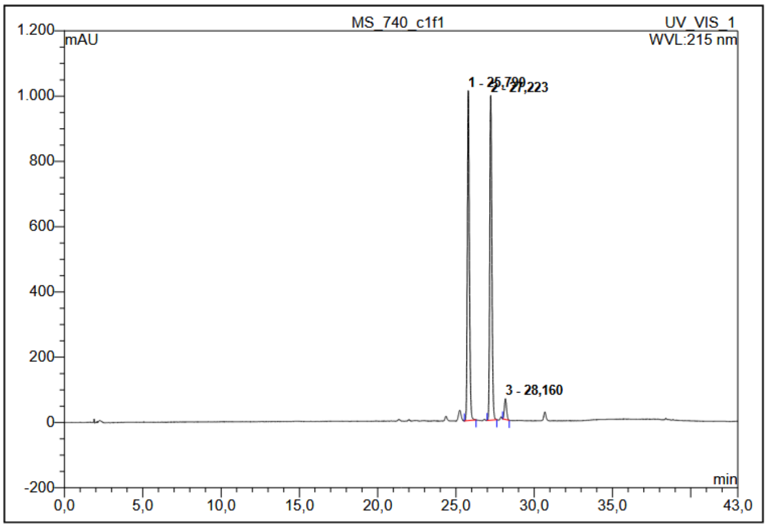

No.	Ret.Time min	Peak Name	Height mAU	Area mAU*min	Rel.Area %	Amount	Type
1	25,79	n.a.	1011,213	154,805	48,49	n.a.	BMB
2	27,22	n.a.	995,544	154,149	48,28	n.a.	BMB
3	28,16	n.a.	64,306	10,297	3,23	n.a.	BMB
Total:			2071,064	319,251	100,00	0,000	

*Enantioenriched Product **11***

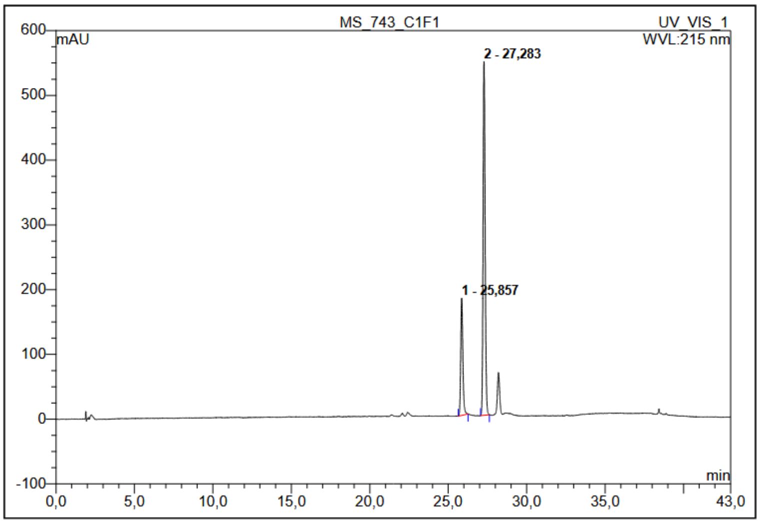

No.	Ret.Time min	Peak Name	Height mAU	Area mAU*min	Rel.Area %	Amount	Type
1	25,86	n.a.	181,446	28,318	25,33	n.a.	BMB
2	27,28	n.a.	546,751	83,487	74,67	n.a.	BMB
Total:			728,197	111,804	100,00	0,000	

(*R*)-*tert*-Butyl((2-(4-iodobenzyl)-5,5-dimethylhexa-2,3-dien-1-yl)oxy)dimethylsilane (12)

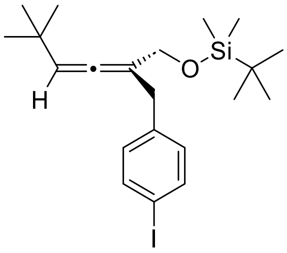

*Racemic Product rac-**12***

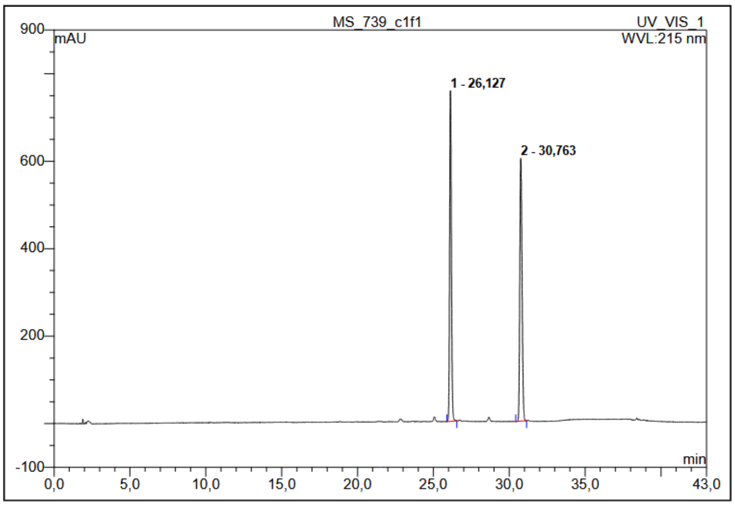

No.	Ret.Time min	Peak Name	Height mAU	Area mAU*min	Rel.Area %	Amount	Type
1	26,13	n.a.	756,508	101,730	49,94	n.a.	BMB
2	30,76	n.a.	601,328	101,977	50,06	n.a.	BMB*
Total:			1357,836	203,708	100,00	0,000	

*Enantioenriched Product **12***

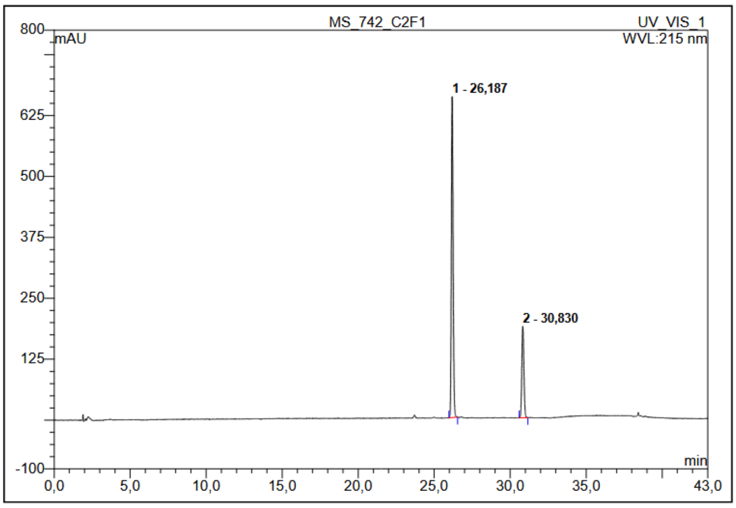

No.	Ret.Time min	Peak Name	Height mAU	Area mAU*min	Rel.Area %	Amount	Type
1	26,19	n.a.	659,070	88,415	74,88	n.a.	BMB
2	30,83	n.a.	187,674	29,657	25,12	n.a.	BMB*
Total:			846,743	118,072	100,00	0,000	

(*R*)-2-Benzyl-4-phenylbuta-2,3-dienoic acid (*rac*-SI-1)

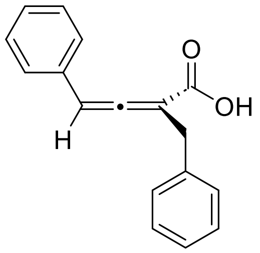

*Racemic Product rac-**SI-1***

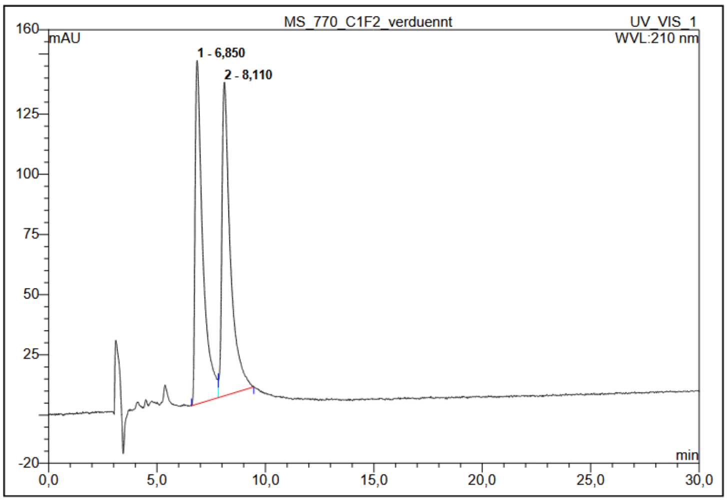

No.	Ret.Time min	Peak Name	Height mAU	Area mAU*min	Rel.Area %	Amount	Type
1	6,85	n.a.	142,647	58,790	49,95	n.a.	BM *
2	8,11	n.a.	130,229	58,915	50,05	n.a.	MB*
Total:			272,876	117,705	100,00	0,000	

*Enantioenriched Product **SI-1***

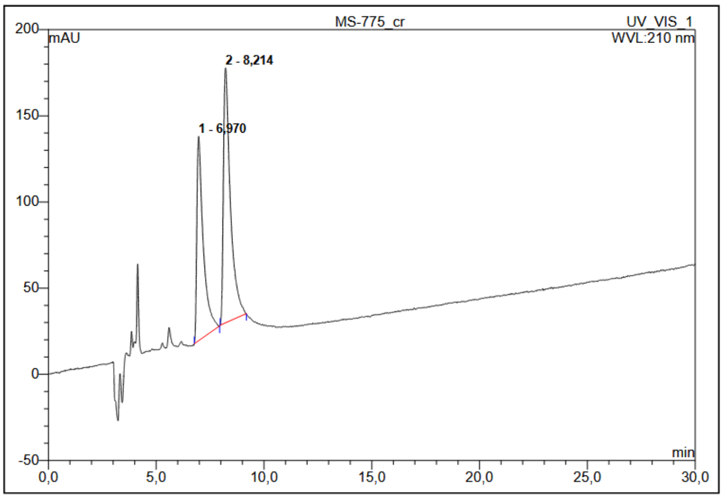

No.	Ret.Time min	Peak Name	Height mAU	Area mAU*min	Rel.Area %	Amount	Type
1	6,97	n.a.	118,529	41,334	42,63	n.a.	BMB*
2	8,21	n.a.	147,966	55,625	57,37	n.a.	BMB*
Total:			266,495	96,960	100,00	0,000	

5,5-Dimethyl-2-phenylhexa-2,3-dienoic acid (*rac*-SI-2)

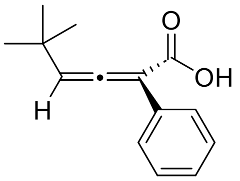

*Racemic Product rac-**SI-2***

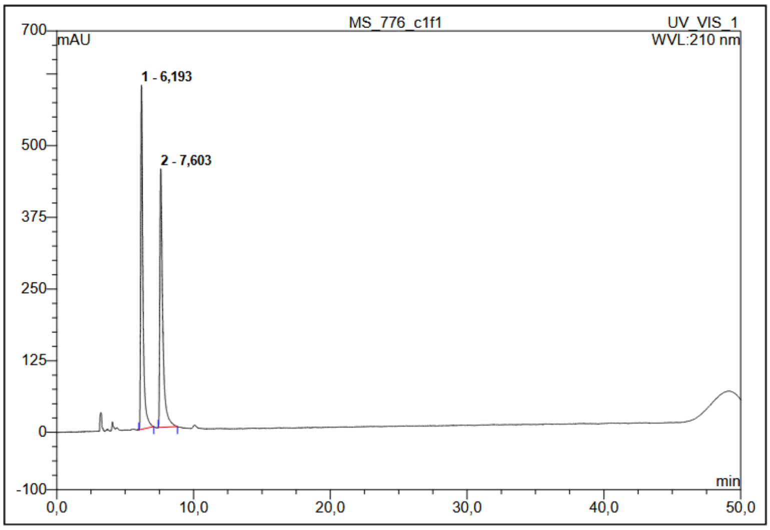

No.	Ret.Time min	Peak Name	Height mAU	Area mAU*min	Rel.Area %	Amount	Type
1	6,19	n.a.	600,517	113,354	50,74	n.a.	BMB*
2	7,60	n.a.	450,754	110,060	49,26	n.a.	BMB*
Total:			1051,271	223,415	100,00	0,000	

*Enantioenriched Product **SI-2***

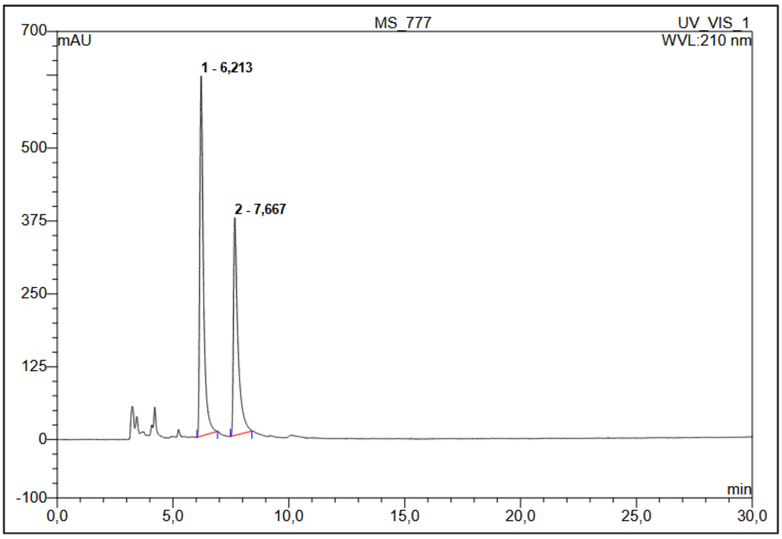

No.	Ret.Time min	Peak Name	Height mAU	Area mAU*min	Rel.Area %	Amount	Type
1	6,21	n.a.	617,761	114,018	57,79	n.a.	BMB
2	7,67	n.a.	373,430	83,269	42,21	n.a.	BMB
Total:			991,191	197,286	100,00	0,000	

13. NMR Spectra

(*R*)-2-(12-Hydroxy-12-oxido-4,5,6,7-tetrahydrodiindeno[7,1-*de*:1',7'-*fg*][1,3,2]dioxaphosphocin-1-yl)-9*H*-thioxanthen-9-one (3e)

^{1}H NMR (500 MHz, DMSO-d_6, 300 K):

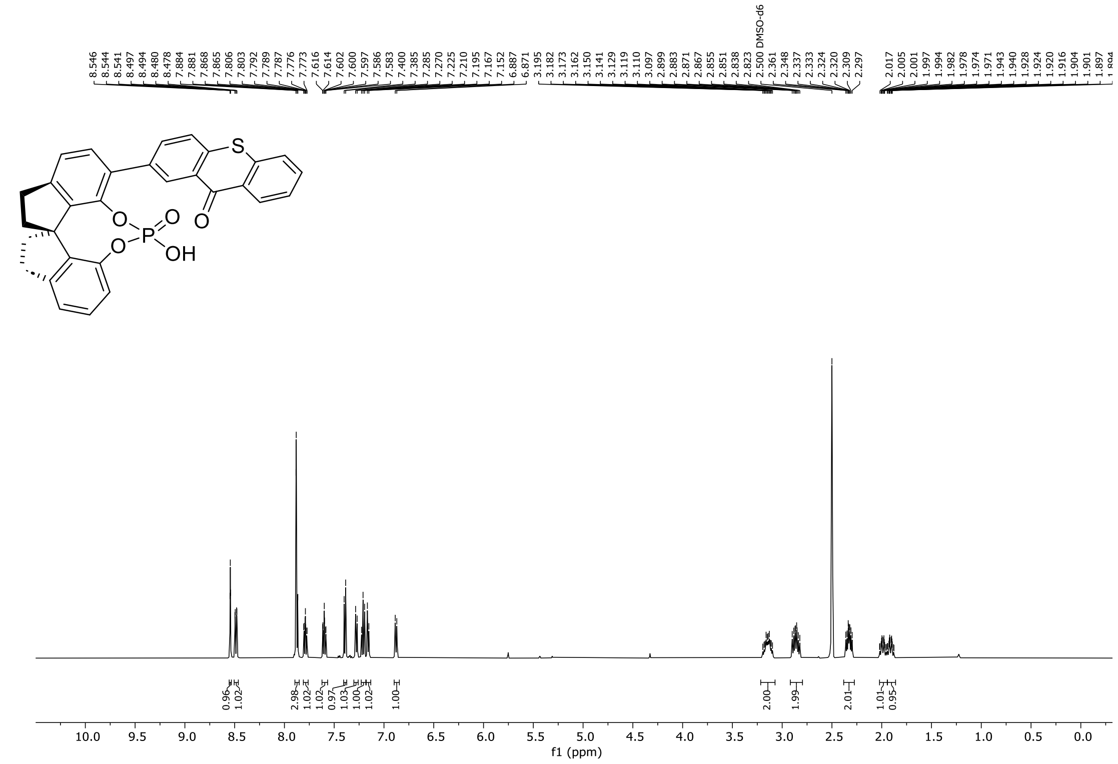

^{13}C NMR (126 MHz, DMSO-d_6, 300 K):

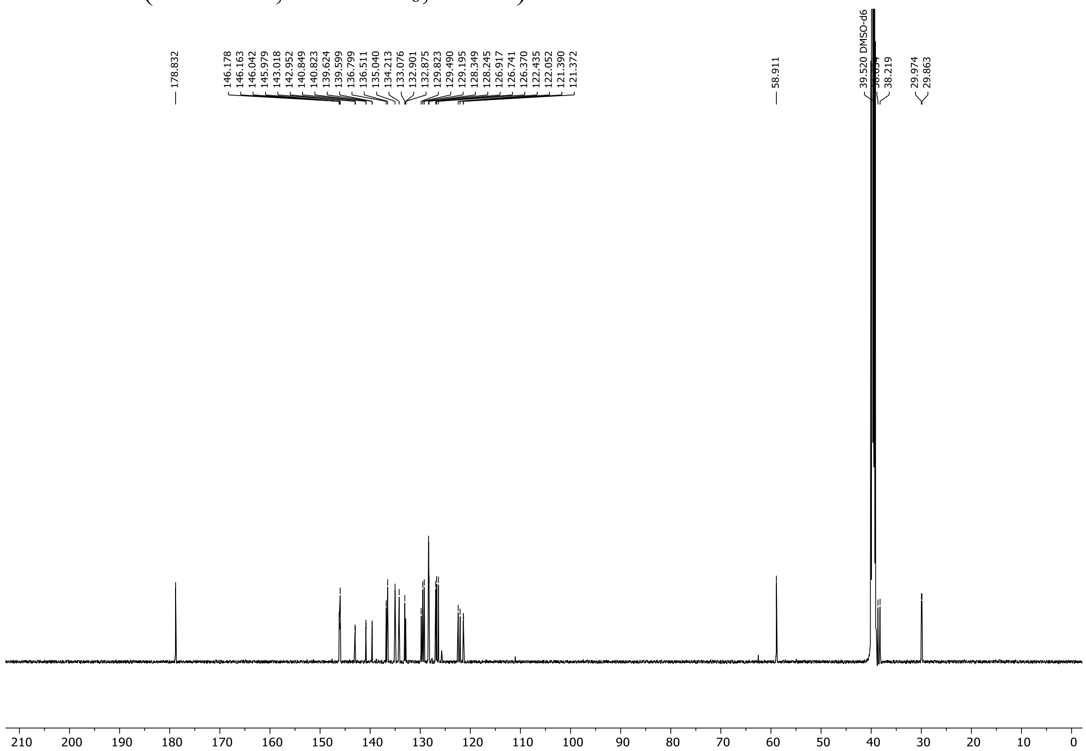

^{31}P NMR (203 MHz, DMSO-d_6, 300 K):

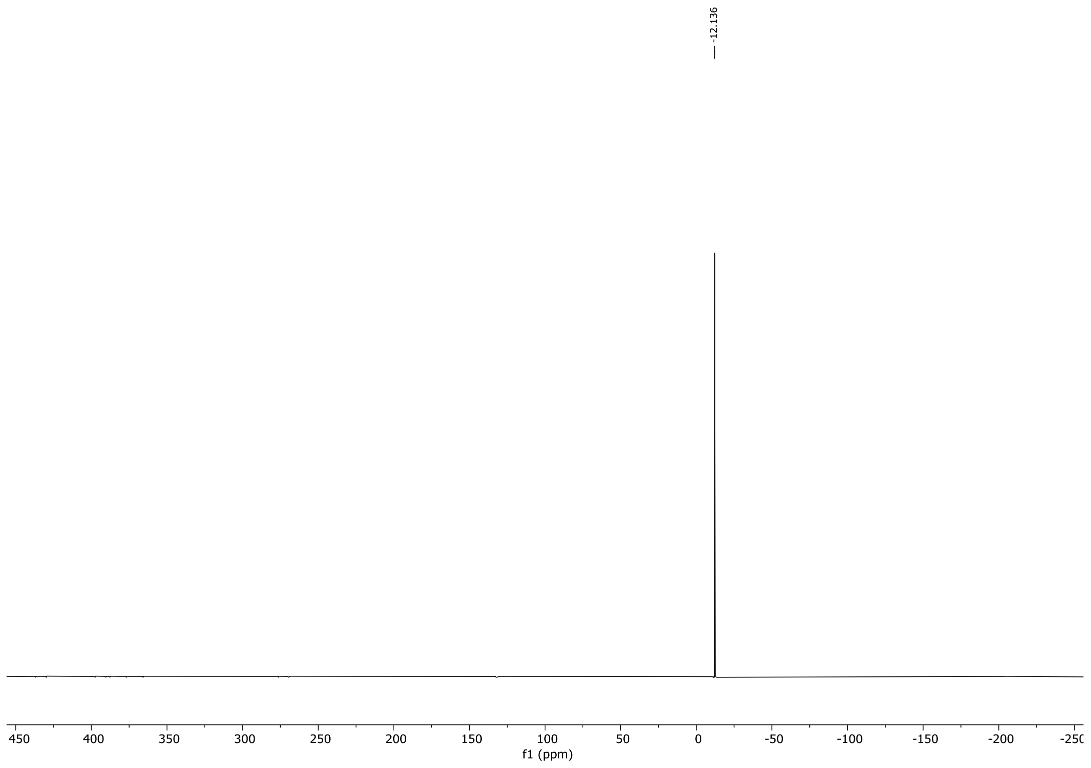

(*R*)-2,2'-(12-Hydroxy-12-oxido-4,5,6,7-tetrahydrodiindeno[7,1-de:1',7'-fg][1,3,2]dioxaphosphocine-1,10-diyl)bis(9H-thioxanthen-9-one) (3f)

^{1}H NMR (500 MHz, DMSO-d_6, 300 K):

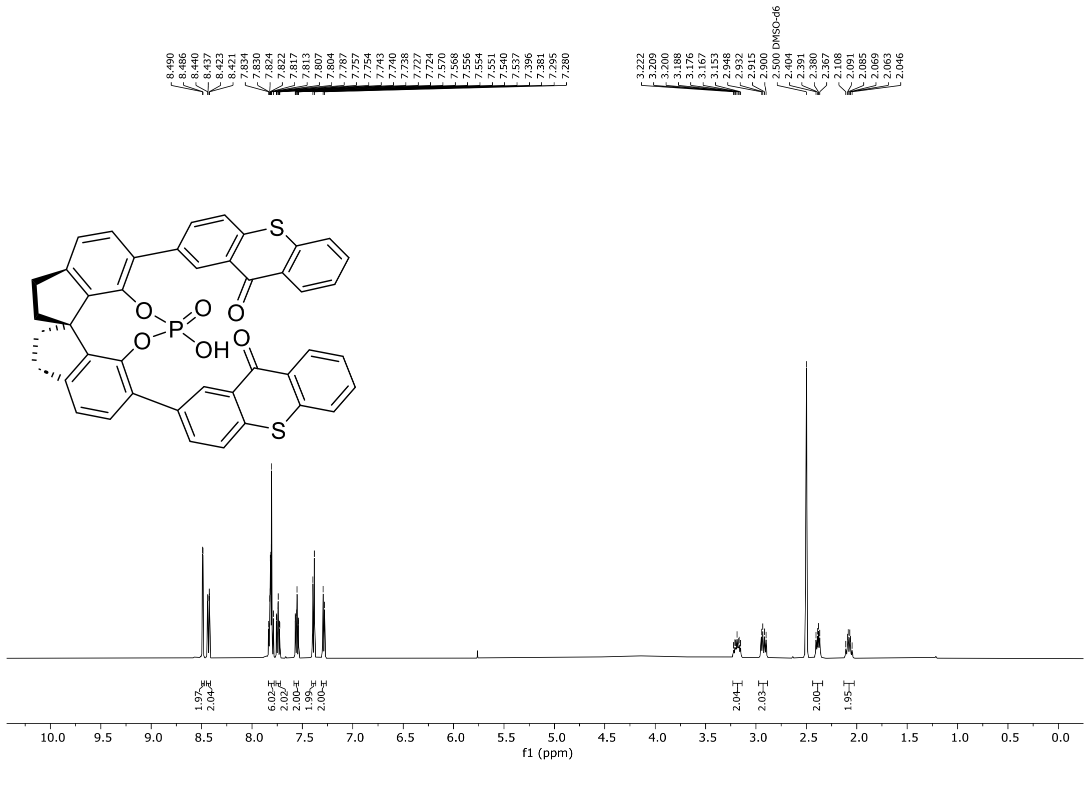

^{13}C NMR (126 MHz, DMSO-d_6, 300 K)

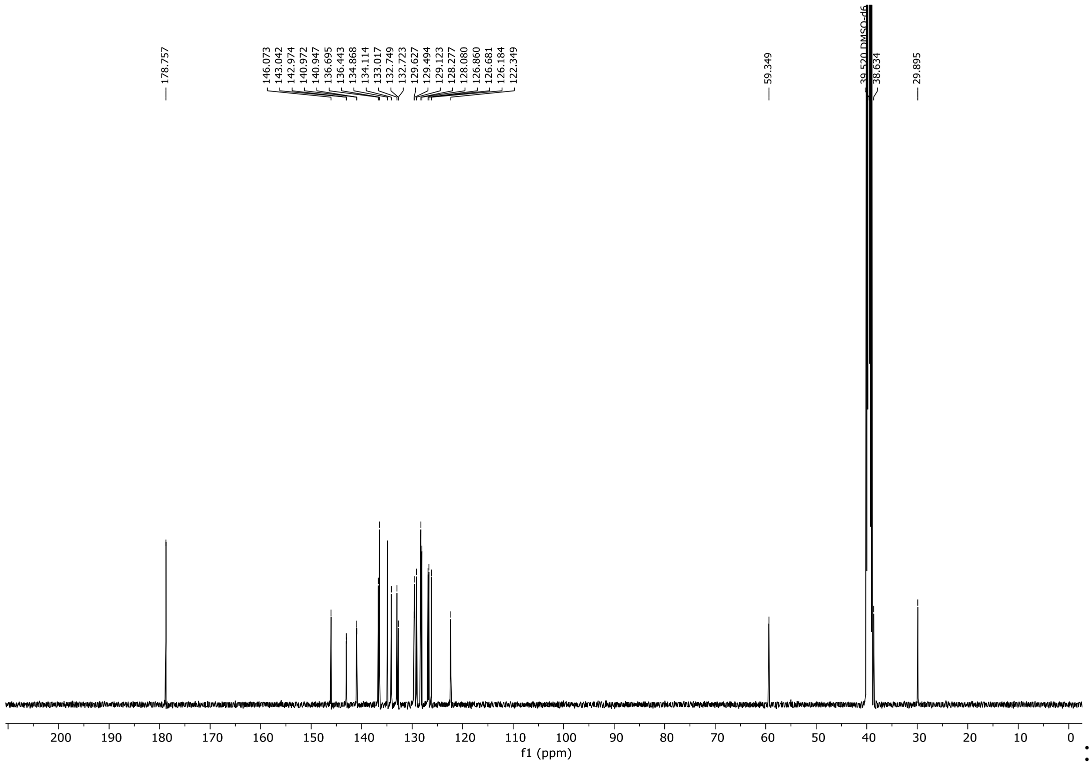

^{31}P NMR (162 MHz, DMSO-d_6, 300 K):

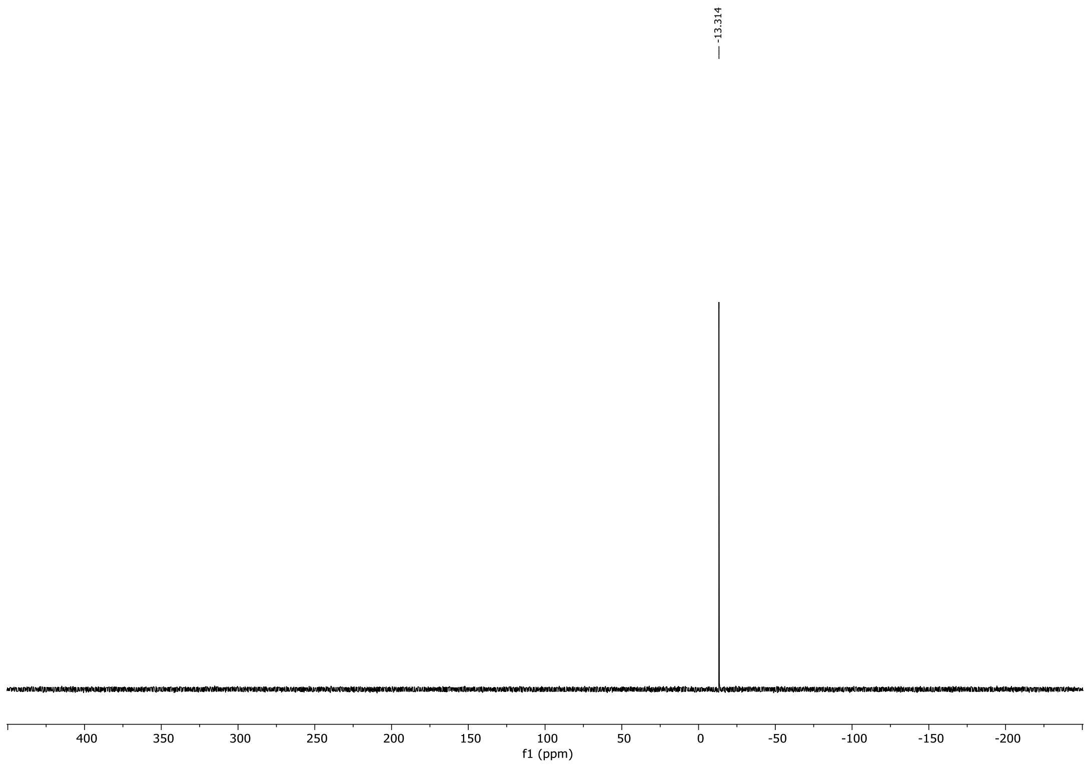

^{1}H NMR (400 MHz, DMSO-d_6, 300 K): *Spectrum of reisolated catalyst*

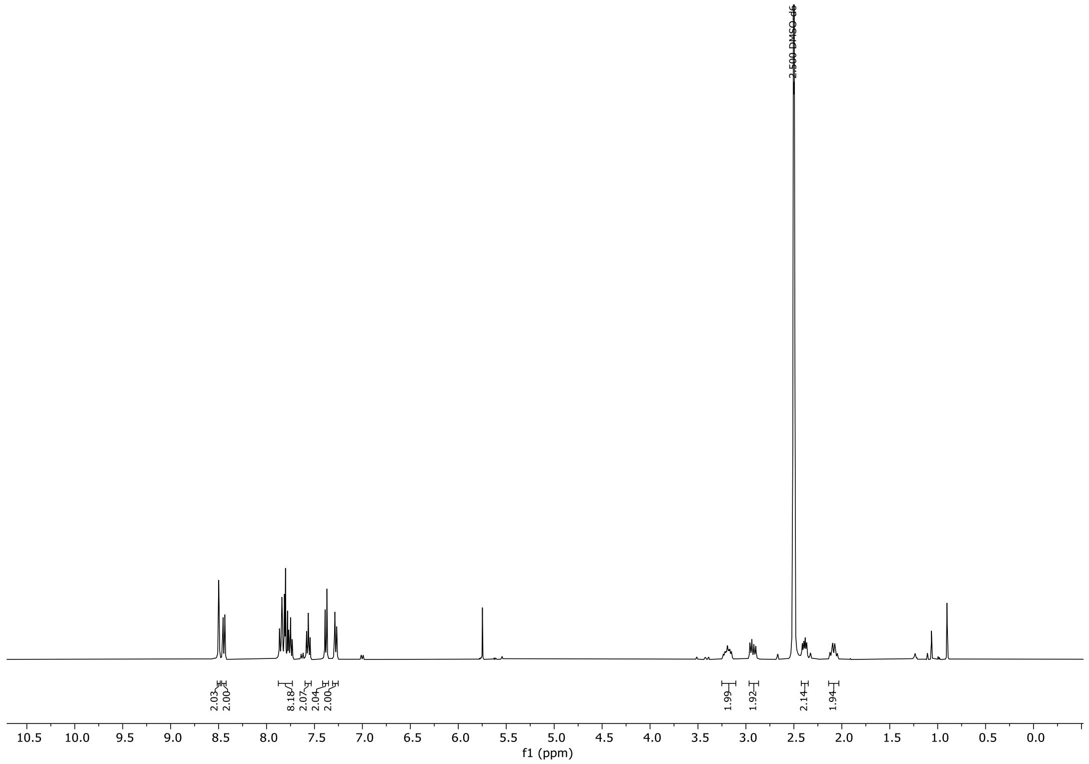

(*R*)-3,3'-(12-Hydroxy-12-oxido-4,5,6,7-tetrahydrodiindeno[7,1-de:1',7'-fg][1,3,2]dioxaphosphocine-1,10-diyl)bis(9H-thioxanthen-9-one) (3g)

^{1}H NMR (500 MHz, DMSO-d_6, 300 K):

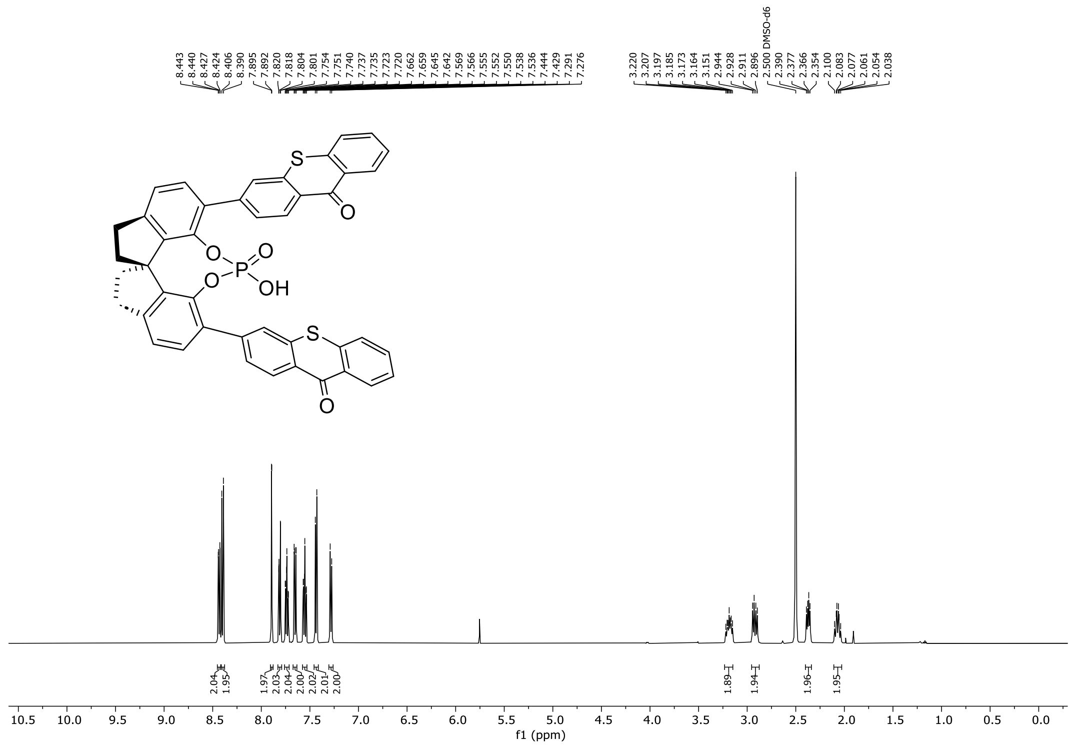

^{13}C NMR (126 MHz, DMSO-d_6, 300 K):

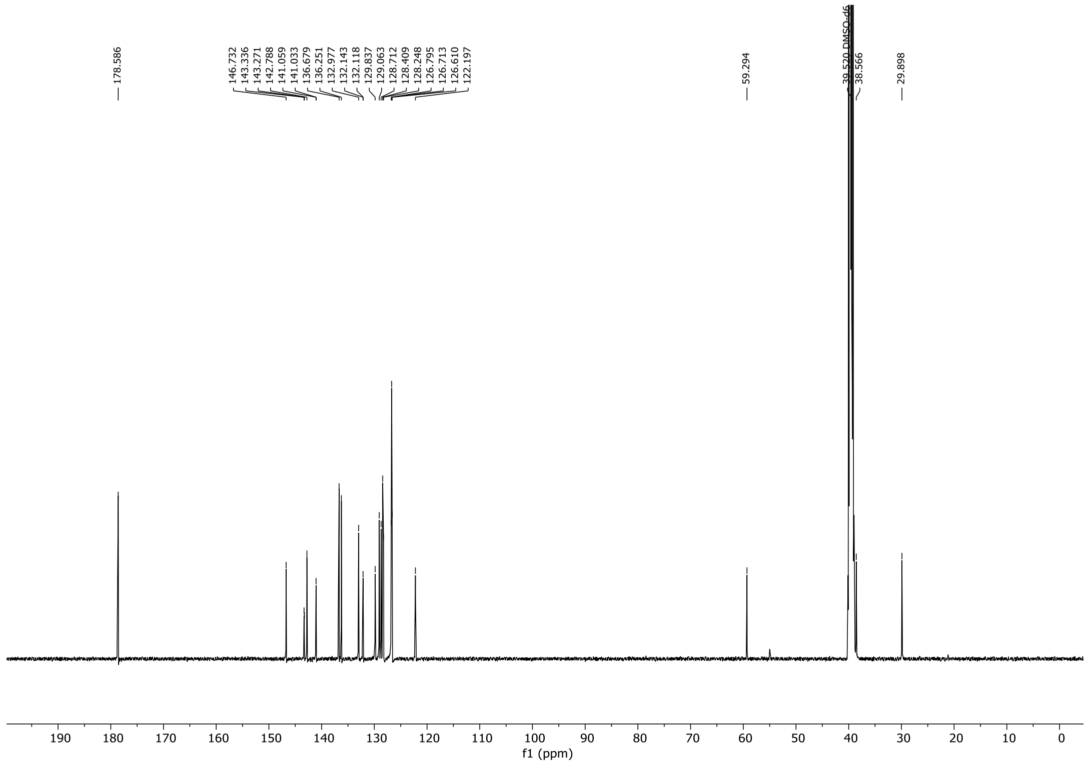

^{31}P NMR (203 MHz, DMSO-d_6, 300 K):

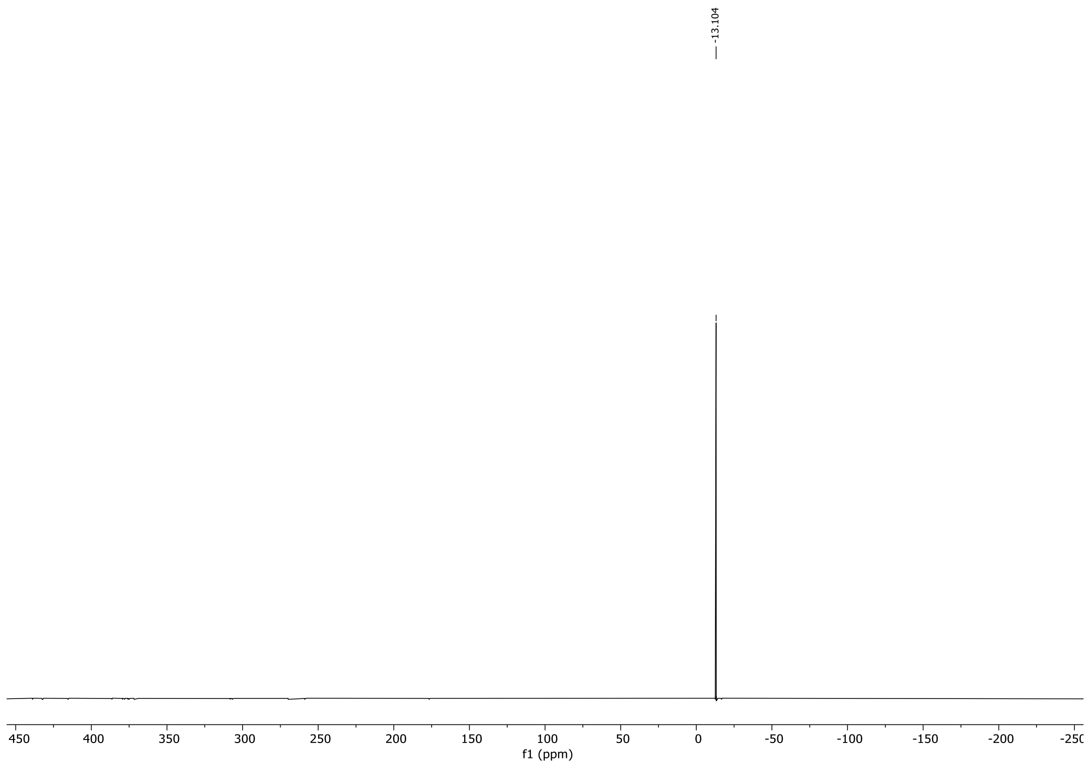

2-Benzyl-5,5-dimethylhexa-2,3-dienoic acid (*rac*-1c)

^{1}H NMR (400 MHz, $CDCl_3$, 300 K):

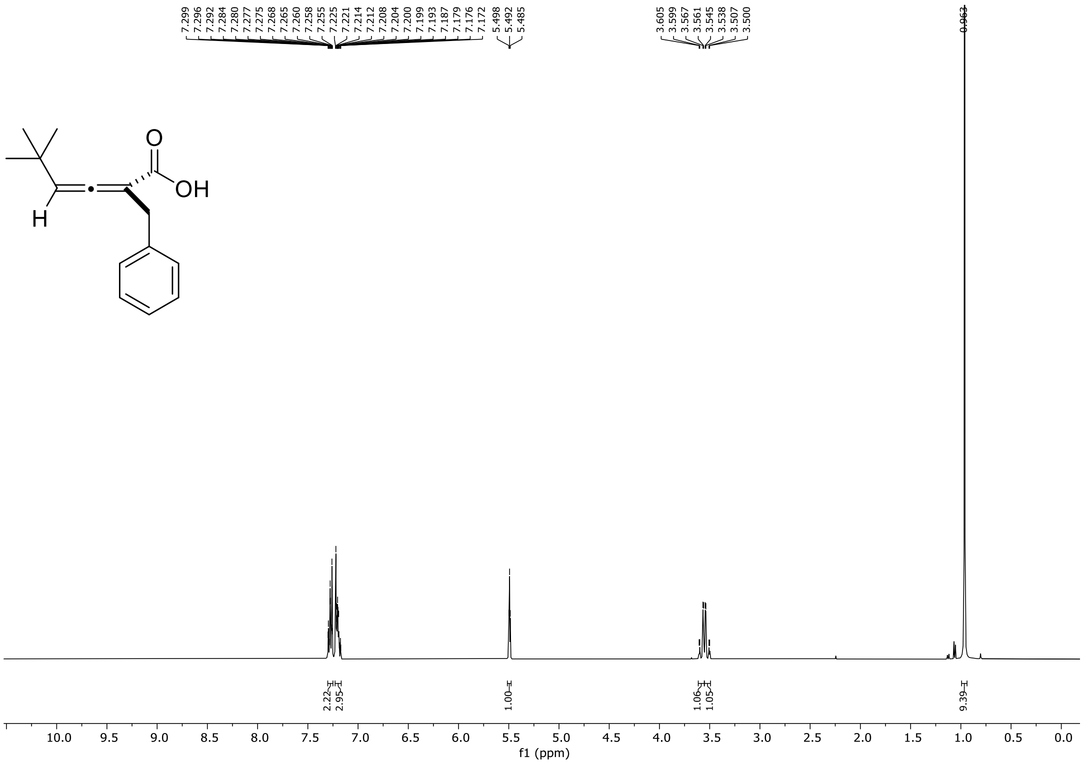

^{13}C NMR (101 MHz, $CDCl_3$, 300 K):

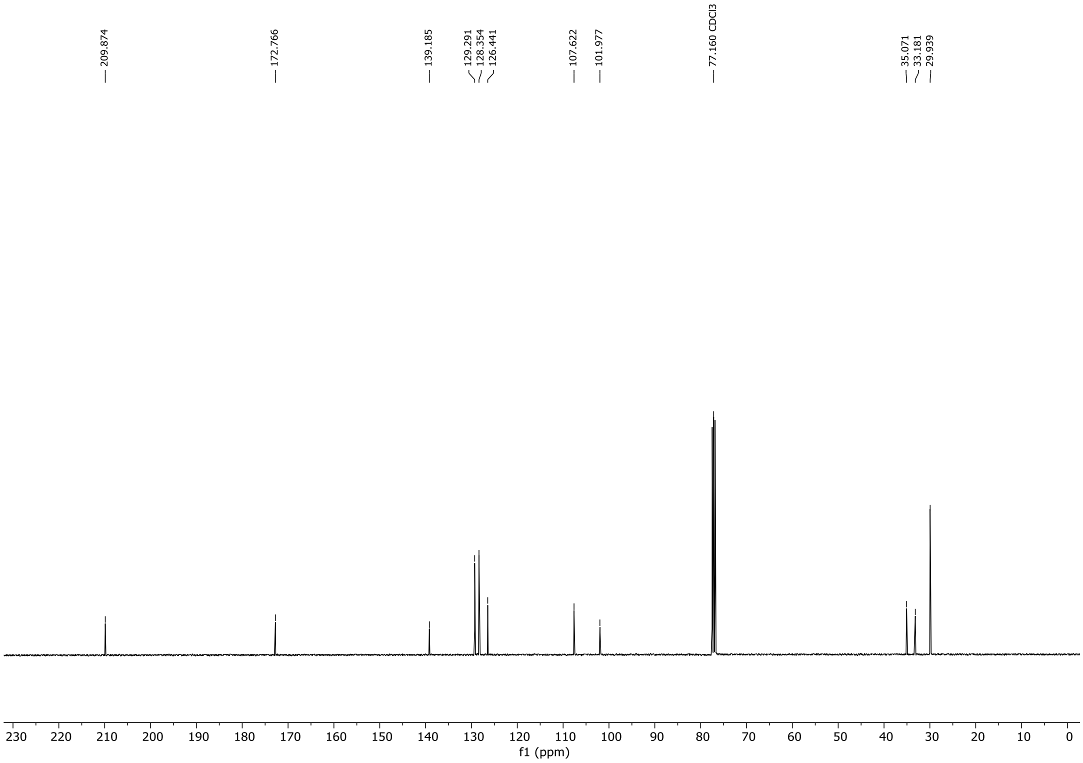

Methyl 2-benzyl-5,5-dimethylhexa-2,3-dienoate (*rac*-2c)

^{1}H NMR (400 MHz, $CDCl_3$, 300 K):

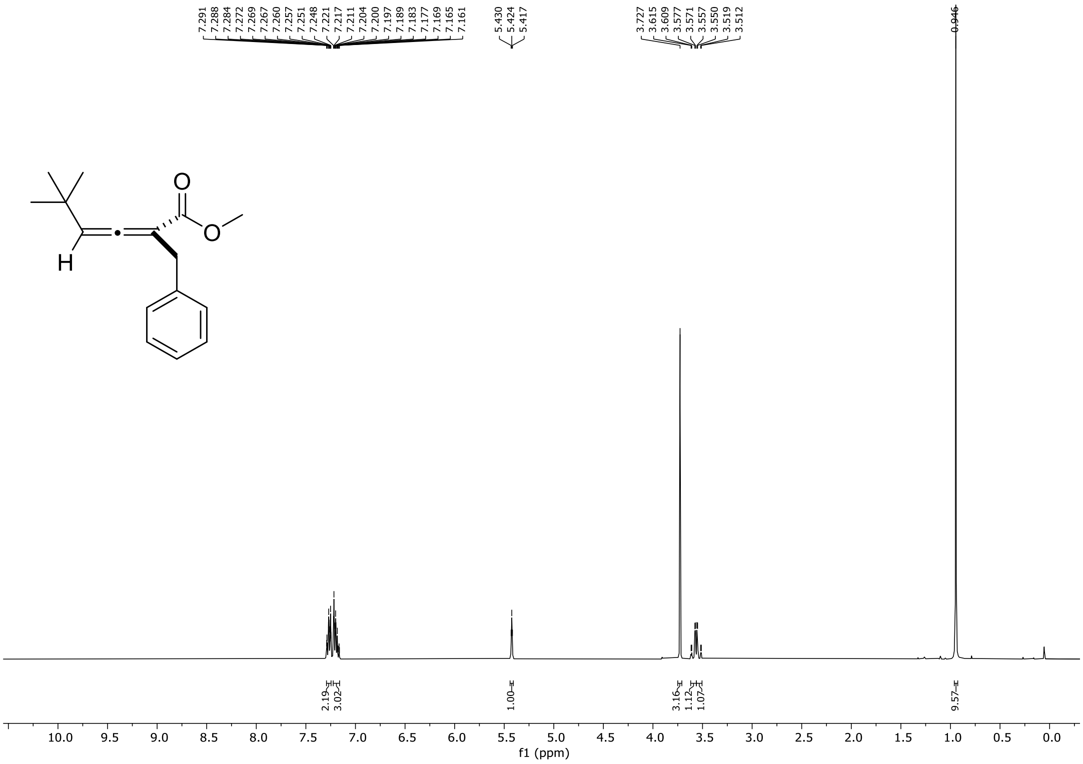

^{13}C NMR (101 MHz, $CDCl_3$, 300 K):

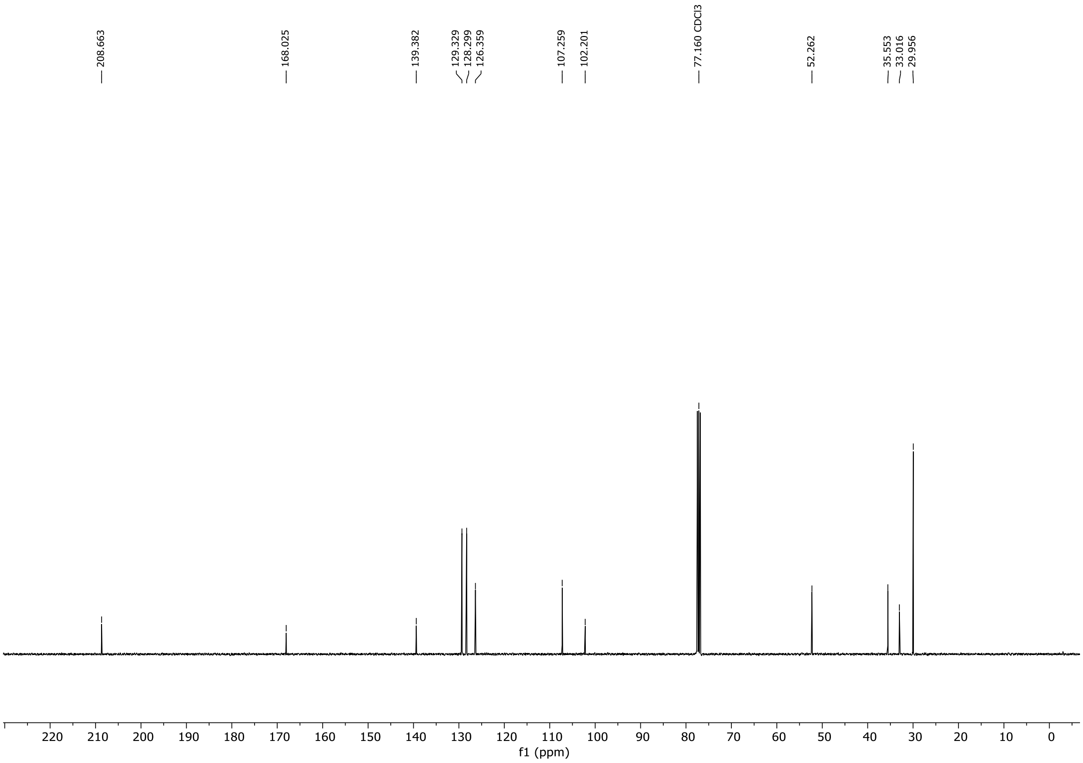

2-(4-Fluorobenzyl)-5,5-dimethylhexa-2,3-dienoic acid (*rac*-1d)

^{1}H NMR (400 MHz, $CDCl_3$, 300 K):

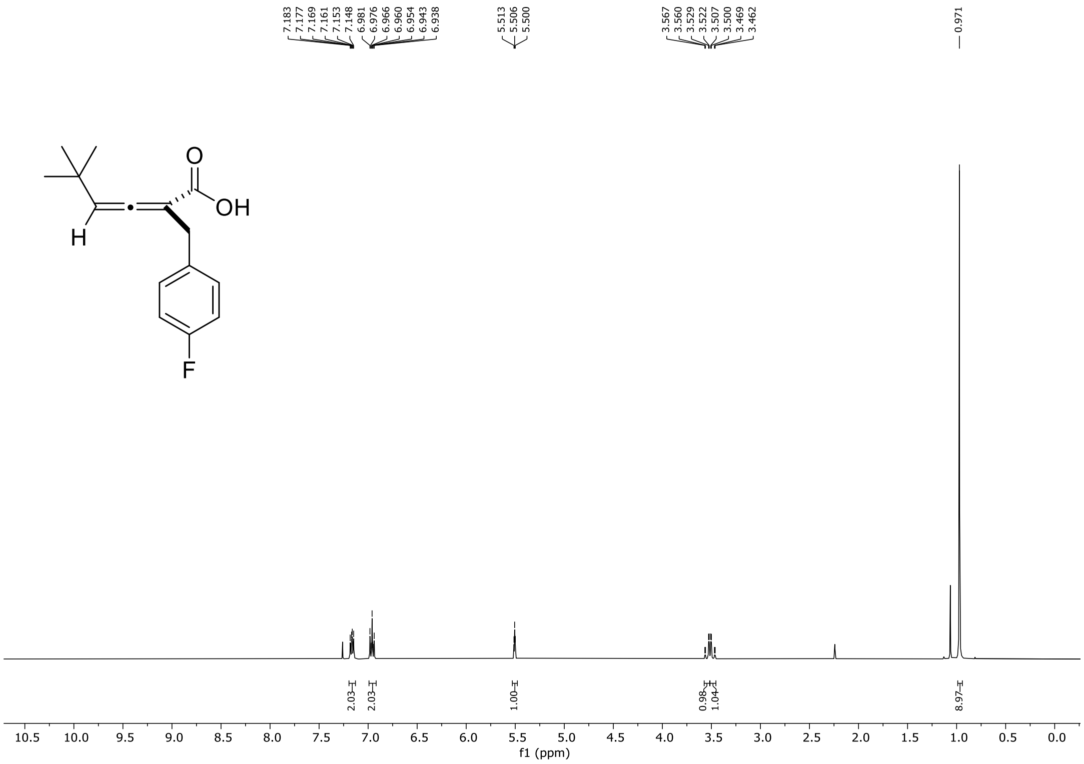

^{13}C NMR (101 MHz, $CDCl_3$, 300 K):

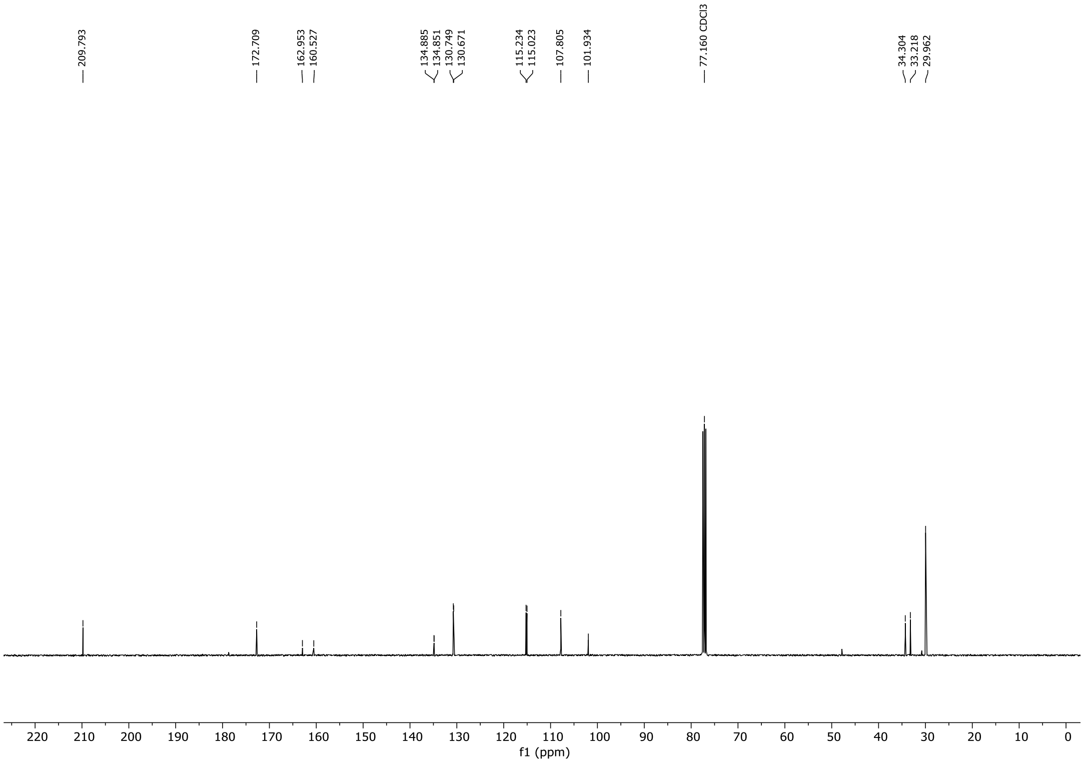

^{19}F NMR (376 MHz, $CDCl_3$, 300 K):

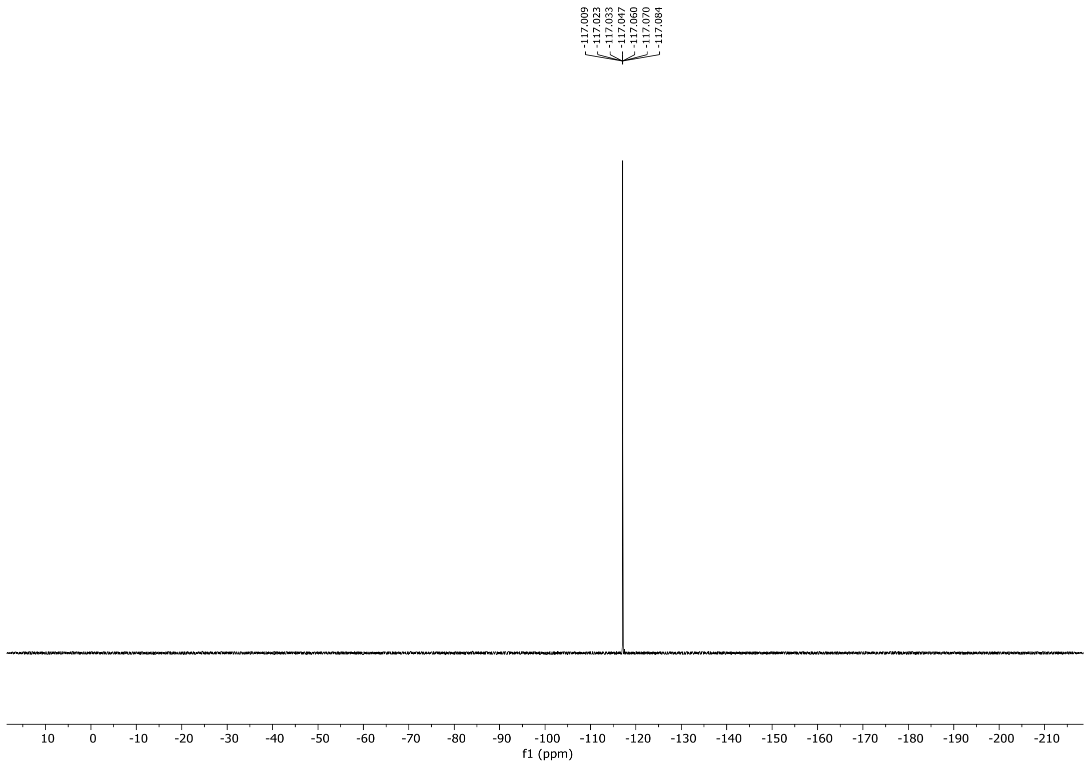

2-(4-Bromobenzyl)-5,5-dimethylhexa-2,3-dienoic acid (*rac*-1e)

^{1}H NMR (400 MHz, $CDCl_3$, 300 K):

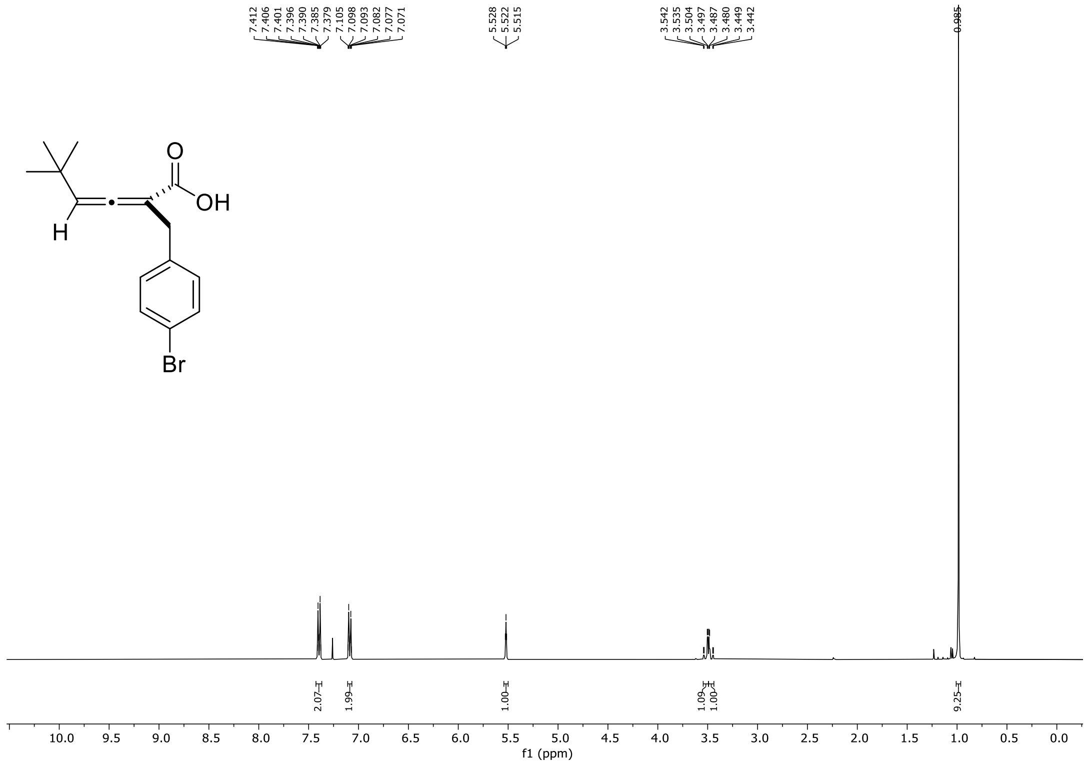

^{13}C NMR (101 MHz, $CDCl_3$, 300 K):

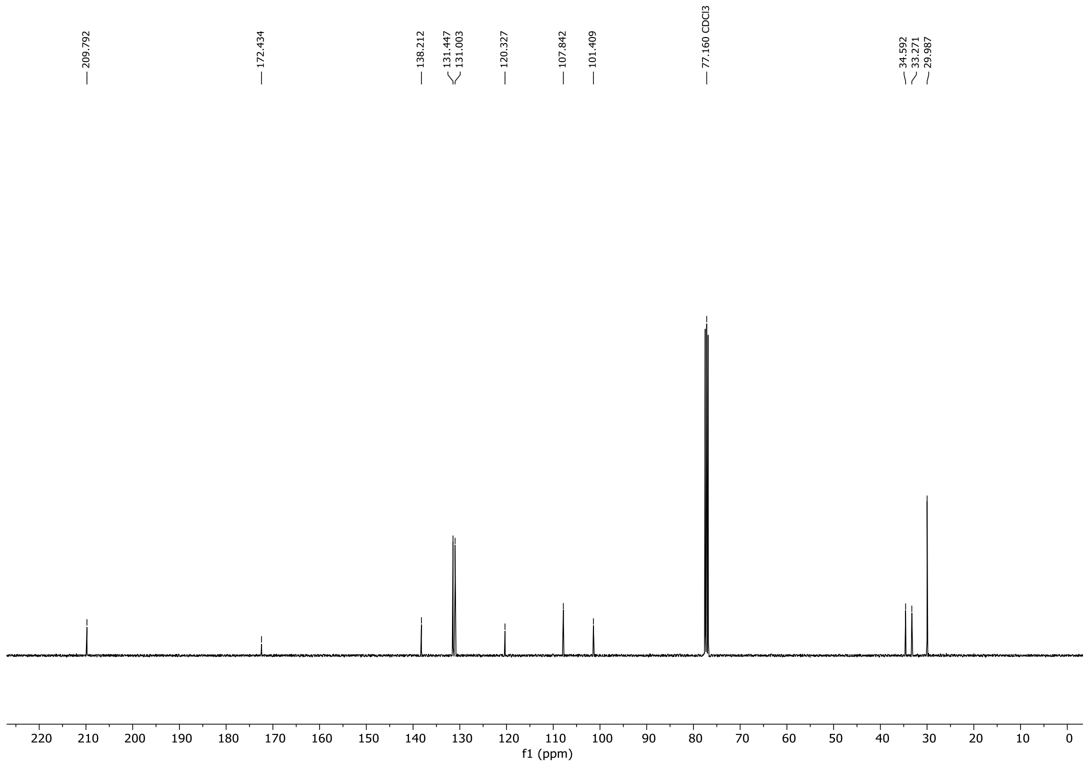

Methyl 2-(4-bromobenzyl)-5,5-dimethylhexa-2,3-dienoate (*rac*-2e)

^{1}H NMR (500 MHz, $CDCl_3$, 300 K):

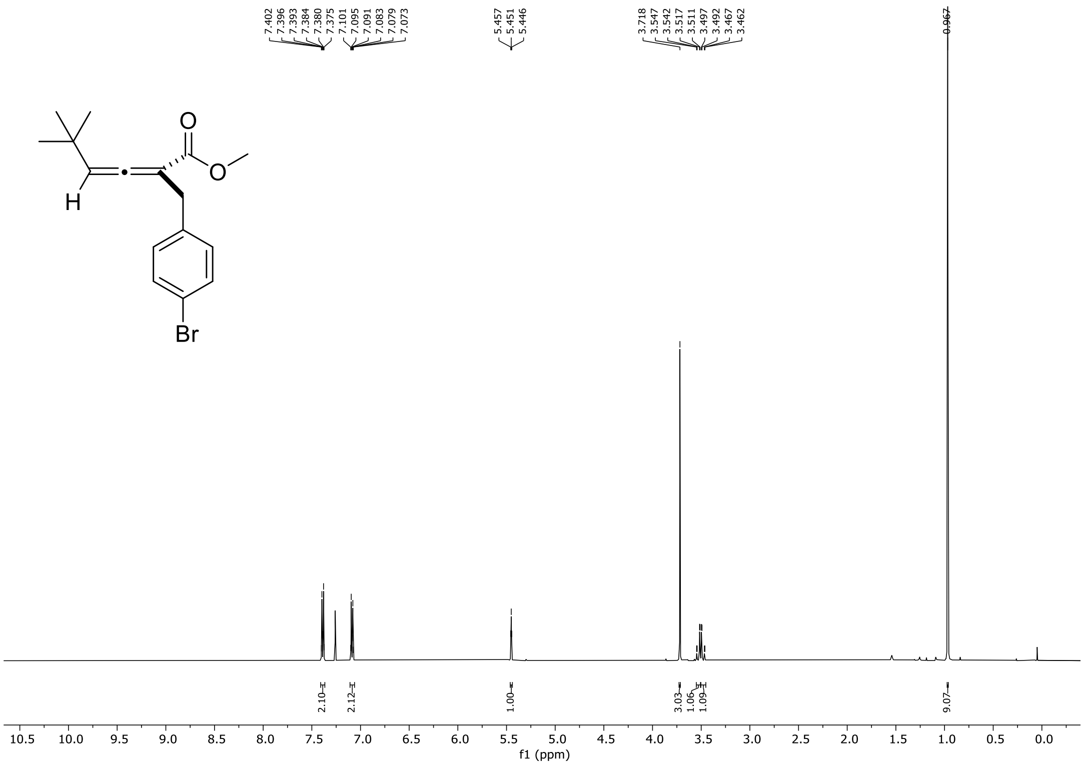

^{13}C NMR (101 MHz, $CDCl_3$, 300 K):

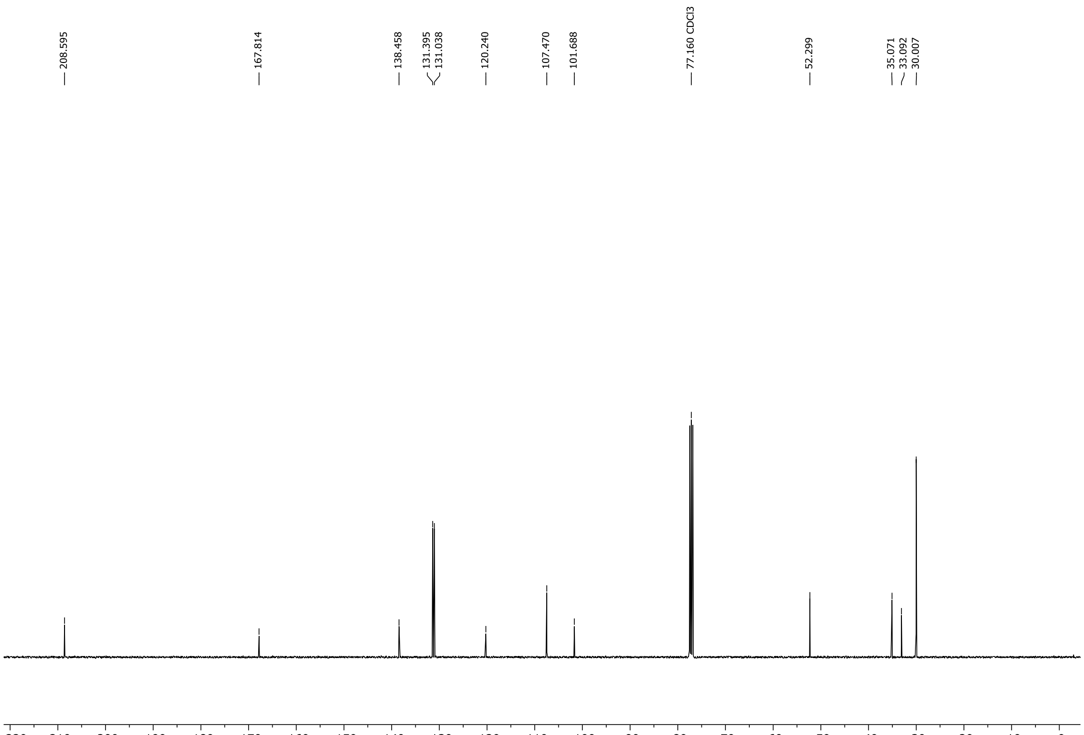

2-(4-Iodobenzyl)-5,5-dimethylhexa-2,3-dienoic acid (*rac*-1f)

^{1}H NMR (400 MHz, $CDCl_3$, 300 K):

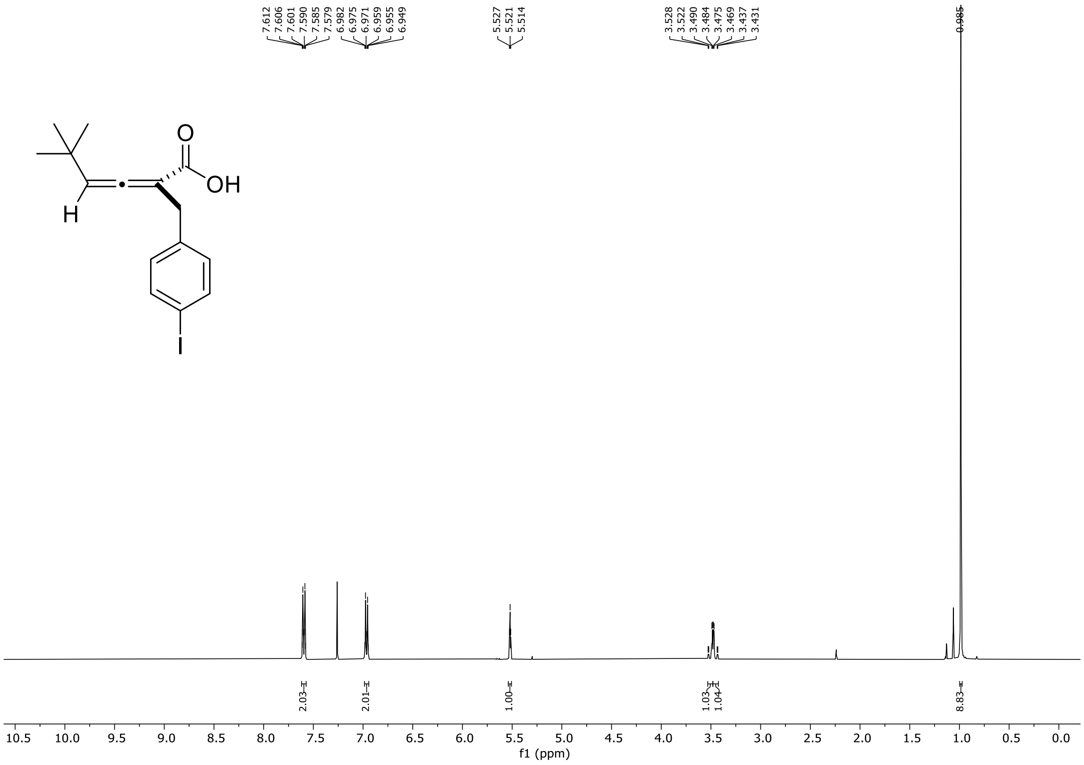

^{13}C NMR (101 MHz, $CDCl_3$, 300 K):

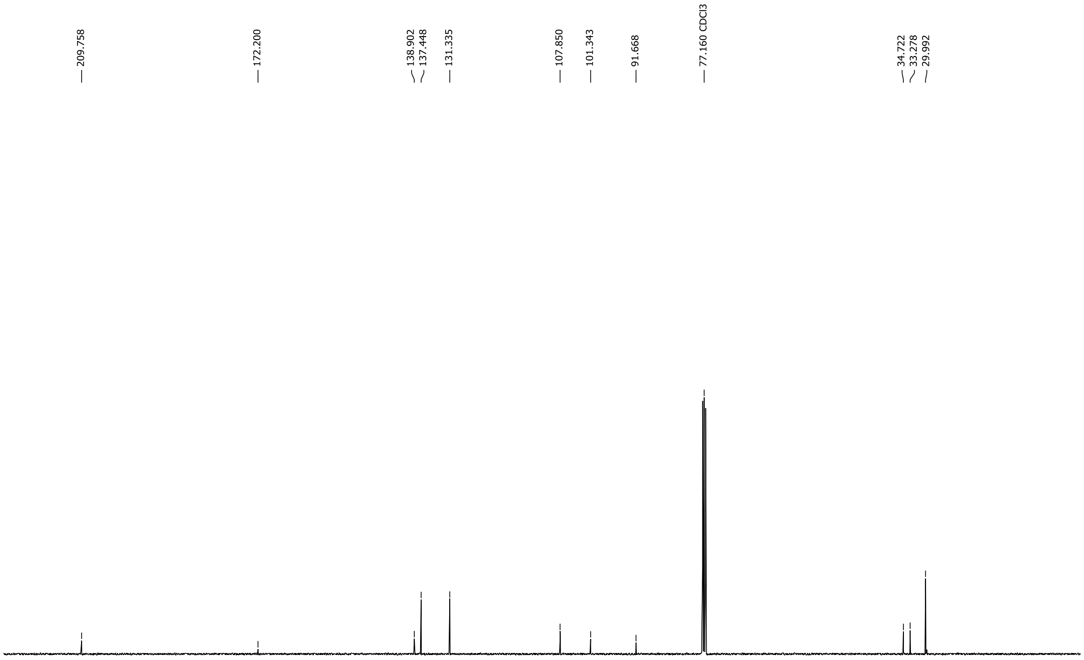

2-(4-(Tert-butyl)benzyl)-5,5-dimethylhexa-2,3-dienoic acid (*rac*-1g)

^{1}H NMR (400 MHz, $CDCl_3$, 300 K):

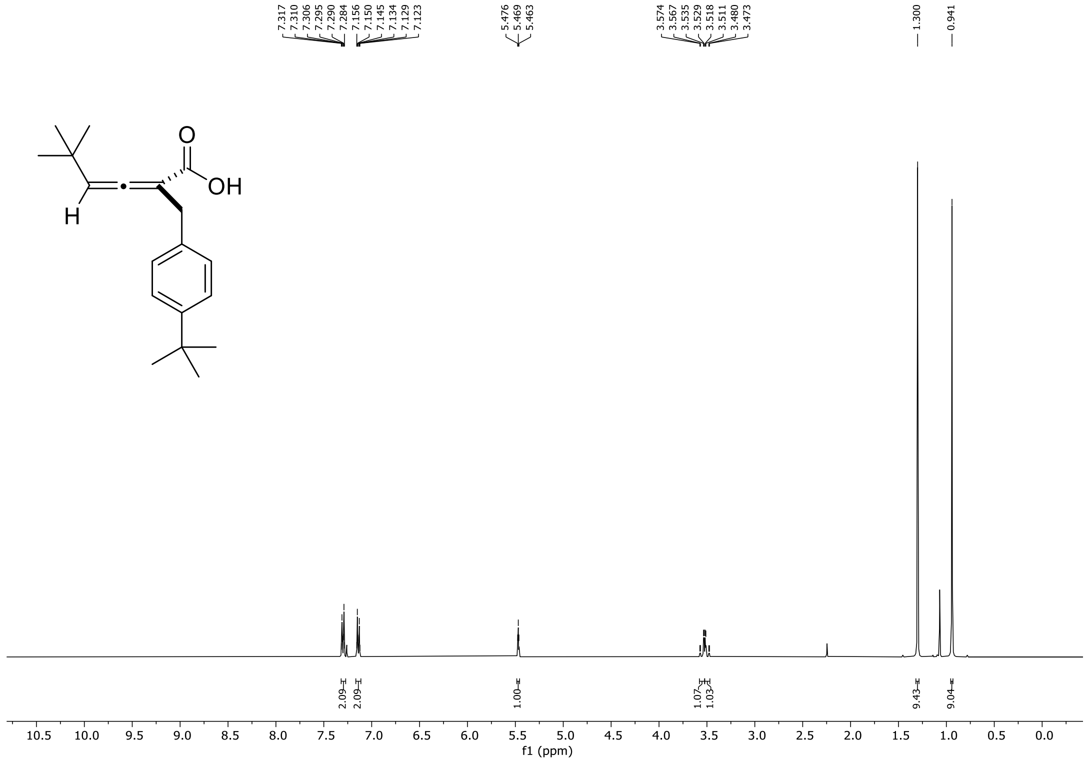

^{13}C NMR (101 MHz, $CDCl_3$, 300 K):

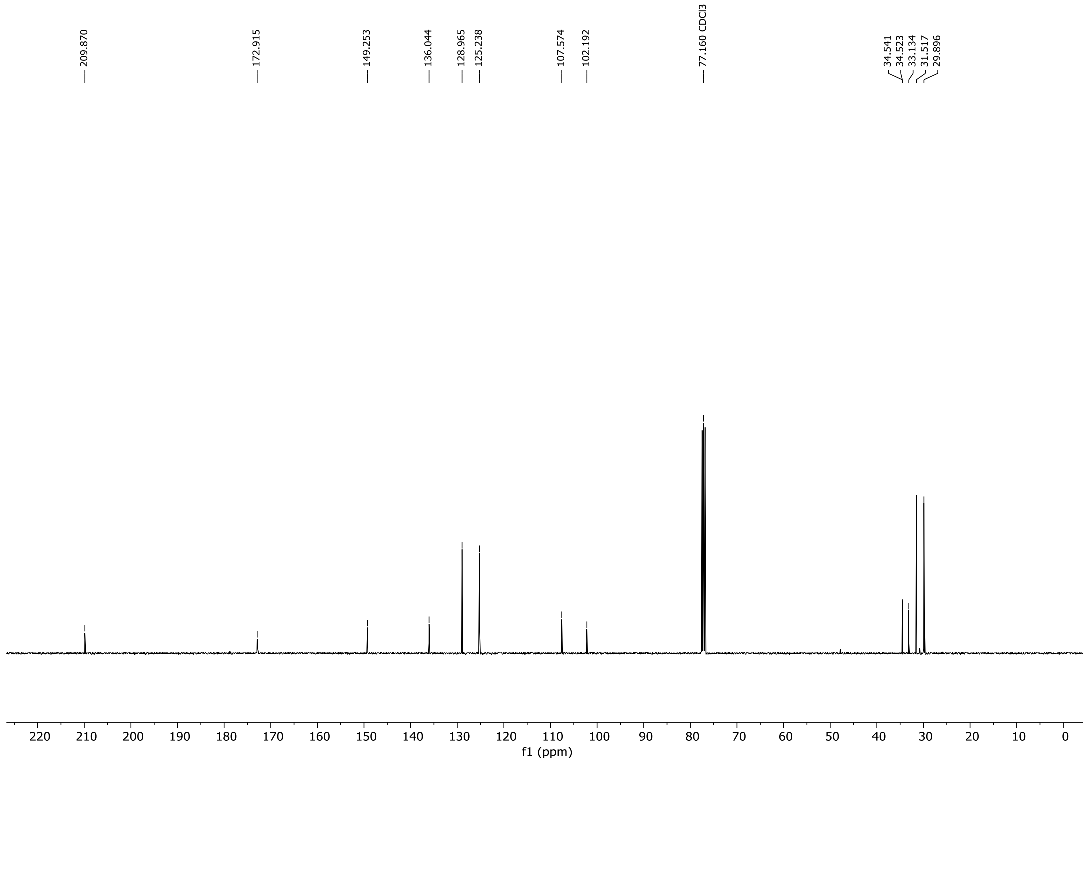

Methyl 2-(4-(tert-butyl)benzyl)-5,5-dimethylhexa-2,3-dienoate (*rac*-2g)

^{1}H NMR (500 MHz, $CDCl_3$, 300 K):

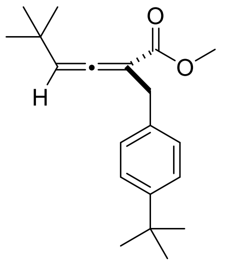

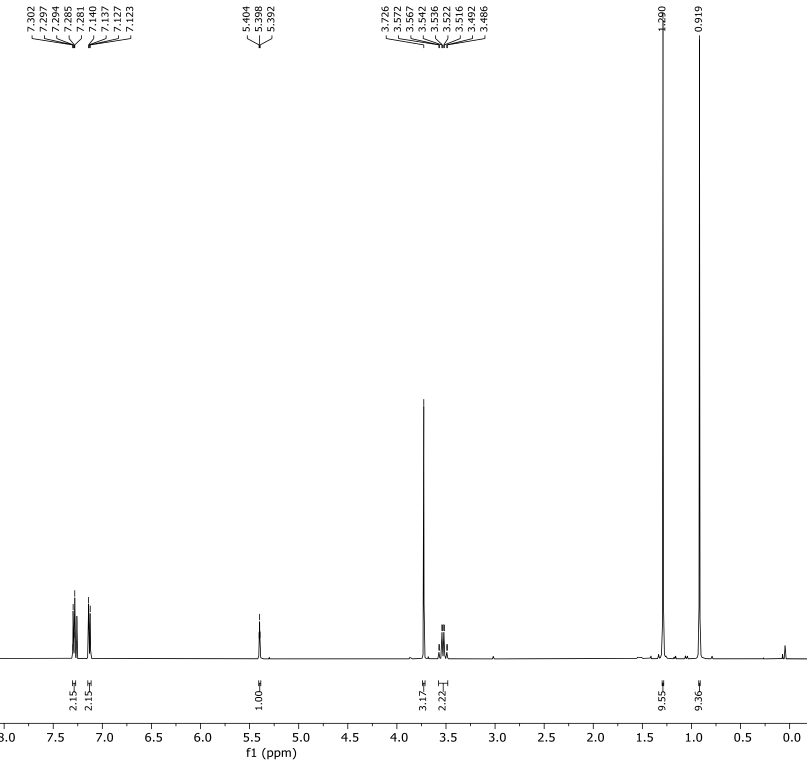

^{13}C NMR (101 MHz, $CDCl_3$, 300 K):

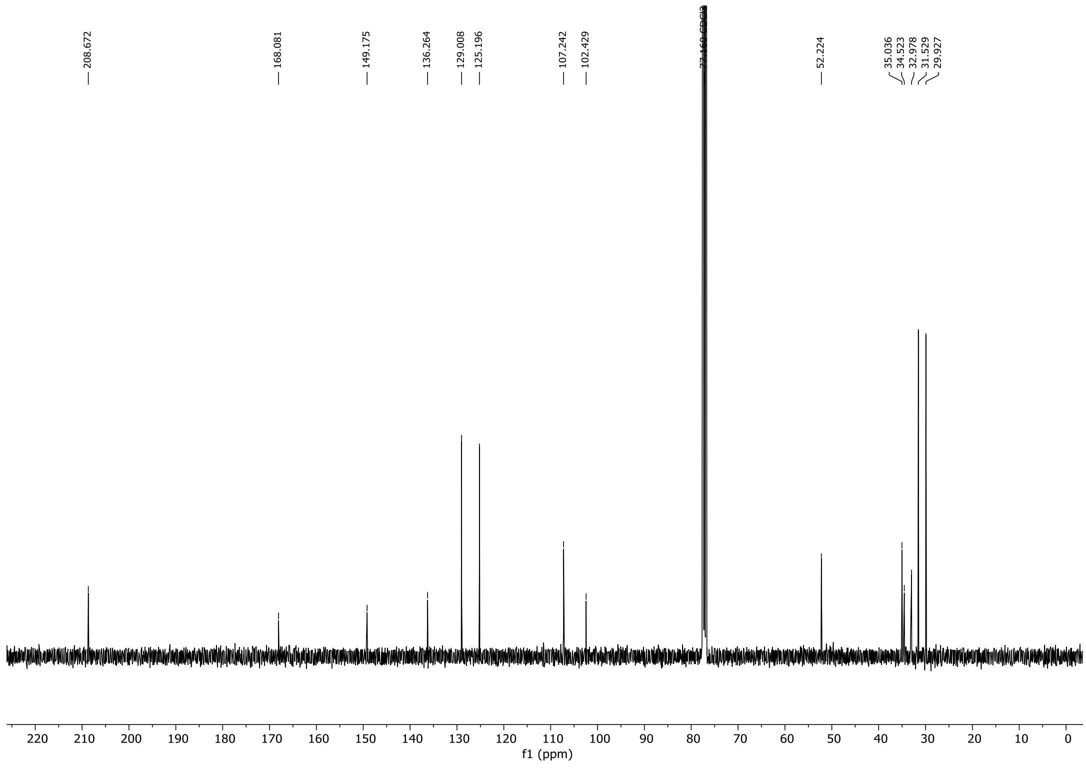

5,5-Dimethyl-2-(4-(methylthio)benzyl)hexa-2,3-dienoic acid (*rac*-1h)

^{1}H NMR (400 MHz, $CDCl_3$, 300 K):

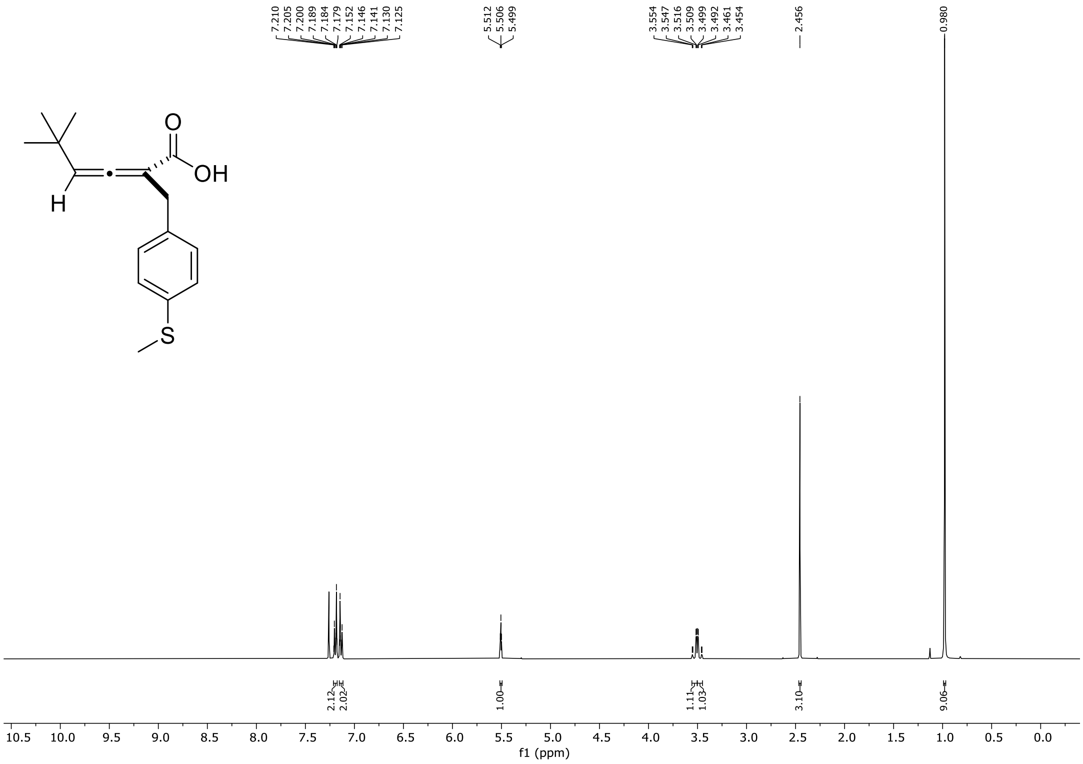

^{13}C NMR (101 MHz, $CDCl_3$, 300 K):

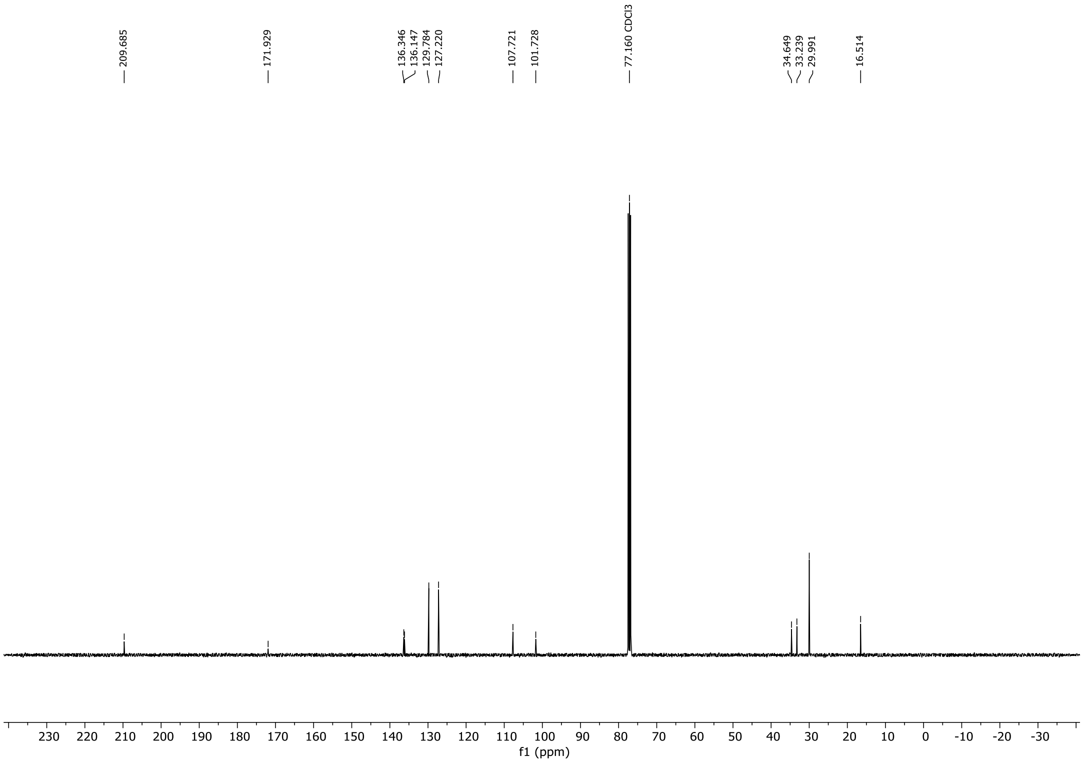

2-(3,5-Dimethylbenzyl)-5,5-dimethylhexa-2,3-dienoic acid (*rac*-1i)

^{1}H NMR (400 MHz, $CDCl_3$, 300 K):

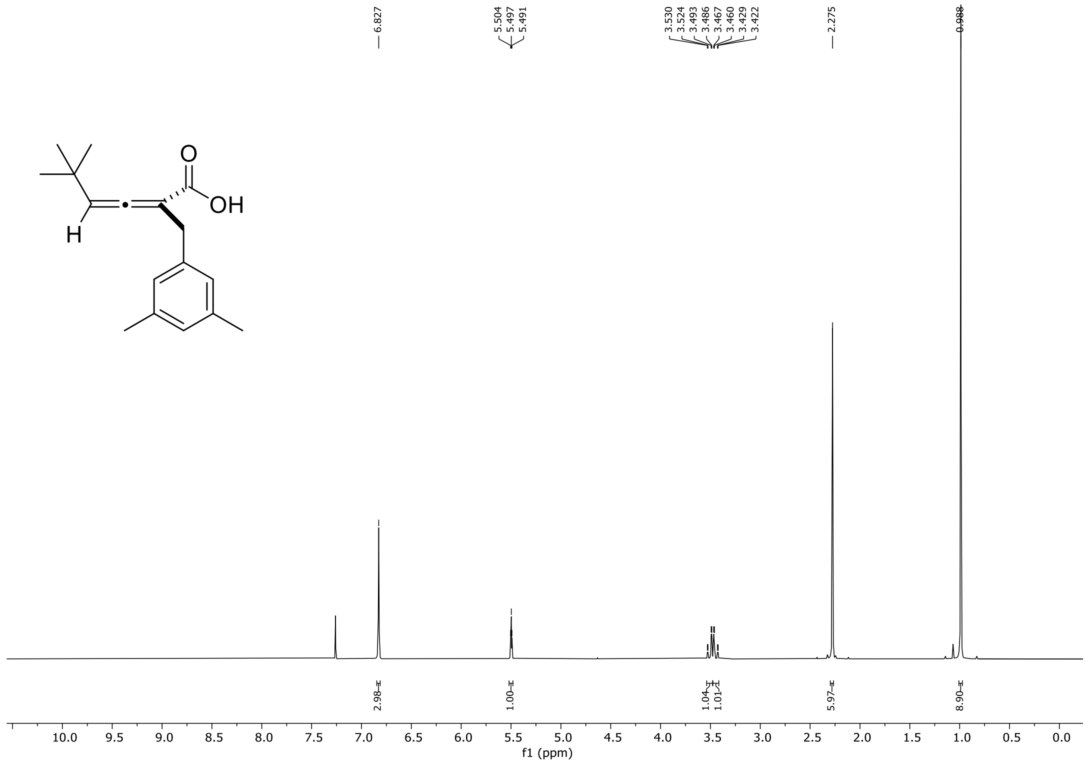

^{13}C NMR (101 MHz, $CDCl_3$, 300 K):

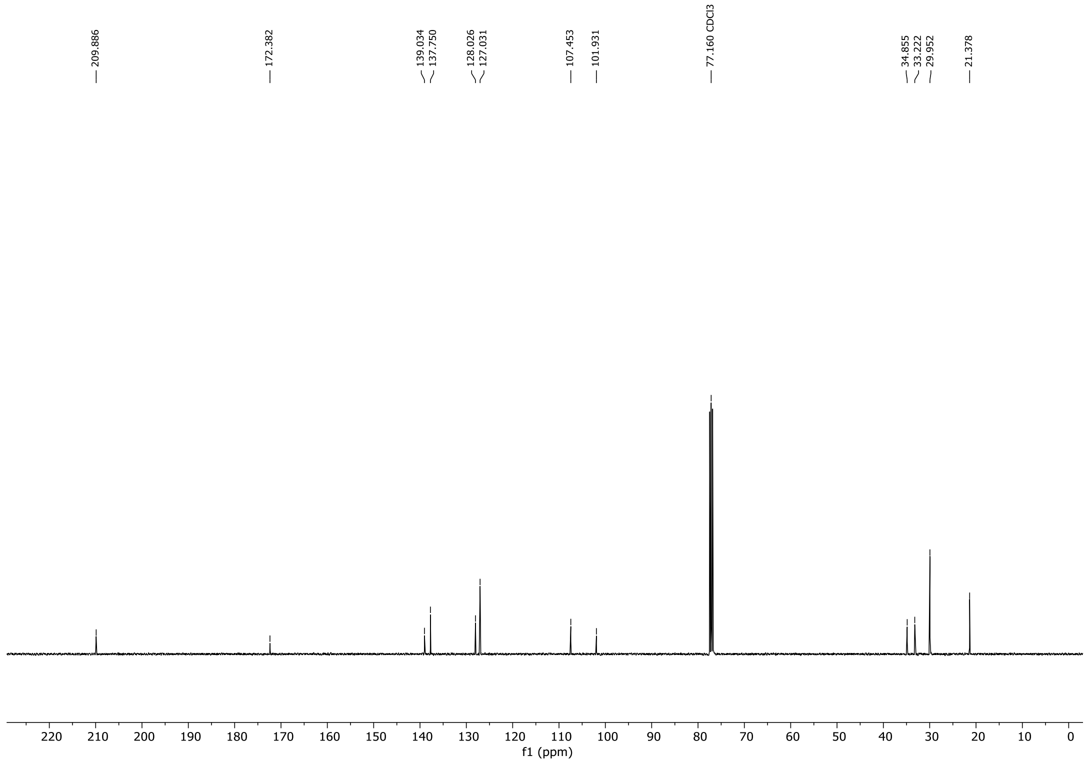

Methyl 2-(3,5-dimethylbenzyl)-5,5-dimethylhexa-2,3-dienoate (*rac*-2i)

^{1}H NMR (400 MHz, $CDCl_3$, 300 K):

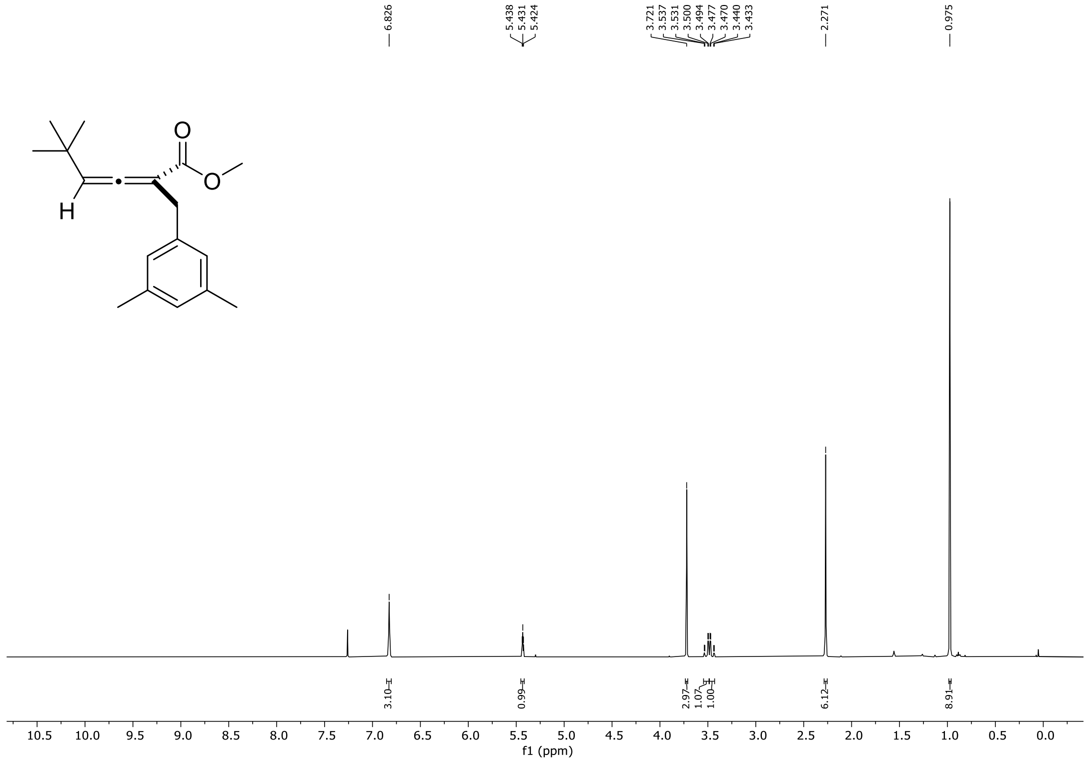

^{13}C NMR (101 MHz, $CDCl_3$, 300 K):

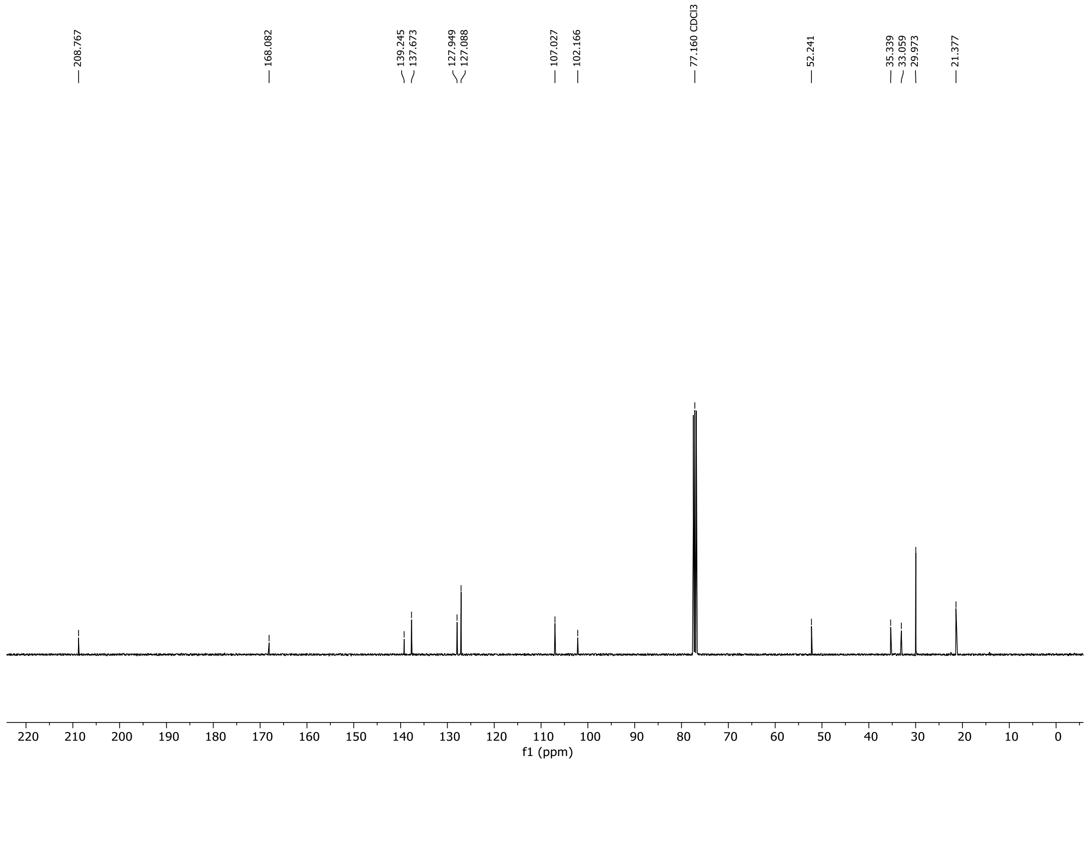

2-(3,5-Di-*tert*-butylbenzyl)-5,5-dimethylhexa-2,3-dienoic acid (*rac*-1j)

^{1}**H NMR** (400 MHz, $CDCl_3$, 300 K):

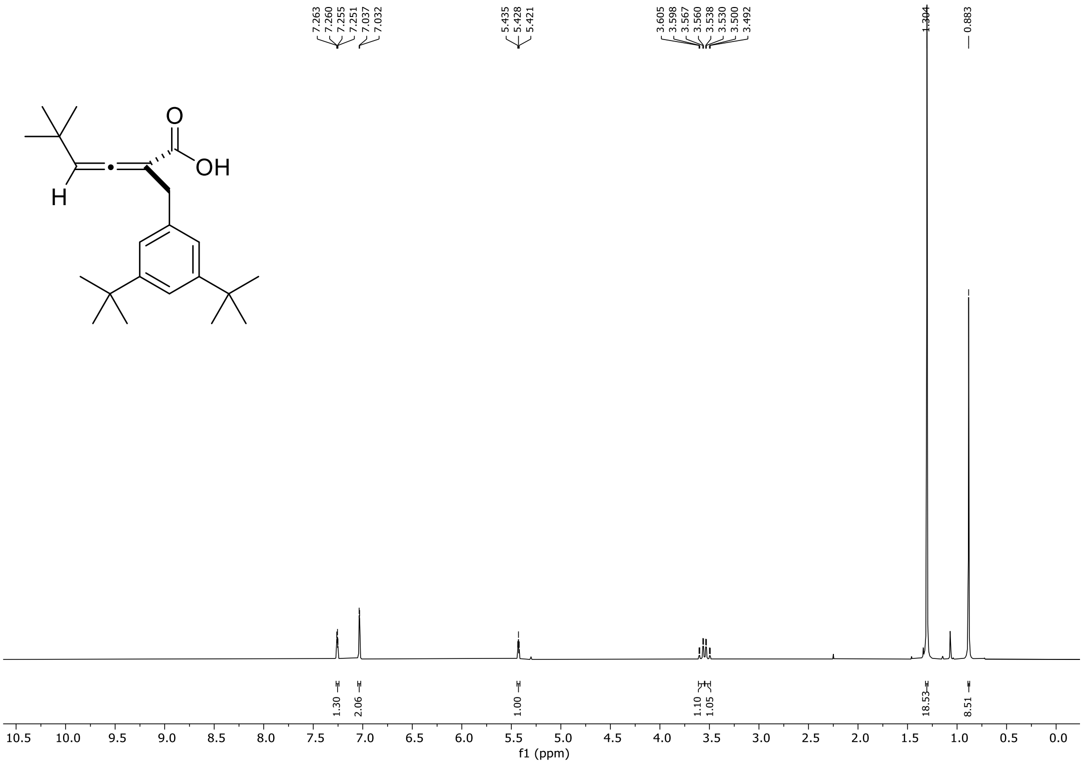

^{13}**C NMR** (101 MHz, $CDCl_3$, 300 K):

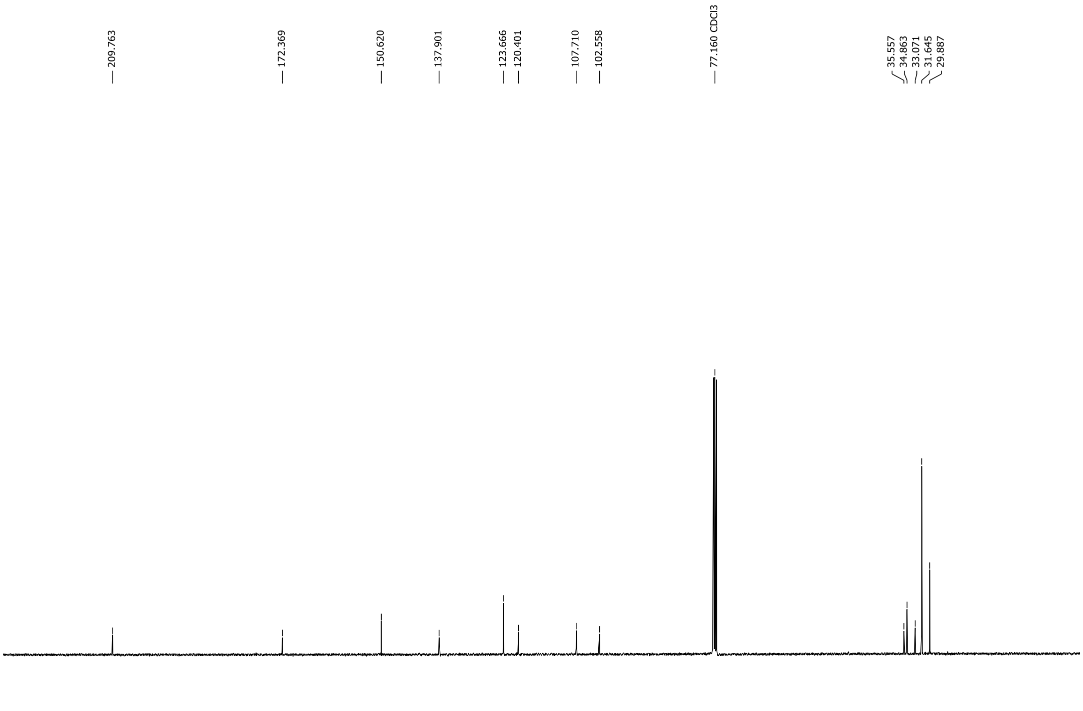

Methyl 2-(3,5-di-tert-butylbenzyl)-5,5-dimethylhexa-2,3-dienoate (*rac*-2j)

^{1}H NMR (500 MHz, $CDCl_3$, 300 K):

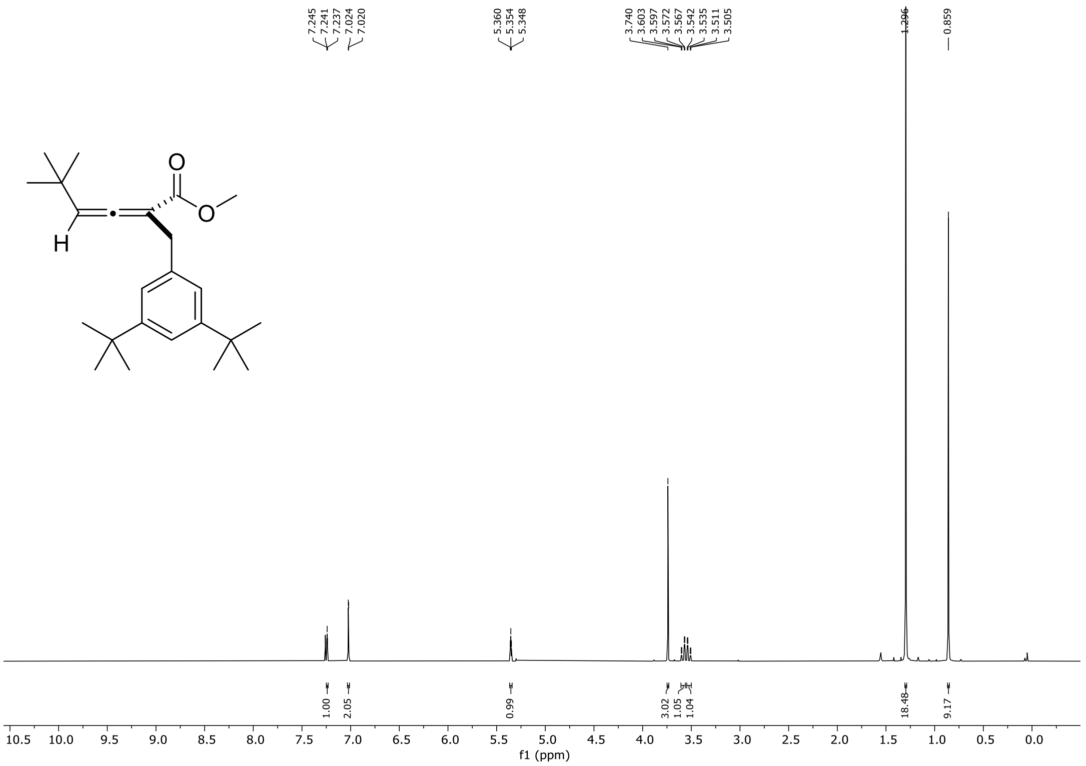

^{13}C NMR (126 MHz, $CDCl_3$, 300 K):

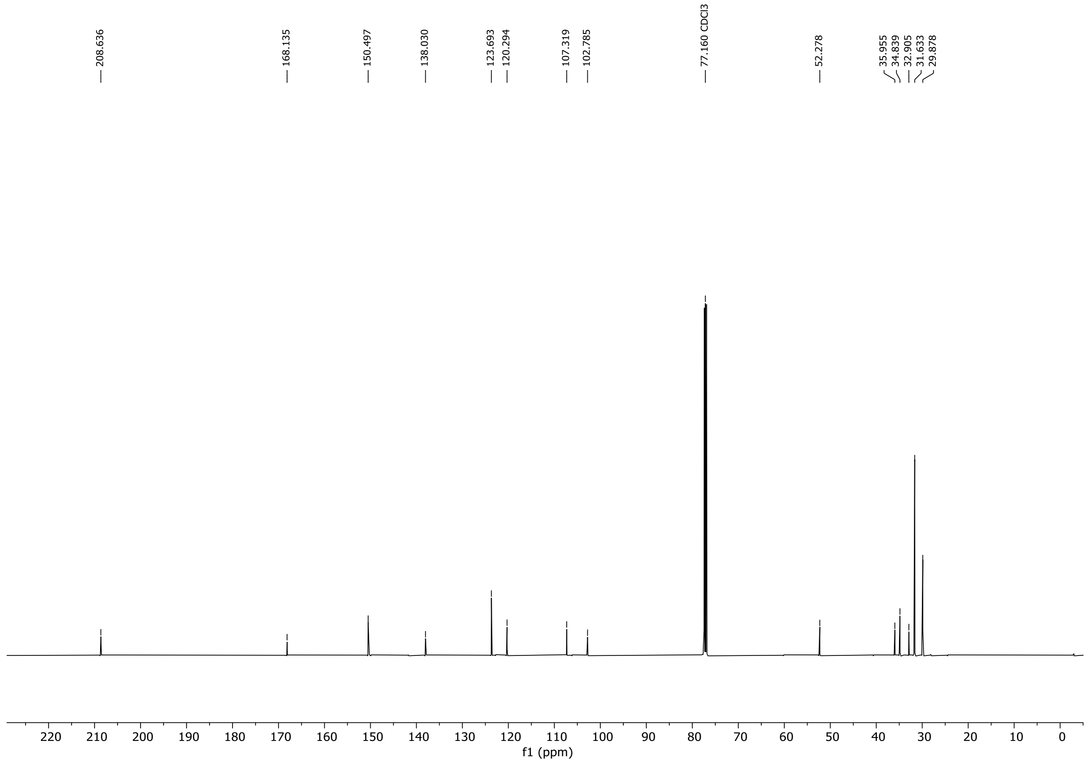

2-([1,1'-Biphenyl]-3-ylmethyl)-5,5-dimethylhexa-2,3-dienoic acid (*rac*-1k)

^{1}H NMR (400 MHz, $CDCl_3$, 300 K):

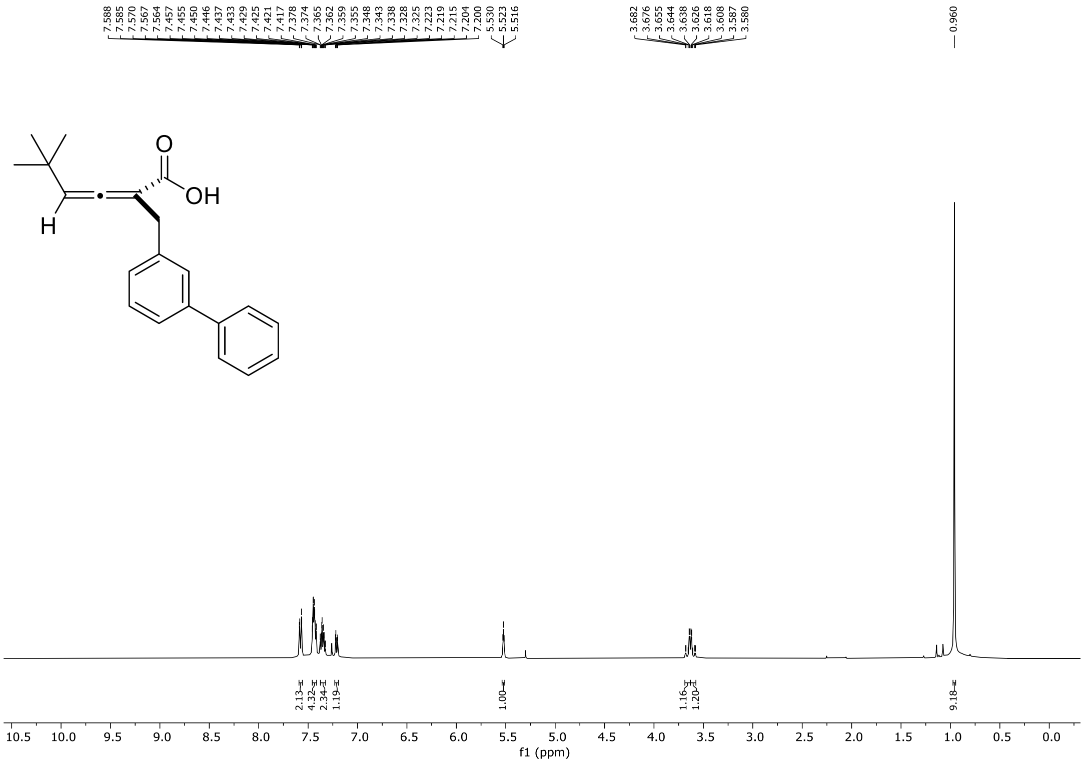

^{13}C NMR (101 MHz, $CDCl_3$, 300 K):

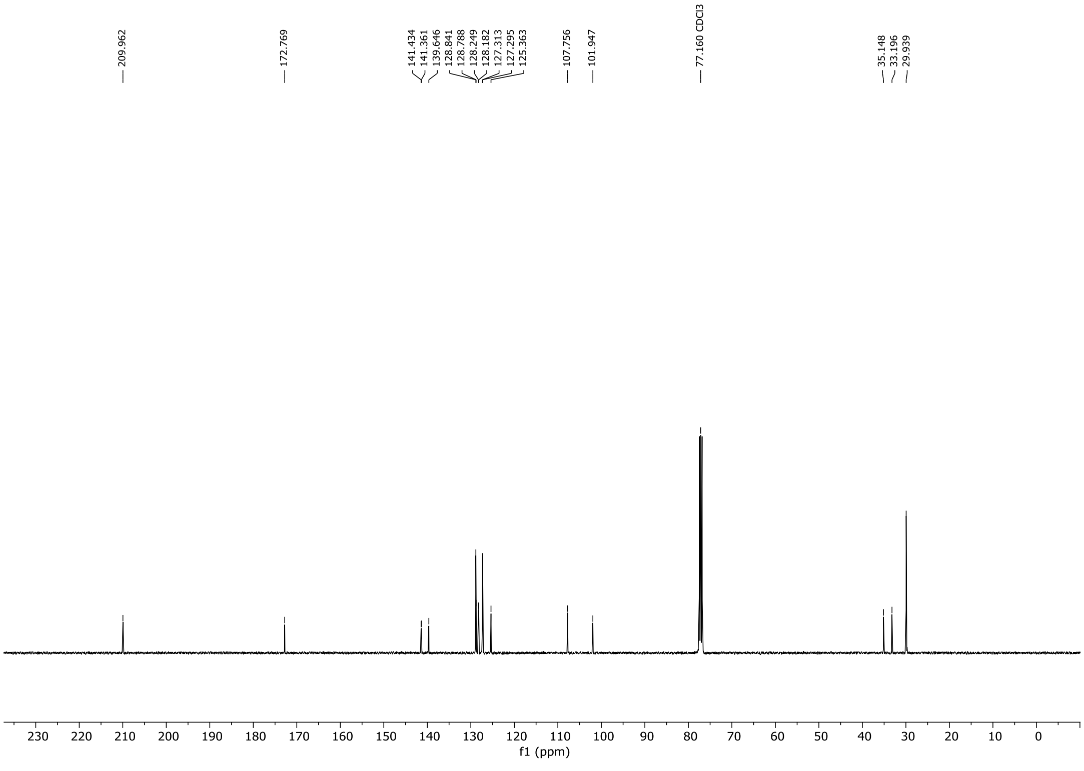

Methyl 2-([1,1'-biphenyl]-3-ylmethyl)-5,5-dimethylhexa-2,3-dienoate (*rac*-2k)

^{1}H NMR (400 MHz, $CDCl_3$, 300 K):

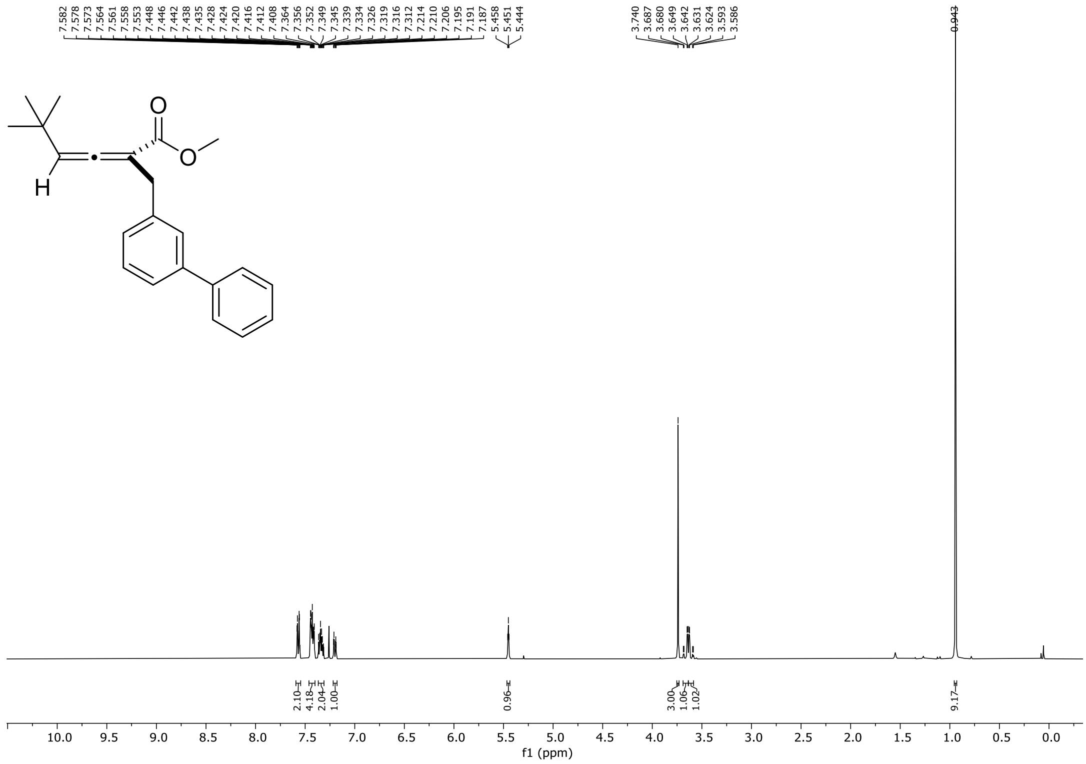

^{13}C NMR (101 MHz, $CDCl_3$, 300 K):

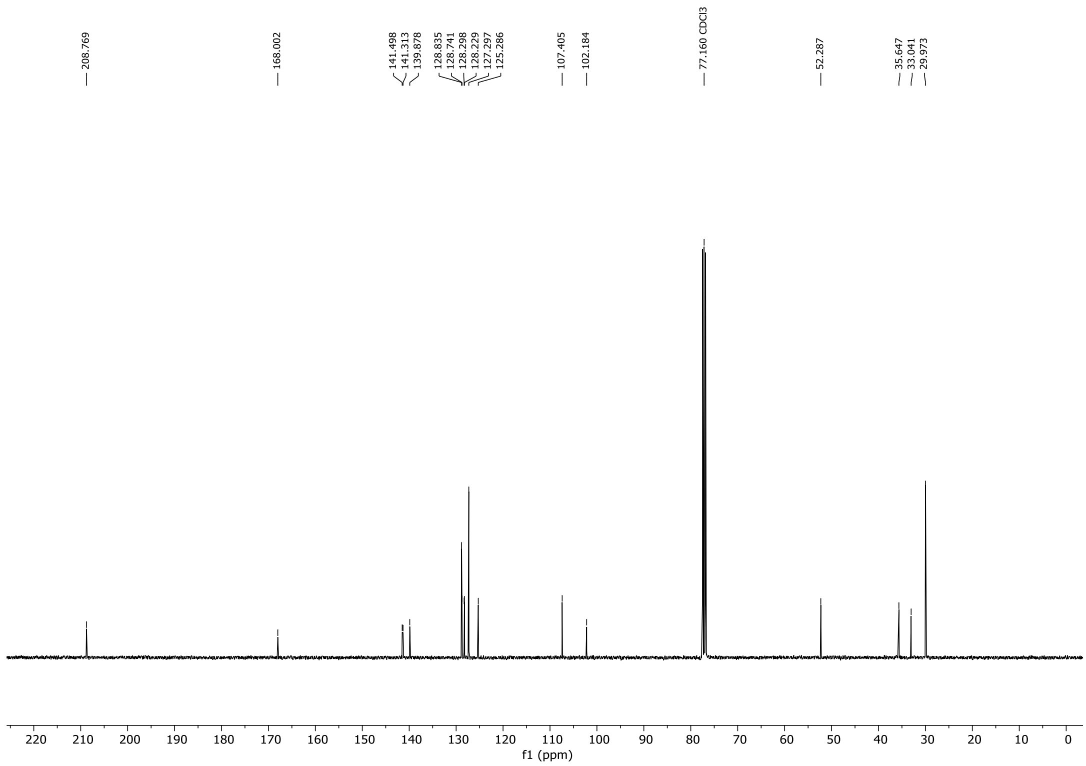

2-(3-Cyanobenzyl)-5,5-dimethylhexa-2,3-dienoic acid (*rac*-1l)

^{1}H NMR (400 MHz, $CDCl_3$, 300 K):

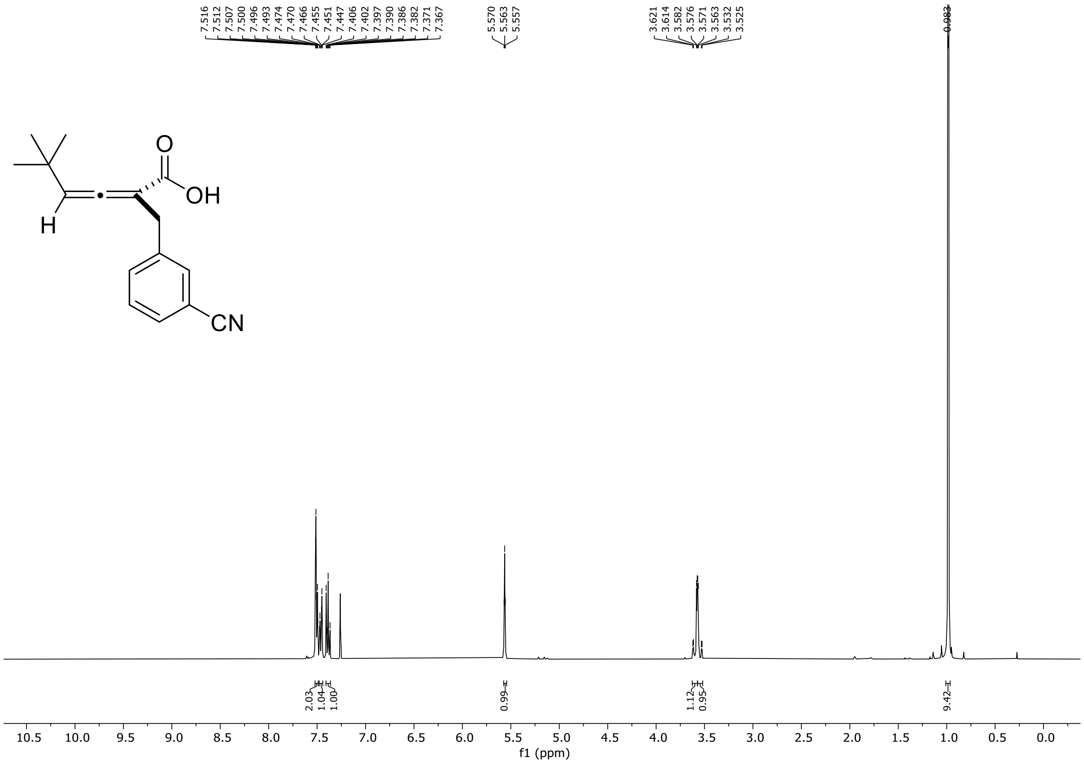

^{13}C NMR (101 MHz, $CDCl_3$, 300 K):

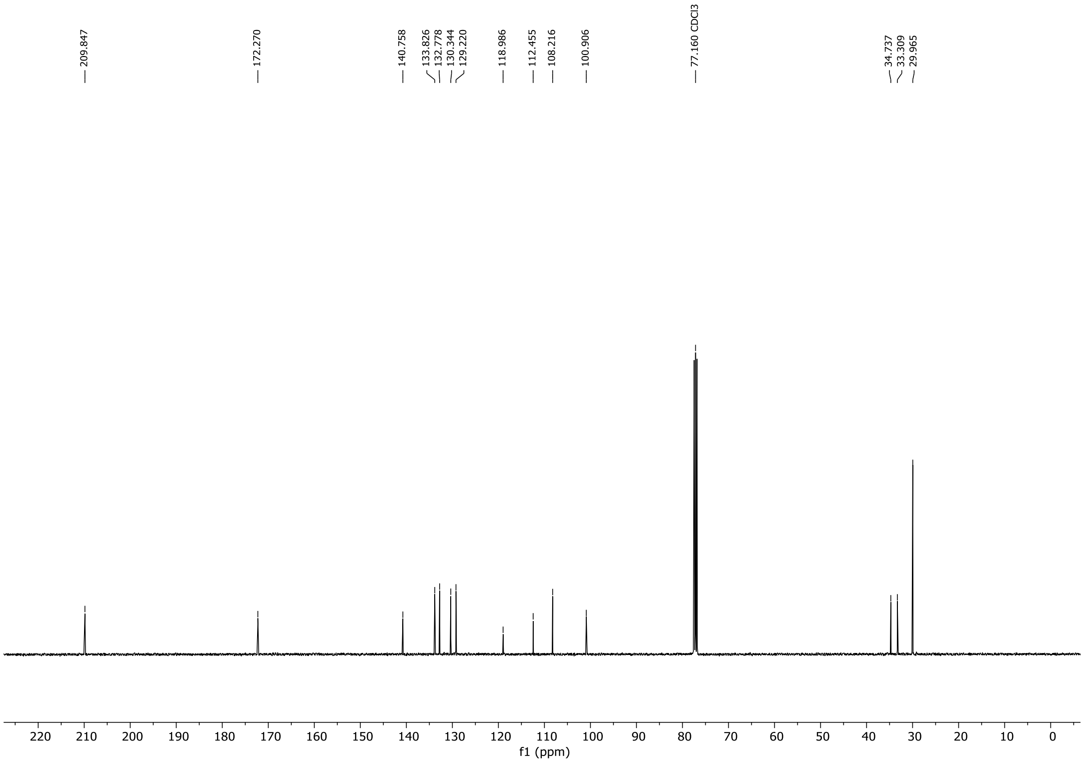

Methyl 2-(3-cyanobenzyl)-5,5-dimethylhexa-2,3-dienoate (*rac*-2l)

^{1}H NMR (400 MHz, $CDCl_3$, 300 K):

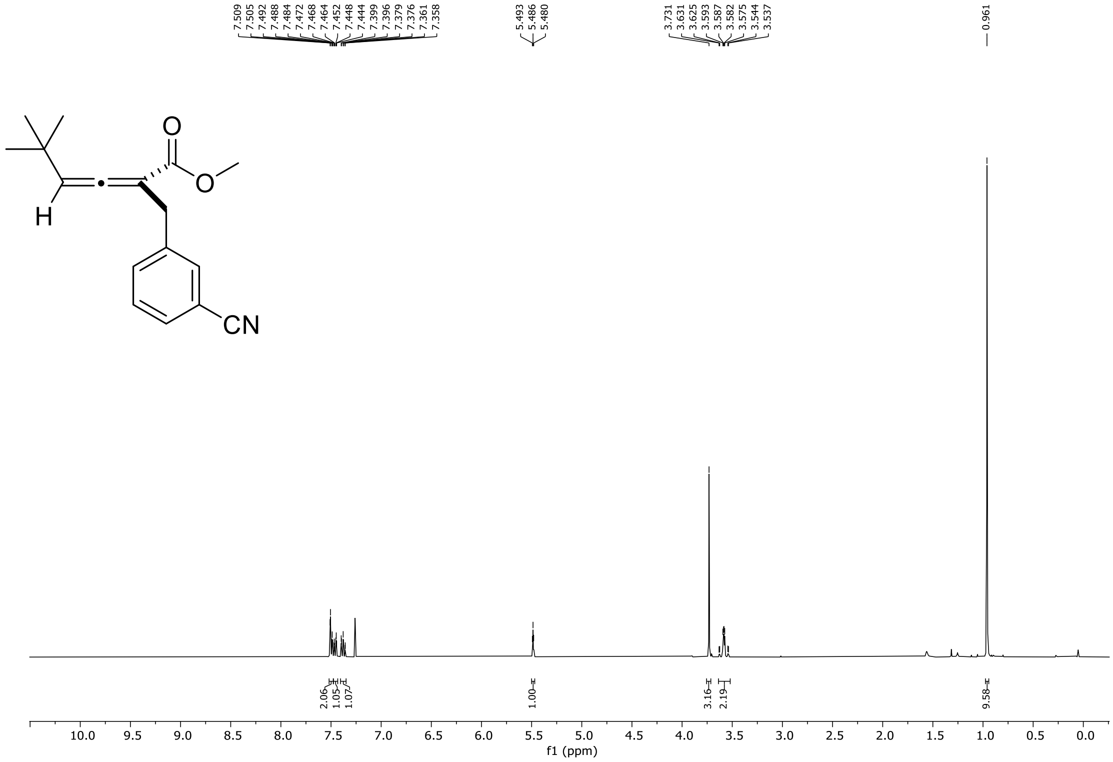

^{13}C NMR (101 MHz, $CDCl_3$, 300 K):

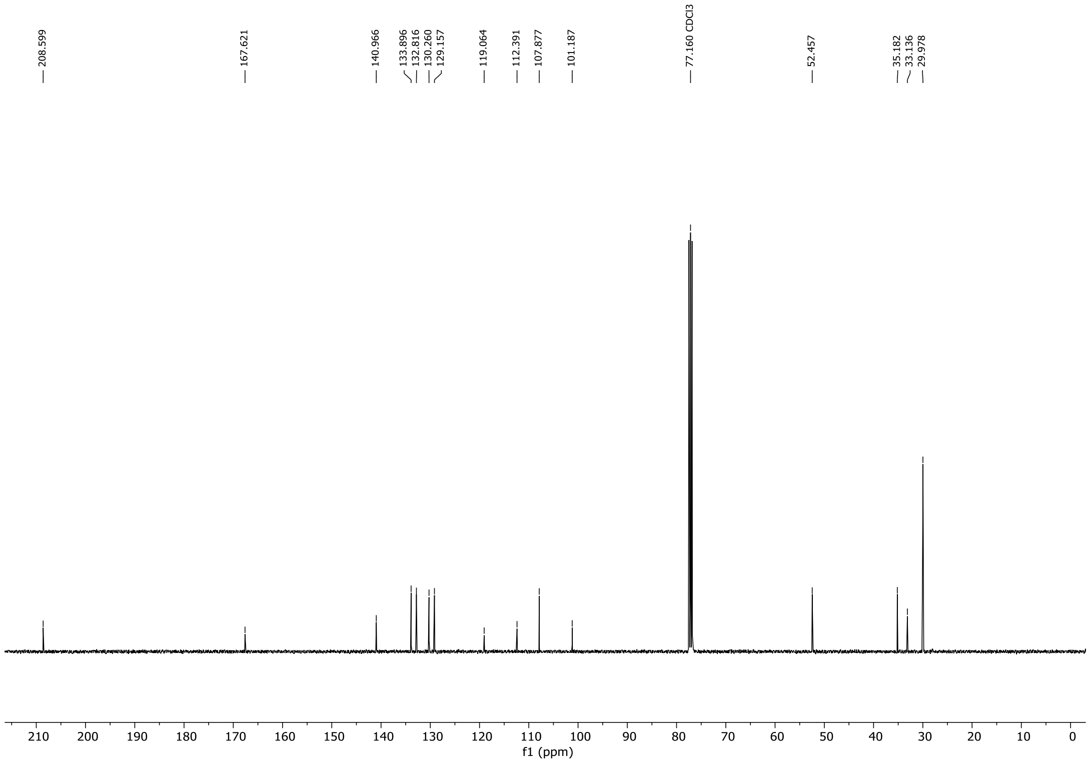

2-(3-Methoxybenzyl)-5,5-dimethylhexa-2,3-dienoic acid (*rac*-1m)

^{1}H NMR (400 MHz, $CDCl_3$, 300 K):

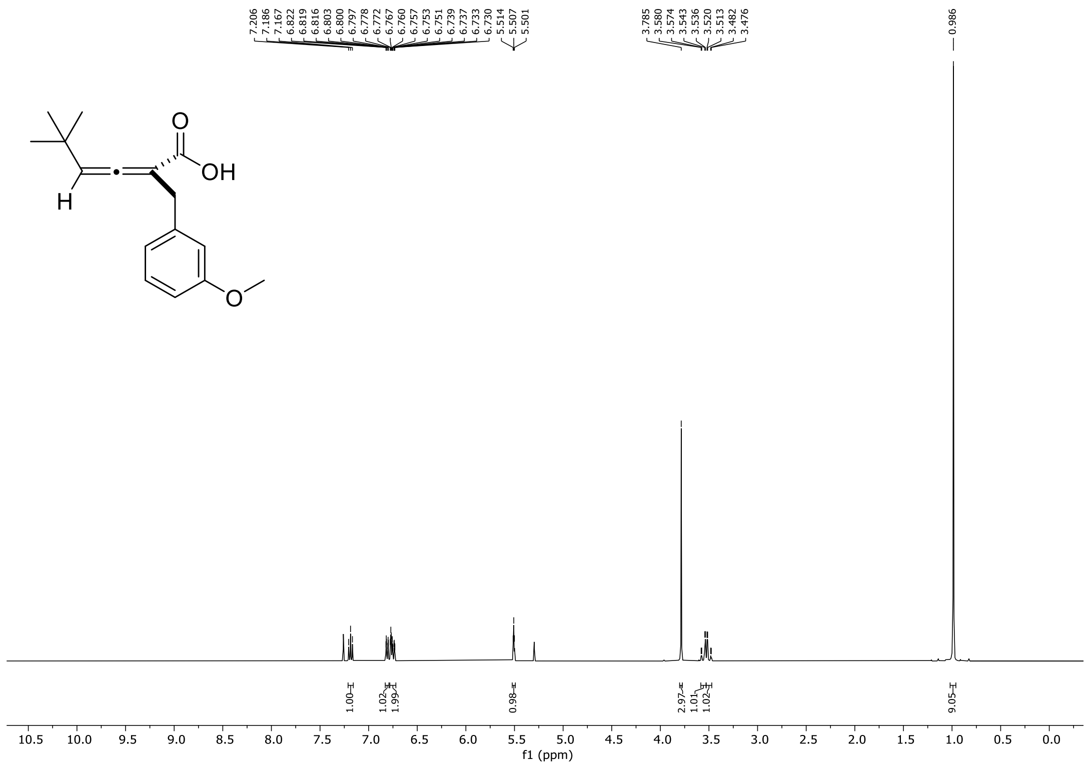

^{13}C NMR (101 MHz, $CDCl_3$, 300 K):

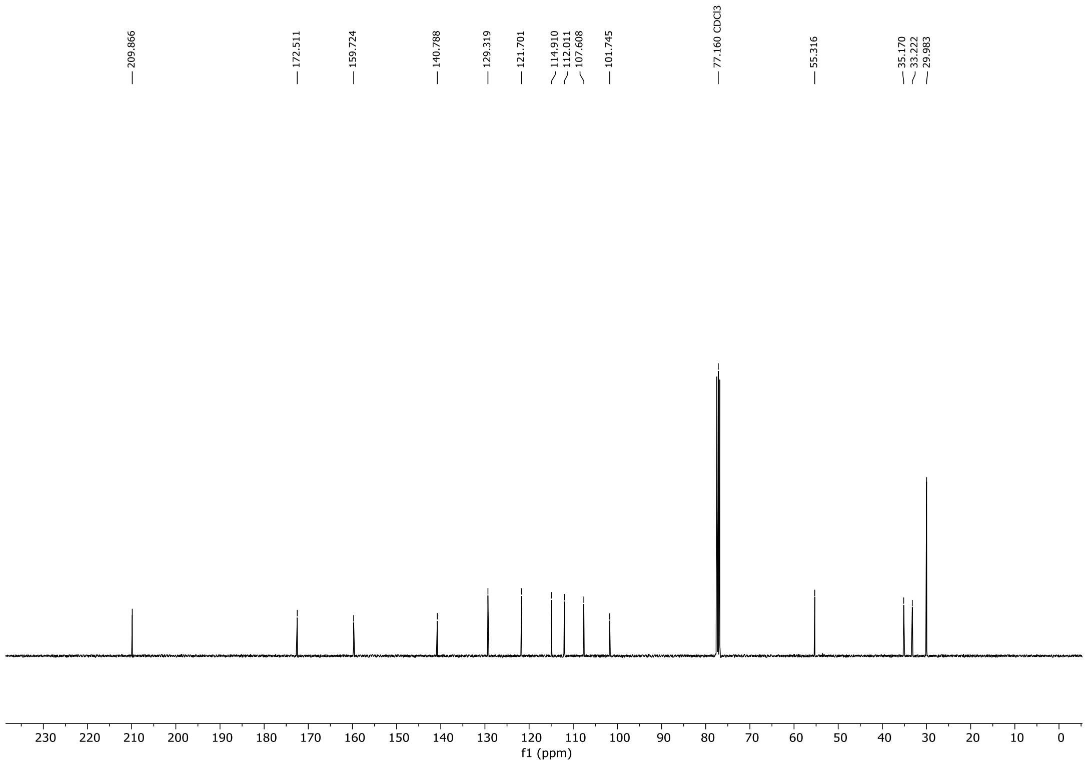

2-(Benzo[*d*][1,3]dioxol-5-ylmethyl)-5,5-dimethylhexa-2,3-dienoic acid (*rac*-1n)

^{1}H NMR (400 MHz, $CDCl_3$, 300 K):

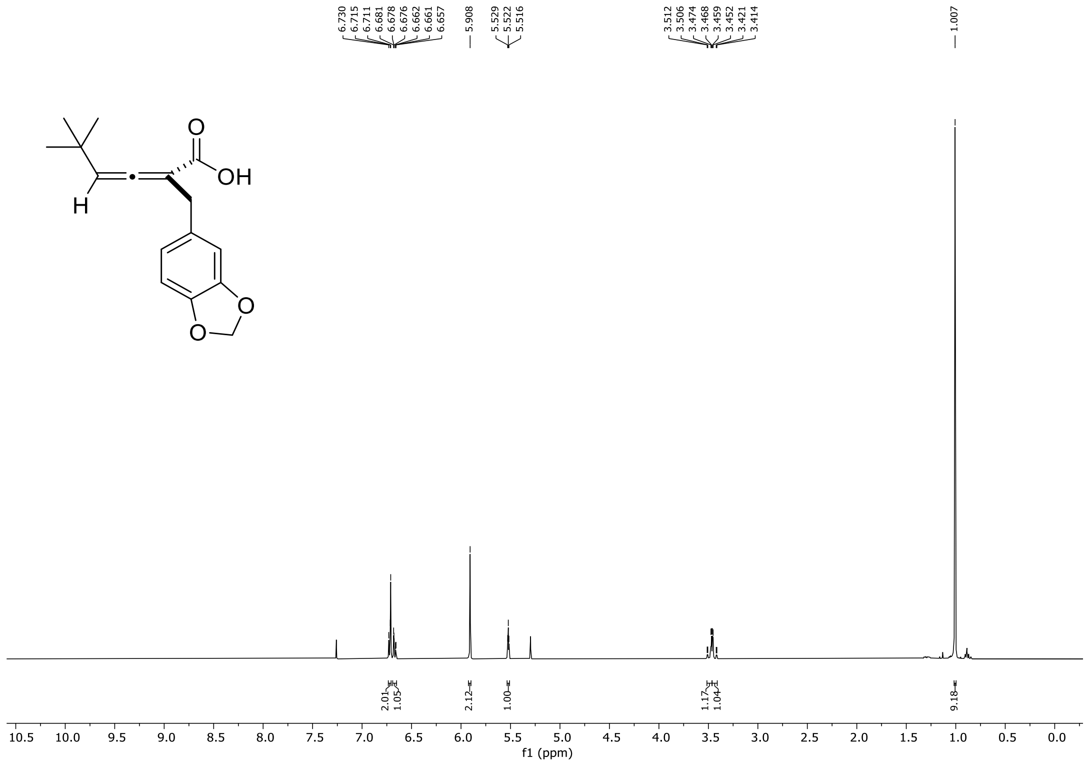

^{13}C NMR (101 MHz, $CDCl_3$, 300 K):

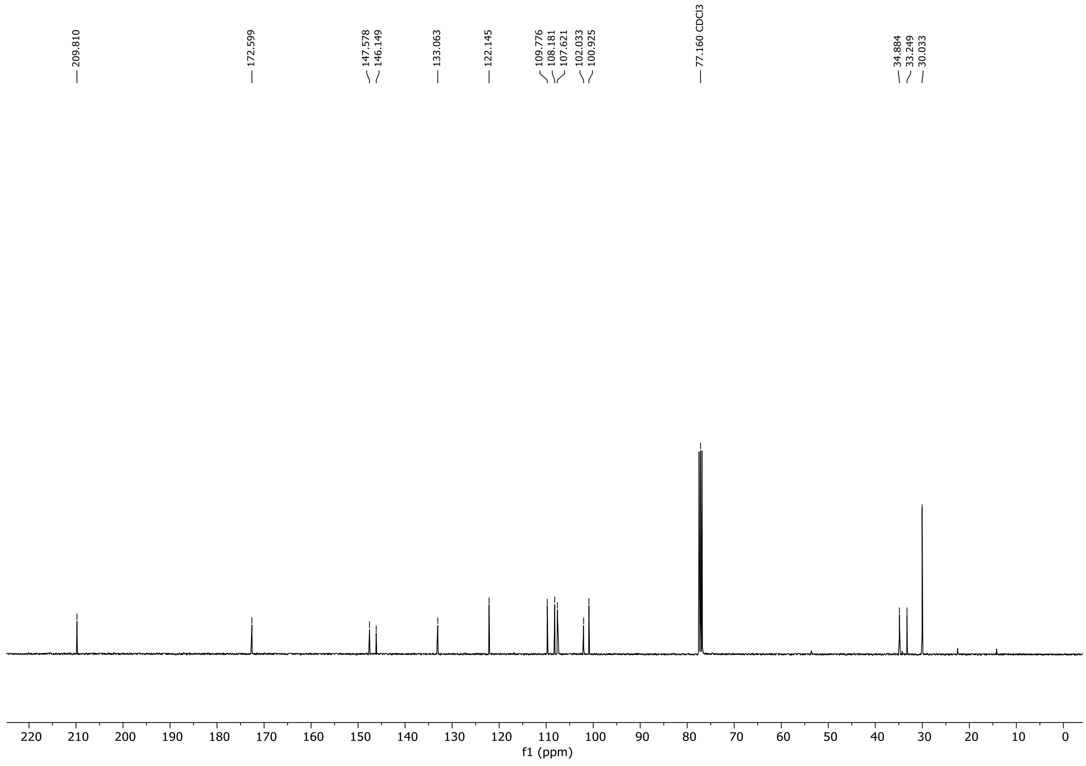

2-(2,6-Dichlorobenzyl)-5,5-dimethylhexa-2,3-dienoic acid (*rac*-1o)

^{1}H NMR (400 MHz, $CDCl_3$, 300 K):

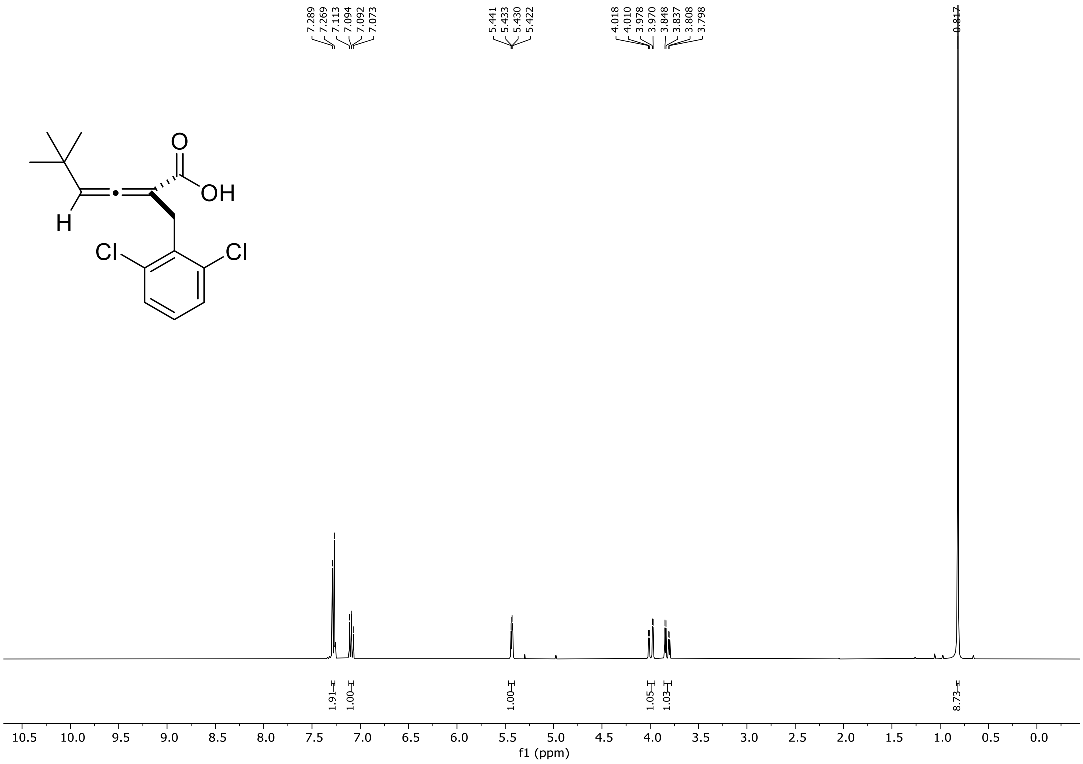

^{13}C NMR (101 MHz, $CDCl_3$, 300 K):

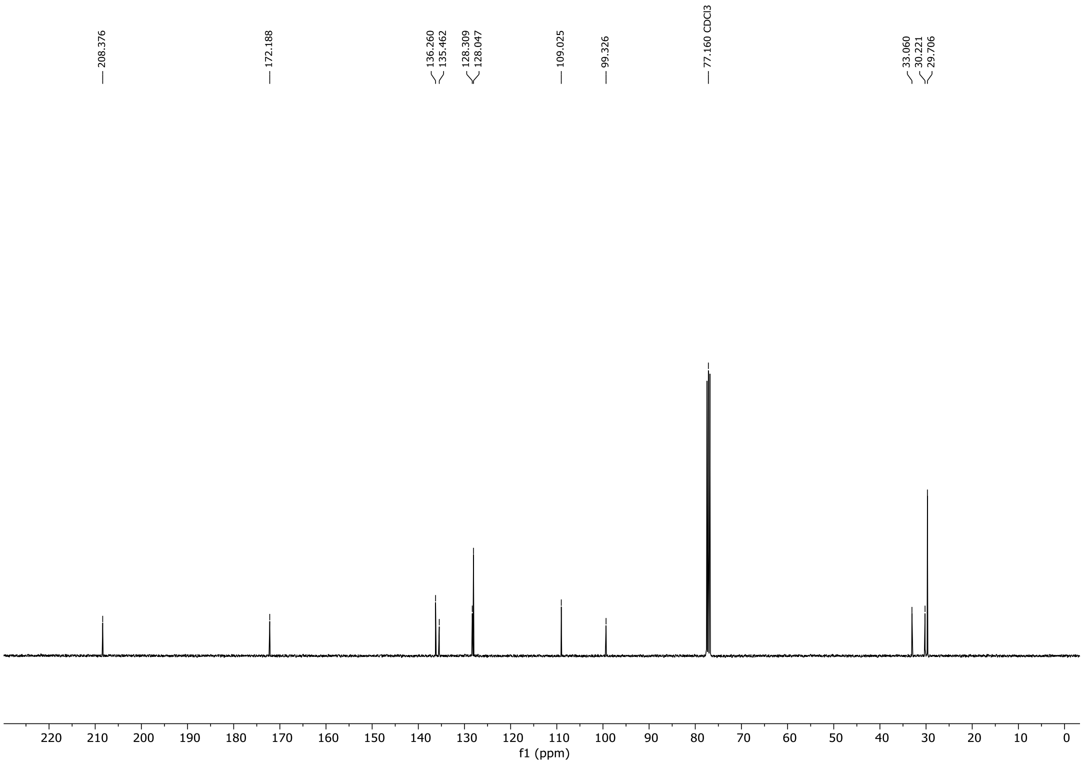

5,5-Dimethyl-2-((perfluorophenyl)methyl)hexa-2,3-dienoic acid (*rac*-1p)

^{1}H NMR (500 MHz, $CDCl_3$, 300 K):

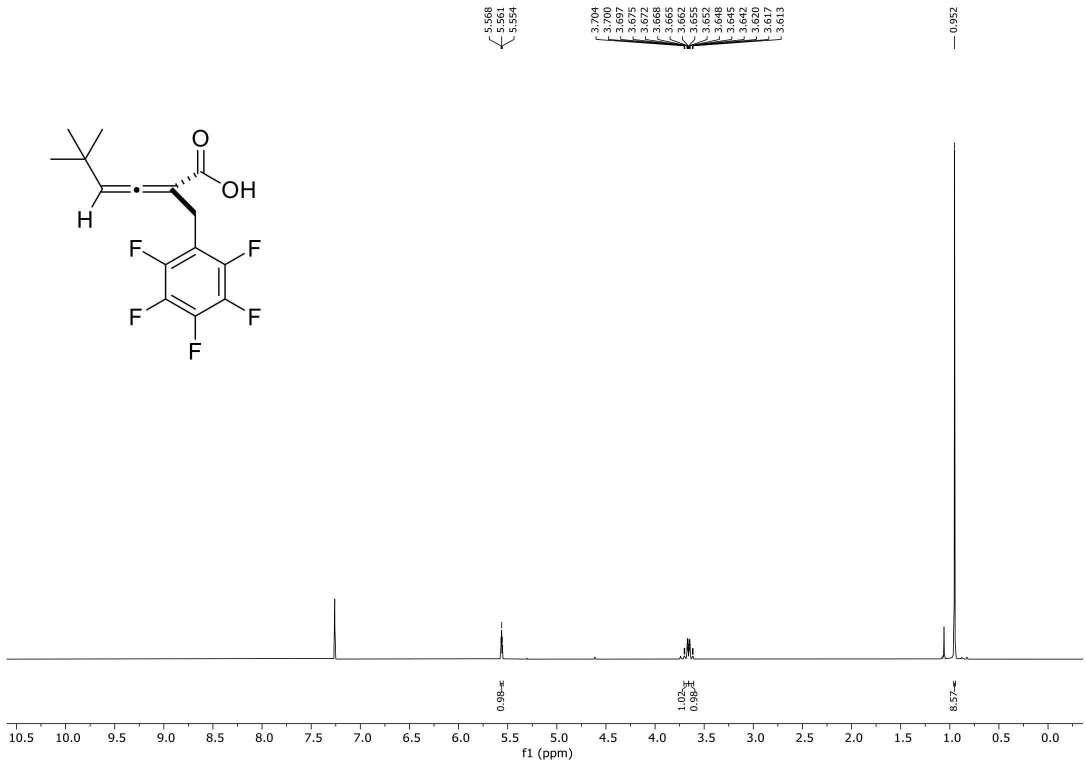

^{13}C NMR (126 MHz, $CDCl_3$, 300 K):

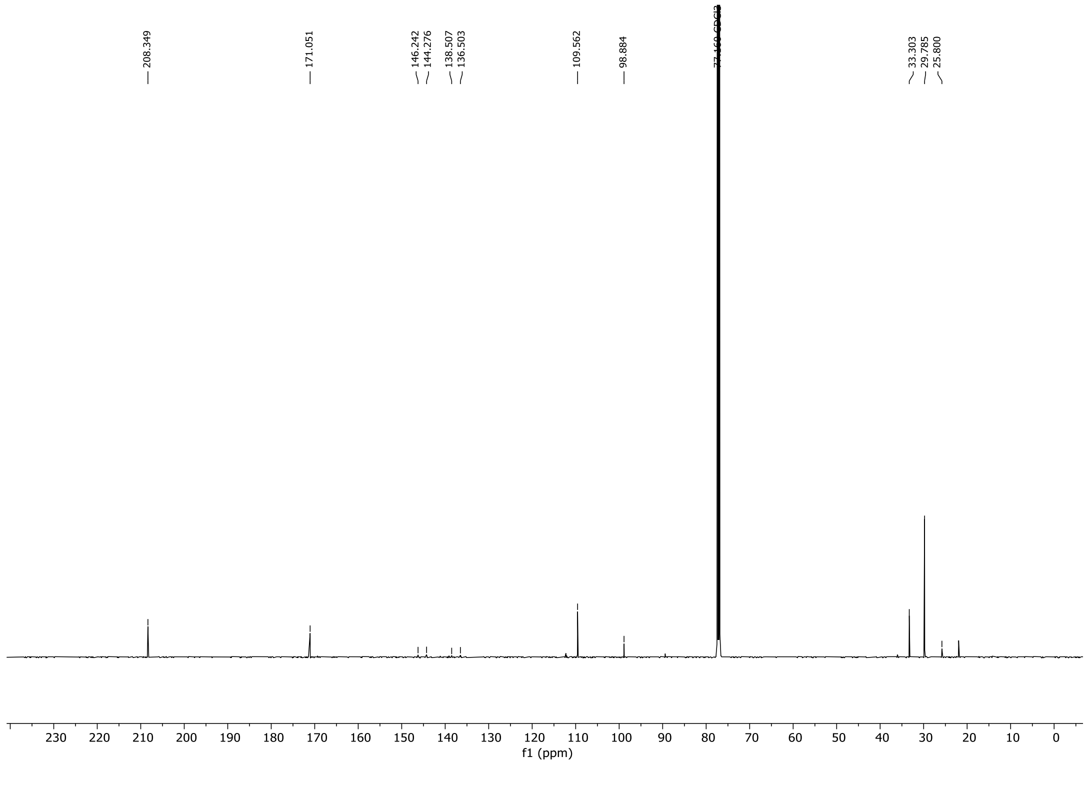

^{19}F NMR (471 MHz, $CDCl_3$, 300 K):

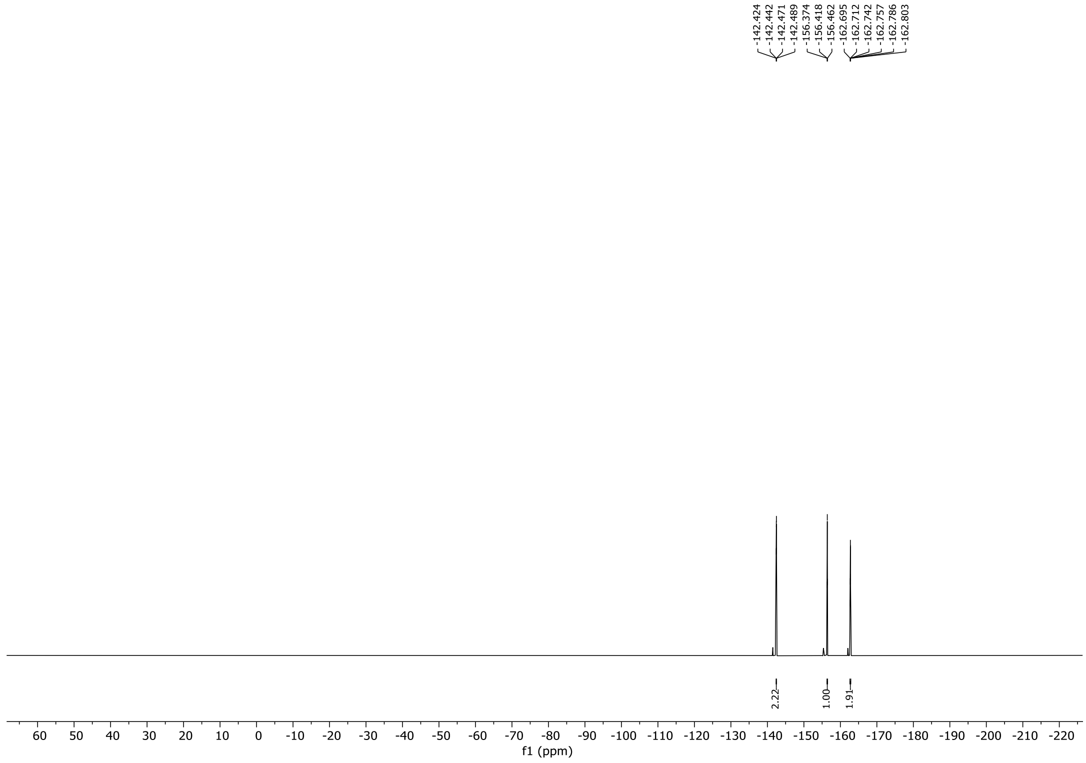

5,5-Dimethyl-2-(naphthalen-1-ylmethyl)hexa-2,3-dienoic acid (*rac*-1q)

^{1}H NMR (400 MHz, $CDCl_3$, 300 K):

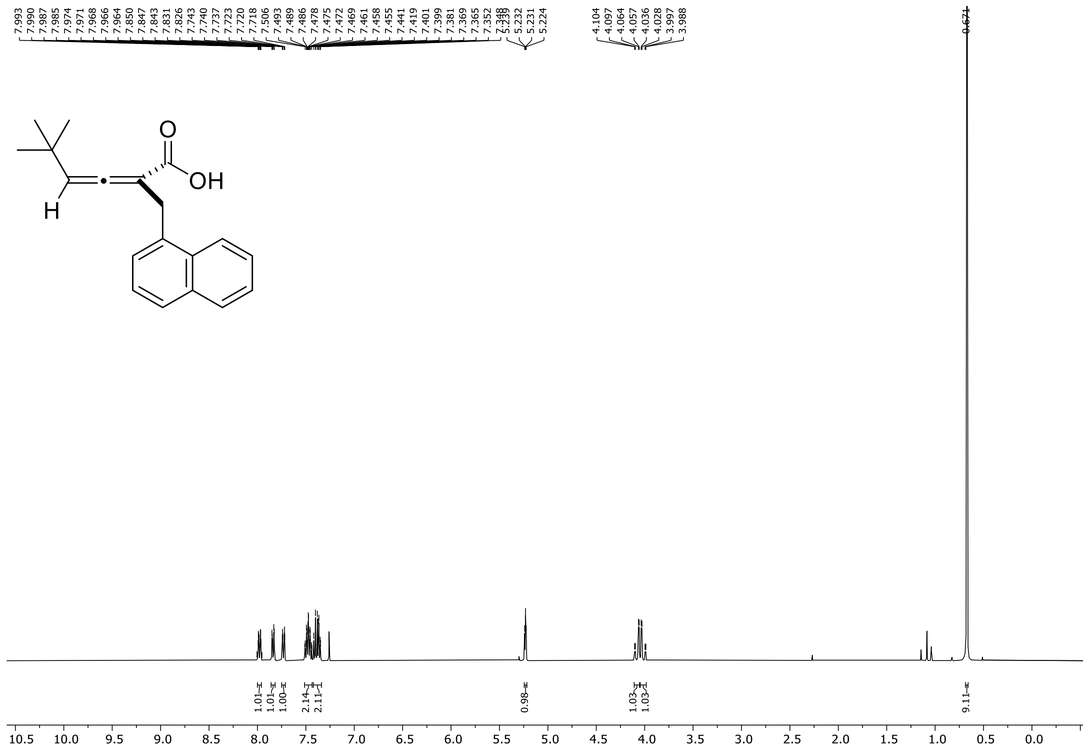

^{13}C NMR (101 MHz, $CDCl_3$, 300 K):

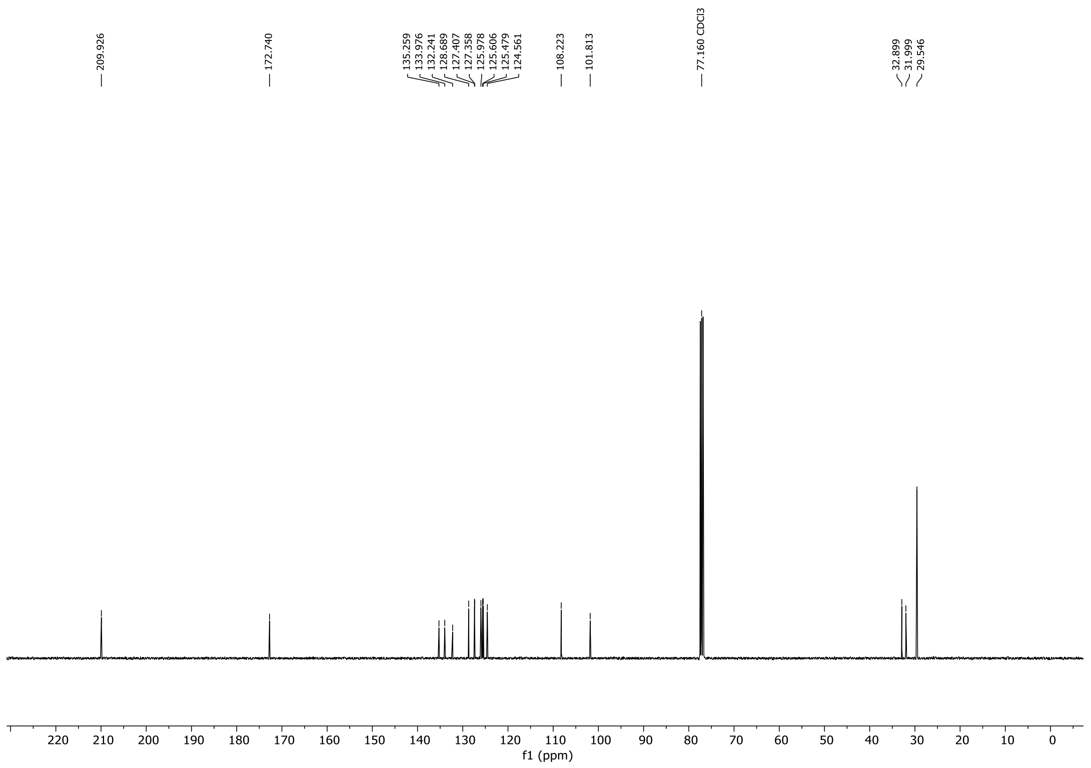

2-Benzylpenta-2,3-dienoic acid (*rac*-1r)

^{1}H NMR (400 MHz, $CDCl_3$, 300 K):

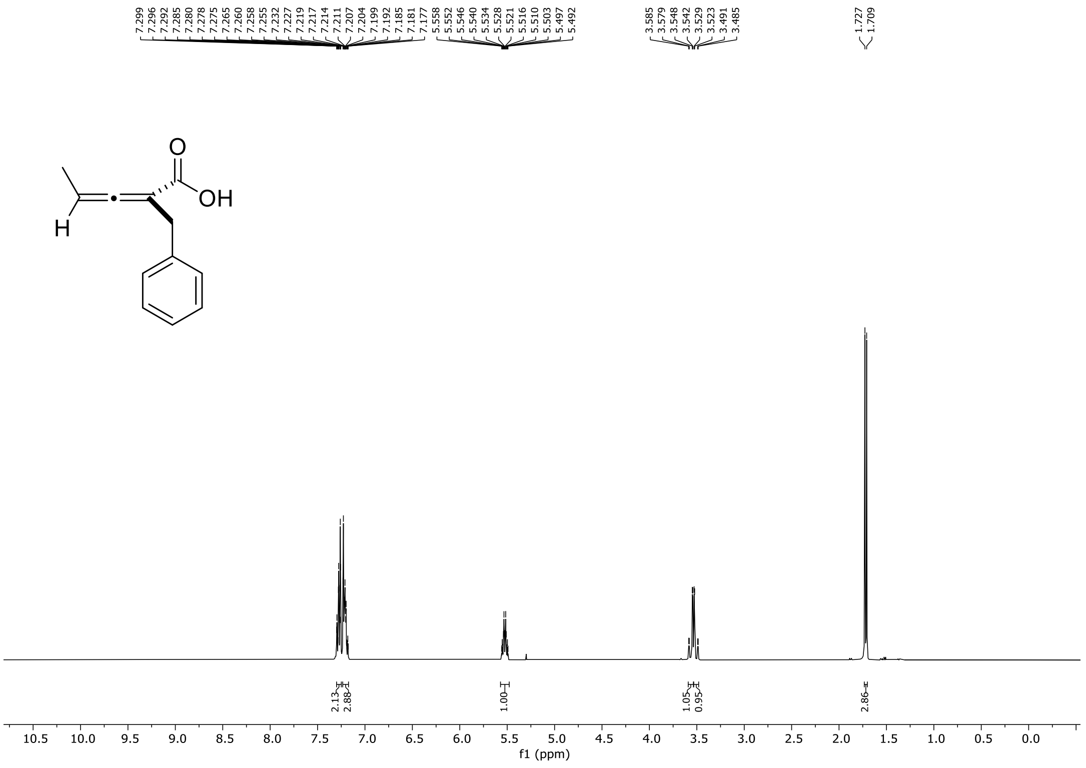

^{13}C NMR (101 MHz, $CDCl_3$, 300 K):

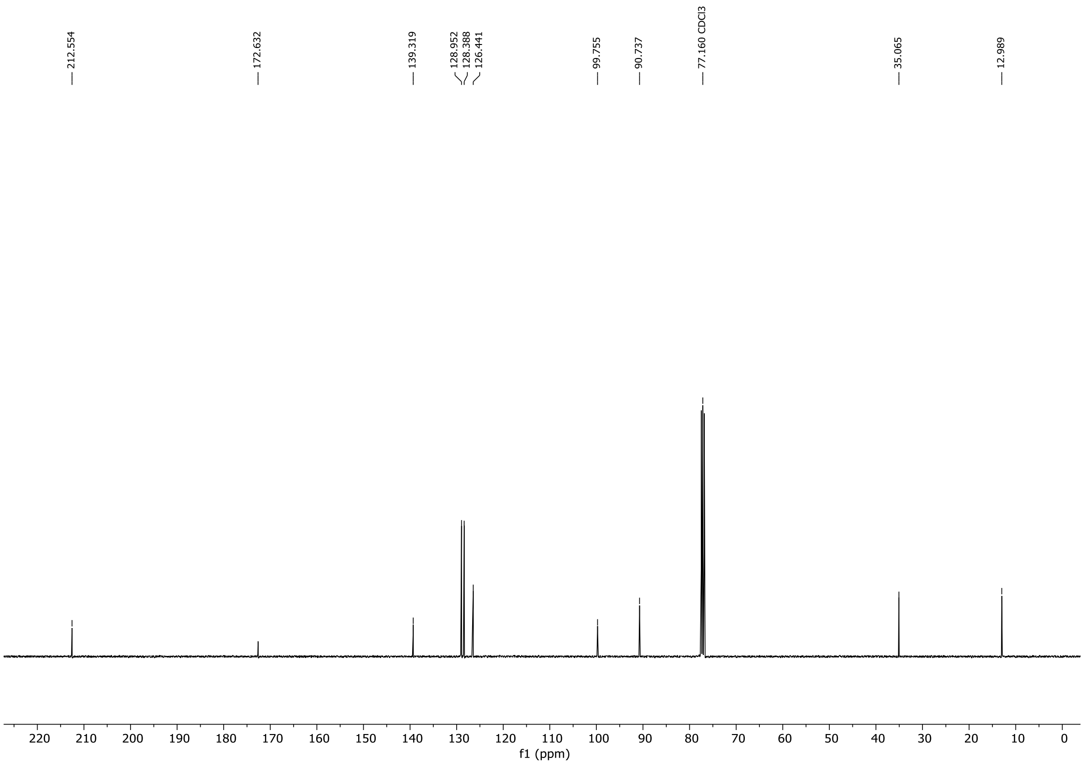

Methyl 2-benzylpenta-2,3-dienoate (*rac*-2r)

^{1}H NMR (400 MHz, $CDCl_3$, 300 K):

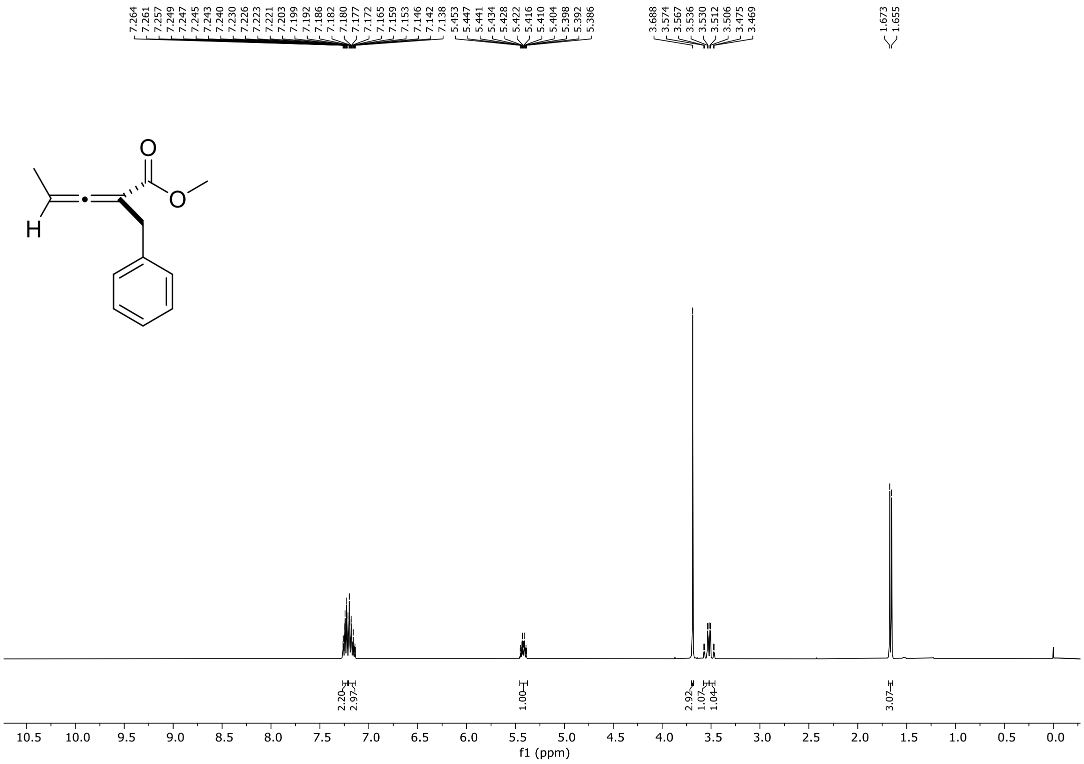

^{13}C NMR (101 MHz, $CDCl_3$, 300 K):

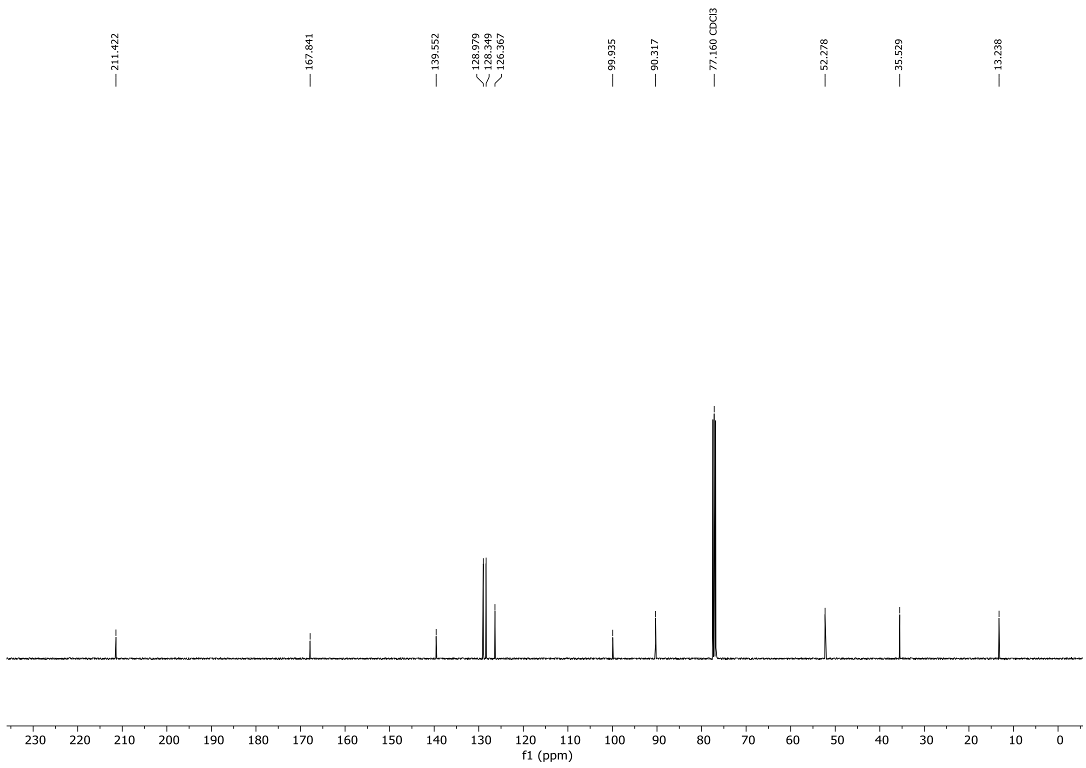

2-Benzyl-4-cyclopentylbuta-2,3-dienoic acid (*rac*-1s)

^{1}H NMR (400 MHz, $CDCl_3$, 300 K):

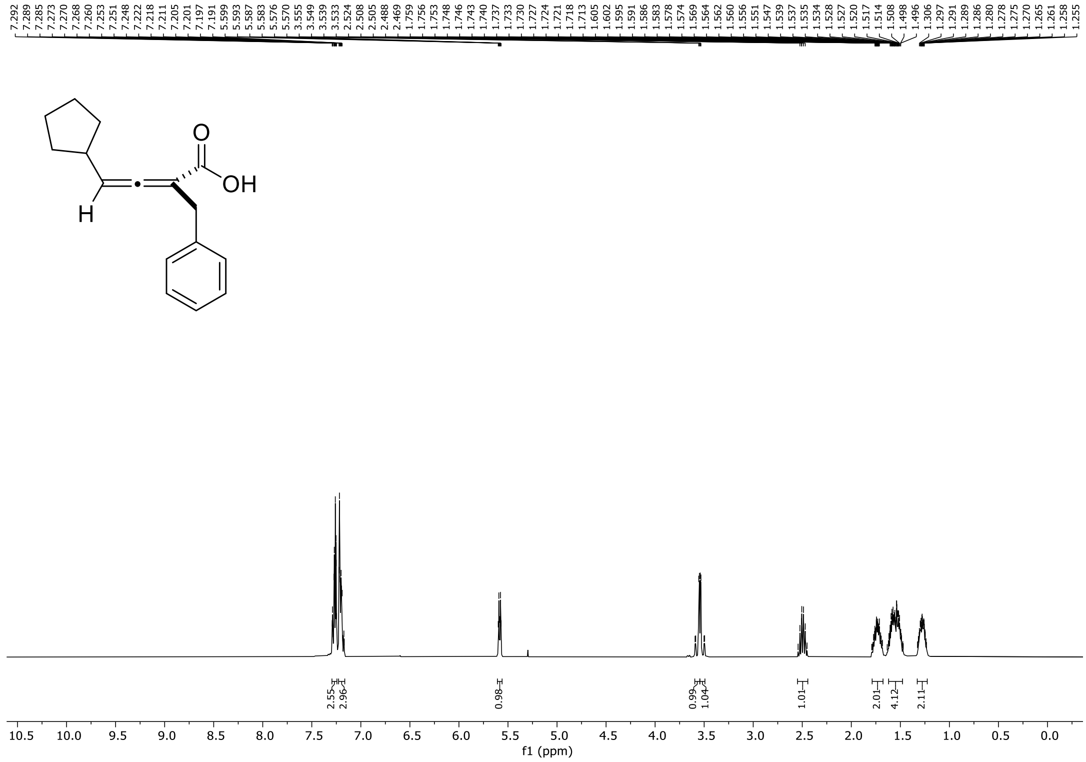

^{13}C NMR (101 MHz, $CDCl_3$, 300 K):

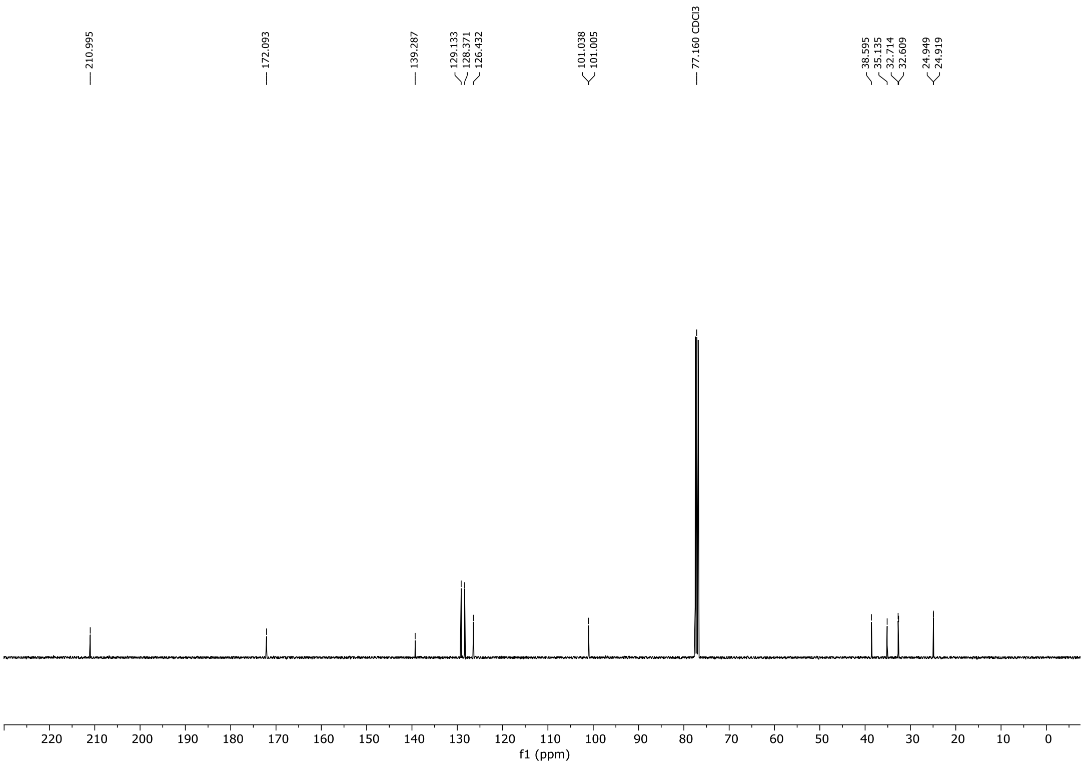

2-Benzyl-5-methylhexa-2,3-dienoic acid (*rac*-1t)

^{1}H NMR (400 MHz, $CDCl_3$, 300 K):

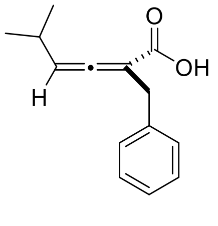

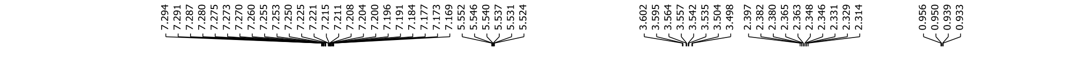

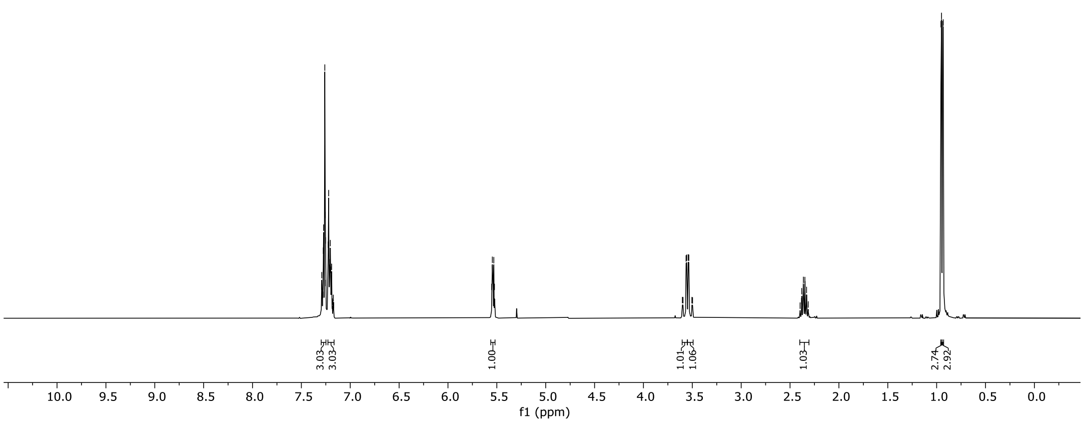

^{13}C NMR (101 MHz, $CDCl_3$, 300 K):

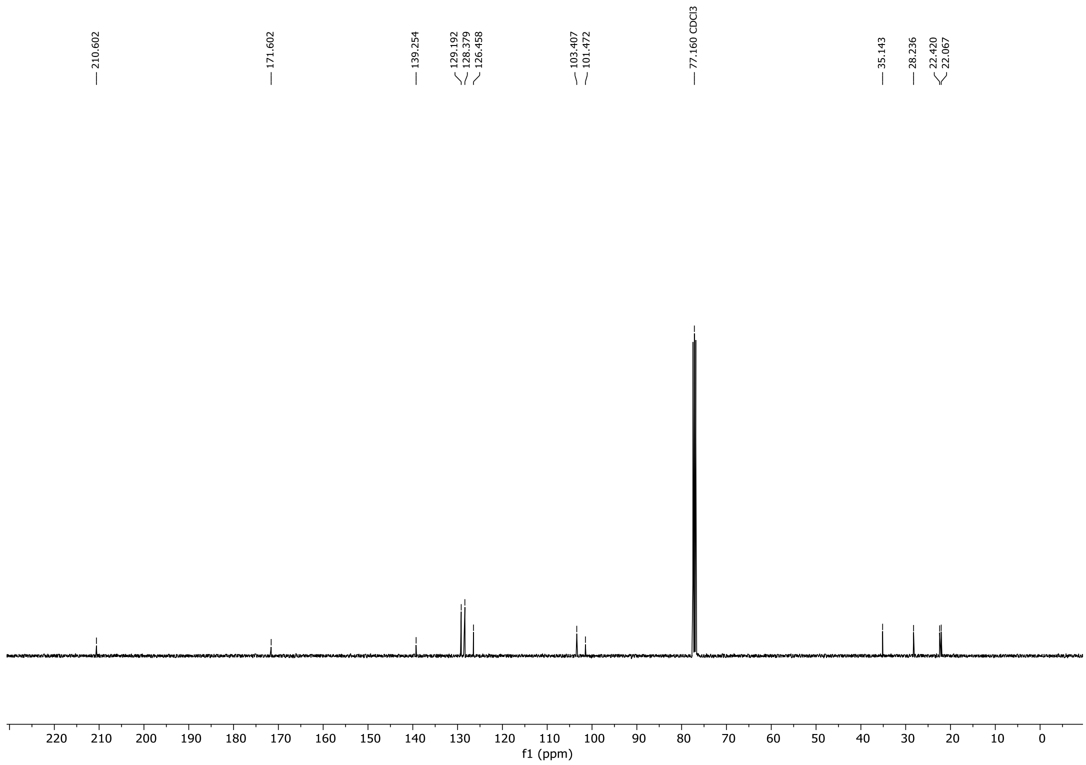

4-(Adamantan-1-yl)-2-benzylbuta-2,3-dienoic acid (*rac*-1u)

^{1}H NMR (400 MHz, $CDCl_3$, 300 K):

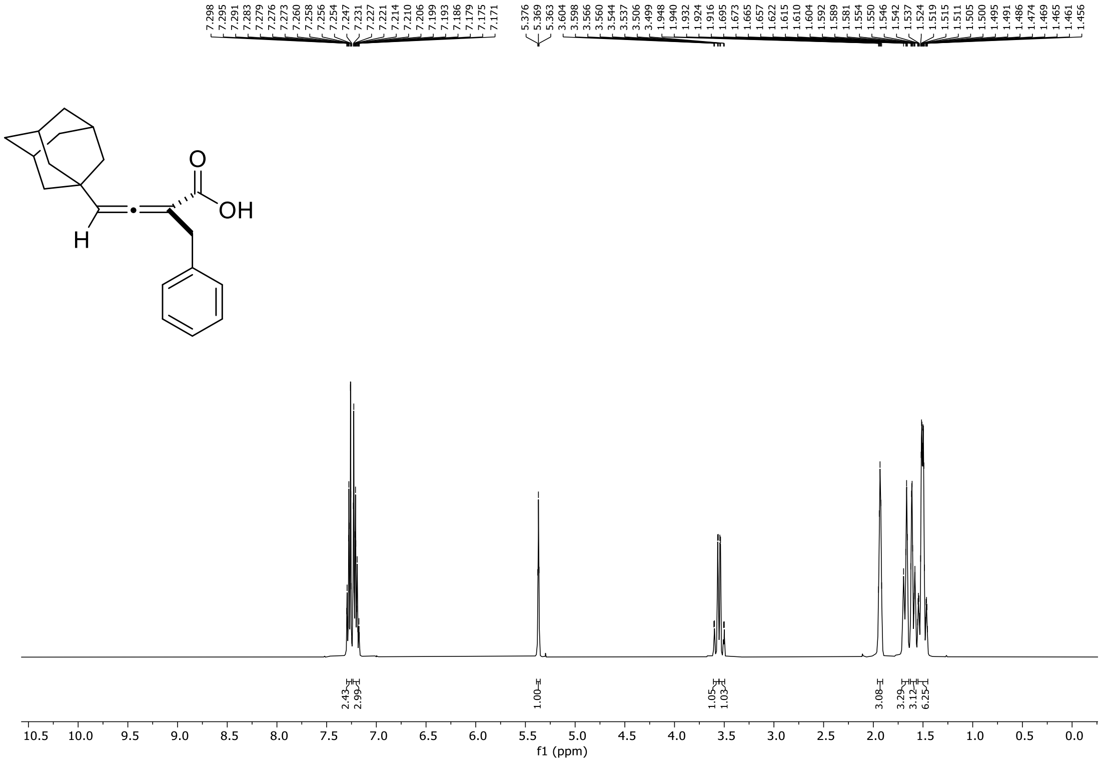

^{13}C NMR (101 MHz, $CDCl_3$, 300 K):

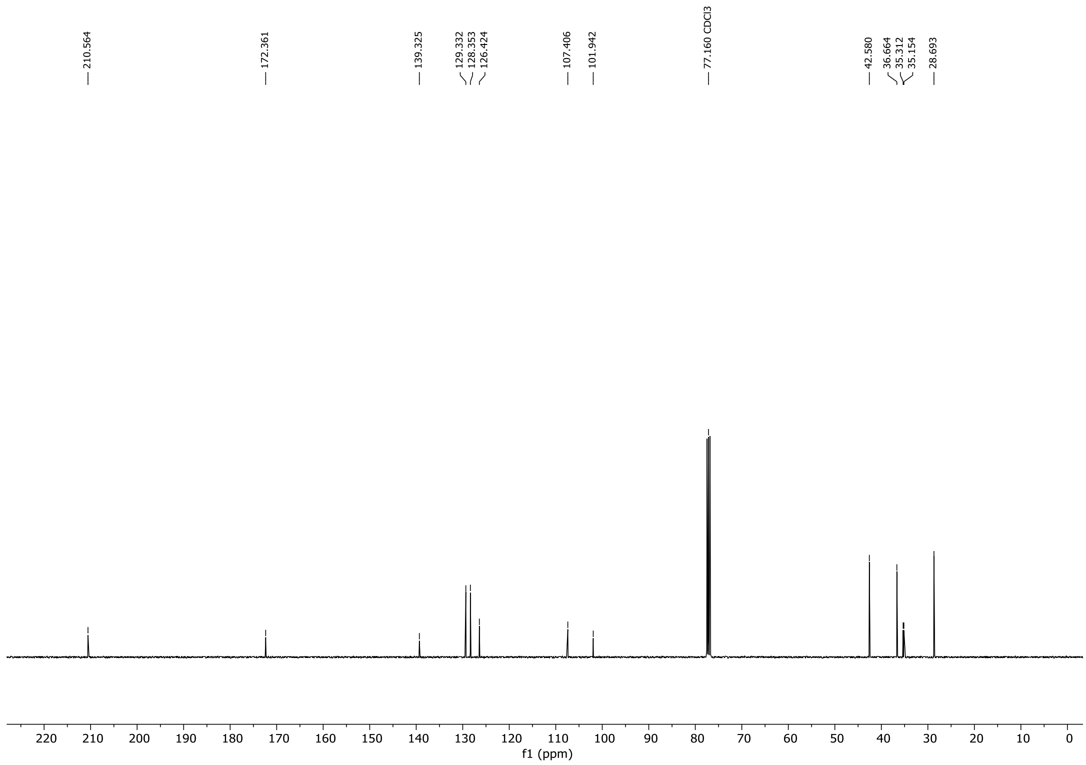

Methyl 4-(adamantan-1-yl)-2-benzylbuta-2,3-dienoate (*rac*-2u)

^{1}H NMR (400 MHz, $CDCl_3$, 300 K):

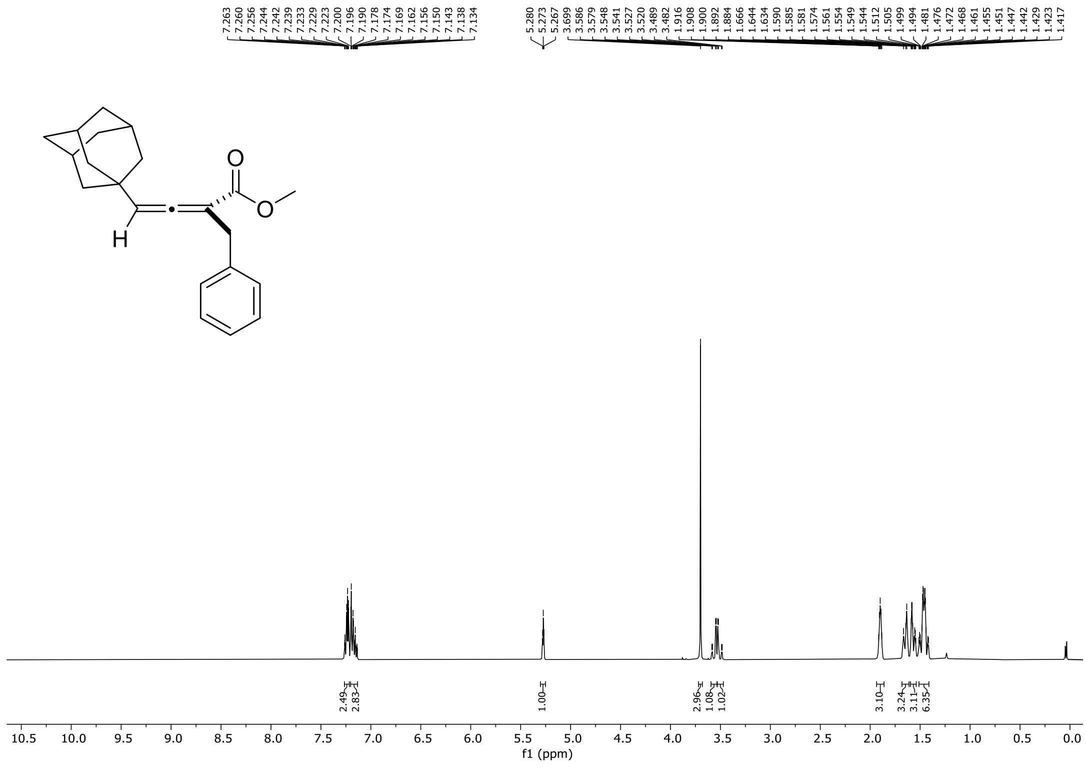

^{13}C NMR (101 MHz, $CDCl_3$, 300 K):

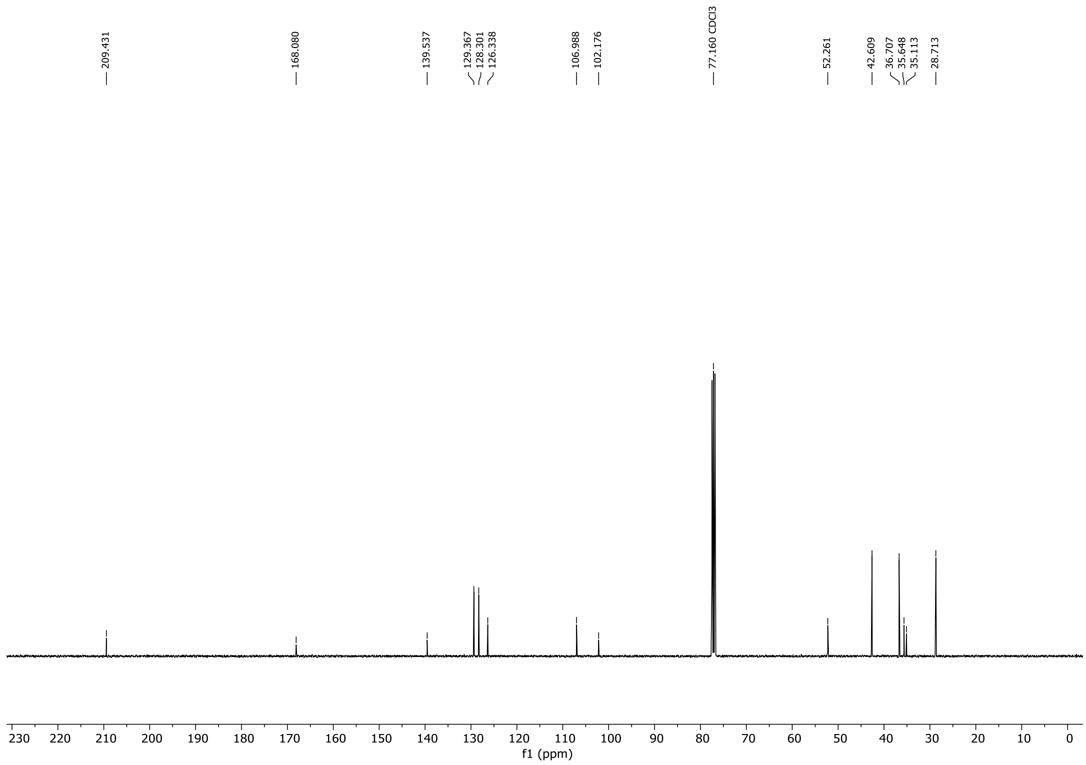

5,5-Dimethylhexa-2,3-dienoic acid (*rac*-1v)

^{1}H NMR (400 MHz, $CDCl_3$, 300 K):

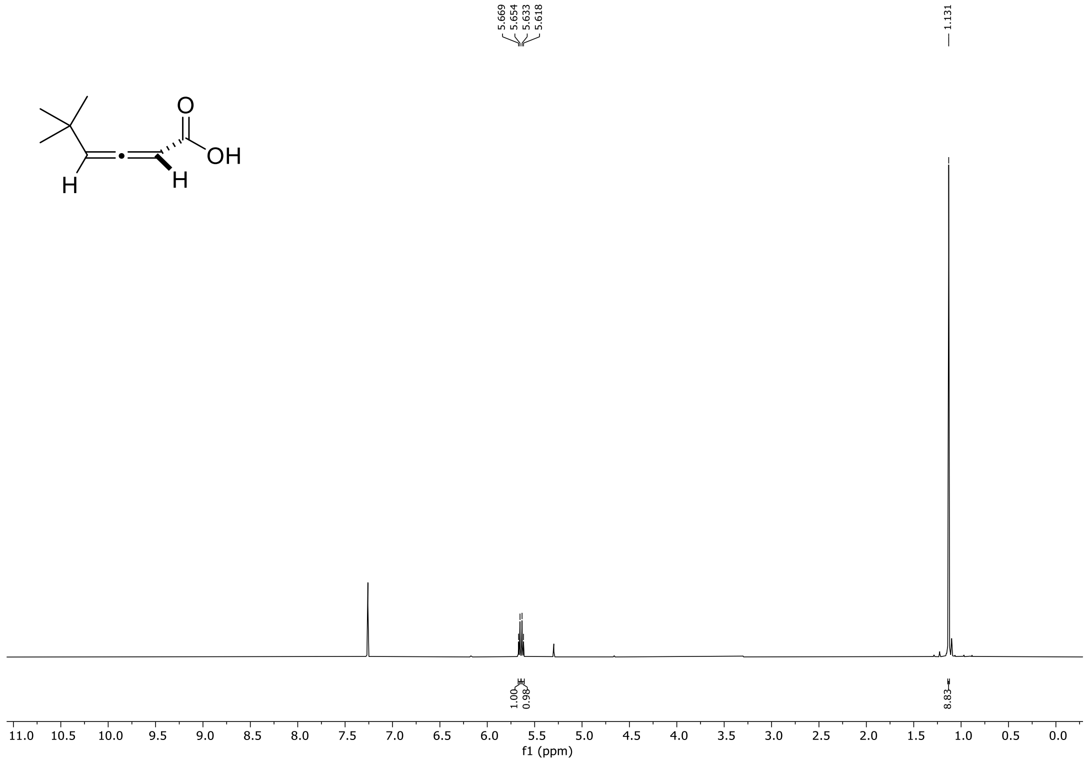

^{13}C NMR (101 MHz, $CDCl_3$, 300 K):

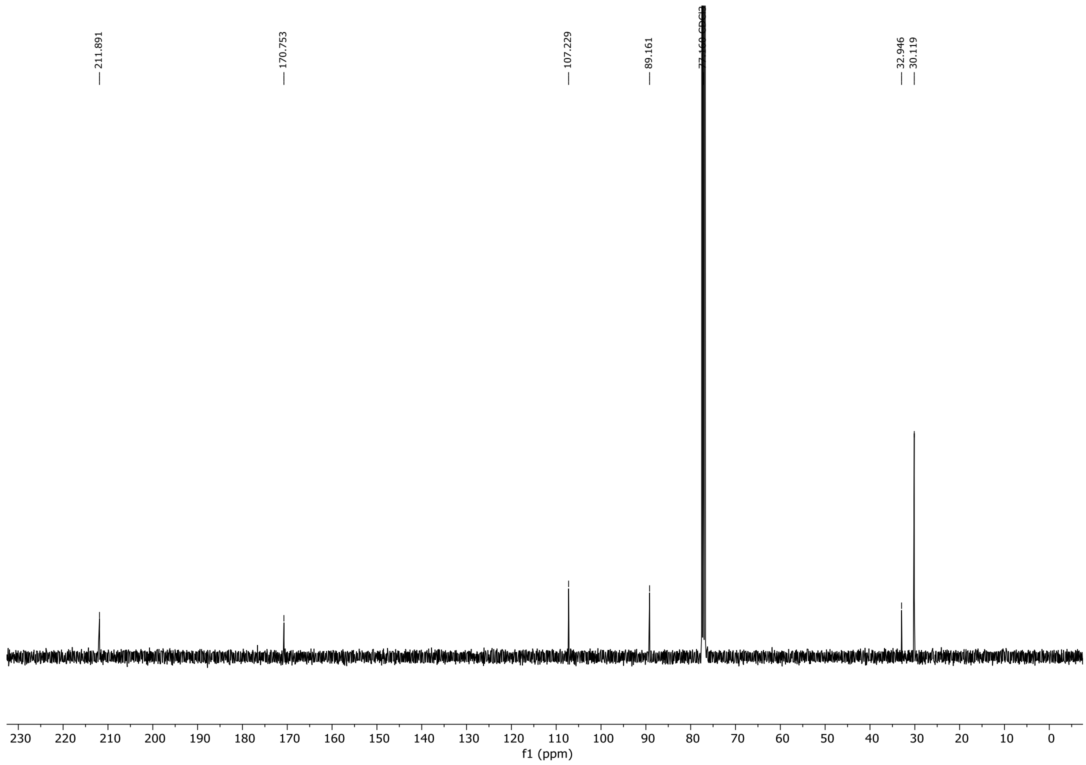

2,5,5-Trimethylhexa-2,3-dienoic acid (*rac*-1w)

^{1}H NMR (400 MHz, $CDCl_3$, 300 K):

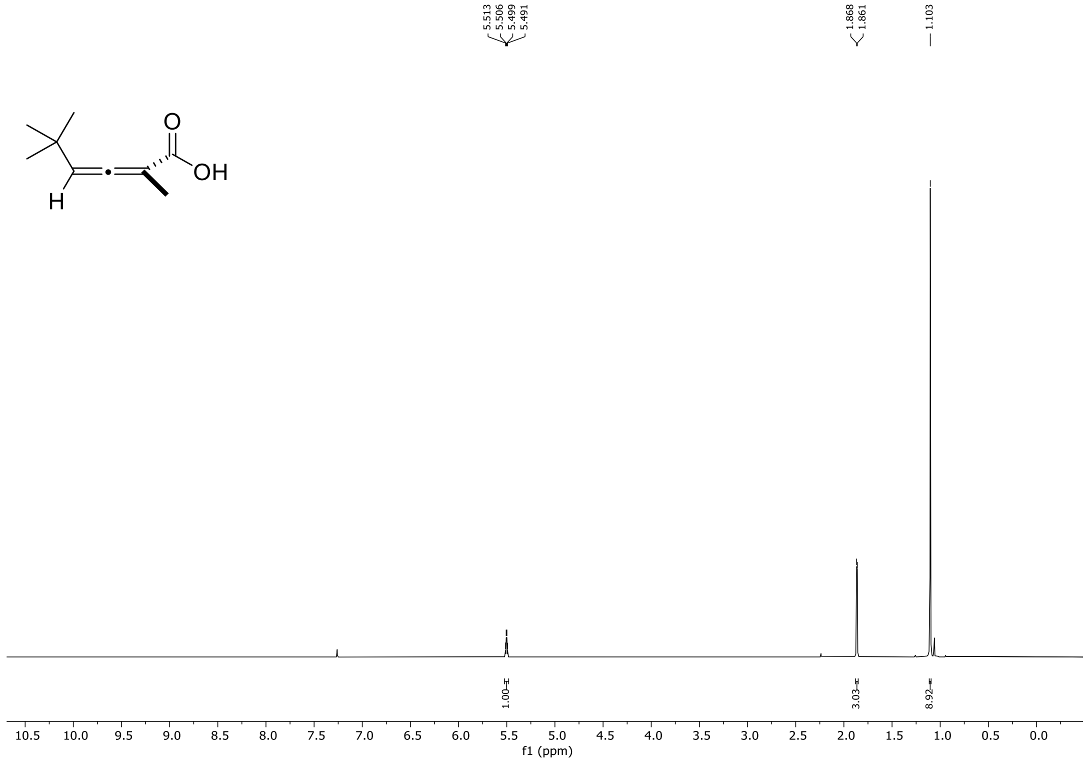

^{13}C NMR (101 MHz, $CDCl_3$, 300 K):

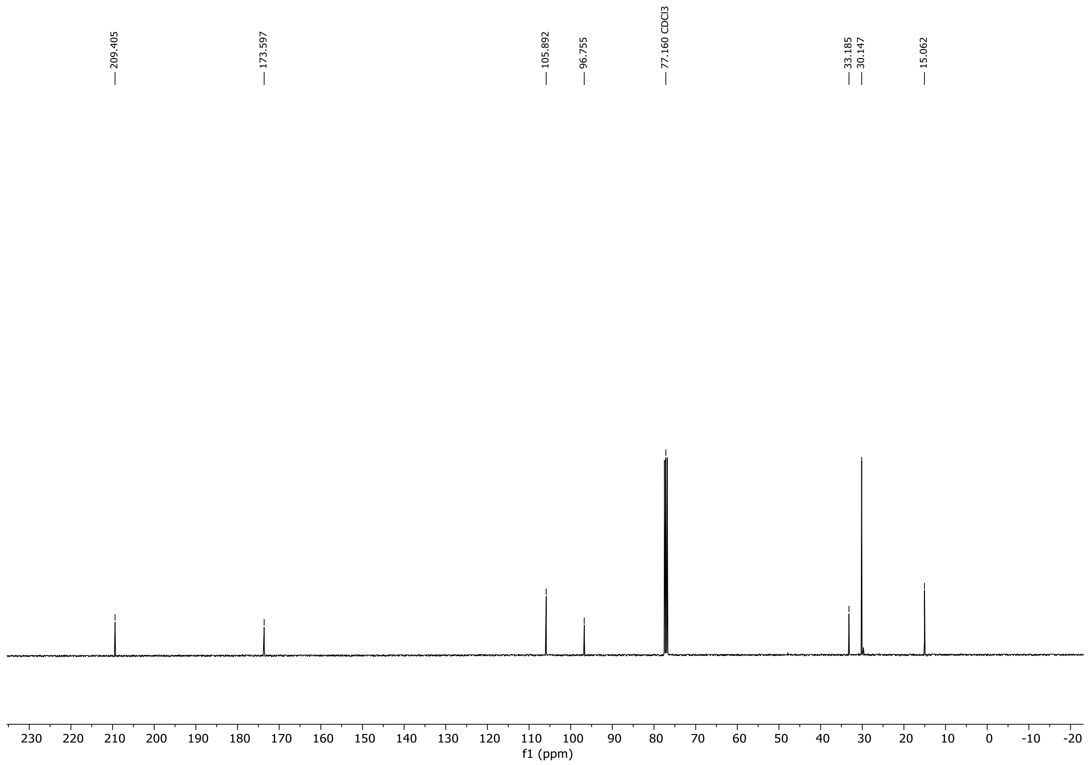

5,5-Dimethyl-2-(3-methylbut-2-en-1-yl)hexa-2,3-dienoic acid (*rac*-1x)

^{1}H NMR (400 MHz, $CDCl_3$, 300 K):

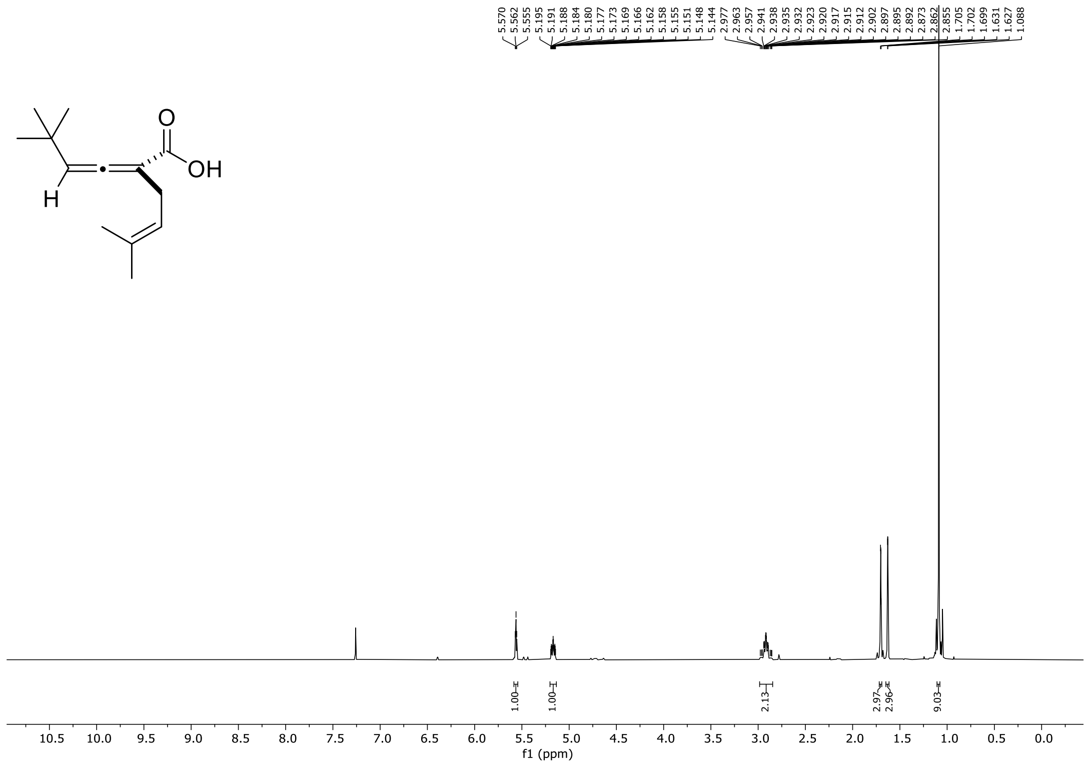

^{13}C NMR (101 MHz, $CDCl_3$, 300 K):

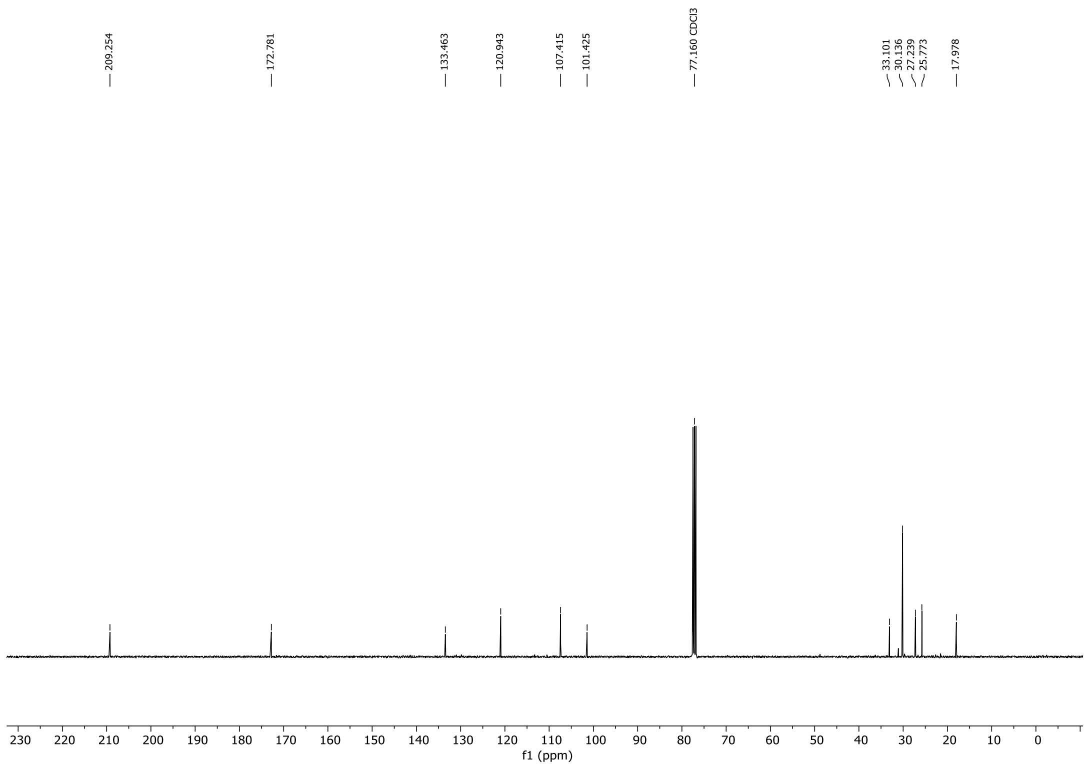

5,5-Dimethyl-2-(3-methylbut-2-en-1-yl)hexa-2,3-dienamide (*rac*-6x)

^{1}H NMR (400 MHz, $CDCl_3$, 300 K):

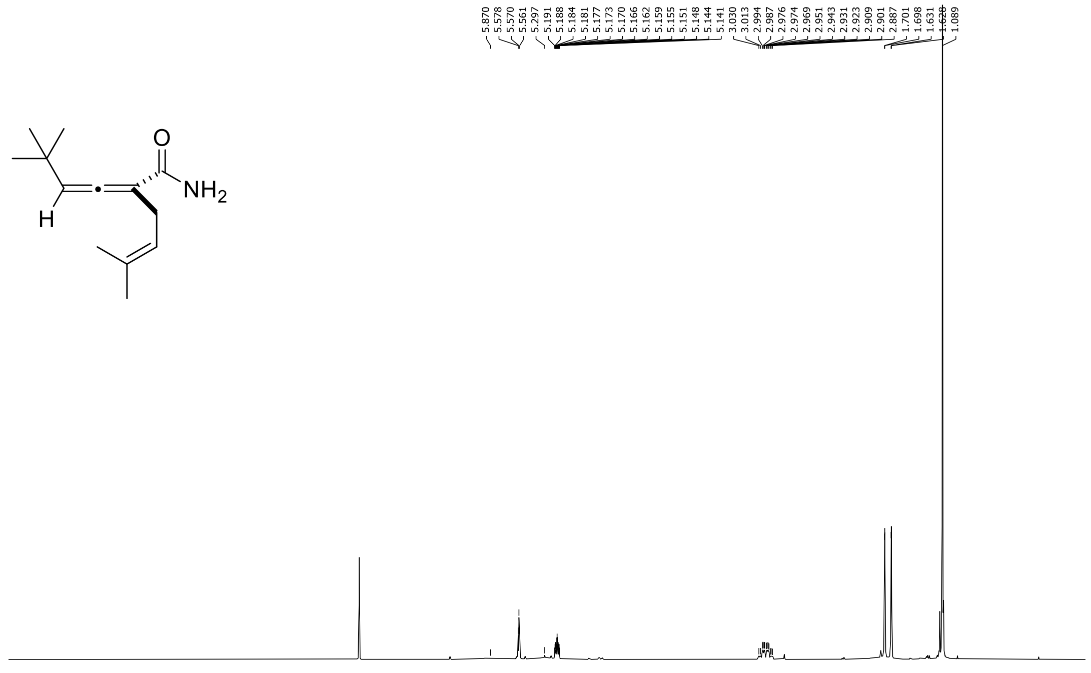

^{13}C NMR (101 MHz, $CDCl_3$, 300 K):

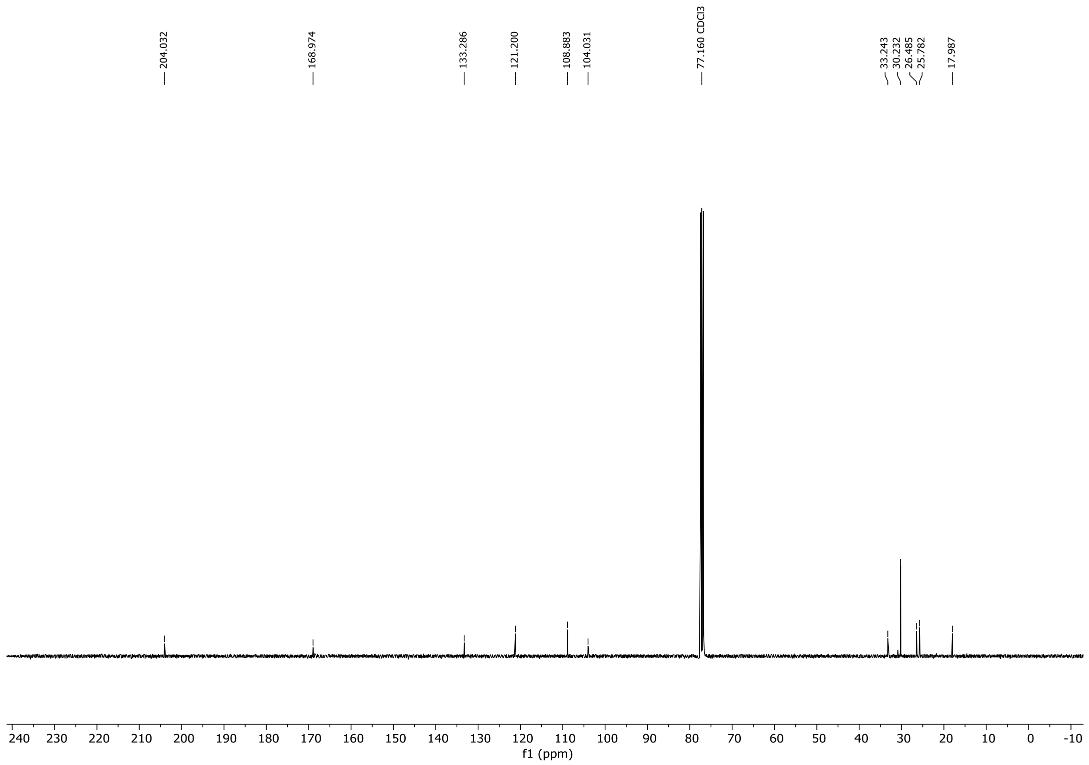

2-Benzyl-5,5-dimethylhexa-2,3-dienamide (*rac*-6c)

^{1}H NMR (400 MHz, $CDCl_3$, 300 K):

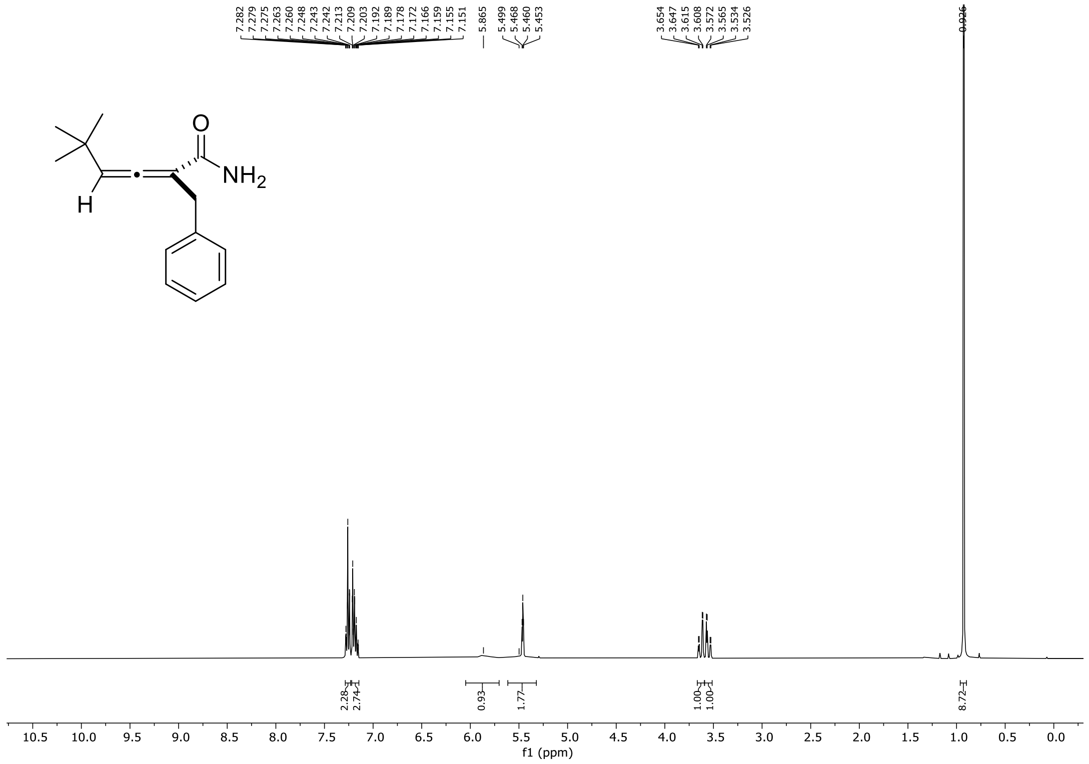

^{13}C NMR (101 MHz, $CDCl_3$, 300 K):

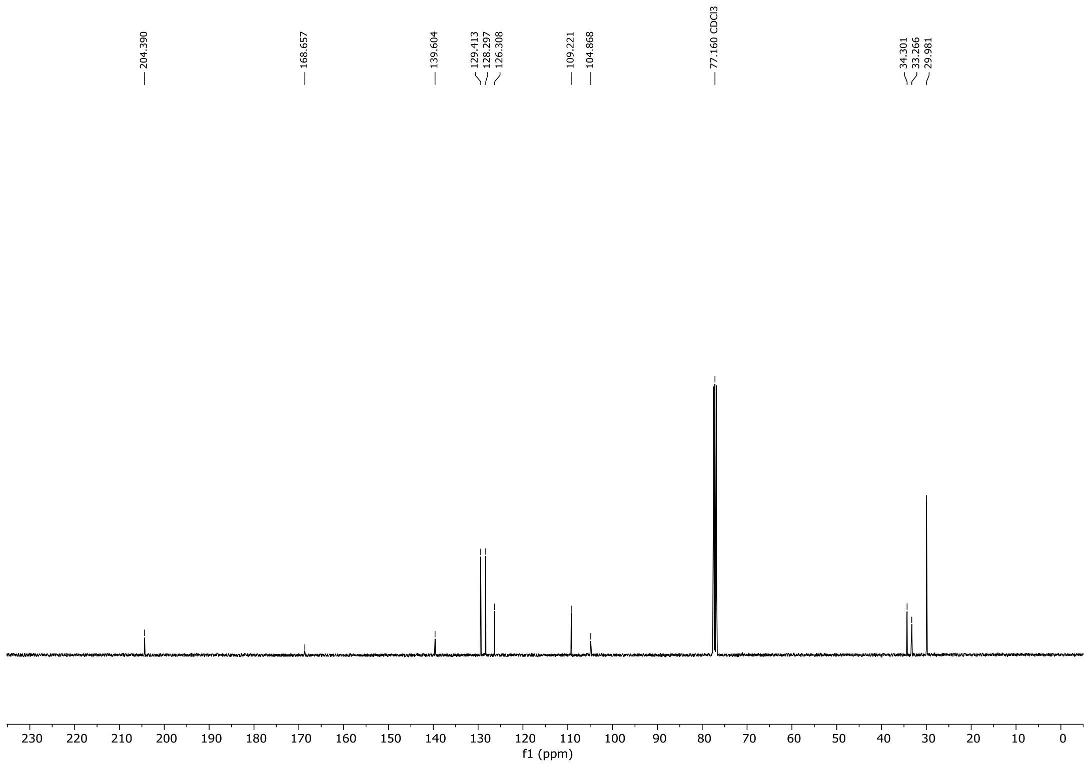

2-(4-Iodobenzyl)-5,5-dimethylhexa-2,3-dienamide (*rac*-6f)

^{1}H NMR (400 MHz, $CDCl_3$, 300 K):

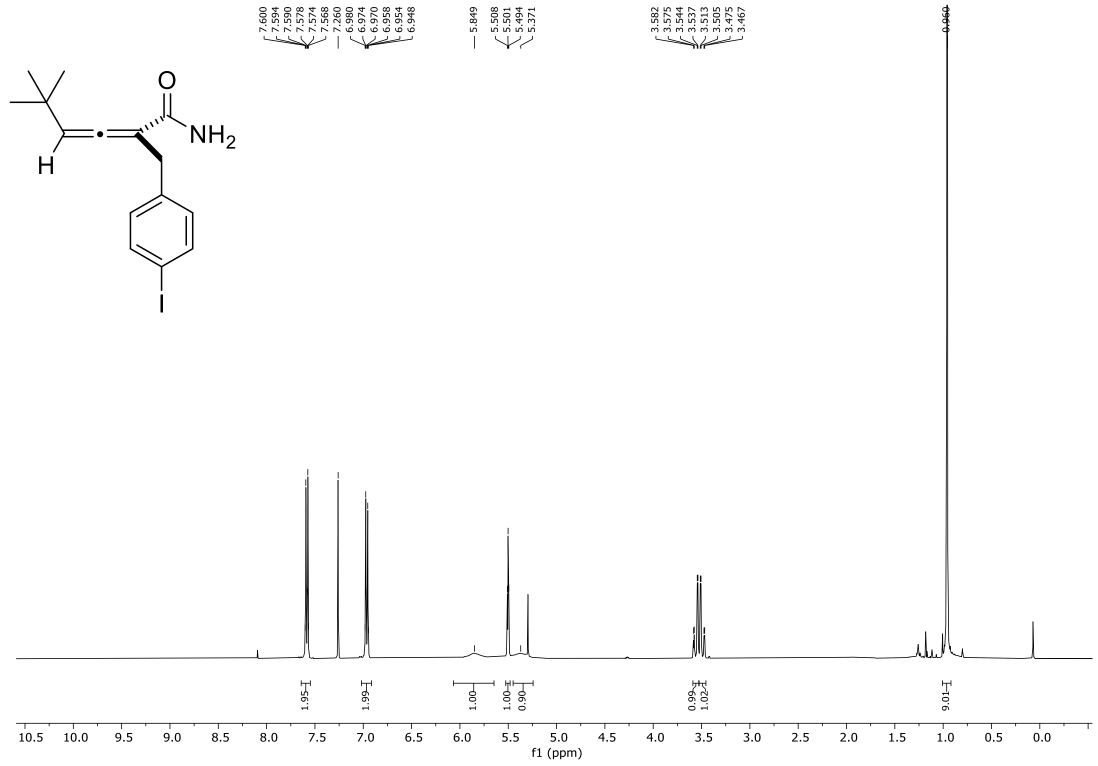

^{13}C NMR (101 MHz, CDCl$_3$, 300 K):

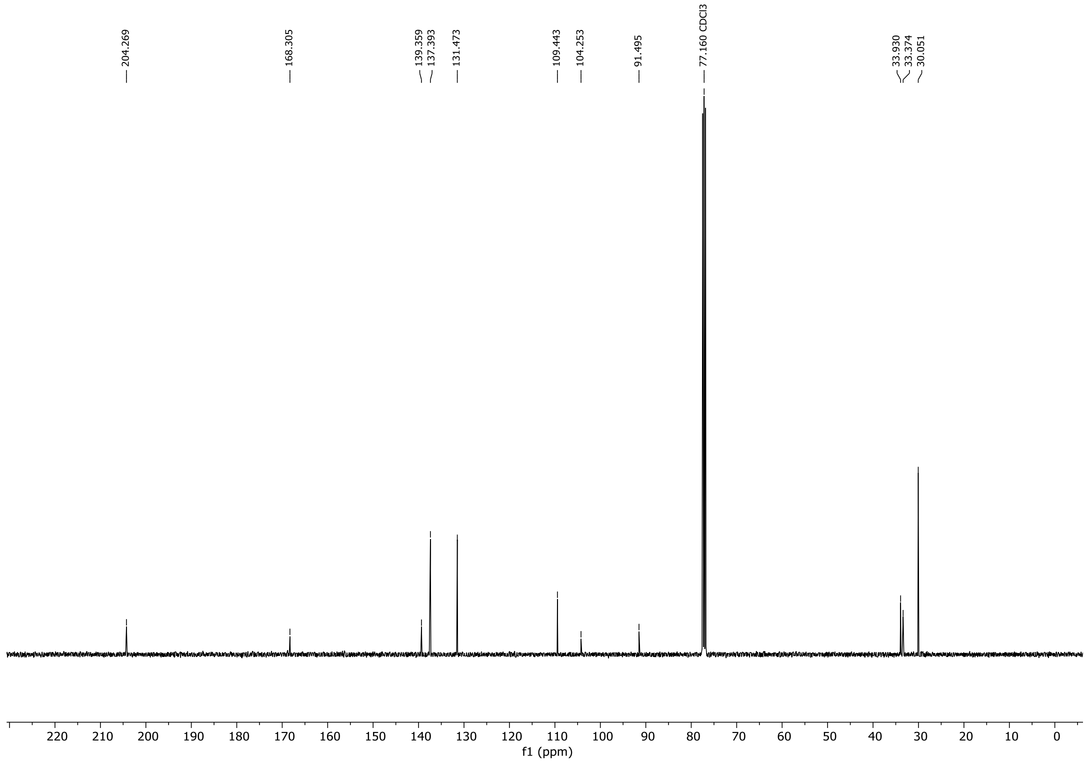

4-Bromo-5-(*tert*-butyl)-3-(4-iodobenzyl)furan-2(5*H*)-one (*rac*-8)

^{1}H NMR (400 MHz, CDCl$_3$, 300 K):

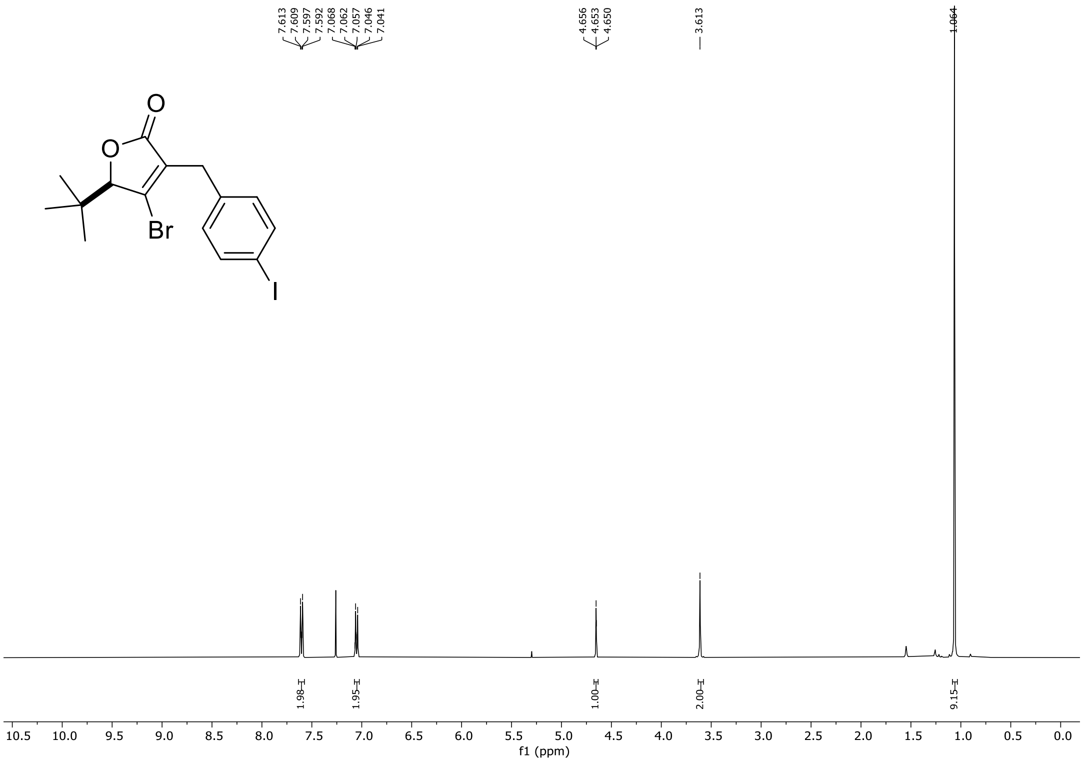

^{13}C NMR (101 MHz, $CDCl_3$, 300 K):

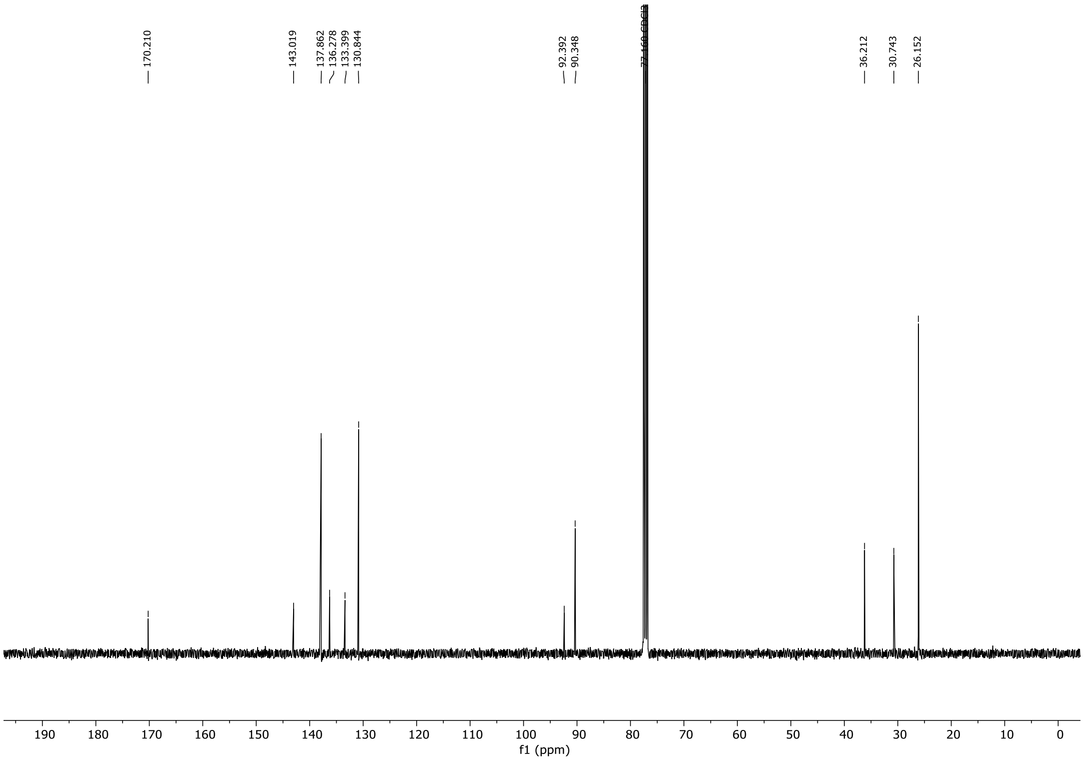

5-(*tert*-Butyl)-4-iodo-3-(4-iodobenzyl)furan-2(5*H*)-one (*rac*-9)

^{1}H NMR (400 MHz, $CDCl_3$, 300 K):

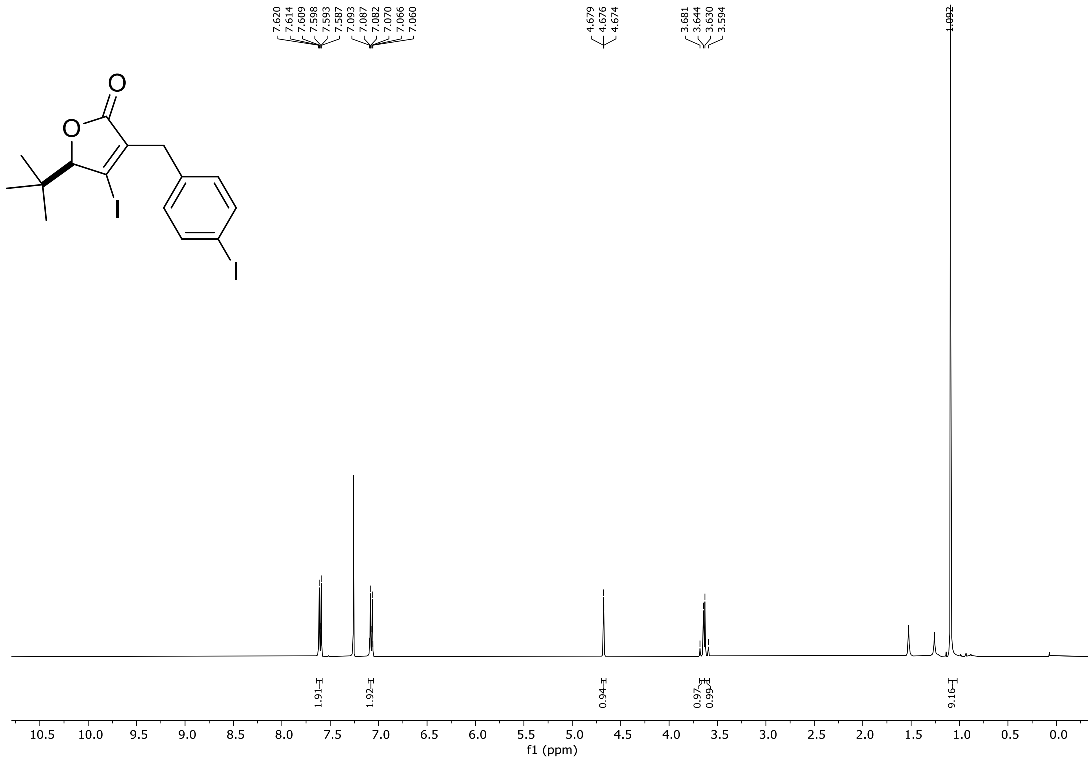

^{13}C NMR (101 MHz, $CDCl_3$, 300 K):

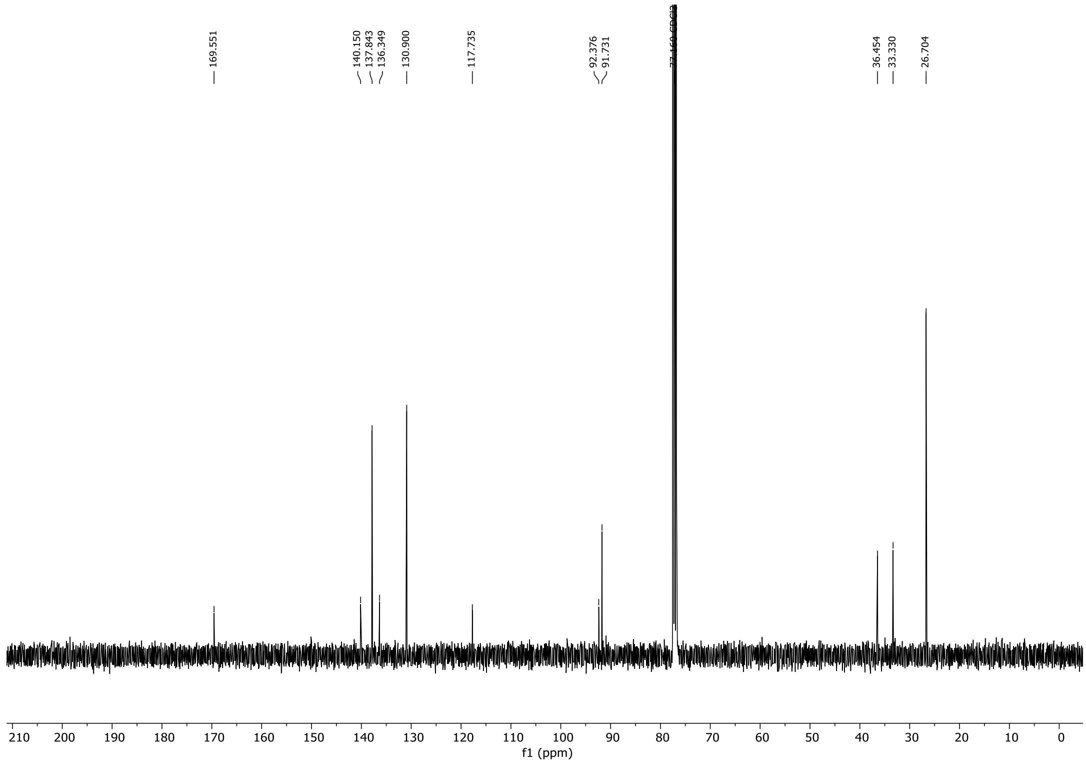

2-(4-Iodobenzyl)-5,5-dimethylhexa-2,3-dien-1-ol (*rac*-10)

^{1}H NMR (400 MHz, $CDCl_3$, 300 K):

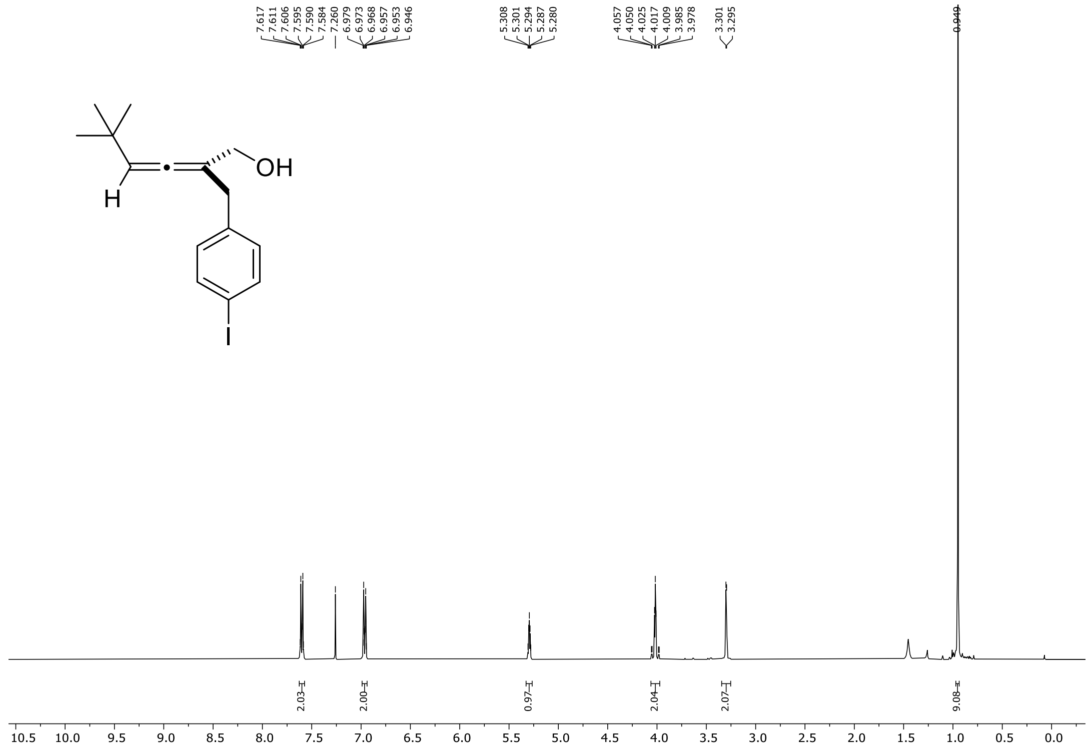

^{13}C NMR (101 MHz, $CDCl_3$, 300 K):

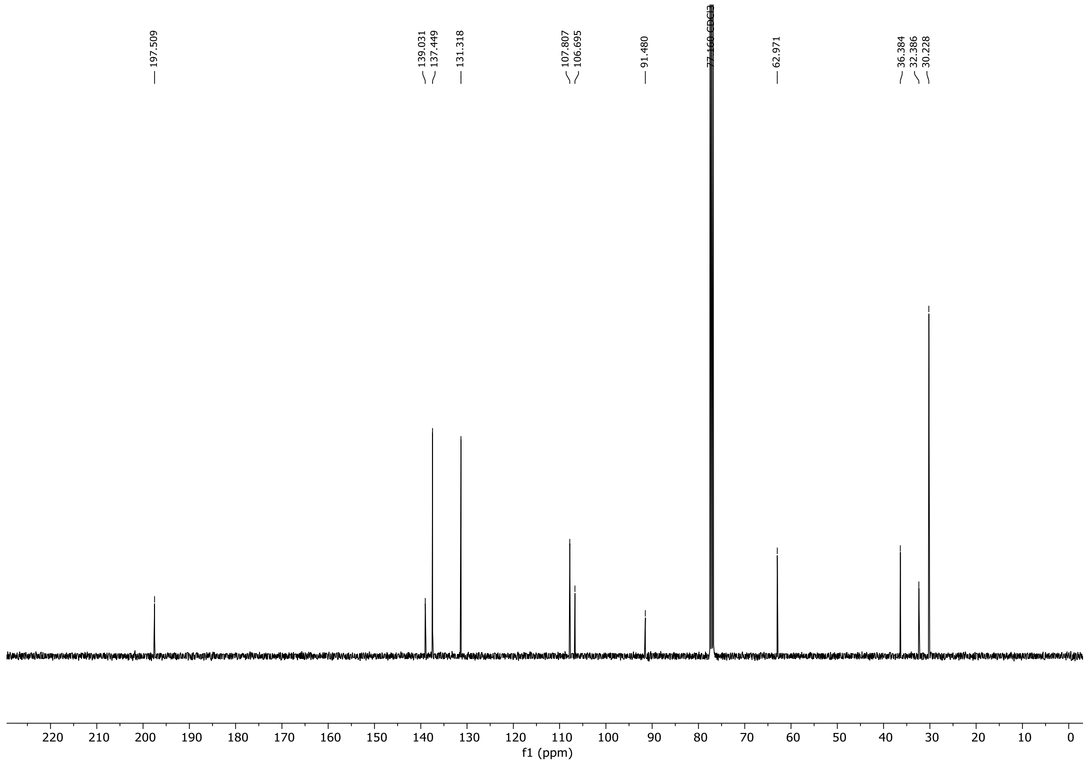

2-(*tert*-Butyl)-3-iodo-4-(4-iodobenzyl)-2,5-dihydrofuran (*rac*-11)

^{1}H NMR (400 MHz, $CDCl_3$, 300 K):

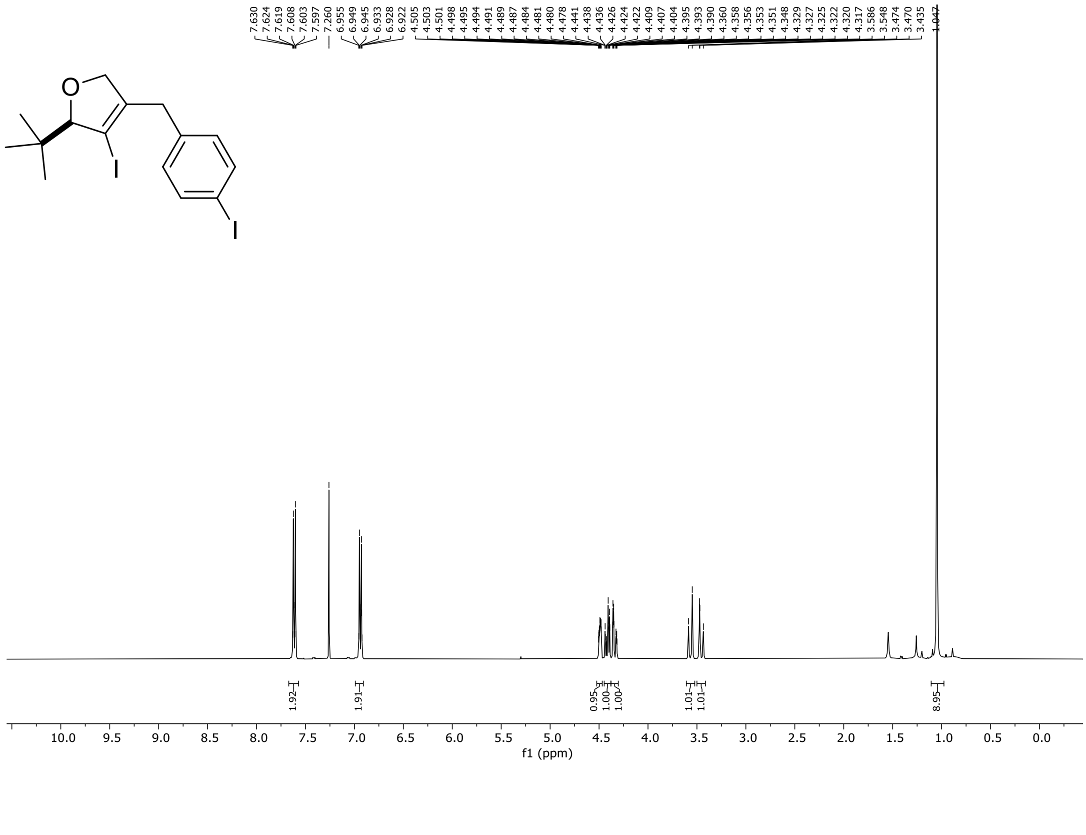

^{13}C NMR (101 MHz, $CDCl_3$, 300 K):

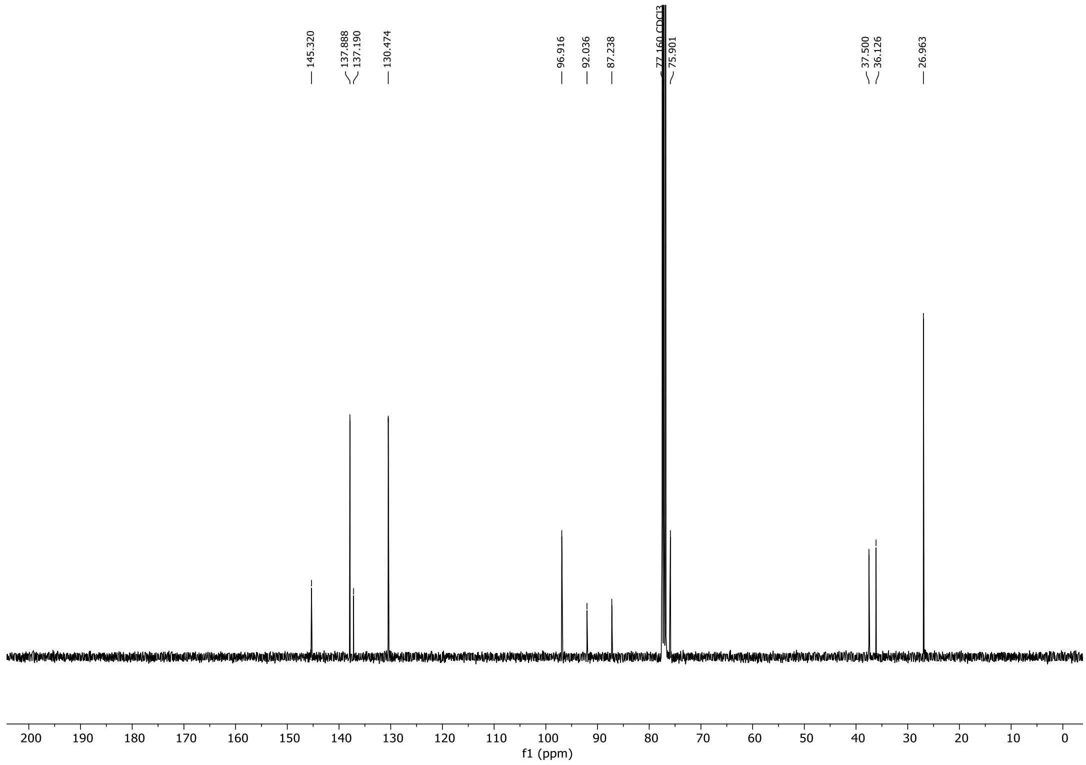

***tert*-Butyl((2-(4-iodobenzyl)-5,5-dimethylhexa-2,3-dien-1-yl)oxy)dimethylsilane (*rac*-12)**

^{1}H NMR (400 MHz, $CDCl_3$, 300 K):

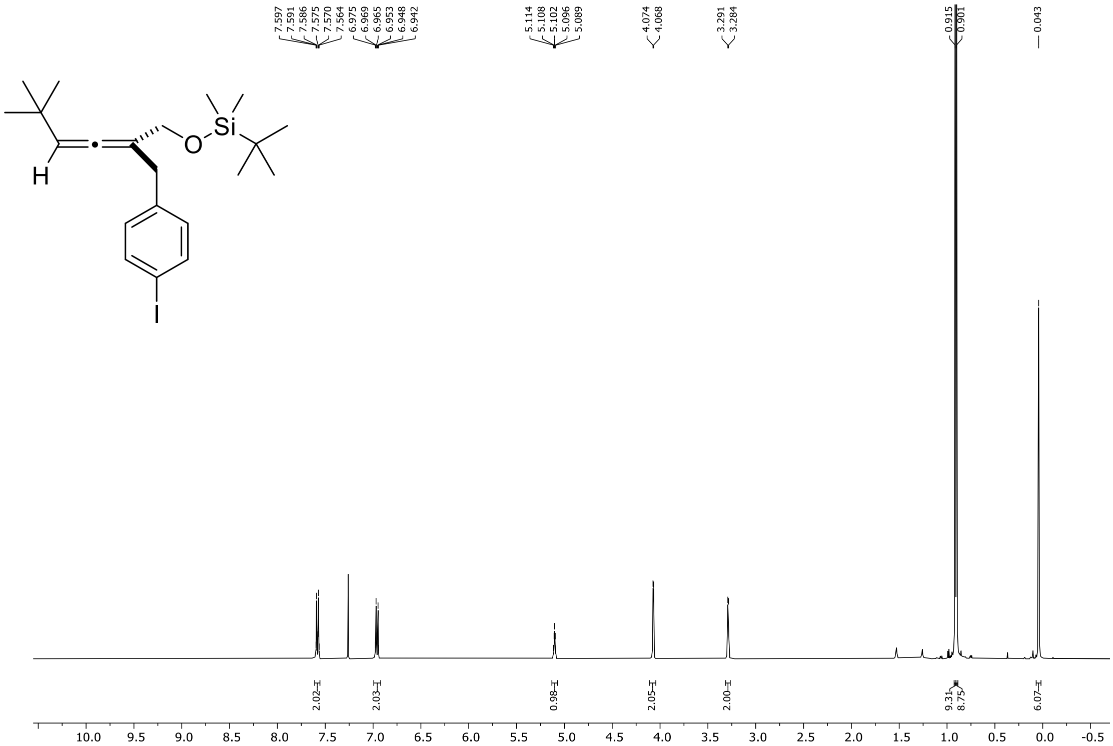

^{13}C NMR (101 MHz, $CDCl_3$, 300 K):

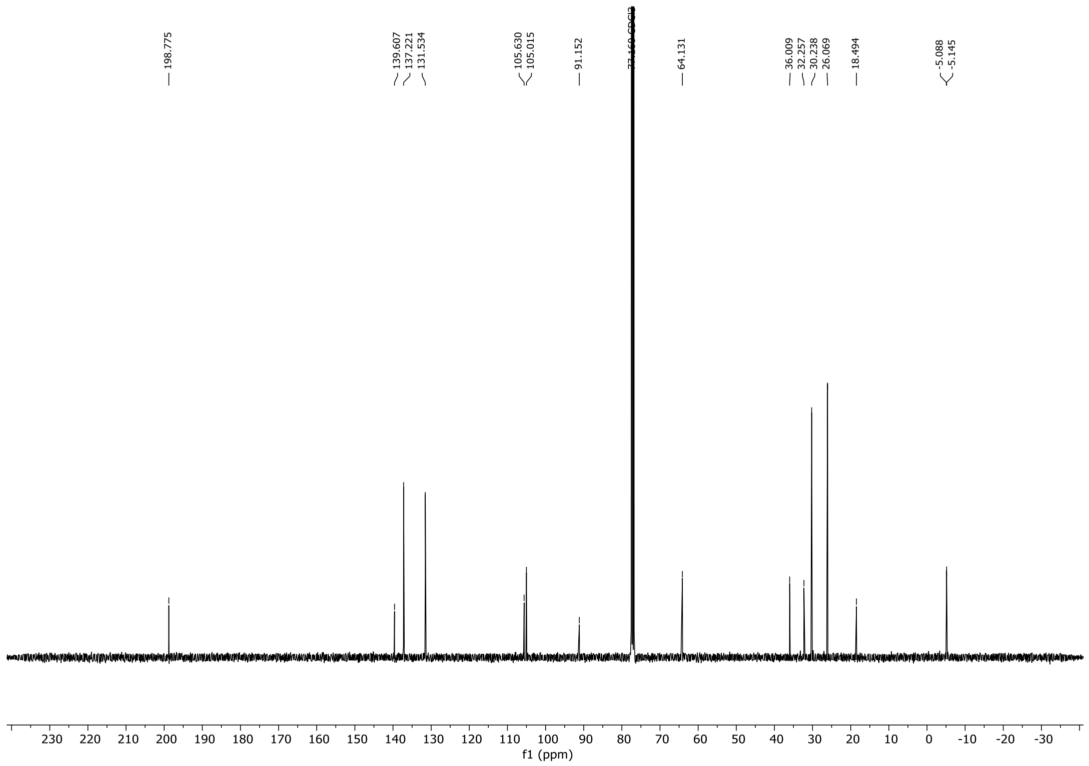

2-Benzyl-4-phenylbuta-2,3-dienoic acid (*rac*-SI-1)

^{1}H NMR (500 MHz, $CDCl_3$, 300 K):

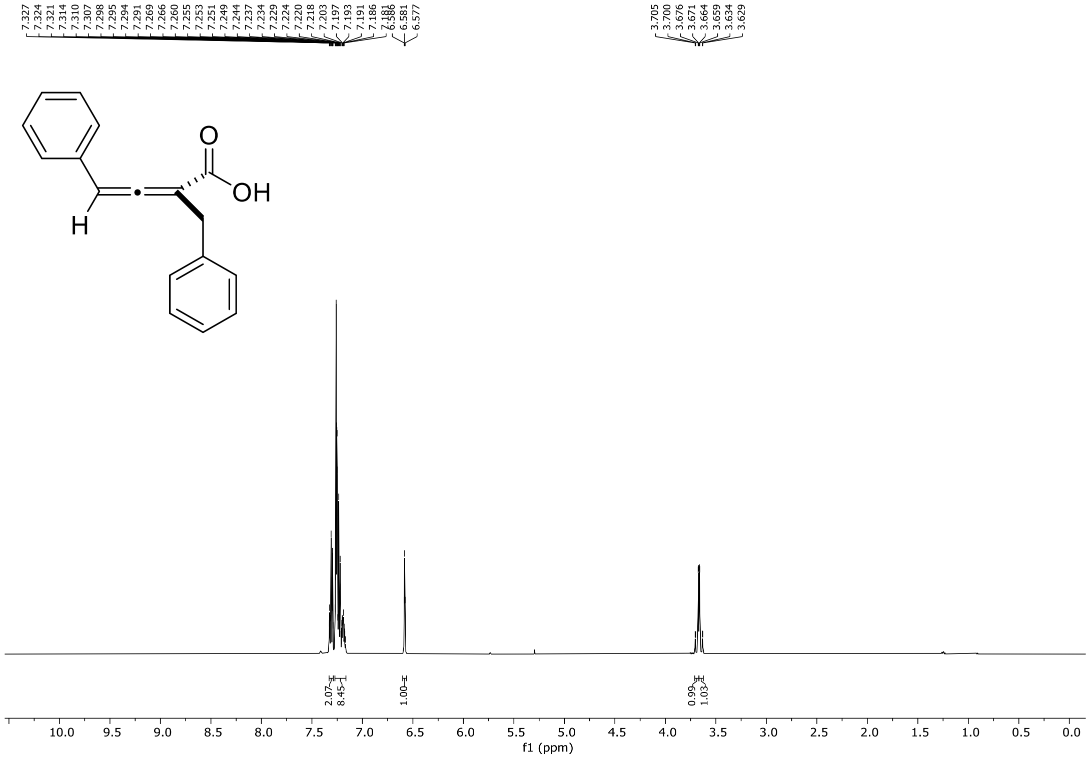

^{13}C NMR (126 MHz, $CDCl_3$, 300 K):

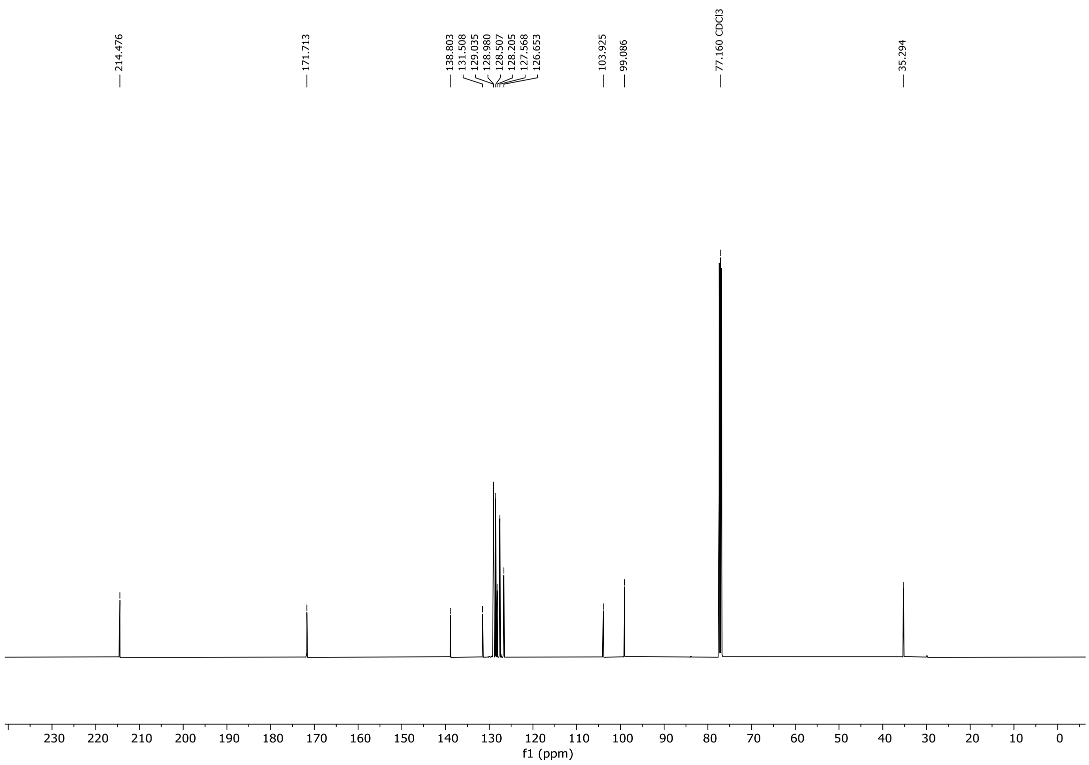

5,5-Dimethyl-2-phenylhexa-2,3-dienoic acid (*rac*-SI-2)

^{1}H NMR (400 MHz, $CDCl_3$, 300 K):

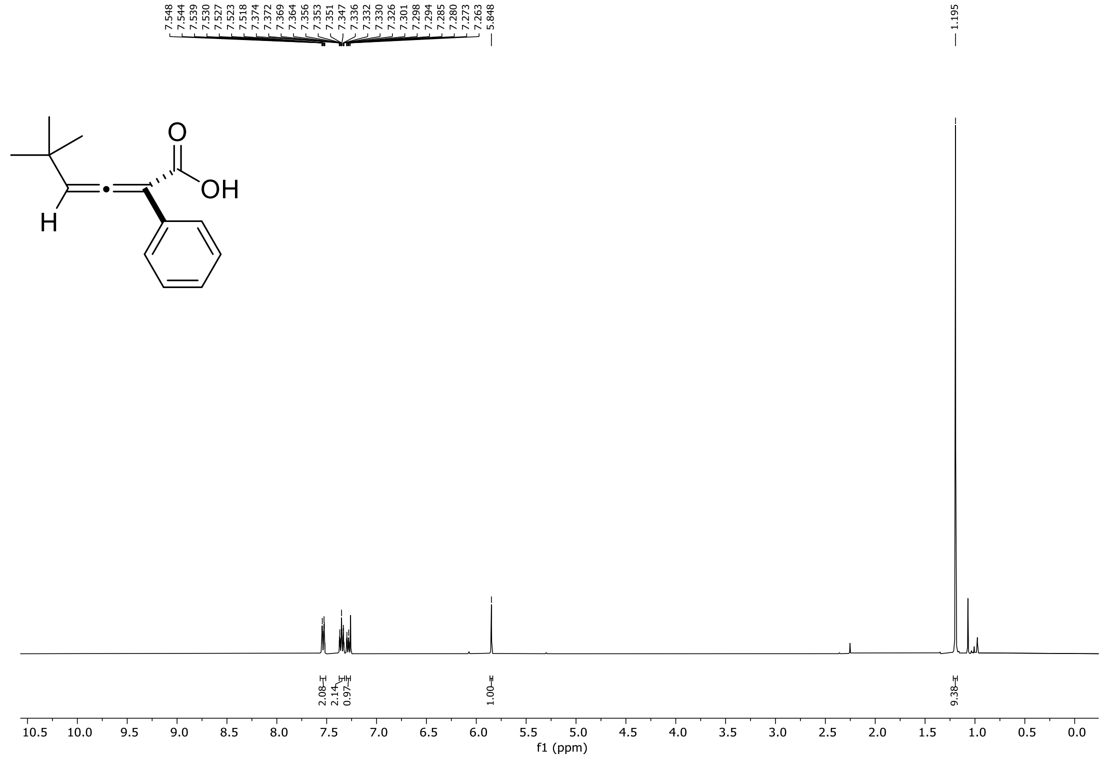

^{13}C NMR (101 MHz, $CDCl_3$, 300 K):

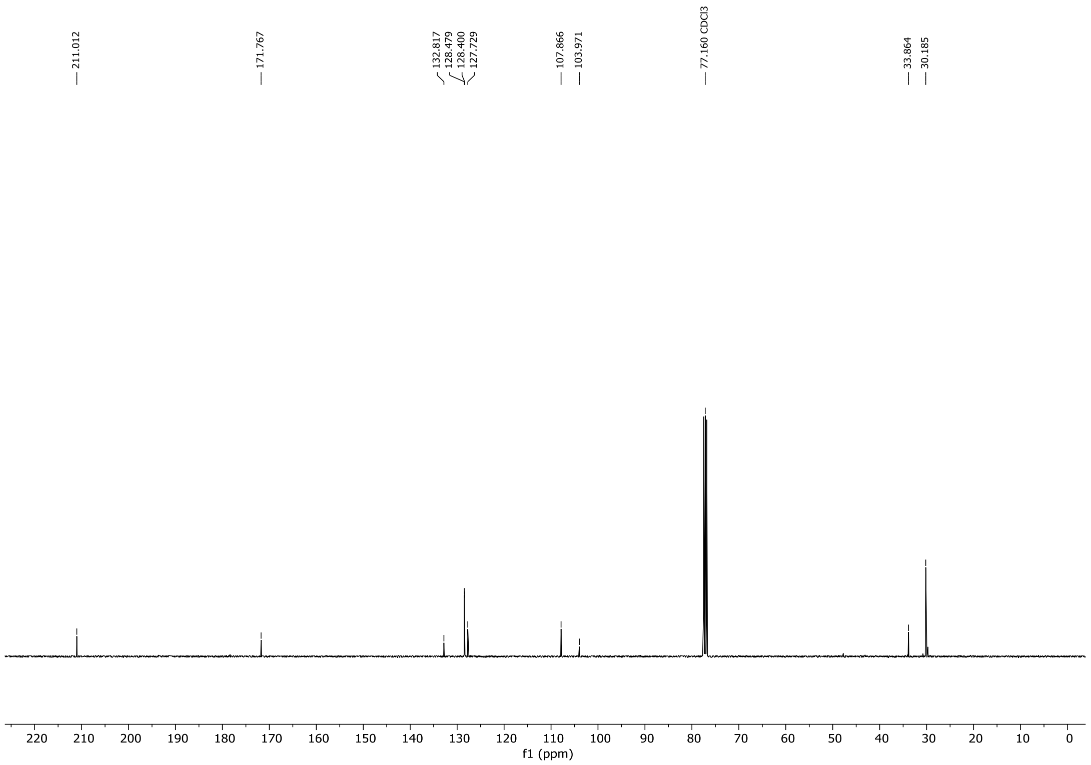

14. Data Sheets of Light Sources

420 nm Reactor

Lehrstuhl OC 1 - TUM

200 nm | 250 nm | 300 nm | 350 nm | 400 nm | 450 nm | 500 nm | 550 nm | 600 nm | 650 nm

Datasheet FLT022 — LZC-420

Basic Information

Type	Fluorescent light tube
Description	Luzchem LZC-420
Manufacturer / Supplier	n/a / Luzchem
Order number / Date of purch.	n/a / 07/2017
Internal lot / serial number	2017-07 / FLT022

Specification Manufacturer

Type / size	T5 tube, G5 socket
Mechanical specification	16 mm diameter, 288 mm length
Electrical specification	8 W
Wavelength (range, typ.)	400 - 440 nm
Spectral width (FWHM)	~ 30 nm
Datasheet	LES-420-016

Characterization

Description of measurement	Measured with Ocean-optics USB4000 spectrometer using a calibrated setup (cosine corrector/fibre). The cosine corrector was placed at 20 mm distance from a single fluorescent tube at half height.	
Measured dominant wavelength / Int.	421 nm	121 µW/mm²nm
Measured spectral width (FWHM)	30 nm	
Integral Reference intensity / range	4142 µW/cm²	350-500 nm

Spectrum

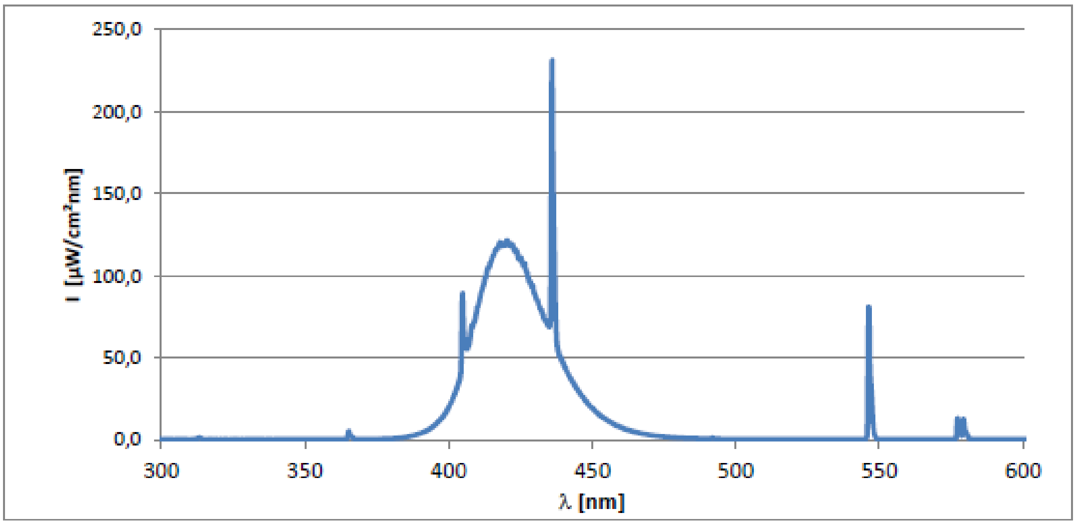

440 nm LED

Lehrstuhl OC 1 - TUM

200 nm | 250 nm | 300 nm | 350 nm | 400 nm | 450 nm | 500 nm | 550 nm | 600 nm | 650 nm

Datasheet LED053 Av-440-3W

Basic Information

Type	High-Power-LED	
Description	Avonec 440-450 nm / 3 W	
Manufacturer / Supplier	n/a / Avonec	
Order number / Date of purch.	n/a / 07/2017	
Internal lot / serial number	2017-01 / LED053	

Specification Manufacturer

Type / size	single emitter / ca. 1 x 1 mm	
Mechanical specification		
Electrical specification	700 mA, UF 3.7 V	
Wavelength (range, typ.)	440-450 nm, typ. n/a	
Spectral width (FWHM)	n/a	
Datasheet	n/a	

Characterization

Description of measurement	Measured with Ocean-optics USB4000 spectrometer using a calibrated setup (cosine corrector/fibre). The distance between the emitting surface and the surface of the cosine corrector was 20 mm. The LED was operated at 700 mA on a passive heat-sink at approx. 20 °C	
Measured dominant wavelength / Int.	440 nm	2098 µW/mm²nm
Measured spectral width (FWHM)	18 nm	
Integral Reference intensity / range	46715 µW/cm²	400-500 nm

Spectrum

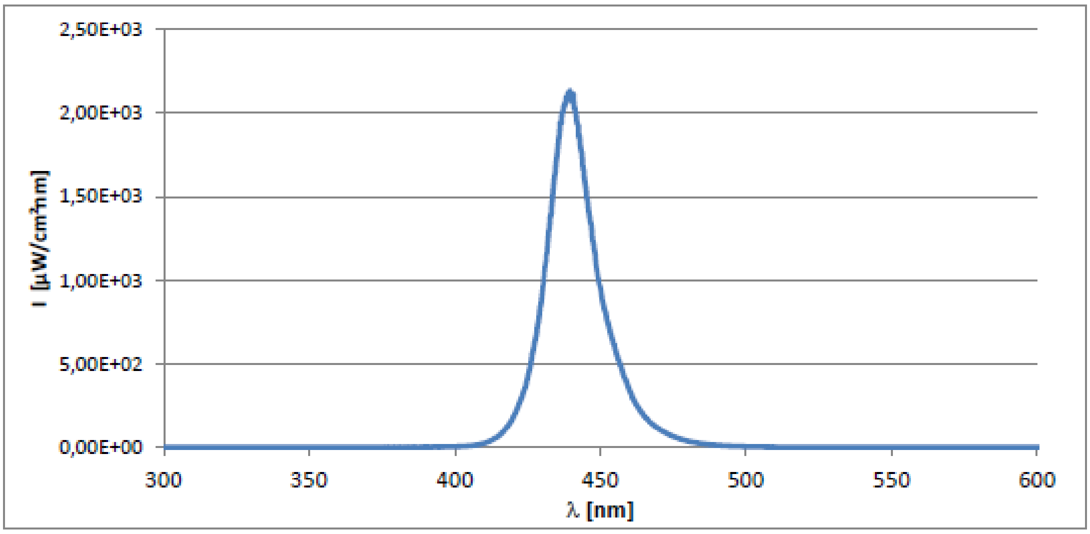

15. References

[1] S. Poplata, T. Bach, *J. Am. Chem. Soc.* **2018**, *140*, 3228.

[2] a) F. Pecho, Y.-Q. Zou, J. Gramüller, T. Mori, S. M. Huber, A. Bauer, R. M. Gschwind, T. Bach, *Chem. Eur. J.* **2020**, *26*, 5190; b) M. Stierle, C. Jaschke, D. J. Grenda, M. T. Peschel, T. Pickl, N. Gessner, P. Nuernberger, B. P. Fingerhut, C. Ochsenfeld, R. de Vivie-Riedle, T. Bach, *Angew. Chem. Int. Ed.* **2025**, *64*, e202501433.

[3] X. Xiong, T. Zheng, X. Wang, Y.-L. S. Tse, Y.-Y. Yeung, *Chem* **2020**, *6*, 919.

[4] Y. Yang, S.-F. Zhu, H.-F. Duan, C.-Y. Zhou, L.-X. Wang, Q.-L. Zhou, *J. Am. Chem. Soc.* **2007**, *129*, 2248.

[5] R. Takagi, T. Tanimoto, *Org. Biomol. Chem.* **2022**, *20*, 3940.

[6] X. Dong, S. Shen, Y. Qin, X. Hu, H. Gao, G. Liu, T. Gao, Z. Pang, P. Wang, Y. Wang, *Chin. Chem. Lett.* **2023**, *34*, 108311.

[7] M. Plaza, J. Großkopf, S. Breitenlechner, C. Bannwarth, T. Bach, *J. Am. Chem. Soc.* **2021**, *143*, 11209.

[8] a) P. Pracht, S. Grimme, C. Bannwarth, F. Bohle, S. Ehlert, G. Feldmann, J. Gorges, M. Müller, T. Neudecker, S. Spicher, P. Steinbach, P. A. Wesołowski, F. Zeller, *J. Chem. Phys.* **2024**, *160*; b) S. Grimme, *J. Chem. Theory Comput.* **2019**, *15*, 2847.

[9] C. Bannwarth, S. Ehlert, S. Grimme, *J. Chem. Theory Comput.* **2019**, *15*, 1652.

[10] S. Ehlert, M. Stahn, S. Spicher, S. Grimme, *J. Chem. Theory Comput* **2021**, *17*, 4250.

[11] P. Pracht, F. Bohle, S. Grimme, *Phys. Chem. Chem. Phys.* **2020**, *22*, 7169.

[12] S. Grimme, J. G. Brandenburg, C. Bannwarth, A. Hansen, *J. Chem. Phys.* **2015**, *143*, 54107.

[13] S. Grimme, S. Ehrlich, L. Goerigk, *J. Comput. Chem.* **2011**, *32*, 1456.

[14] S. Grimme, J. Antony, S. Ehrlich, H. Krieg, *J. Chem. Phys.* **2010**, *132*, 154104.

[15] H. Kruse, S. Grimme, *J. Chem. Phys.* **2012**, *136*, 154101.

[16] a) V. Barone, M. Cossi, *J. Phys. Chem. A* **1998**, *102*, 1995; b) M. Garcia-Ratés, F. Neese, *J. Comput. Chem.* **2020**, *41*, 922.

[17] a) Y. Zhao, D. G. Truhlar, *J. Phys. Chem. A* **2005**, *109*, 5656; b) F. Weigend, R. Ahlrichs, *Phys. Chem. Chem. Phys.* **2005**, *7*, 3297.

[18] F. Neese, *WIREs Comput. Mol. Sci.* **2025**, *15*.

[19] a) F. Weigend, *Phys. Chem. Chem. Phys.* **2006**, *8*, 1057; b) F. Neese, *J. Comput. Chem.* **2003**, *24*, 1740.

[20] C. Bannwarth, E. Caldeweyher, S. Ehlert, A. Hansen, P. Pracht, J. Seibert, S. Spicher, S. Grimme, *WIREs Comput. Mol. Sci.* **2021**, *11*.

[21] S. Alvarez, *Dalton Trans.* **2013**, *42*, 8617.

[22] J. Lyu, M. Leone, A. Claraz, C. Allain, L. Neuville, G. Masson, *RSC Adv.* **2021**, *11*, 36663.

[23] S. Ma, Z. Shi, Z. Yu, *Tetrahedron Lett.* **1999**, *40*, 2393.

[24] F. M. Harvey, A. H. Heidecker, C. Merten, T. Bach, *Org. Biomol. Chem.* **2023**, *21*, 4422.

[25] J. Li, C. Fu, G. Chen, G. Chai, S. Ma, *Adv. Synth. Catal.* **2008**, *350*, 1376.
